# Phosphatidylinositol 3 kinase modulation of trophoblast cell differentiation

**DOI:** 10.1186/1471-213X-10-97

**Published:** 2010-09-14

**Authors:** Lindsey N Kent, Toshihiro Konno, Michael J Soares

**Affiliations:** 1 The Institute for Reproductive Health and Regenerative Medicine, Department of Pathology & Laboratory Medicine, University of Kansas Medical Center, Kansas City, Kansas 66160, USA

## Abstract

**Background:**

The trophoblast lineage arises as the first differentiation event during embryogenesis. Trophoblast giant cells are one of several end-stage products of trophoblast cell differentiation in rodents. These cells are located at the maternal-fetal interface and are capable of invasive and endocrine functions, which are necessary for successful pregnancy. Rcho-1 trophoblast stem cells can be effectively used as a model for investigating trophoblast cell differentiation. In this report, we evaluated the role of the phosphatidylinositol 3-kinase (PI3K) signaling pathway in the regulation of trophoblast cell differentiation. Transcript profiles from trophoblast stem cells, differentiated trophoblast cells, and differentiated trophoblast cells following disruption of PI3K signaling were generated and characterized.

**Results:**

Prominent changes in gene expression accompanied the differentiation of trophoblast stem cells. PI3K modulated the expression of a subset of trophoblast cell differentiation-dependent genes. Among the PI3K-responsive genes were those encoding proteins contributing to the invasive and endocrine phenotypes of trophoblast giant cells.

**Conclusions:**

Genes have been identified with differential expression patterns associated with trophoblast stem cells and trophoblast cell differentiation; a subset of these genes are regulated by PI3K signaling, including those impacting the differentiated trophoblast giant cell phenotype.

## Background

Hemochorial placental development is a complex process involving multiple signaling pathways. Effectively two placental compartments are established. One compartment contains trophoblast cells specialized for interactions with the maternal environment, while the other contains trophoblast cells directed toward the bidirectional transport of nutrients and wastes between the mother and the fetus. Trophoblast cells of the rat and mouse have the capacity to differentiate along a multi-lineage pathway. Cell lineages directed toward the maternal environment, include trophoblast giant cells, spongiotrophoblast, glycogen cells, and invasive trophoblast cells; whereas syncytial trophoblast regulate maternal-fetal nutrient and waste delivery [1-3]. Each lineage possesses specialized functions necessary for a normal pregnancy.

Trophoblast giant cells are the first trophoblast lineage to differentiate [4]. Trophoblast giant cells are located at the maternal-fetal interface and have several functions. They produce steroid and peptide hormones [1] and have the ability to invade into the uterine vasculature [5,6].

The phosphatidylinositol 3-kinase/protein kinase B (PI3K/AKT), pathway is involved in trophoblast cell development [7]. Upon differentiation of trophoblast cells, PI3K is activated leading to the phosphorylation and constitutive activation of AKT [7]. Inhibition of PI3K disrupts AKT activation and interferes with trophoblast cell differentiation [7,8]. The predominant isoform of AKT in developing trophoblast giant cells is AKT1 [7,9]. Mice possessing a null mutation at the *Akt1 *locus exhibit defects in placental development [9]. Their placentas are smaller and accumulate less glycogen than wild-type mice.

In this report, we utilize Rcho-1 rat trophoblast stem cells as an in vitro model to gain a better understanding of trophoblast cell differentiation. Rcho-1 trophoblast cells are remarkable in that they can be maintained in a stem cell state or induced to differentiate along the trophoblast giant cell lineage [10-13]. This in vitro system represents an excellent model for investigating regulatory pathways controlling trophoblast giant cell differentiation. In order to gain new insights about trophoblast cell differentiation we performed genome wide screens for transcripts expressed in trophoblast stem cells, differentiating trophoblast cells, and differentiating trophoblast cells with disrupted PI3K signaling. Genes selected for further analyses exhibited high levels of expression, prominent differences among the experimental groups, and/or encoded proteins with actions potentially relevant to trophoblast biology. Expression patterns of a subset of genes identified from the array were verified by northern analysis and/or quantitative RT-PCR (qRT-PCR). In vivo placental expression patterns of the selected genes identified from the gene profiles were also determined. 'Trophoblast stem cell-associated', 'differentiation-associated', and 'PI3K-regulated' genes were identified. A subset of the 'differentiation-associated' genes is regulated by the PI3K signaling pathway and may contribute to the trophoblast cell phenotype.

## Methods

### Reagents and cDNA generation

All reagents were purchased from Sigma-Aldrich (St. Louis, MO) unless otherwise noted. cDNAs to selected transcripts were obtained from Invitrogen (Carlsbad, CA), American Type Culture Collection (ATCC, Rockville, MD), or cloned using TOPO TA cloning kit (Invitrogen). Other cDNAs were gifts from the following investigators: *Atp1a1*, Dr. Gustavo Blanco, University of Kansas Medical Center (Kansas City, KS); *Cyp11a1*, Dr. JoAnne Richards, Baylor College of Medicine (Houston, TX); *Mmp9*, Dr. Ruth Muschel, University of Pennsylvania (Philadelphia, PA), and *Prl4a1*, Dr. Mary Lynn Duckworth, University of Manitoba (Winnipeg, Manitboa, Canada). Additional file 1: **Supplemental Table S1 **includes information on the source of cDNAs and primer sequences used for the generation of cDNAs and for qRT-PCR.

### Animals and tissue collection

Holtzman Sprague-Dawley rats were obtained from Harlan Laboratories (Indianapolis, IN). Animals were housed in an environmentally controlled facility with lights on from 0600-2000 h and were allowed free access to food and water. Timed pregnancies were generated by cohabitation of female and male animals. The presence of a copulatory plug or sperm in the vaginal smear was designated d0.5 of pregnancy. Rat placental tissues were collected on gestation d11.5 and d18.5. At d11.5 of gestation, the placenta contains a mixture of proliferating and differentiating trophoblast cells, while at gestation d18.5, the placenta is fully mature and comprised of differentiated trophoblast cells. D11.5 tissue samples contained all trophoblast present within the placentation site, whereas d18.5 tissue samples were restricted to the junctional zone. Placentation site dissections were performed as previously described [14]. Tissues for histological analysis were frozen in dry-ice cooled heptane and stored at -80°C. Tissue samples for RNA extraction were frozen in liquid nitrogen and stored at -80°C. The University of Kansas Animal Care and Use Committee approved protocols for the care and use of animals.

### Maintenance of Rcho-1 trophoblast stem cells

Rcho-1 trophoblast stem cells were maintained at subconfluent conditions in Stem Medium [RPMI-1640 culture medium (Cellgro, Herndon, VA) supplemented with 20% fetal bovine serum (FBS; Atlanta Biologicals, Norcross, GA) 50 μM 2-mercaptoethanol, 1 mM sodium pyruvate (Cellgro), 100 μM penicillin, and 100 U/ml streptomycin (Cellgro)] as previously reported [13,15]. Differentiation was induced by growing cells to near confluence in FBS-supplemented culture medium and then replacing the medium with Differentiation Medium [NCTC-135 medium (Sigma-Aldrich) supplemented with 1% horse serum (HS; Atlanta Biologicals), 50 μM 2-mercaptoethanol, 1 mM sodium pyruvate, 10 mM HEPES, 4-(2-hydroxyethyl)-1-piperazineethanesulfonic acid (Fisher, Pittsburgh, PA), 38 mM sodium bicarbonate (Fisher), 100 μM penicillin and 100 U/ml streptomycin (Cellgro)]. High cell density and the absence of sufficient growth stimulatory factors (removal of FBS) facilitate trophoblast giant cell formation [12,13]. Trypsin (0.25%)-ethylenediamine tetraacetic acid (EDTA, 0.1% in Hank's Balanced Salt Solution, Cellgro) was used to passage the cells. Cells in the stem cell condition were grown in Stem Medium and collected 24 h after subculture to restrict the accumulation of spontaneously differentiating cells. Cells in the differentiation condition were grown for eight days in Differentiation Medium prior to harvesting unless otherwise noted. RNA samples were extracted using TRIzol (Invitrogen) according to the manufacturer's instructions.

### Inhibition of PI3K

LY294002 (Calbiochem, La Jolla, CA) was used to inhibit PI3K [16]. For chronic treatment experiments, Rcho-1 trophoblast stem cells were grown to near confluence and then shifted to Differentiation Medium containing vehicle (0.1% final concentration of dimethyl sulfoxide, DMSO) or Differentiation Medium supplemented with LY294002 (10 μM). This LY294002 treatment regimen was based on our earlier report, which effectively disrupts PI3K signaling in Rcho-1 trophoblast cells [7]. Cells were harvested after eight days of treatment. For acute inhibition of PI3K, cells were cultured for 6-12 days in Differentiation Medium and then shifted to Differentiation Medium containing vehicle (0.1% DMSO) or LY294002 (10 μM) for 48 h. Culture medium was replaced daily.

### DNA microarray

Affymetrix 230 2.0 DNA microarray chips (Affymetrix, Santa Clara, CA) were probed with cDNAs generated from Rcho-1 trophoblast cells grown under stem or differentiation conditions with chronic exposure to LY294002 or vehicle. Each treatment group was repeated in triplicate. RNA samples were hybridized to the Affymetrix 230 2.0 DNA microarray chip using the GeneChip^® ^Hybridization Oven 640 (Affymetrix). Washing and staining of hybridized chips were conducted using the GeneChip^® ^Fluidics Station 450 (Affymetrix). Chips were scanned using the Affymetrix GeneChip^® ^Scanner 3000 (Affymetrix) with autoloader by the KUMC Biotechnology Support Facility. Hybridization signals were normalized with internal controls using the Mas5 algorithm in Expression Console (Affymetrix) and fold change computed. Significant differences were determined by paired two-tailed Student *t*-tests. Microarray data was processed for functional analysis using Ingenuity Pathway Analysis (Redwood City, CA). Expression of genes in Rcho-1 trophoblast stem cells and mouse trophoblast stem cells was compared using the "*Compare Lists of Genes*" program (http://elegans.uky.edu/MA/progs/Compare.html; Dr. James Lund, University of Kentucky, personal communication). Only genes annotated identically by Affymterix in both rat and mouse chips were included. Mouse trophoblast stem cell array data were downloaded from the Gene Expression Omnibus (GEO) database http://www.ncbi.nlm.nih.gov/geo/. TS 3.5 d0 (GSM325436) was compared to TS 3.5 d6 (GSM325442) [17]. Probe sets included in the analysis were restricted to those changing at least 1.5 fold between group comparisons with signal strengths of ≥ 800 for the maximal value.

### Northern blotting

Northern blotting analysis was performed as previously described [18]. Total RNA (20 μg) was separated in 1% formaldehyde-agarose gels and transferred to nitrocellulose membranes (Schleicher & Schuell Bioscience, Keene, NH). cDNA inserts were obtained by enzymatic digestion and labeled with [^32^P] (NEN Life Science Products, Boston, MA) using Prime-it II random primer labeling kits (Stratagene, La Jolla, CA). See Additional file 1: **Supplemental Table S1 **for information on cDNAs. Probes were incubated with the blots at 42°C overnight and washed with 2XSSPE/0.1XSDS at 42°C twice for 25 min and 1XSSPE/0.1XSDS at 50°C for 35 min. Blots were then exposed to x-ray film at -80°C. Glyceraldehyde-3-phosphate dehydrogenase (*Gapdh*) was used to assess RNA integrity and as a loading control.

### qRT-PCR

cDNAs were reverse transcribed (RT) from RNA using reagents from Promega (Madison, WI) according to the manufacturer's instructions. SYBR GREEN PCR Master Mix (Applied Biosystems, Foster City, CA) was used in the PCR reaction. Reactions were run using a 7500 Real-Time PCR System (Applied Biosystems). Conditions included an initial holding stage (50°C for 2 min and 95°C for 10 min) and 40 cycles (95°C for 15 s and 60°C for 1 min) followed by a dissociation stage (95°C for 15 s, 60°C for 1 min, and then 95°C for 15 s). Primers are listed in Additional file 1: **Supplemental Table S1**. Expression of 18 S ribosomal RNA was used as an internal control. At least four replicates were run for each condition. Samples were normalized to the control sample for each gene. Statistical comparisons of two means were evaluated with Student's *t*-test.

### In situ hybridization

mRNAs were localized in placental tissues using nonradioactive in situ hybridization as previously described [3,19]. Ten μm cryosections were prepared and stored at -80°C until used. Plasmids containing cDNAs were used as templates to synthesize sense and antisense digoxigenin-labeled riboprobes according to the manufacturer's instructions (Roche Molecular Biochemicals, Indianapolis, IN). Information on the cDNAs for probe generation is presented in Additional file 1: **Supplemental Table S1**. Tissue sections were air dried and fixed in ice cold 4% paraformaldehyde in PBS. Prehybridization, hybridization, and detection of alkaline phosphatase-conjugated anti-digoxigenin were performed as previously reported [3,19]. Images were captured using a Leica MZFLIII stereomicroscope equipped with a Leica CCD camera (Leica Microsystems GmbH, Welzlar, Germany).

### Immunocytochemistry

Rcho-1 trophoblast stem cells were cultured on chamber slides under stem, differentiation, or differentiation conditions with chronic exposure to LY294002. Cells were fixed in ice-cold 4% paraformaldehyde. Actin filaments were visualized using rhodamine-conjugated phalloidin (Molecular Probes, Eugene, OR). Nuclei were stained with 4,6'-diamidino-2-phenylindole (DAPI, Molecular Probes). Bright field and fluorescence images were captured using either Leica MZFLIII stereomicroscope or DMI 4000 microscopes equipped with CCD cameras (Leica).

### Analysis of DNA content

DNA content was estimated by flow cytometry [20]. Cells were trypsinized and fixed in 70% ethanol and then stained with propidium iodine and analyzed using a BDLSRIII flow cytometer (BD Biosciences, San Jose, CA).

### Steroid hormone measurements

Steroid radioimmunoassays (RIAs) were performed as previously reported [21]. Androstenedione and progesterone concentrations were measured in Rcho-1 trophoblast cell conditioned medium with ^125^I-labelled RIA kits (Diagnostic Products, Los Angeles, CA) and normalized to cellular DNA content. DNA samples were obtained by lysis of cells with digestion buffer containing proteinase K. Samples were then incubated at 37°C overnight and diluted 10X with water. DNA content was then measured with the PicoGreen^® ^dsDNA Quantitation Kit (Molecular Probes) according to the manufacturer's instructions. Statistical comparisons of two means were evaluated with Student's *t*-test.

## Results

### Identification of genes associated with trophoblast differentiation

**Figure 1:**
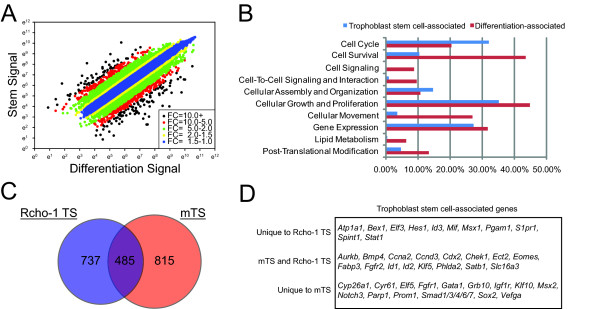
**Analysis of microarray data**. **A) **Scatter graph showing the signal intensity of probe sets. **B) **Functional analysis of 'trophoblast stem cell-associated' genes and 'differentiation-associated' genes using Ingenuity Pathway Analysis software. **C) **Comparison of 'trophoblast stem cell-associated' genes in Rcho-1 trophoblast stem cells and mouse trophoblast stem cells. **D) **Examples of 'trophoblast stem cell-associated' genes unique to or shared between Rcho-1 trophoblast stem cells and mouse trophoblast stem cells.

Phenotypes of trophoblast cells connected to distinct developmental states were assessed by DNA microarray analysis. Gene-restricted expression patterns associated with stem cell and differentiated states were identified (Fig. 1A). All DNA microarray data presented in this report are deposited in the Gene Expression Omnibus (GEO) repository under the GSE21938 accession number http://www.ncbi.nlm.nih.gov/geo/query/acc.cgi?acc=GSE21938.

### Trophoblast stem-associated genes

Approximately half of the genes differentially expressed between the stem cell- and differentiated cell-states were specific to the stem cell state, termed 'trophoblast stem cell-associated' genes. Additional file 2: **Supplemental Table S2 **shows an abbreviated list of 'trophoblast stem cell-associated' genes. Genes listed in this table are those with arbitrary expression signal strengths ≥ 800 in the stem cell condition and displaying a significantly higher level of expression in the stem cell state versus the differentiated state (≥ 1.5 fold; P ≤ 0.05). We used Ingenuity Pathway Analysis software to investigate 'trophoblast stem cell-associated' genes. Of the 1720 probe sets listed in Additional file 2: **Supplemental Table S2**, 584 genes were annotated by Ingenuity Pathway Analysis software. Functions associated with the annotated 'trophoblast stem cell-associated' genes included cellular growth and proliferation (35%), cell cycle (32%), and cellular assembly and organization (15%), (Fig. 1B; Additional file 3: **Supplemental Table S3**). Not surprisingly, the analysis indicates that a large percentage of 'trophoblast stem cell-associated' genes have functions that correlate with the proliferative phenotype of these cells.

**Figure 2:**
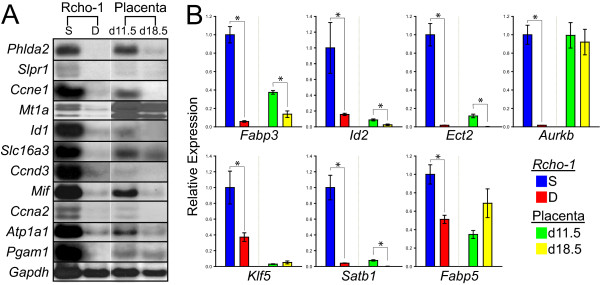
**Expression of a subset of 'trophoblast stem cell-associated' genes**. **A) **Representative northern blot analysis of 'trophoblast stem cell-associated' genes identified by DNA microarray analysis. **B) **qRT-PCR analysis of 'trophoblast stem cell-associated' genes identified by DNA microarray analysis. See Table 1 for a list and description of the mRNAs investigated. Rcho-1 trophoblast stem cells were cultured under stem (S) or differentiating (D) conditions. Rat placental samples were also included in the analysis and are from gestation d11.5 trophoblast and d18.5 junctional zone. Student's *t*-tests (*P < 0.05).

A subset of 'trophoblast stem cell-associated' genes identified from the microarray analysis was further evaluated (Table 1). Transcript levels were estimated by northern analysis or qRT-PCR in Rcho-1 trophoblast cells from stem and differentiated states. Each of the genes was expressed at higher levels in the trophoblast stem cell state (Fig. 2). Approximately half of the 'trophoblast stem cell-associated' genes showed elevated expression in midgestation versus late gestation trophoblast tissues (Fig. 2). The validated 'trophoblast stem cell-associated' genes encode proteins involved in cell cycle regulation (*Ccne1*, *Ccna2*, *Ccnd3*, *Klf5*, *Ect2*), inhibition of differentiation (*S1pr1*, *Id1, Id2*), inhibition of placental growth (*Phlda2*), and protection from cytotoxic agents (*Mt1a1*). Other 'trophoblast stem cell-associated' genes were previously detected in proliferative populations of trophoblast (*Slc16a3*, *Mif, Atp1a1*). Many of the 'trophoblast stem cell-associated' genes identified in Rcho-1 cells are also found in mouse trophoblast stem cells (Fig. 1C, D). Conspicuous among the genes unique to mouse trophoblast stem cells is *Elf5*, while *Atp1a1*, *Id3*, *Mif*, *Pgam1*, and *S1pr1 *are unique to the Rcho-1 trophoblast stem cell population (Fig. 1D).

**Table 1:** Trophoblast stem cell associated genes

Gene name	Abbreviation	Synonyms	Functional Group	**GenBank Accession No**.	Fold Change S/D	Fold Change D/D+LY
Fatty acid binding protein 3	*Fabp3*		Fatty acid binding	NM_024162	-71.41	-

Pleckstrin homology-like domain, family A, member 2	*Phlda2*	*Ipl, Tssc3*	Placental growth	NM_001100521	-48.67	-

Inhibitor of DNA binding 2	*Id2*		Transcription regulator	NM_013060	-21.03	3.53

Sphingosine-1-phosphate receptor 1	*S1pr1*	*Edg1, IpB1*	Lipid receptor	NM_017301	-16.08	-

Cyclin E	*Ccne1*	*Ccne*	Cell cycle regulator	NM_001100821	-11.52	-

Metallothionein 1a	*Mt1a*	*Mt*	Protection from oxidative stress	NM_138826	-10.75	-

Ect2 oncogene	*Ect2*		Ras signaling	NM_001108547	-10.06	-

Aurora kinase B	*Aurkb*	*Aim1, Stk12*	Kinase	NM_053749	-9.67	-

Kruppel-like factor 5	*Klf5*	*IKLF, bteb2*	Transcription regulator	NM_053394	-8.85	-

Inhibitor of DNA binding 1	*Id1*		Transcription regulator	NM_012797	-8.64	-

Solute carrier family 16 (monocarboxylic acid transporters), member 3	*Slc16a3*	*Mct3, Mct4*	Transporter	NM_030834	-6.57	-

Special AT-rich sequence binding protein 1	*Satb1*		DNA binding	NM_001012129	-6.170	-

Cyclin D3	*Ccnd3*		Cell cycle regulator	NM_012766	-6.40	-

Macrophage migration inhibitory factor	*Mif*	*GIF, Glif*	Ligand, chemokine	NM_031051	-5.32	-

Cyclin A2	*Ccna2*	*Ccn1, Ccna, Cyca*	Cell cycle regulator	NM_053702	-4.49	-

ATPase, Na+/K+ transporting, alpha 1 polypeptide	*Atp1a1*	*Nkaa1b, Atpa-1*	NA+/K+ pump	NM_012504	-3.95	-

Phosphoglycerate mutase 1	*Pgam1*	*Pgmut*	Metabolism	NM_053290	-3.03	-

Fatty acid binding protein 5, epidermal	*Fabp5*	*C-Fabp, E-Fabp*	Fatty acid binding	NM_145878	-1.43	-3.23

S/D: Stem cell state/differentiation cell state ratio

D/D+LY: Differentiation cell state/differentiation cell state + LY294002 treatment

### Trophoblast differentiation-associated genes

The second collection of genes exhibiting changes in mRNA expression is upregulated in association with differentiation and referred to as 'differentiation-associated' genes (Additional file 4: **Supplemental Table S4**). Genes listed in this table are those with arbitrary expression signal strengths ≥ 800 in the differentiated cell condition and displaying a significantly higher level of expression in the differentiated cell state versus the stem cell state (≥ 1.5 fold; P ≤ 0.05). Of the 1585 probe sets listed in Additional file 4: **Supplemental Table S4**, 537 genes were annotated by Ingenuity Pathway Analysis software. Functions associated with the annotated 'differentiation-associated' genes included cellular growth and proliferation (45%), cell survival (43%), gene expression (32%), cellular movement (27%), and lipid metabolism (6%) (Fig. 1B; Additional file 3: **Supplemental Table S3**). Many of the genes associated with the cellular growth and proliferation classification encode growth factors, cytokines, and peptide hormones (e.g. *Igf2, Grn*, members of the prolactin, PRL, family, etc); and represent features of the endocrine phenotype of trophoblast giant cells. Genes linked to cell movement and lipid metabolism, include those encoding proteins contributing to the invasive and steroid hormone producing phenotypes of trophoblast giant cells.

**Figure 3:**
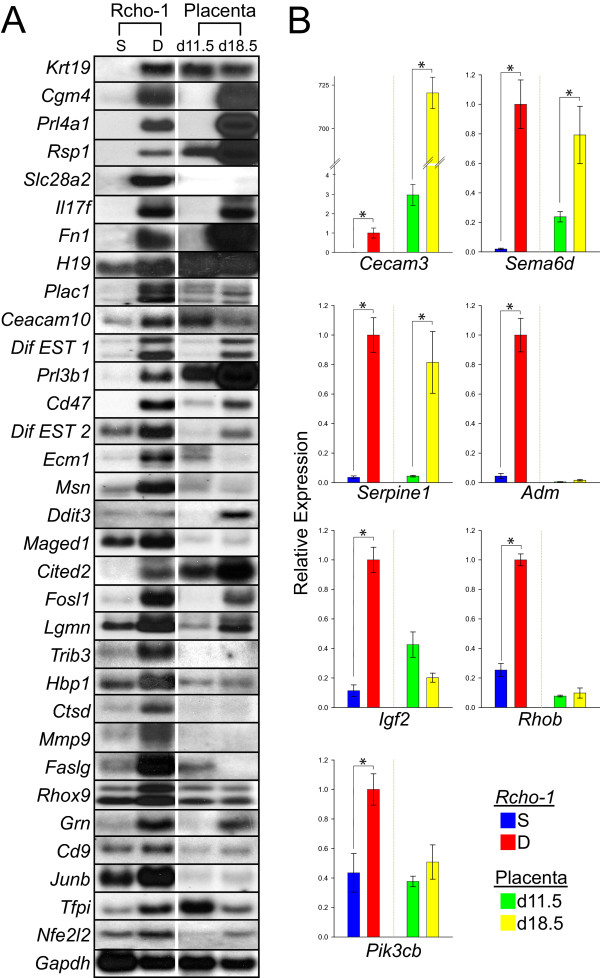
**Expression of a subset of trophoblast 'differentiation-associated' genes**. **A) **Representative northern blot analysis of trophoblast 'differentiation-associated' genes identified by DNA microarray analysis. **B) **qRT-PCR analysis of trophoblast 'differentiation-associated' genes identified by DNA microarray analysis. See Table 2 for a list and description of the mRNAs investigated. Rcho-1 trophoblast stem cells were cultured under stem (S) or differentiating (D) conditions. Rat placental samples were also included in the analysis and are from gestation d11.5 trophoblast and d18.5 junctional zone. Student's *t*-tests (*P < 0.05).

A sampling of 'differentiation-associated' genes identified from the microarray analysis was further examined (Table 2). Transcript levels were estimated by northern analysis or qRT-PCR in Rcho-1 trophoblast cells from stem and differentiated states. Each of the genes was expressed at higher levels in the differentiated cell state (Fig. 3). Most of the 'differentiation-associated' genes were detected in placental tissues and approximately half showed elevated expression in late gestation versus midgestation trophoblast tissues (Fig. 3). Several of the validated 'differentiation-associated' genes (Table 2; Fig. 3) have been previously reported as upregulated during trophoblast giant cell development, while others have not been associated with trophoblast lineages (e.g. *Rsp1, Sema6 d, Ceacam10, Cd47, Maged1, Trib3, Hbp1 *and *Pik3cb*). Functions of the 'differentiation-associated' genes have been connected to the regulation of cell movement and invasion (*Serpine1*, *Adm*, *Msn*, *Maged1*, *Cited2*, *Fosl1*, *Ifg2*, *Hbp1*, *Mmp9*, *Grn*, *Cd9*), interactions with maternal immune and vascular systems, (*Cgm4*, *Prl4a1*, *Cd47, Ecm1*, *Ctsd, Faslg*, *Grn*, *Cd9*, *Tfpi*), and the endocrine phenotype of trophoblast giant cells (PRL family and steroid biosynthesis).

**Table 2:** Trophoblast differentiation associated genes

Gene name	Abbreviation	Synonyms	Functional Group	**GenBank Accession No**.	Fold Change D/S	Fold Change D/D+LY
Keratin complex 1, acidic, gene 19	*Krt19*	*EndoC, K19*	Cytoskeletal protein	NM_199498	149.07^t^	-

Carcinoembryonic antigen gene family 4	*Cgm4*	*Psg16, Psg38*	Secretory protein, unknown function	NM_012525	89.73	-2.79

Carcinoembryonic antigen-related cell adhesion molecule 3	*Ceacam3*	*Cgm1*	Cell adhesion molecule	NM_012702	88.71	-1.75

Cytochrome P450, family 11, subfamily a, polypeptide 1	*Cyp11a1*	*P450scc*	Steroidogenic enzyme	NM_017286	47.11	-

Prolactin family 4, subfamily a, member 1	*Prl4a1*	*PLP-A*	Ligand/cytokine	NM_017036	33.05	-3.91

Spleen protein 1 precursor	*Rsp1*	*Sslp1, LOC171573*	Unknown	NM_138537	30.29	-4.43

Solute carrier family 28 (sodium-coupled nucleoside transporter), member 2	*Slc28a2*	*Cnt2*	Transporter, nucleotide	NM_031664	23.94	-1.71

Interleukin 17F	*Il17f*	*ML1*	Ligand/cytokine	NM_001015011	22.49	-2.61

Fibronectin 1	*Fn1*	*Fn*	Extracellular matrix protein	NM_019143	19.68	-

H19 fetal liver mRNA	*H19*	*ASM1*	Unknown	NR_027324	13.10	-

Cytochrome P450, family 17, subfamily a, polypeptide 1	*Cyp17a1*	*Cyp17, p450c17*	Steroidogenic enzyme	NM_012753	10.76	-4.57

Hydroxysteroid (17-beta) dehydrogenase 2	*Hsd17b2*		Steroidogenic enzyme	NM_024391	10.56	--

Placenta-specific 1	*Plac1*	*Epcs26*	Cell-cell comunication	NM_001024894	9.62	--

CEA-related cell adhesion molecule 10	*Ceacam10*	*C-CAM4*	Cell adhesion molecule	NM_173339	8.66	-1.86

Differentaly expressed X chromosome EST 1	*Dif EST 1*			AI012949	8.61	--

Prolactin family 3, subfamily b, member 1	*Prl3b1*	*PL-II, Csh2*	Ligand/cytokine	NM_012535	8.38	-7.73

CD47 antigen (Rh-related antigen, integrin-associated signal transducer)	*Cd47*	*IAP, Itgp*	Receptor, thrombospondin	NM_019195	7.90	-

Sema domain, transmembrane domain (TM), and cytoplasmic domain, (semaphorin) 6D	*Sema6d*		Receptor	NM_001107768	8.38	-1.56

Differentaly expressed X chromosom EST 2	*Dif EST 2*	LOC681066		AA964255	7.40	-

Serine (or cysteine) peptidase inhibitor, clade E, member 1	*Serpine1*	*Pai1, Planh*	blood coagulation, angiogenesis	NM_012620	6.41	-3.95*

Extracellular matrix protein 1	*Ecm1*		Extracellular protein	NM_053882	6.11	-

Adrenomedullin	*Adm*		Hypotensive peptide	NM_012715	5.81	-14.40

Moesin	*Msn*		Cell-cell comunication	NM_030863	5.66	-

DNA-damage inducible transcript 3	*Ddit3*	*Chop10, Gadd153*	Cell stress/death	NM_001109986	5.65	-1.86

Melanoma antigen, family D, 1	*Maged1*	*Nrage, Dlxin1*	Apoptosis, cell cycle, transcription	NM_053409	5.61	-

Cbp/p300-interacting transactivator, with Glu/Asp-rich carboxy-terminal domain, 2	*Cited2*	*Mrg1, Msg2, p35srj*	Transcription regulator	NM_053698	5.49	-

Hydroxy-delta-5-steroid dehydrogenase, 3 beta- and steroid delta-isomerase 1	*Hsd3b1*		Steroidogenic enzyme	NM_001007719	5.15	-

Fos-like antigen 1	*Fosl1*	*Fra1*	Transcription regulator	NM_012953	5.04	-

Legumain	*Lgmn*	*Prsc1*	Putative cysteine protease	NM_022226	4.73	-

Insulin-like growth factor 2	*Igf2*	*Igf-II*	Ligand, growth factor	NM_031511	4.38	-3.74

Tribbles homolog 3 (Drosophila)	*Trib3*	*Trb3, Ifld2, Nipk*	Metabolism	NM_144755	4.37	-2.09

High mobility group box transcription factor 1	*Hbp1*	*HMGB1*	Transcription regulator	NM_013221	4.36	-

Cathepsin D	*Ctsd*	*CD, CatD*	Lysosomal aspartic endopeptidase	NM_134334	4.13	-3.00

Matrix metallopeptidase 9	*Mmp9*	*Gelatinase B*	Extracellular matrix remodeling	NM_031055	4.00	-3.11

Fas ligand (TNF superfamily, member 6)	*Faslg*	*Faslg, Tnfsf6*	Ligand/membrane anchored	NM_012908	3.80	-3.06

Reproductive homeobox on X chromosome, 9	*Rhox9*	*Gpbox, Psx2*	Transcription regulator	NM_001024874	3.43	-

Granulin	*Grn*	*Gep, Pcdgf, Pgrn*	Ligand, growth factor	NM_017113	3.39	-

CD9 antigen	*Cd9*	*Tspan29*	Cell surface glycoprotein	NM_053018	3.14	-

RhoB gene	*Rhob*	*Arhb*	Ras family	NM_022542	3.07	2.08*

Phosphatidylinositol 3-kinase, catalytic, beta polypeptide	*Pik3cb*		Inositol lipid kinase	NM_053481	2.75	-

Kruppel-like factor 2 (lung)	*Klf2*		Transcription regulator	NM_001007684	2.73	4.70

Jun-B oncogene	*Junb*		Transcription regulator	NM_021836	2.35	-

Tissue factor pathway inhibitor	*Tfpi*	*EPI, LACI*	Kunitz family serine protease inhibitor	NM_017200	2.20	-

Nuclear factor, erythroid derived 2, like 2	*Nfe2l2*	*Nrf2*	Transcription regulator	NM_031789	2.09	-

S/D: Stem cell state/differentiation cell state ratio

D/D+LY: Differentiation cell state/differentiation cell state + LY294002 treatment

* Fold change values based on qRT-PCR analysis

^t ^Not significant by student t-test

**Figure 4:**
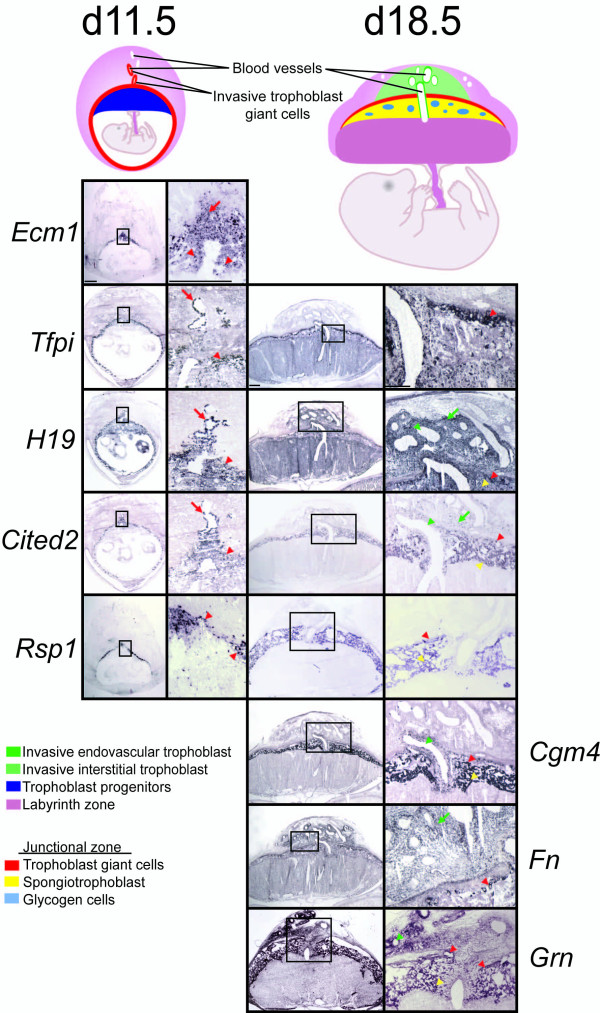
**'Differentiation-associated' genes are expressed by trophoblast cells developing within the chorioallantoic placenta**. In situ detection of mRNA expression of 'differentiation-associated' genes in gestational d11.5 and d18.5 rat placentation sites is presented. Bars = 1 mm. Left panels, d11.5 placentation sites, far left: low magnification; middle left: high magnification of boxed area. Right panels, d18.5 placentation sites, middle right: low magnification; far right: high magnification of boxed area. Red arrowheads, trophoblast giant cells; red arrows, invasive trophoblast giant cells; yellow arrows, spongiotrophoblast; green arrowheads, endovascular invasive trophoblast; green arrows, interstitial invasive trophoblast.

A subset of 'differentiation-associated' mRNAs highly expressed in rat placental samples (Fig. 3) was localized to the placentation site via in situ hybridization (Fig 4). 'Differentiation-associated' transcripts were all found in trophoblast giant cells and in most instances other trophoblast lineages. *Ecm1 *mRNA is expressed in trophoblast giant cells and some progenitor trophoblast cells on gestation d11.5. *Tfpi, Cited2*, and *Rsp1 *transcripts were localized to trophoblast giant cells on gestation d11.5, including those penetrating into the uterine spiral arterioles. On gestation d18.5, *Tfpi, Cited2*, and *Rsp1 *were also identified in spongiotrophoblast. *Cgm4 *and *Grn *transcripts were expressed in trophoblast giant cells, spongiotrophoblast, and invasive trophoblast cells on gestation d18.5. *H19 *mRNA was expressed in all trophoblast lineages on gestation d11.5 and d18.5. *Fn *mRNA was expressed in all trophoblast lineages on d18.5.

### PI3K signaling and trophoblast differentiation

**Figure 5:**
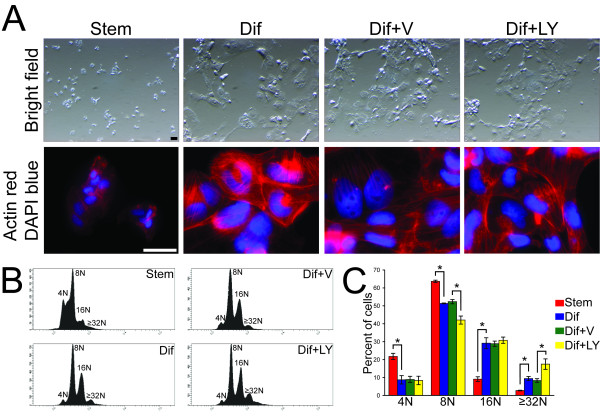
**PI3K impact on trophoblast cell differentiation: morphology and DNA content**. Morphology and DNA content were assessed in Rcho-1 trophoblast stem cells cultured in the following conditions: stem (Stem), differentiating (Dif), differentiating with vehicle exposure (0.1% DMSO; Dif+V), or LY294002 (10 μM; Dif+LY). **A) **Morphology was determined by bright field microscopy (top panels). Actin filaments were stained with rhodamine-conjugated phalloidin; nuclei were visualized with DAPI (bottom). Bar= 50 μm. **B) **DNA content was estimated by propidium iodine staining followed by flow cytometry. Due to the tetraploid nature of the Rcho-1 trophoblast cells, 4N and 8N cell populations correspond to dividing cells, whereas cell populations with more than 8N DNA content have undergone endoreduplication. **C) **Graphic representation of three independent flow cytometry experiments. Student's t-tests (*P < 0.05).

**Figure 6:**
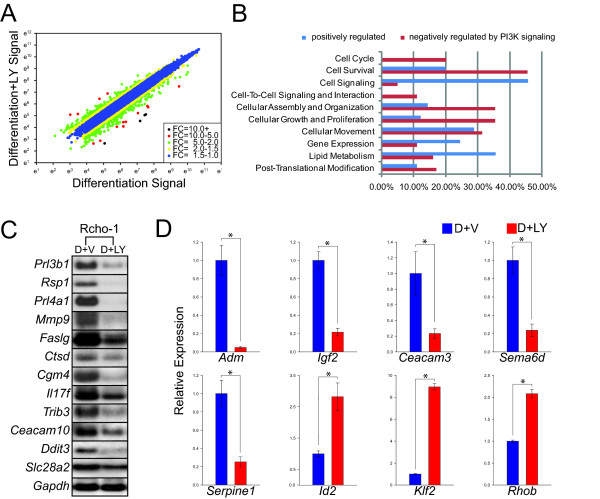
**PI3K regulation: Analysis of microarray data and expression of a subset of PI3K regulated genes**. **A) **Scatter graph showing the signal intensity of probe sets. **B) **Functional analysis of negatively and positively regulated genes by PI3K using Ingenuity Pathway Analysis software. **C) **Representative northern blot analyses of genes regulated by PI3K originally identified by DNA microarray analysis. **D) **qRT-PCR analysis of genes regulated by PI3K originally identified by DNA microarray analysis. See Tables 1 and 2 for a list and description of the mRNAs investigated. Rcho-1 trophoblast stem cells were cultured under differentiating conditions with exposure to vehicle (0.1% DMSO; D+V) or LY294002 (10 μM; D+LY). Student's *t*-tests (*P < 0.05).

The PI3K signaling pathway has been implicated in the regulation of trophoblast differentiation [7,8] and was further investigated in this report. Initially we examined the effect of disruption of PI3K during trophoblast differentiation on the distribution of actin filaments and DNA content (Fig. 5). Actin filaments were not significantly affected by the PI3K inhibitor treatment regimen used (LY294002, 10 μM; Fig. 5A). However, inhibition of PI3K did affect ploidy. Disruption of PI3K resulted in a significant fraction of cells with increased DNA content, and thus the generation of giant cells with elevated ploidy levels (Fig. 5B, C). The findings suggest that PI3K restricts the formation of trophoblast giant cells with high ploidy levels (> 32N). Higher concentrations of PI3K inhibitors interfere with actin filament distributions and cell survival (data not shown). Phenotypes of differentiating trophoblast cells treated with the PI3K inhibitor (LY294002, 10 μM) or vehicle were also assessed by DNA microarray analysis. Some genes identified were negatively regulated and others positively regulated by PI3K signaling (Fig. 6A).

### PI3K signaling: negatively regulated genes

The 'negatively regulated' PI3K dependent genes are diverse in their expression patterns (Additional file 5: **Supplemental Table S5**). Some are 'trophoblast stem cell-associated' genes, others are 'differentiation-associated' genes, while still others were not affected by differentiation state. Genes listed in Additional file 5: **Supplemental Table S5 **are those with arbitrary expression signal strengths ≥ 800 in the differentiated cell condition and displaying a significantly lower level of expression in the differentiated cell state versus the differentiated cell state treated with the PI3K inhibitor (≥ 1.5 fold; P ≤ 0.05). Of the 257 probe sets listed in Additional file 5: **Supplemental Table S5**, 99 genes were annotated by Ingenuity Pathway Analysis software. Functions associated with the annotated 'negatively regulated' genes included cell survival (45%), cellular assembly and organization (35%), cellular growth and proliferation (35%), cellular movement (31%), and lipid metabolism (16%) (Fig. 6B; Additional file 3: **Supplemental Table S3**). These functions overlap with those observed for both the 'trophoblast stem cell associated' and 'differentiation-associated' gene profiles (Fig. 1). Of the sixteen validated 'trophoblast stem cell-associated' genes only *Id2 *was regulated by PI3K signaling (Fig 6D). *Klf2 *and *Rhob *expression was not affected by differentiation state but was negatively regulated by PI3K (Fig. 6D).

### PI3K signaling: positively regulated genes

**Figure 7:**
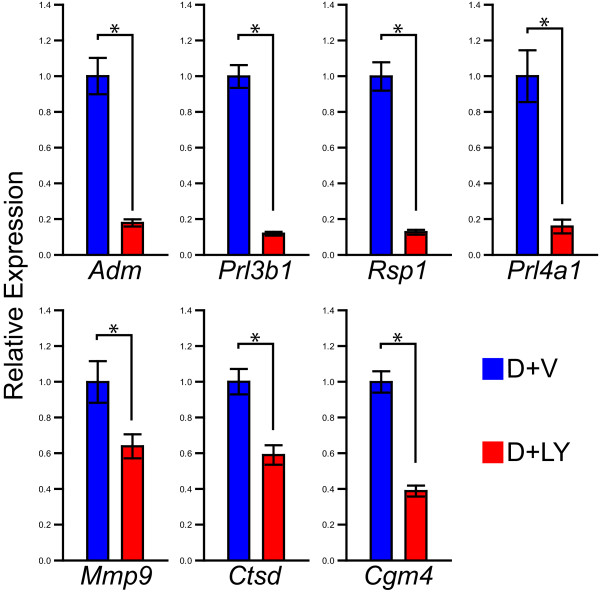
**Acute inhibition of PI3K is sufficient to regulate expression of PI3K sensitive genes**. qRT-PCR analysis of trophoblast 'differentiation-associated' genes regulated by PI3K under acute PI3K inhibition. Rcho-1 trophoblast stem cells were cultured under differentiating conditions for 6 days and for an additional 48 h of treatment with vehicle (0.1% DMSO; D+V) or LY294002 (10 μM; D+LY). Student's *t*-tests (*P < 0.05).

The majority of 'positively regulated' PI3K dependent genes are included in the 'differentiation-associated' gene set. Genes listed in Additional file 6: **Supplemental Table S6 **are those with arbitrary expression signal strengths ≥ 800 in the differentiated cell condition and displaying a significantly higher level of expression in the differentiated cell state versus the differentiated cell state treated with the PI3K inhibitor (≥ 1.5 fold; P ≤ 0.05). Of the 226 probe sets listed in Additional file 6: **Supplemental Table S6**, 90 genes were annotated by Ingenuity Pathway Analysis software. Functions associated with the annotated 'positively regulated' genes included cell survival (46%), gene expression (36%), cellular growth and proliferation (29%), small molecule biochemistry (27%), cellular development (26%), cellular movement (24%), and lipid metabolism (11%) (Fig. 6B; Additional file 3: **Supplemental Table S3**). These results are similar to that observed for the 'differentiation-associated' data set (Fig. 1; Additional file 3: **Supplemental Table S3**). Not all 'differentiation-associated' genes are regulated by PI3K suggesting that other signaling pathways contribute to the regulation of trophoblast differentiation. A subset of the 'positively regulated' PI3K-dependent genes identified from the microarray analysis was further evaluated by northern analysis or qRT-PCR in Rcho-1 trophoblast cells treated with the PI3K inhibitor or vehicle (Fig. 6C, D). The 'differentiation-associated' genes sensitive to PI3K regulation have potential roles in cell invasion (e.g. *Igf2, Mmp9, Serpine1*), immune and vascular cell regulation (e.g. *Adm*, *Cgm4, Ceacam 3, Ceacam10, Prl4a1, Il17f, Ctsd, Faslg, Sema6d*), and the endocrine phenotype of trophoblast giant cells (e.g. *Prl3b1*, *Prl4a1*, *Cyp17a1*). In an additional experiment, we sought to determine whether the effects of the PI3K inhibitor on trophoblast gene expression required exposure throughout the differentiation process or whether the inhibitor could act acutely to affect differentiated trophoblast cell function. Several of the 'differentiation-associated' genes were also sensitive to acute disruption of the PI3K signaling pathway (Fig. 7).

### PI3K regulation of trophoblast steroidogenesis

**Figure 8:**
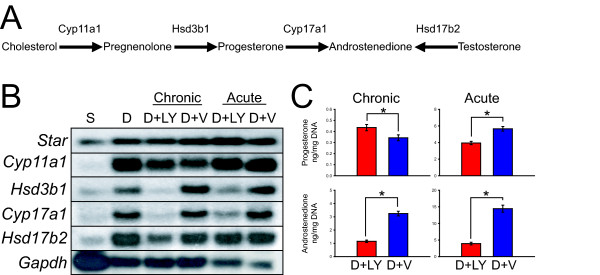
**PI3K regulates steroidogenic potential of trophoblast cells**. **A**) Overview of the steroidogenic pathway in trophoblast giant cells. **B) **Representative northern blot analysis of genes encoding components of the steroidogenic pathway in Rcho-1 trophoblast cells. Rcho-1 trophoblast cells were cultured under stem (S), differentiating (D) and differentiating with chronic or acute exposure to LY294002 (10 μM; D+LY) or vehicle (0.1% DMSO; D+V) conditions. **C) **Progesterone and androstenedione concentrations were measured by RIA in conditioned medium from Rcho-1 trophoblast stem cells cultured in differentiating conditions with chronic or acute exposure to LY294002 (10 μM D+LY) or vehicle (0.1% DMSO; D+V). Acute conditions consisted of 12 days of differentiation and an additional 48 h of treatment with vehicle or LY294002 treatment. Steroid measurements were normalized to DNA content. Student's *t*-tests (*P < 0.05).

Trophoblast giant cells are known sites for the biosynthesis of progesterone and androstenedione [12,21]. Several genes encoding proteins involved in the biosynthesis of steroid hormones are upregulated during trophoblast differentiation (Additional file 4: **Table S4; **Fig. 8). These include *Star*, which encodes a protein involved in transporting cholesterol to the mitochondria, and a series of genes encoding enzymes responsible for the production of progesterone and androstenedione (*Cyp11a1, Hsd3b1, Cyp17a1, Hsd17b2*; Fig. 8A). *Hsd3b1 *and *Cyp17a1 *expression were positively regulated by PI3K signaling (Fig. 8B). Consistent with this observation, the production of androstenedione by differentiating trophoblast cells was also dependent upon PI3K (Fig. 8C).

## Discussion

Organization of the hemochorial placenta is the result of signaling pathways directing the expansion and differentiation of trophoblast stem cell and progenitor cell populations. This decision-making culminates in the systematic activation and inactivation of gene networks within trophoblast cell populations and elaboration of specific functions that facilitate redirection of resources from the mother to the fetus. In this report, we utilized the Rcho-1 trophoblast stem cell model and induced differentiation through increased cell density and removal of growth stimuli. The growth factor deprivation may also lead to activation of stress pathways, which have been shown to influence trophoblast differentiation [22]. Using this strategy, we have identified genes associated with trophoblast stem cell expansion, differentiation, and those impacted by the PI3K signaling pathway.

### Trophoblast stem cell-associated genes

Stem cells possess the potential to proliferate and to differentiate. Several genes implicated in maintenance of the trophoblast stem cell state were identified in Rcho-1 trophoblast stem cells and are similarly present in mouse trophoblast stem cells. These include an assortment of genes implicated as cell cycle regulators in numerous cell types and also genes that have been more specifically shown to have a role in the specification and maintenance of trophoblast stem cells (e.g. *Cdx2*, *Eomes*, *Id1*, *Id2*) [23-25].

*Phlda2 *displayed one of the most striking differences in its expression profile in stem versus differentiated cells. It was high in stem cells and virtually undetectable following differentiation, which is also found in mouse trophoblast stem cells. *Phlda2 *is intriguing for a number of reasons. *Phlda2 *is an imprinted gene exhibiting maternal allele-specific expression in extraembryonic and embryonic structures and in postnatal tissues, including the kidney [26,27]. In the mouse, disruption of the *Phlda2 *gene leads to placental overgrowth, while overexpression of *Phlda2 *results in placental growth restriction [28-30]. Given that PHLDA2 restrains placental growth it seems counter-intuitive that it would be abundantly expressed in stem cell populations. Insights will likely be forthcoming when more is learned about the cellular actions of PHLDA2. The activities of PHLDA2 may be linked to its pleckstrin homology domain and ability to bind phosphoinositides and could include an intracellular signal transduction function [31].

Some differences in the behavior of mouse trophoblast stem cells and Rcho-1 trophoblast stem cells are noteworthy. *Elf5*, a member of the ETS transcription factor family and a player in the derivation and maintenance of mouse trophoblast stem cells [32-34] is not among the 'trophoblast stem cell-associated' genes of the Rcho-1 trophoblast stem cell model. This may relate to differences in the requirements for exogenous factors to maintain trophoblast stem cell populations. Mouse trophoblast stem cells are dependent upon fibroblast growth factor-4 (FGF4)/FGF receptor 2 signaling [35], whereas maintenance of Rcho-1 trophoblast stem cells does not require FGF4 [10]. Evidence indicates that ELF5 may be a downstream effector of FGF4 signaling needed to sustain activation of *Cdx2 *and *Eomes *genes and the trophoblast stem cell state [33]. The requirement for *Elf5 *must in some way be circumvented in Rcho-1 trophoblast stem cell maintenance. In addition to Rcho-1 trophoblast stem cells other recently derived trophoblast cell lines from the rat and common vole also grow in the absence of exogenous FGF4 [36,37]. These observations do not reflect a fundamental species difference in the regulation of trophoblast stem cells. FGF4-dependent trophoblast stem cell lines can be established from the rat blastocyst (K. Asanoma and M.J. Soares, unpublished data). Instead, the FGF4 independence of the trophoblast stem cell populations is probably the consequence of genetic and/or epigenetic modifications and in vitro selection.

Several 'trophoblast stem cell-associated genes' were not shared with mouse trophoblast stem cells. Among these genes were *Mif *and *S1pr1*. *Mif *encodes a pro-inflammatory cytokine implicated in the regulation of angiogenesis [38], the migration and adhesion of monocytes [39], and modulation of uterine natural killer cell cytolytic activity [40]. *S1pr1 *encodes a Gi protein-coupled receptor for sphingosine 1-phosphate (S1P). S1P has been implicated in a range of functions, including controlling cell proliferation and differentiation [41]. In human trophoblast, S1P inhibits differentiation [42]. Activation of some of the 'trophoblast stem cell-associated' genes may represent a developmental progression beyond the trophoblast stem cell state exhibited by mouse trophoblast stem cells or alternatively may provide Rcho-1 cells with their tumorigenic features [18,43].

### Trophoblast differentiation-associated genes

'Differentiation-associated' genes possess a broader range of functions than noted for the 'trophoblast stem cell-associated' gene cluster. Many of these genes are characteristic of the trophoblast giant cell phenotype. The trophoblast giant cell is conspicuous in its location at the maternal-fetal interface and its functions are in large part directed toward uterine structures and in facilitating maternal adaptations to pregnancy. These functions include endocrine activities (PRL family and steroidogenesis) and intrauterine invasion and modulation of the maternal vasculature and immune cells (*Il17f, Tfpi, Cgm4, Ecm1, Cd47, Fn, Lgmn, Mmp9, Grn, Igf2*).

Among the 'differentiation-associated' genes was a subgroup of genes encoding transcriptional regulators (*Hbp1, Ddit3, Rhox9, Nrf2, Fosl1, Junb, Cited2*). Mouse mutagenesis experimentation has implicated a few of these genes (*Fosl1*, *Junb*, *Cited2*) as regulators of placental development [44-46]. However, the specific roles of FOSL1, JUNB. CITED2, and the other transcriptional regulators in the regulation of trophoblast differentiation are yet to be determined. Some may participate in the regulation or maintenance of the differentiated trophoblast cell phenotype.

There is a connection between the 'differentiation-associated' genes and the PI3K/AKT signaling pathway. As trophoblast stem cells differentiate, the PI3K/AKT signaling pathway becomes constitutively activated [7]. IGF2 and GRN are candidate autocrine activators of the PI3K/AKT signaling pathway [47,48]. *Trb3 *and *Msn *were also classified as 'differentiation-associated' genes. They encode proteins with potential roles downstream of PI3K/AKT signaling pathway [49,50].

### PI3K signaling-sensitive genes

PI3K regulates the phenotype of differentiating trophoblast cells [7]. Endoreduplication and/or survival of trophoblast giant cells are influenced by PI3K signaling. An active PI3K pathway favors trophoblast giant cells with lower ploidy levels. These cells may be more motile and phenotypically resemble midgestation trophoblast lining uterine spiral arteries [51]. PI3K signaling also possesses dramatic effects on gene expression patterns.

Overall, the functions of the PI3K-sensitive genes are biologically less diverse. Most interestingly, they include genes encoding proteins potentially impacting trophoblast invasion (*Mmp9*, [52]; *Igf2*, [53-55]; *Serpine1*, [56-58]), directed to the maternal uterine environment influencing immune and vascular cells (*Cgm4, Faslg, Prl4a1, Adm, Il17f*), and also regulating androgen biosynthesis (*Hsd3b1, Cyp17a1*).

*Cgm4 *is one of the most abundant genes expressed by differentiating trophoblast cells. It encodes a member of the expanded pregnancy specific glycoprotein (PSG) family called PSG16. PSGs act on immune cells, potentially through CD9, to influence cytokine production [59-62]; they also target the vasculature and modulate endothelial cell function [63]. The presence of *Cd9 *in differentiating trophoblast cells implies that PSGs may also possess autocrine/paracrine actions on trophoblast development, which may include regulating the trophoblast invasive phenotype [63].

FAS ligand (FASLG), PRL-like protein A (PLP-A; *Prl4a1*), adrenomedullin (ADM), and interleukin 17f (IL17F) are cytokines produced by differentiating trophoblast that are exquisitely sensitive to PI3K regulation. FASLG binds to the FAS receptor and can initiate cell death. Trophoblast derived FASLG has been implicated as a modulator of intraplacental immune cell trafficking [64,65] and is hypothesized to be a key participant in uterine spiral arteriole remodeling [66,67]. PLP-A targets natural killer cells and contributes to placentation site-specific adaptations to physiological stressors [3,68,69]. ADM may possess an autocrine role regulating trophoblast invasion [70] but also probably affects the uterine vasculature by regulating vessel diameter, permeability, and angiogenesis [71-73]. Insights about IL17F and its potential role at the placentation site are limited. IL17F is proinflammatory with prominent effects on immune and vascular cells [74-76]. Whether IL17F contributes to the organization of the hemochorial placentation site remains to be determined.

Key components of the enzymatic machinery required for trophoblast cell androgen biosynthesis are positively regulated by PI3K, including 17α hydroxylase (encoded by *Cyp17a1*). Trophoblast giant cells are sites of androstenedione biosynthesis [77,78]. Androstenedione can serve as a prohormone for the biosynthesis of estrogens and more potent androgens, such as testosterone. Estrogens possess a vital luteotropic role essential for the maintenance of pregnancy [78]. Differentiating rodent trophoblast cells also express 17β hydroxysteroid dehydrogenase type 2 (encoded by *Hsd17b2*), which is responsible for converting testosterone to less biologically potent androgens, thereby protecting the fetus from excessive androgen exposure [79,80]. Thus, PI3K signaling has a vital role in determining the steroid hormone milieu at the maternal-fetal interface.

## Conclusions

In summary, the PI3K signaling pathway regulates the differentiated trophoblast cell phenotype. Under the direction of the PI3K signaling pathway, trophoblast cells produce a battery of cytokines and hormones. These extracellular signals modulate intrauterine immune and inflammatory cells, regulate vascular remodeling, and collectively ensure a successful pregnancy.

## Authors' contributions

LNK participated in the design of the experimentation and conducted the majority of the experiments. TK contributed to in situ hybridization and histological analyses. MJS contributed to the design and coordination of the study. All authors contributed to the preparation of the manuscript and read and approved the final version for submission.

## Supplementary Material

Additional file 1 Table S1: Plasmid source and primers. Click here for file

Additional file 2 Table S2: Trophoblast stem-associated genes. Click here for file

Additional file 3 Table S3: Gene functions. Click here for file

Additional file 4 Table S4: Trophoblast differentiation-associated genes Click here for file

Additional file 5 Table S5: PI3K signaling: negatively regulated genes Click here for file

Additional file 6 Table S6: PI3K signaling: positively regulated genes Click here for file
