# Supplementary material for: Phosphatidylinositol 3 kinase modulation of trophoblast cell differentiation
Source: BMC Dev Biol. 2010 Sep 14;10:97. doi: 10.1186/1471-213X-10-97 (PMC2944162; doi:10.1186/1471-213X-10-97)
Supplement: Additional file 1 — Table S1: Plasmid source and primers. [file 1471-213X-10-97-S1.PDF]

Table S1. Plasmid source and primers

Symbol	GenBank Accession No.	Est ID	Vendor	Cloning Primers Fwd	Cloning Primers Rev	qRT-PCR Primers Fwd	qRT-PCR Primers Rev	Other Sources
Adm	NM_012715					5'acgtctcggactttctgctt3'	5'gctgctggacgcttgtagtt3'	
Atp1a1	NM_012504							Dr Blanco, University of Kansas Medical Center
Aurbk	NM_053749					5'catccctgaggaggaagacca3'	5'actgtggctagggctctcaa3'	
Ccna2	NM_053702			5'tgttgaatcaccccatgcta3'	5'agccaagtcaaaagcaagga3'			
Ccnd3	NM_012766	BI291261	Invitrogen					
Ccne1	NM_001100821	BE112508	Invitrogen					
Cd47	NM_019195	AI044792	Invitrogen					
Cd9	NM_053018	BF555259	Invitrogen					
Ceacam10	NM_173339	BF556893	Invitrogen					
Ceacam3	NM_012702					5'tggtacaaagggctgacaaa3'	5'tccacaggtcaaagtggagaa3'	
Cgm4	NM_012525	BI275410	Invitrogen			5'tagcccgatacagaacagcaa3'	5'agggtcacagcatgaggaaa3'	
Cited2	NM_053698	AA900476	Invitrogen					
Ctsd	NM_134334			5'gccaagtttgatggcatctt3'	5'atgaagccactcaggcagat3'	5'tacctgaacgtcacccgaaa3'	5'caggctggacaccttctcac3'	
Cyp11a1	NM_017286							Dr. JoAnne Richards, Baylor College of Medicine, Oonk RB et al 1989. J Biol Chem 264: 21934-21942
Cyp17a1	NM_012753			5'atgtgggaacttgtgggtctc3'	5'tgtggcctctaggtgctaac3'			
Ddit3	NM_001109986	BF395052	Invitrogen					
Ecm1	NM_053882			5'ccagaagacatccctgtgta3'	5'ttccatgtgcaagtgtggtt3'			
Ect2	NM_001108547					5'acgtcagaggagcttccaaa3'	5'ccctctgagctatgggatga3'	
Fabp3	NM_024162					5'tttgacgaggtcacagcaga3'	5'acattgccatgggtgagagt3'	
Fabp5	NM_145878					5'tggccaaaccagactgcatcat3'	5'tcgtcttcaccgtgctctcagt3'	
Fn1	NM_019143	BI292076	Invitrogen					
Fosl1	NM_012953			5'gtgcagaaaccgaagaaagg3'	5'cctcacaaagccaggagtgt3'			
Grn	NM_017113	BF5555139	Invitrogen					
H19	NR_027324	BE117886	Invitrogen					
Hbp1	NM_013221	AA891261	ATCC					
Hsd17b2	NM_024391			5'ttctctgcaaagcctggagt3'	5'aacaccttggtgacctcgac3'			
Hsd3b1	NM_001007719	BF559905	Invitrogen					
Id1	NM_012797	BI292304	Invitrogen					
Id2	NM_013060					5'gacatcagcatcctgtcctt3'	5'tctcctggtgaaatggttgat3'	
Igf2	NM_031511					5'ggaagtcgatgttggtgctt3'	5'cttgcccacggggtatct3'	
Il17f	NM_001015011			5'tggtcaagtctctgctgctg3'	5'ttcggtatgtggctttgtca3'			

Table S1. Plasmid source and primers

Symbol	GenBank Accession No.	Est ID	Vendor	Cloning Primers Fwd	Cloning Primers Rev	qRT-PCR Primers Fwd	qRT-PCR Primers Rev	Other Sources
Junb	NM_021836			5'atgtgcacgaaaatggaaca3'	5'gcaggcacataggagggata3'			
Klf2	NM_001007684					5'cctgtgaccgaggagaacaa3'	5'ccggctccgggtagtaga3'	
Klf5	NM_053394					5'cacctcagcttcctccagtt3'	5'tacgcatggtctctgggatt3'	
Krt19	NM_199498	BI279605	Invitrogen					
Lgmn	NM_022226			5'attaccgacaccaggcagac3'	5'cccatactgcatgacgtgac3'			
Maged1	NM_053409			5'cagctaggcagacaccatca3'	5'atgggcaccttcgtgtagtc3'			
Mif	NM_031051	BG380752	Invitrogen					
Mmp9	NM_031055					5'aacttcgacgctgacaagaa3'	5'tttagagccacgaccatacaga3'	Dr. Ruth Muschel (University of Pennsylvania, Philadelphia, PA) Peters et al 1999
Msn	NM_030863			5'cccaaagagtcttggagcag3'	5'agctcttccttctcccgttc3'			
Mt1a	NM_138826			5'caccagatctcggaatggac3'	5'tcggtagaaaacggggttta3'			
Nfe2l2	NM_031789	AI177161	Invitrogen					
Pgam1	NM_053290	BI278242	Invitrogen					
Phlda2	NM_001100521			5'gcgacagcctgttccaggtatg3'	5'tgggttggaagcaggtaaccta3'			
Pik3cb	NM_053481					5'aaatttggcaggttccgccagt3'	5'tcaacatcagcgcaaacagggt3'	
Plac1	NM_001024894			5'accctgctgcaagaatgaaa3'	5'cgcccatgttactgctaggt3'			
Prl4a1	NM_017036					5'gaccaccagatgccacactt3'	5'caggagctttatgtttgattcctt3'	ML Duckworth, Unv Manitoba, Duckworth et al J Bio Chem 261: 10879-10889, 1986
Prl3b1	NM_012535			5'gcaccaaattaccgaatgtccact3'	5'tcagcagttgttgttataaatatcgcg3'	5'accatgcttctctgggacact3'	5'aggcttccagtggacattcggtaa3'	
Rhox9	NM_001024874			5'gctgccaaagtttccaaaag3'	5'gatcttctcatccggaacca3'			
Rhob	NM_022542					5'aaacctccctccctctccc3'	5'tagaagtaccactgggctgggaa3'	
Rsp1	NM_138537			5'ctgggcctctcatttgtgat3'	5'agacaactctgaaggattgatga3'	5'gaggctaaagaggaccaggaa3'	5'tgcagcagaattgagcaagaa3'	
S1pr1	NM_017301			5'gaaactacacaacggcagca3'	5'atgatggggttggtacctga3'			
Satb1	NM_001012129					5'tgagagggaaaggagcttga3'	5'tgttctctggcttcccattc3'	
Seam6d	NM_001107768					5'ggccagtgatgctgtcattt3'	5'tatgttccacggcgatttct3'	
Serpine1	NM_012620					5'agtctttccgaccaagagca3'	5'gtgccgaaccacaaagagaa3'	
Slc16a3	NM_030834	BE112961	Invitrogen					
Slc28a2	NM_031664	AI059393	Invitrogen					
Star	NM_031558			5'tctcaactggaagcaacactcta3'	5'taccagtcagtcctagtgtct3'			
Dif EST #1	AI012949	AI012949	ATCC					
Dif EST #2	AA964255	AA964255	Invitrogen					
Tfpi	NM_017200	BF551171	Invitrogen					
Gapdh	NM_017008			5'accacagtccatgccatcac3'	5'tccaccaccctgttgctgta3'			
