# Supplementary material for: Phosphatidylinositol 3 kinase modulation of trophoblast cell differentiation
Source: BMC Dev Biol. 2010 Sep 14;10:97. doi: 10.1186/1471-213X-10-97 (PMC2944162; doi:10.1186/1471-213X-10-97)
Supplement: Additional file 2 — Table S2: Trophoblast stem-associated genes. [file 1471-213X-10-97-S2.PDF]

Table S2. Trophoblast stem-associated genes

Affymetrix ID	Gene Title	Gene Symbol	Stem Ave	Dif Ave	GenBank	romosomal Locat	Stem/ Dif	t-test
1374863_at	similar to retinoid binding protein 7 (predicted)	RGD1562168_predicted	3217	33	NM_001108693 /// XM_001075584 /// XM_575960	5q36	97.63	0.001
1367660_at	fatty acid binding protein 3	Fabp3	23051	323	NM_024162	5	71.41	0.001
1378106_at	pleckstrin homology-like domain, family A, member 2 (predicted)	Phlda2_predicted	7644	157	XM_001065824 /// XM_215132	1q41	48.67	0.000
1374273_at	leucine zipper-EF-hand containing transmembrane protein 1	Cxadr	980	24	BG665433	---	41.22	0.000
1370884_at	sepiapterin reductase	Spr	3630	115	NM_019181 /// XM_001069367 /// XM_342714	4q34	31.45	0.000
1391509_at	tumor-associated calcium signal transducer 2	Tacstd2	2215	93	NM_001009540	4q31	23.92	0.007
1367919_at	nuclear pore membrane glycoprotein 210	Pom210	1013	45	NM_053322	4q34	22.72	0.000
1368870_at	inhibitor of DNA binding 2	Id2	1958	93	NM_013060	6q16	21.03	0.004
1369063_at	acidic (leucine-rich) nuclear phosphoprotein 32 family, member A	Anp32a	3956	197	NM_012903 /// XM_001075603	8q24	20.05	0.008
1389378_at	CDC42 effector protein (Rho GTPase binding) 5 (predicted)	Cdc42ep5_predicted	2771	145	NM_001108469 /// XM_001070494 /// XM_341784	1q12	19.08	0.000
1389253_at	vanin 1	Vnn1	1163	61	NM_001025623	1p12	18.94	0.000
1372326_at	Solute carrier family 2 (facilitated glucose transporter), member 3	Slc2a3	4491	258	NM_017102	4q42	17.44	0.000
1372629_at	similar to KIAA0925 protein	LOC300768	819	48	XM_001074062 /// XM_217183	8q24	16.95	0.000
1371840_at	endothelial differentiation sphingolipid G-protein-coupled receptor 1	Edg1	4220	263	NM_017301	2q41	16.08	0.000
1390566_a_at	creatine kinase, mitochondrial 1, ubiquitous	Ckmt1	1538	108	NM_001012738	3q35	14.21	0.000
1378262_at	Transcribed locus	---	2266	160	BG377362	---	14.13	0.000
1387135_at	a disintegrin and metallopeptidase domain 15 (metargidin)	Adam15	1370	99	NM_020308	2q34	13.87	0.000
1390839_at	PQ loop repeat containing 3	Pqlc3	11449	843	NM_001034952	6q15	13.58	0.001
1367654_at	fat tumor suppressor homolog (Drosophila)	Fath	1695	130	NM_031819	16q11	13.06	0.000
1389609_at	transmembrane 7 superfamily member 3	Tm7sf3	1347	104	NM_001011970	4q44	13.00	0.002
1387707_at	solute carrier family 2 (facilitated glucose transporter), member 3	Slc2a3	1986	156	NM_017102	4q42	12.72	0.000
1375532_at	inhibitor of DNA binding 2	Id2	14665	1162	AI008792	6	12.62	0.030
1371986_at	adaptor-related protein complex AP-4, sigma 1 (predicted)	Anp32a	2066	167	AI576652	8	12.37	0.000
1373530_at	cyclin E	Ccne1	2506	218	NM_001100821 /// XM_001077331 /// XM_574426	1q22	11.52	0.000
1383496_at	similar to hypothetical protein FLJ20171 (predicted)	RGD1560481_predicted	5046	451	XM_001064461 /// XM_575771	5q13	11.19	0.000
1388781_at	vitamin D receptor interacting protein	Ssbp3	2807	252	AW434329	---	11.13	0.000
1368165_at	phosphoribosyl pyrophosphate synthetase 1	Prps1	1978	181	NM_017243	Xq35	10.94	0.000
1383935_at	Transcribed locus	---	859	79	AW252428	---	10.89	0.000
1371237_a_at	metallothionein 1a	Mt1a	7363	685	NM_138826	19p12	10.75	0.000
1383290_at	serine peptidase inhibitor, Kunitz type 1	Spint1	1350	129	NM_001004265	3q35	10.45	0.002
1386120_at	hypothetical protein LOC689147	LOC689147	2139	205	XM_001069739	5q13	10.43	0.000
1387232_at	bone morphogenetic protein 4	Bmp4	1035	100	NM_012827	15p14	10.36	0.004

Table S2. Trophoblast stem-associated genes

Affymetrix ID	Gene Title	Gene Symbol	Stem Ave	Dif Ave	GenBank	romosomal Locat	Stem/ Dif	t-test
1367735_at	acetyl-Coenzyme A dehydrogenase, long-chain	Acadl	3366	327	NM_012819	9q32	10.29	0.000
1368812_at	lamin B1	Lmnb1	936	91	NM_053905	18q12.1	10.27	0.001
1383747_at	ect2 oncogene (predicted)	Ect2_predicted	5018	499	NM_001108547 /// XM_001057016 /// XM_342220	2q24	10.06	0.000
1391063_at	kinesin family member 23 (predicted)	Kif23_predicted	2262	228	NM_001108155 /// XM_001073723 /// XM_236313	8q24	9.93	0.000
1389085_at	Transcribed locus	---	1576	162	BI296359	---	9.75	0.000
1368260_at	aurora kinase B	Aurkb	2482	257	NM_053749	10q24	9.67	0.000
1382830_at	suppressor of variegation 3-9 homolog 2 (Drosophila) (predicted)	Suv39h2_predicted	2720	284	NM_001108883 /// XM_001066854 /// XM_344633	17q12.3	9.56	0.000
1377940_at	hypothetical LOC287534	RGD1359691	1303	138	NM_001007611 /// XM_001080740	10q24	9.43	0.000
1368689_at	gap junction membrane channel protein beta 5	Gjb5	1488	159	NM_019241	5q36	9.35	0.001
1383926_at	budding uninhibited by benzimidazoles 1 homolog, beta (S. cerevisiae)	Bub1b	2547	275	XM_001080736 /// XM_342494	3q35	9.26	0.000
1387812_at	proprotein convertase subtilisin/kexin type 6	Pcsk6	2968	327	NM_012999	1q22	9.08	0.000
1370432_at	POU domain, class 3, transcription factor 1	Pou3f1	2034	228	NM_138838	5q36	8.93	0.000
1368363_at	Kruppel-like factor 5	Klf5	5887	665	NM_053394	15q21	8.85	0.000
1368517_at	single stranded DNA binding protein 3	Ssbp3	1536	175	NM_053358	5q34	8.79	0.000
1387028_a_at	inhibitor of DNA binding 1	Id1	8485	982	NM_012797	3q41	8.64	0.000
1377725_at	Transcribed locus	---	861	100	AI575322	8	8.58	0.000
1393904_at	similar to lin-28 homolog (predicted)	RGD1566408_predicted	2594	304	NM_001109269 /// XM_001066291 /// XM_575928	5q36	8.53	0.005
1368437_at	carbonic anhydrase 4	Ca4	3337	396	NM_019174	10q26	8.42	0.000
1395652_at	glycosyltransferase-like 1B	Gyltl1b	1437	172	NM_199107	3q24	8.37	0.000
1393816_at	hypothetical protein LOC689399	LOC689399	2099	252	XM_001058771 /// XM_001070657	1p11	8.32	0.001
1368180_s_at	glutathione-S-transferase, alpha type2	Gsta2	3070	374	NM_017013	8q31	8.22	0.000
1389440_at	Transcribed locus	---	3105	383	AI178476	---	8.10	0.000
1380611_at	FK506 binding protein 5	Fkbp5	5674	701	NM_001012174	20p12	8.09	0.000
1379448_at	Ttk protein kinase (predicted)	Ttk_predicted	2359	296	NM_001108172 /// XM_001062174 /// XM_236477	8q31	7.97	0.000
1377743_at		Spred1	1578	203	BI292687	---	7.78	0.011
1375937_a_at	similar to RIKEN cDNA G630055P03 gene	RGD1309383	1095	142	NM_001024257	19q12	7.71	0.001
1390454_at	4-nitrophenylphosphatase domain and non-neuronal SNAP25-like protein homolog 1 (C. elegans)	Nipsnap1	1186	157	XM_001067526 /// XM_341249	14q21	7.57	0.000
1376084_a_at	extra spindle poles like 1 (S. cerevisiae) (predicted)	Espl1_predicted	2037	271	XM_001067790 /// XM_235691	7q36	7.52	0.001
1374540_at	cell division cycle associated 7	Cdca7	2333	313	NM_001025693	3q22	7.46	0.000
1389054_at	similar to RIKEN cDNA 0610040J01	LOC498368	860	116	NM_001017500	14p11	7.41	0.000
1375277_at	similar to Notch-regulated ankyrin repeat protein	LOC499745 /// LOC688240	1058	146	XM_575080 /// XR_009475	3p13	7.23	0.000
1394039_at	Kruppel-like factor 5	Klf5	8621	1233	NM_053394	15q21	6.99	0.000

Table S2. Trophoblast stem-associated genes

Affymetrix ID	Gene Title	Gene Symbol	Stem Ave	Dif Ave	GenBank	romosomal Locat	Stem/ Di	t-test
1370894_at	claudin 7	Cldn7	1889	273	NM_031702	10q24	6.92	0.002
1385668_at	---	---	827	120	AI044864	---	6.90	0.000
1391317_at	similar to RIKEN cDNA 2810433K01 (predicted)	RGD1310784_predicted	2011	292	NM_001106134 /// XM_001053750 /// XM_214527	18q12.2	6.89	0.001
1378006_at	Transcribed locus, moderately similar to XP_893119.1 PREDICTED: similar to melanoma antigen [Mus musculus]	---	1239	181	AI233832	---	6.83	0.003
1368811_at	lamin B1	Lmnb1	2374	351	NM_053905	18q12.1	6.77	0.000
1379380_at	sprouty homolog 1 (Drosophila) (predicted)	Spry1_predicted	1279	190	NM_001106427 /// XM_001069163 /// XM_227050	2q25	6.72	0.000
1391051_at	gene rich cluster, C2f gene (predicted)	Gjb3	9250	1378	AA997873	5	6.71	0.000
1371976_at	hypothetical protein LOC682248 /// hypothetical protein LOC685545	LOC682248 /// LOC685545	1491	223	NM_001109475 /// XM_001060664 /// XM_001064248	10q12	6.68	0.000
1387747_at	gap junction membrane channel protein beta 3	Gjb3	5356	810	NM_019240	5q36	6.61	0.000
1368965_at	solute carrier family 16 (monocarboxylic acid transporters), member 3	Slc16a3	3437	523	NM_030834	10q32.3	6.57	0.000
1382138_at	similar to Notch-regulated ankyrin repeat protein	LOC499745 /// LOC688240	918	141	XM_575080 /// XR_009475	3p13	6.51	0.000
1379386_at	similar to B99 protein (predicted)	RGD1563164_predicted	1960	301	XM_001078275 /// XM_217016	7q34	6.51	0.000
1385733_at	exonuclease 1 (predicted)	Exo1_predicted	1839	284	NM_001107198 /// XM_001059185 /// XM_222932	13q25	6.47	0.000
1369935_at	cyclin D3	Ccnd3	13904	2172	NM_012766	8q24	6.40	0.000
1397341_at	PDZ binding kinase (predicted)	Pbk_predicted	1083	171	NM_001079937 /// XM_001066776 /// XM_224300	15p12	6.32	0.000
1385781_at	similar to SNF2/RAD54 family protein (predicted)	RGD1565734_predicted	911	144	XM_001070442 /// XM_228546	Xq31	6.32	0.000
1393581_at	asp (abnormal spindle)-like, microcephaly associated (Drosophila) (predicted)	Aspm_predicted	1827	290	NM_001105955 /// XM_001067212 /// XM_213891	13q13	6.30	0.000
1389668_at	spindle pole body component 25 homolog (S. cerevisiae)	Spbc25	5121	817	NM_001009654	3q21	6.27	0.000
1391626_at	similar to RIKEN cDNA 2610034E18 gene (predicted)	RGD1309007_predicted	1235	198	XM_001072054 /// XM_215595	2q34	6.24	0.000
1373026_at	spindle pole body component 24 homolog (S. cerevisiae) (predicted)	Spbc24_predicted	2274	366	XM_001077474 /// XM_343359	8q13	6.22	0.000
1378629_at	special AT-rich sequence binding protein 1	Satb1	9335	1514	NM_001012129	9q11	6.17	0.000
1389735_at	ribosomal protein S6 kinase polypeptide 6 (predicted)	Rps6ka6_predicted	1004	163	XM_001061436 /// XM_228473	Xq31	6.14	0.000
1369629_at	adenosine kinase	Adk	1184	193	NM_012895	15p16	6.13	0.000
1385522_at	origin recognition complex, subunit 1-like (S.cereviaiae)	Orc1l	1646	270	NM_177931	5q35	6.10	0.000
1368799_at	baculoviral IAP repeat-containing 5	Birc5	2192	360	NM_022274	---	6.10	0.000
1371455_at	phosphomannomutase 1	Pmm1	3113	517	NM_001008323	7q34	6.02	0.001
1383696_at	similar to protein tyrosine phosphatase-like (proline instead of catalytic arginine), member a isoform 1	LOC680115 /// LOC683916	858	143	NM_001101001 /// XM_001056393 /// XM_001056514 /// XM_001067746 /// XM_001067857	17q12.3	6.01	0.000

Table S2. Trophoblast stem-associated genes

Affymetrix ID	Gene Title	Gene Symbol	Stem Ave	Dif Ave	GenBank	romosomal Locat	Stem/ Di	t-test
1379057_at	hypothetical protein LOC683460	LOC683460	801	134	XM_001065347 /// XM_001065403	---	5.98	0.000
1374449_at	cell division cycle associated 3 /// similar to cell division cycle associated 3 (predicted)	Cdca3 /// RGD1562067_predicted	2984	502	NM_001007648 /// XR_007938	4q42 /// 9q22	5.95	0.000
1389800_at	PWWP domain containing 2 (predicted)	Pwwp2_predicted	1820	307	NM_001108507 /// XM_001058109 /// XM_341955	1q41	5.93	0.000
1387895_s_at	cell division cycle 20 homolog (S. cerevisiae)	Cdc20	1853	315	NM_171993	5q36	5.89	0.000
1373754_at	Transcribed locus	---	3011	524	AI599232	---	5.75	0.000
1392065_at	TAR DNA binding protein (predicted)	RGD1561740_predicted	929	162	BE116803	---	5.72	0.000
1373897_at	Lamin B1	Lmnb1	10711	1877	NM_053905	18q12.1	5.71	0.000
1374388_at	EF hand domain containing 2	Efhd2	9193	1615	NM_001031648	5q36	5.69	0.000
1390838_at	---	---	2424	427	BE115823	---	5.68	0.000
1388650_at	topoisomerase (DNA) 2 alpha	Top2a	4847	854	NM_022183	10q31	5.68	0.005
1385961_at	Kruppel-like factor 5	Klf5	2942	520	NM_053394	15q21	5.66	0.001
1387769_a_at	inhibitor of DNA binding 3	Id3	1364	243	NM_013058	5q36	5.62	0.001
1374794_at	kinesin family member 15	Kif15	1758	316	NM_181635	8q32	5.57	0.000
1374119_at	E74-like factor 3	Elf3	1523	275	NM_001024768	13q13	5.53	0.001
1389457_at	myeloblastosis oncogene-like 2 (predicted)	Mybl2_predicted	2695	487	NM_001106536 /// XM_001070125 /// XM_215922	3q42	5.53	0.000
1371928_at	cell division cycle associated 8	Cdca8	2081	377	NM_001025050	5q36	5.52	0.000
1383578_at	similar to DNA repair protein RAD51 homolog 1 (predicted)	RGD1563603_predicted	5067	922	NM_001109204 /// XM_001075212 /// XM_575211	3q35	5.49	0.000
1384231_at	similar to Shc SH2-domain binding protein 1	LOC687121	2895	529	XM_001077162	---	5.47	0.000
1374775_at	antigen identified by monoclonal antibody Ki-67 (predicted)	Mki67_predicted	4566	841	XM_001056221 /// XM_225460	1q41	5.43	0.001
1385086_at	budding uninhibited by benzimidazoles 1 homolog (S. cerevisiae) (predicted)	Bub1_predicted	4644	858	NM_001106507 /// XM_001078727 /// XM_215849	3q36	5.41	0.000
1374912_at	kinesin family member 2C	Kif2c	1428	264	NM_001085369 /// NM_134472	5q36	5.41	0.000
1375224_at	pleckstrin homology-like domain, family A, member 3	Phlda3	1113	207	NM_001012206	13q13	5.38	0.000
1382627_at		Satb1	2659	498	AA956525	9	5.34	0.005
1367609_at	macrophage migration inhibitory factor	Mif	9661	1816	NM_031051	20p12	5.32	0.000
1373625_at	serine hydroxymethyltransferase 1 (soluble)	Shmt1	1695	319	NM_001047842 /// XM_001076217 /// XM_213324	10q22	5.31	0.000
1373722_at	kinesin family member 20A (predicted)	Kif20a_predicted	2655	504	NM_001108426 /// XM_001061764 /// XM_341592	18p12	5.27	0.000
1387264_at	potassium channel, subfamily K, member 6 /// similar to potassium channel, subfamily K, member 6	Kcnk6 /// LOC501662	1065	202	NM_053806 /// XR_006802 /// XR_007641	1q21 /// Xq37	5.27	0.000
1391518_at	---	---	921	177	AI579823	1	5.20	0.000
1389033_at	similar to RIKEN cDNA 2900010M23 (predicted)	RGD1306917_predicted	2774	536	NM_001108528 /// XM_342099	20p12	5.18	0.000
1373761_at	similar to Protein FAM60A (Tera protein)	LOC686611	3128	607	XM_001074776 /// XM_001074804	---	5.15	0.000

Table S2. Trophoblast stem-associated genes

Affymetrix ID	Gene Title	Gene Symbol	Stem Ave	Dif Ave	GenBank	romosomal Locat	Stem/ Dif	t-test
1393202_a_at	Insulin-like growth factor 2, binding protein 3	Igf2bp3	1286	250	NM_001047888 /// XM_001055450 /// XM_231739	4q24	5.15	0.001
1390050_at	similar to Golgi phosphoprotein 2 (Golgi membrane protein GP73)	LOC680692 /// LOC682869	1366	266	XM_001056825 /// XM_001056896 /// XM_001061205 /// XM_001061265	17p14	5.14	0.000
1390918_at	GH regulated TBC protein 1	Grtp1	2363	460	BE099056	16q12.5	5.14	0.000
1394419_at	similar to RIKEN cDNA 6530401L14 gene	RGD1309107	7080	1392	XM_001074602 /// XM_230459	3q34	5.09	0.000
1388882_at	FK506 binding protein 3 (predicted)	Fkbp3_predicted	4544	894	NM_001106736 /// XM_001080093 /// XM_216717	6q24	5.08	0.000
1389756_at	maternal embryonic leucine zipper kinase (predicted)	Melk_predicted	2288	457	NM_001108662 /// XM_001064686 /// XM_342828	5q22	5.01	0.000
1389858_at	thymidine kinase 1	Tk1	1664	332	NM_052800 /// XM_001081763	---	5.01	0.000
1368226_at	Nucleoside 2-deoxyribosyltransferase domain containing protein RGD620382	RGD620382	2129	429	NM_133525	9q12	4.96	0.002
1388901_at	FK506 binding protein 5	Fkbp5	2531	510	NM_001012174	20p12	4.96	0.000
1393947_at	Solute carrier family 25 (mitochondrial carrier; ornithine transporter) member 15	Slc25a15	1941	393	NM_001047880 /// XM_001064456 /// XM_001064514 /// XM_001064627 /// XM_001064682 /// XM_224969	16q12.5	4.93	0.000
1388547_at	claudin 4	Cldn4	2905	591	NM_001012022	12q12	4.92	0.000
1388853_at	mitochondrial ribosomal protein L54 (predicted)	Mrpl54_predicted	2833	581	NM_001106770 /// XM_001075791 /// XM_216854	7q11	4.88	0.000
1373283_at	Transcribed locus, strongly similar to NP_035735.1 thymopoietin [Mus musculus]	---	1637	340	BE113158	7	4.82	0.000
1367721_at	syndecan 4	Sdc4	4809	1004	NM_012649	3q42	4.79	0.000
1378674_at	similar to engrailed protein (predicted)	RGD1561842_predicted	1125	235	NM_001109214 /// XM_001058797 /// XM_575315	4q11	4.78	0.001
1369380_at	kinesin family member 2C	Kif2c	945	199	NM_001085369 /// NM_134472	5q36	4.75	0.000
1369213_at	L1 cell adhesion molecule	L1cam	912	192	NM_017345 /// XM_001053015	Xq37	4.74	0.028
1373451_at	similar to hypothetical protein FLJ11712	MGC94780	1255	265	NM_001007007	15p12	4.73	0.000
1375849_at	RGM domain family, member A (predicted)	Rgma_predicted	1092	231	NM_001107524 /// XM_001062354 /// XM_218791	1q31	4.73	0.000
1367585_a_at	ATPase, Na+/K+ transporting, alpha 1 polypeptide	Atp1a1	5807	1228	NM_012504	2q34	4.73	0.000
1370823_at	BMP and activin membrane-bound inhibitor, homolog (Xenopus laevis)	Bambi	1536	325	NM_139082	17q12.1	4.72	0.029
1398326_at	similar to Nur77 downstream protein 2	MGC105647	2918	622	NM_001007008 /// XM_001079820	20p12	4.69	0.000
1369630_at	adenosine kinase	Adk	2952	630	NM_012895	15p16	4.69	0.000
1383940_at	cell division cycle associated 1	Cdca1	3879	828	NM_001012028	13q24	4.68	0.000
1385222_at	eomesodermin	LOC316052	3490	748	XM_001061749 /// XM_001077231	8q32	4.66	0.000

Table S2. Trophoblast stem-associated genes

Affymetrix ID	Gene Title	Gene Symbol	Stem Ave	Dif Ave	GenBank	romosomal Locat	Stem/ Dif	t-test
1392899_at	protein regulator of cytokinesis 1 (predicted)	Prc1_predicted	2944	635	NM_001107529 /// XM_001061201 /// XM_218820	1q31	4.63	0.000
1375559_at	similar to thyroid receptor-interacting protein 6	LOC686323	2741	593	XM_001069537	---	4.62	0.000
1370308_at	RS21-C6 protein	Rs21c6	2713	588	NM_138892	1q36	4.61	0.000
1387024_at	dual specificity phosphatase 6	Dusp6	1485	323	NM_053883	7q13	4.60	0.000
1371539_at	nucleolar protein family A, member 2 (predicted)	Nola2_predicted	9562	2104	NM_001105779 /// XM_001071992 /// XM_213293	10q22	4.54	0.000
1388436_at	small nuclear ribonucleoprotein polypeptide A	Snrpa	2298	507	NM_001008303	1q21	4.54	0.000
1375205_at	p300/CBP-associated factor	Pcaf	970	214	NM_001024252	9q11	4.53	0.001
1379582_a_at	cyclin A2	Ccna2	11416	2544	NM_053702	2q25	4.49	0.001
1367987_at	arginyl aminopeptidase (aminopeptidase B)	Rnpep	7055	1576	NM_031097	13q13	4.48	0.000
1370158_at	myosin, heavy polypeptide 10, non-muscle	Myh10	4095	916	NM_031520	10q24	4.47	0.000
1372692_at	tyrosine kinase, non-receptor, 2	Tnk2	1000	225	NM_001008336 /// XM_001072701	11q22	4.45	0.000
1371862_at	ribonucleotide reductase M1	Rrm1	3974	894	NM_001013236 /// XM_001052982	14q21	4.44	0.000
1383316_at	HD domain containing 2 (predicted)	Hddc2_predicted	998	226	NM_001108460 /// XM_001057933 /// XM_341741	1p11	4.42	0.000
1378640_at	ubiquitin-like, containing PHD and RING finger domains, 1	Uhrf1	928	210	NM_001008882	9q11	4.41	0.003
1389748_at	Transcribed locus	---	832	190	BE118122	---	4.39	0.001
1392061_at	minichromosome maintenance deficient 10 (S. cerevisiae) (predicted)	Mcm10_predicted	1238	283	NM_001107366 /// XM_001071383 /// XM_225570	17q12.3	4.38	0.000
1373829_at	fibroblast growth factor receptor 2	Fgfr2	3813	877	NM_001109892 /// NM_001109893 /// NM_001109894 /// NM_001109895 /// NM_001109896 /// NM_012712 /// XM_001077699 /// XM_001077810 /// XM_001079420 /// XM_001079450 /// XM_001079458 /// XM_001079467 /// XM_001079477 /// XM_001079488 /// XM_001079498 /// XM_001079510 /// XM_001079521 /// XM_001079530 /// XM_341940	1q37	4.35	0.000
1373250_at	similar to Anillin (predicted)	RGD1566097_predicted	3502	806	XM_001067026 /// XM_225479	17q12.1	4.34	0.000
1387361_s_at	phosphoglycerate kinase 1	Pgk1	12161	2814	NM_053291	Xq31	4.32	0.000
1368400_at	translocase of inner mitochondrial membrane 8 homolog a (yeast)	Timm8a	2175	504	NM_053370	Xq34	4.31	0.000
1372186_a_at	topoisomerase (DNA) 2 alpha	Top2a	2955	685	NM_022183	10q31	4.31	0.006
1372685_at		Cdkn3_predicted	1218	283	BE113362	15	4.30	0.002

Table S2. Trophoblast stem-associated genes

Affymetrix ID	Gene Title	Gene Symbol	Stem Ave	Dif Ave	GenBank	romosomal Locat	Stem/ Di	t-test
1380775_at	M-phase phosphoprotein 1 (predicted)	Mphosph1_predicted	3193	743	NM_001107609 /// XM_001080017 /// XM_220055	1q52	4.30	0.000
1378296_at	similar to Condensin complex subunit 2 (Barren homolog protein 1) (Chromosome-associated protein H) (mCAP-H) (XCAP-H homolog)	LOC680089	2386	558	XM_001055639	3q36	4.27	0.000
1376690_at	---	---	2013	477	BI296277	4	4.22	0.001
1372250_at	SCF apoptosis response protein 1	LOC499941	2446	580	NM_001047111 /// XM_001071480 /// XM_575284	3q42	4.21	0.000
1368649_at	dyskeratosis congenita 1, dyskerin	Dkc1	1351	321	NM_133419	---	4.21	0.002
1374195_at	ladinin (predicted)	Lad1_predicted	1318	313	NM_001107942 /// XM_001063663 /// XM_233122	13q13	4.21	0.000
1370999_at	sperm associated antigen 5	Spag5	1897	454	NM_001044224 /// XM_001080820 /// XM_340848	10q25	4.18	0.001
1392981_at	Iroquois related homeobox 4 (Drosophila) (predicted)	Irx4_predicted	3397	816	NM_001107330 /// XM_001057125 /// XM_001057181 /// XM_001057247 /// XM_225068	17p14	4.16	0.000
1372997_at		Csnk2b	2011	488	AI105243	---	4.12	0.000
1368756_at	thioesterase domain containing 1	Thedc1	1222	297	NM_022705	17q12.3	4.11	0.000
1382778_at	Dual specificity phosphatase 6	Dusp6	982	240	NM_053883	7q13	4.09	0.004
1368002_at	mutS homolog 2 (E. coli)	Msh2	2306	570	NM_031058	6q12	4.05	0.000
1372516_at	kinesin family member 22	Kif22	2796	692	NM_001009645	1q36	4.04	0.000
1368470_at	gamma-glutamyl hydrolase	Ggh	1992	495	NM_012960	5q21	4.03	0.001
1382493_at	Transcribed locus, strongly similar to XP_223060.3 PREDICTED: similar to LEK1 [Rattus norvegicus]	---	2105	524	BG377427	---	4.02	0.008
1371277_at	caudal type homeo box 1	Cdx1	1231	309	XM_001062129 /// XM_344691	18q12.1	3.98	0.000
1367834_at	spermidine synthase	Srm	1512	381	NM_053464 /// XM_001075160	5q36	3.97	0.000
1393342_at	integrin alpha 9	Slc25a15	1629	418	BG380998	16	3.90	0.002
1368356_a_at	type 1 tumor necrosis factor receptor shedding aminopeptidase regulator	Arts1	2203	567	NM_030836	2q11	3.89	0.000
1392953_at	similar to protein tyrosine phosphatase-like (proline instead of catalytic arginine), member a isoform 1	LOC680115 /// LOC683916	1081	278	NM_001101001 /// XM_001056393 /// XM_001056514 /// XM_001067746 /// XM_001067857	17q12.3	3.89	0.001
1372903_at	similar to 3000004C01Rik protein	RGD1310360	1894	488	NM_001039019	10q32.1	3.88	0.000
1367780_at	pituitary tumor-transforming 1	Pttg1	3013	778	NM_022391	10q21	3.87	0.000
1377832_at	polo-like kinase 4 (Drosophila) (predicted)	Plk4_predicted	2442	630	NM_001107669 /// XM_001070843 /// XM_227064	2q26	3.87	0.001
1387206_at	UDP-Gal:betaGlcNAc beta 1,4- galactosyltransferase, polypeptide 6	B4galt6	2592	669	NM_031740	18p12	3.87	0.000
1367593_at	selenoprotein W, muscle 1	Sepw1	1002	259	NM_013027	1q21	3.87	0.000
1388488_at	LSM3 homolog, U6 small nuclear RNA associated (S. cerevisiae) (predicted)	Lsm3_predicted	5704	1474	NM_001106611 /// XM_001073506 /// XM_216220	4q34	3.87	0.002

Table S2. Trophoblast stem-associated genes

Affymetrix ID	Gene Title	Gene Symbol	Stem Ave	Dif Ave	GenBank	romosomal Locat	Stem/ Di	t-test
1382096_at	chemokine-like factor super family 5 (predicted)	Cklfsf5_predicted	2126	550	NM_001106034 /// XM_001056147 /// XM_214187	15p13	3.87	0.000
1378592_at	tripartite motif-containing 59 (predicted)	Trim59_predicted	1759	455	NM_001108945 /// XM_001062051	2q33	3.86	0.001
1385437_at	carbamyl phosphatate synthetase 2	Cad	860	223	NM_001105710 /// XM_001063872 /// XM_001063923 /// XM_001063974 /// XM_001064023 /// XM_343027	6q14	3.86	0.004
1387889_at	folate receptor 1 (adult)	Folr1	5347	1392	NM_133527	1q32	3.84	0.000
1367576_at	glutathione peroxidase 1	Gpx1	7955	2075	NM_030826	---	3.83	0.000
1392580_at	Transcribed locus	---	900	235	BF282184	---	3.83	0.001
1389471_at	translocase of outer mitochondrial membrane 34 (predicted)	Tomm34_predicted	2336	613	NM_001044244 /// XM_001069920 /// XM_001069958 /// XM_230832	3q42	3.81	0.000
1370345_at	cyclin B1	Ccnb1	4214	1105	NM_171991	2q12	3.81	0.001
1376951_at	MAD2 (mitotic arrest deficient, homolog)-like 1 (yeast) (predicted)	Mad2l1_predicted	1166	307	NM_001106594 /// XM_001061025 /// XM_216161	4q31	3.80	0.001
1392754_at	similar to cysteine-rich glycoprotein (predicted)	RGD1566394_predicted	845	222	XM_001056204 /// XM_001056266 /// XM_574584	1q41	3.80	0.002
1389809_at	transmembrane, prostate androgen induced RNA (predicted)	Tmepai_predicted	1269	335	NM_001107807 /// XM_001054581 /// XM_230899	3q42	3.79	0.000
1389419_at	Transcribed locus, weakly similar to XP_001005337.1 PREDICTED: similar to zinc finger protein 97 [Mus musculus]	---	1795	474	BI296079	---	3.78	0.002
1373482_at	Tnf receptor-associated factor 3 (predicted)	Traf3_predicted	822	218	NM_001108724 /// XM_001071666 /// XM_343113	6q32	3.78	0.000
1382459_at	---	---	1591	423	BF287235	---	3.76	0.000
1383675_at	Transcribed locus, strongly similar to NP_955518.1 hypothetical protein LOC107094 [Mus musculus]	---	1781	476	BG371620	---	3.74	0.000
1398868_at	translocase of inner mitochondrial membrane 13 homolog (yeast)	Timm13	8115	2170	NM_145781	7q11	3.74	0.000
1392818_at	growth arrest specific 5	Gas5	2842	760	NR_002704	13q22	3.74	0.001
1383008_at	SMC4 structural maintenance of chromosomes 4-like 1 (yeast)	Smc4l1	1088	291	XM_001066172 /// XM_001069997	2q31	3.74	0.001
1382604_at	similar to DNA-directed RNA polymerase III 32 kDa polypeptide (RNA polymerase III C32 subunit)	LOC685465	1351	362	NM_001109468 /// XM_001061237 /// XM_001063897	2q11	3.73	0.000
1379530_at	vascular endothelial zinc finger 1 (predicted)	Itga9	4644	1244	AA964658	---	3.73	0.000
1387196_at	KH domain containing, RNA binding, signal transduction associated 3	Khdrbs3	1223	329	NM_022249	7q34	3.72	0.004
1373884_at	kelch-like 2, Mayven (Drosophila) (predicted)	Klhl2_predicted	1320	355	XM_001073589 /// XM_214331	16p13	3.71	0.034
1390891_at	kinesin family member 11	Kif11	1592	431	XM_001060913 /// XM_001080591	1q53	3.70	0.000

Table S2. Trophoblast stem-associated genes

Affymetrix ID	Gene Title	Gene Symbol	Stem Ave	Dif Ave	GenBank	romosomal Locat	Stem/ Dif	t-test
1375060_at	Transcribed locus, strongly similar to XP_345605.2 PREDICTED: similar to RIKEN cDNA 2610040C18 [Rattus norvegicus]	---	844	230	BE112912	5	3.66	0.004
1370376_a_at	cold shock domain protein A	Csda	2931	804	NM_031979 /// XM_001069862	4q42	3.64	0.000
1384042_at	Transcribed locus	---	2039	561	BE108303	---	3.64	0.000
1376939_at	Transcribed locus	---	1236	340	BI284907	---	3.63	0.001
1368115_at	claudin 3	Cldn3	2665	735	NM_031700	12	3.63	0.000
1371873_at	Acidic (leucine-rich) nuclear phosphoprotein 32 family, member E	Anp32e	1966	543	NM_001013200	2q34	3.62	0.000
1387036_at	hairy and enhancer of split 1 (Drosophila)	Hes1	1950	538	NM_024360	11q22	3.62	0.000
1371392_at	glucose phosphate isomerase	Gpi	6328	1749	NM_207592	1q21	3.62	0.000
1388484_at	ubiquitin-conjugating enzyme E2C (predicted)	Ube2c_predicted	1652	457	NM_001106542 /// XM_001070446 /// XM_215924	3q42	3.61	0.001
1389969_at	translocase of outer mitochondrial membrane 40 homolog (yeast)	Tomm40	2972	825	NM_212520	1q21	3.60	0.001
1389566_at	cyclin B2	Ccnb2	2794	776	NM_001009470	8q24	3.60	0.000
1388932_at	laminin, alpha 5	Lama5	3854	1071	XM_001060527 /// XM_215963	3q43	3.60	0.005
1390137_at	TRAF4 associated factor 1	Traf4af1	817	227	NM_001004264 /// XM_001080771	3q35	3.60	0.000
1372297_at	glutathione S-transferase, alpha 4	Gsta4	2262	630	NM_001106840 /// XM_001059683 /// XM_217195	8q31	3.59	0.023
1393848_at	ribonucleotide reductase M2	Rrm2	8398	2344	NM_001025740	6q16	3.58	0.002
1374800_at	karyopherin (importin) beta 1	Slc25a15	2079	581	BI303627	16	3.58	0.002
1390415_at	retinoic acid induced 3	Trip13	2689	754	BM392319	1	3.57	0.000
1376185_at	kinesin family member C1	Kifc1	2575	724	NM_001005878	20p12	3.56	0.000
1374840_at	similar to peptidyl prolyl isomerase H (predicted)	RGD1564921_predicted	2624	741	XM_001073803 /// XM_345576	5q36	3.54	0.000
1368181_at	methylenetetrahydrofolate dehydrogenase (NADP+ dependent), methenyltetrahydrofolate cyclohydrolase, formyltetrahydrofolate synthase	Mthfd1	7208	2043	NM_022508	6q24	3.53	0.000
1370294_a_at	cell division cycle 20 homolog (S. cerevisiae)	Cdc20	1480	420	NM_171993	5q36	3.53	0.000
1384230_at	similar to keratinocytes associated protein 3	LOC683980	819	233	NM_001109674 /// XM_001068323	18	3.52	0.001
1374805_at	similar to hypothetical protein MGC5528 (predicted)	RGD1561749_predicted	1438	409	XM_216934 /// XR_008375	7q31	3.52	0.000
1377802_at	similar to MYST histone acetyltransferase monocytic leukemia 4 (predicted)	RGD1566399_predicted	1595	454	XR_008228	15p16	3.52	0.009
1392597_at	similar to ionized calcium binding adapter molecule 2 (Iba2) (predicted)	RGD1305081_predicted	3501	997	NM_001108578 /// XM_001077954 /// XM_342410	3p11	3.51	0.000
1390602_a_at	similar to RIKEN cDNA C430004E15	LOC499749	1839	524	NM_001024309	3p13	3.51	0.001
1392514_at	brix domain containing 1 (predicted)	Bxdc1_predicted	2439	696	NM_001106391 /// XM_001063295 /// XM_215404	20q12	3.51	0.000
1383938_at	similar to nuclear receptor coactivator 7 (predicted)	RGD1566426_predicted	1360	388	XM_001058653 /// XM_574285	1p11	3.50	0.000

Table S2. Trophoblast stem-associated genes

Affymetrix ID	Gene Title	Gene Symbol	Stem Ave	Dif Ave	GenBank	romosomal Locat	Stem/ Di	t-test
1372886_at	transforming, acidic coiled-coil containing protein 3	Tacc3	2348	671	NM_001004424	14q21	3.50	0.000
1370346_at	cyclin B1	Ccnb1	5262	1510	NM_171991	2q12	3.48	0.000
1393293_at	checkpoint with forkhead and ring finger domains	Chfr	1149	331	NM_001009258	12q16	3.47	0.000
1370297_at	polo-like kinase 1 (Drosophila)	Plk1	1730	498	NM_017100	1q36	3.47	0.001
1386996_at	myosin light chain, regulatory B	Mrlcb	4629	1334	NM_017343	9q38	3.47	0.000
1388353_at	proliferation-associated 2G4	Pa2g4	4020	1162	NM_001004206	7q11	3.46	0.000
1388753_at	sulfatase 2	Sulf2	3640	1055	NM_001034927 /// XM_001072989 /// XM_230861	3q42	3.45	0.000
1388318_at	phosphoglycerate kinase 1	Pgk1	18066	5238	NM_053291	Xq31	3.45	0.002
1389446_at	FtsJ homolog 2 (E. coli) (predicted)	Snrpa1_predicted	4751	1379	AI576586	1	3.44	0.000
1386867_at	brain protein 44-like	Brp44l	6161	1793	NM_133561	1q11	3.44	0.000
1383315_at	hepatic protein EIIH	Eiih	957	280	NM_001009965	1q32	3.42	0.003
1385220_at	similar to Zwilch	LOC691493	2546	747	XM_001078534	8q24	3.41	0.000
1372620_at	acidic (leucine-rich) nuclear phosphoprotein 32 family, member E	Anp32e	3886	1140	NM_001013200	2q34	3.41	0.000
1373902_at	similar to mKIAA1238 protein (predicted)	RGD1560851_predicted	1499	440	XM_001060530 /// XM_342749	4q42	3.41	0.000
1370330_at	signal-induced proliferation-associated 1 like 1	Sipa1l1	1195	351	NM_139330	6q24	3.40	0.000
1373074_at	similar to RIKEN cDNA 2700002I20	RGD1307279	2597	766	NM_001008343	18q12.3	3.39	0.000
1384407_at	similar to RIKEN cDNA 1110025F24 (predicted)	RGD1311451_predicted	1290	381	XM_001079214 /// XM_213217	10q12	3.38	0.003
1373068_at	interferon-related developmental regulator 2 (predicted)	Ifrd2_predicted	1705	505	NM_001047871 /// XM_001073137 /// XM_217254	8q32	3.38	0.000
1377350_at	Transcribed locus	---	1440	428	AA859898	---	3.36	0.001
1387793_at	solute carrier family 9 (sodium/hydrogen exchanger), isoform 3 regulator 1	Slc9a3r1	2455	731	NM_021594	10q32.2	3.36	0.000
1381124_at	BRCA2 and CDKN1A interacting protein (predicted)	Slc25a15	1133	338	AA819672	16	3.35	0.002
1398903_at	esterase D/formylglutathione hydrolase	Esd	5348	1601	NM_001106051 /// XM_001071513 /// XM_214241	15q11	3.34	0.000
1374121_at	Transcribed locus	---	961	288	AI102735	---	3.34	0.000
1376231_at	casein kinase 2, beta subunit	RGD1562246_predicted	977	294	BF389244	---	3.32	0.000
1372431_at	ribosomal protein, mitochondrial, L12	Mrpl12	4942	1487	NM_001029900	10q32.3	3.32	0.000
1388341_at	RAN GTPase activating protein 1	Rangap1	2055	619	NM_001012199	7q34	3.32	0.000
1373001_at	coiled-coil-helix-coiled-coil-helix domain containing 4	Chchd4	1485	447	NM_001013431	4q34	3.32	0.000
1388443_at	CDK2 (cyclin-dependent kinase 2)-associated protein 1 (predicted) /// similar to CDK2 (cyclin-dependent kinase 2)-associated protein 1	Cdk2ap1_predicted /// LOC687549	3694	1114	XM_001079062 /// XM_341076	12q15	3.32	0.000
1374799_at	similar to mKIAA0159 protein (predicted)	RGD1562596_predicted	1248	377	XM_001065923 /// XM_575668	4q42	3.31	0.000
1367662_at	hydroxyacyl-Coenzyme A dehydrogenase type II	Hadh2	877	266	NM_031682	Xq21	3.30	0.001
1393041_at	SMC2 structural maintenance of chromosomes 2-like 1 (yeast) (predicted)	Smc2l1_predicted	1760	534	NM_001108666 /// XM_001053521 /// XM_342837	5q23	3.30	0.001

Table S2. Trophoblast stem-associated genes

Affymetrix ID	Gene Title	Gene Symbol	Stem Ave	Dif Ave	GenBank	romosomal Locat	Stem/ Di	t-test
1390579_at	similar to RIKEN cDNA 1810029B16 (predicted)	RGD1305222_predicted	4290	1302	XM_001073162 /// XM_214332	16p14	3.29	0.000
1370630_a_at	fibroblast growth factor receptor 2	Fgfr2	988	300	NM_001109892 /// NM_001109893 /// NM_001109894 /// NM_001109895 /// NM_001109896 /// NM_012712 /// XM_001077699 /// XM_001077810 /// XM_001079420 /// XM_001079450 /// XM_001079458 /// XM_001079467 /// XM_001079477 /// XM_001079488 /// XM_001079498 /// XM_001079510 /// XM_001079521 /// XM_001079530 /// XM_341940	1q37	3.29	0.002
1368036_at	protein tyrosine phosphatase, receptor type, F	Ptprf	993	302	NM_019249	5q36	3.29	0.016
1388320_at	serine protease inhibitor, Kunitz type 2	Spint2	4211	1280	NM_001082549 /// NM_199087	1q21	3.29	0.000
1379360_at	similar to Putative protein 15E1.2 (predicted)	RGD1309698_predicted	1354	413	NM_001108339 /// XM_001077390 /// XM_341095	12q16	3.28	0.000
1377172_at	similar to Pins (predicted)	RGD1560967_predicted	917	280	XM_575015	2q34	3.27	0.005
1371035_at	general transcription factor III A	Gtf3a	1284	392	XM_001069721	---	3.27	0.000
1376052_at	similar to hypothetical protein FLJ20512 (predicted)	RGD1564549_predicted	3570	1092	NM_001106227 /// XM_001063152 /// XM_214831	1q21	3.27	0.000
1398602_at	MAD2 (mitotic arrest deficient, homolog)-like 1 (yeast) (predicted)	Mad2l1_predicted	2481	759	NM_001106594 /// XM_001061025 /// XM_216161	4q31	3.27	0.006
1395376_at	DEAD/H (Asp-Glu-Ala-Asp/His) box polypeptide 11 (CHL1-like helicase homolog, S. cerevisiae) (predicted)	Ddx11_predicted	1292	395	XM_001070646 /// XM_237570	9q37	3.27	0.000
1371113_a_at	transferrin receptor	Tfrc	7349	2255	XM_001072774 /// XM_340999	11q22	3.26	0.000
1390481_a_at	ubiquitin-conjugating enzyme E2T (putative) (predicted)	Ube2t_predicted	2134	657	NM_001108344 /// XM_001062580 /// XM_341124	13q13	3.25	0.000
1386897_at	heterogeneous nuclear ribonucleoproteins methyltransferase-like 2 (S. cerevisiae)	Hrmt1l2	6975	2147	NM_024363	1q22	3.25	0.000
1382674_a_at	similar to KIAA1582 protein (predicted)	RGD1310027_predicted	1131	348	XR_006923 /// XR_009495	10q32.3	3.25	0.000
1372071_at	CD320 antigen	Cd320	1686	520	NM_001014201 /// XM_001067944	7q12	3.24	0.000

Table S2. Trophoblast stem-associated genes

Affymetrix ID	Gene Title	Gene Symbol	Stem Ave	Dif Ave	GenBank	romosomal Locat	Stem/ Di	t-test
1389600_at	hypothetical protein LOC363306 /// similar to Discs large homolog 5 (Placenta and prostate DLG) (Discs large protein P-dlg) /// hypothetical LOC363433 /// hypothetical LOC501092 /// hypothetical LOC501226 /// hypothetical LOC501250 /// hypothetical protein LOC680443 /// hypothetical protein LOC684587 /// hypothetical protein LOC685573 /// hypothetical protein LOC685668 /// similar to similar to RIKEN cDNA 1700001E04 /// hypothetical protein LOC685863 /// hypothetical protein LOC686842 /// region containing hypothetical protein LOC501396; similar to RIKEN cDNA 1700001E04 /// hypothetical protein LOC688586 /// similar to hypothetical protein LOC363306	LOC363306 /// LOC363320 /// LOC363433 /// LOC498374 /// LOC501091 /// LOC501092 /// LOC501226 /// LOC501250 /// LOC501399 /// LOC680443 /// LOC680475 /// LOC684587 /// LOC685563 /// LOC685573 /// LOC685668 /// LOC685792 /// LOC685796 /// LOC685863 /// LOC686842 /// LOC688479 /// LOC688568 /// LOC688586 /// LOC690474 /// LOC690490 /// LOC690590 /// LOC690619 /// LOC690700 /// LOC690739 /// LOC690748 /// LOC691487 /// LOC691569 /// LOC691633 /// LOC691672	829	257	XM_001056972 /// XM_001057024 /// XM_001057208 /// XM_001057350 /// XM_001062328 /// XM_001064312 /// XM_001064363 /// XM_001064760 /// XM_001065304 /// XM_001065537 /// XM_001067463 /// XM_001067519 /// XM_001068086 /// XM_001071142 /// XM_001074545 /// XM_001074598 /// XM_001074893 /// XM_001074985 /// XM_001075284 /// XM_001075438 /// XM_001075455 /// XM_001075495 /// XM_001075968 /// XM_001078495 /// XM_001078865 /// XM_001079096 /// XM_001079224 /// XM_217497 /// XM_343660 /// XM_343755 /// XM_573609 /// XM_576506 /// XM_576507 /// XM_576649 /// XM_576669 /// XM_576812	14p11 /// 14q11 ///	3.23	0.001
1381403_at	malic enzyme 2, NAD(+)-dependent, mitochondrial (predicted)	Me2_predicted	1167	362	NM_001107376 /// XM_001053085 /// XM_225729	18q12.2	3.22	0.002
1382802_x_at	---	---	1612	501	AW920828	---	3.22	0.001
1383301_at	hypothetical protein LOC498154	LOC498154	837	260	NM_001025033	12q11	3.22	0.000
1371484_at	hypothetical protein LOC686518 /// hypothetical protein LOC690349	LOC686518 /// LOC690349	4227	1314	NM_001109581 /// XM_001076769 /// XM_001076788 /// XM_001076808 /// XM_001077700 /// XM_001077716	1q43	3.22	0.000
1388802_at	brain expressed X-linked 1	Bex1	12559	3905	NM_001037365	Xq35	3.22	0.001
1367908_at	glycine cleavage system protein H (aminomethyl carrier)	Gcsh	2549	793	NM_133598	19q12	3.21	0.000
1381252_at	Transcribed locus, weakly similar to XP_001158686.1 PREDICTED: SA hypertension-associated homolog isoform 3 [Pan troglodytes]	---	922	287	BE098160	---	3.21	0.000
1378507_at	Transcribed locus, moderately similar to NP_007230.1 ATP synthase F0 subunit 6 [Rattus norvegicus]	---	3348	1044	BE108914	---	3.21	0.000

Table S2. Trophoblast stem-associated genes

Affymetrix ID	Gene Title	Gene Symbol	Stem Ave	Dif Ave	GenBank	romosomal Locat	Stem/ Dif	t-test
1372343_at	exosome component 8 (predicted)	Exosc8_predicted	2842	888	NM_001106432 /// XM_001055849 /// XM_215566	2q26	3.20	0.001
1383313_at	checkpoint with forkhead and ring finger domains	Chfr	2849	891	NM_001009258	12q16	3.20	0.017
1389302_at	similar to RIKEN cDNA D030070L09	RGD1310199	1062	332	NM_001017446	18p12	3.20	0.001
1373439_at		Lbr	7573	2381	AI178491	13	3.18	0.000
1371108_a_at	ATPase, Na+/K+ transporting, alpha 1 polypeptide	Atp1a1	4255	1342	NM_012504	2q34	3.17	0.000
1384280_at	nucleolar and spindle associated protein 1 (predicted)	Nusap1_predicted	1571	499	NM_001107762 /// XM_001075591 /// XM_230478	3q35	3.15	0.001
1390378_at	---	---	1900	604	BE107173	16	3.15	0.006
1393494_at	c-myc binding protein (predicted)	Pdgfa	957	305	AA866419	12	3.14	0.003
1371797_at	carbamyl phosphatate synthetase 2	Cad	3468	1104	NM_001105710 /// XM_001063872 /// XM_001063923 /// XM_001063974 /// XM_001064023 /// XM_343027	6q14	3.14	0.001
1374465_at	ubiquitously expressed transcript	Uxt	1154	367	NM_001006982	Xq12	3.14	0.004
1367901_at	glucuronidase, beta	Gusb	1484	473	NM_017015	12q13	3.14	0.000
1373935_at	polymerase (DNA directed), delta 2, regulatory subunit	Pold2	3114	993	NM_001013050	14q21	3.14	0.000
1397508_at	DEAD (Asp-Glu-Ala-Asp) box polypeptide 18	Ddx18	1802	575	NM_001006996	13q11	3.13	0.000
1374424_at	acidic (leucine-rich) nuclear phosphoprotein 32 family, member A	Skb1_predicted	2089	668	BE112720	15	3.13	0.000
1372811_at	kinectin 1 (predicted)	Ktn1_predicted	2610	835	XM_001073656 /// XM_341305	15p14	3.13	0.000
1371683_at	LSM4 homolog, U6 small nuclear RNA associated (S. cerevisiae) (predicted)	Lsm4_predicted	3638	1166	NM_001106073 /// XM_001067054 /// XM_214318	16p14	3.12	0.000
1395645_at	---	---	1141	366	BF408105	---	3.12	0.000
1376065_at	RRS1 ribosome biogenesis regulator homolog (S. cerevisiae) (predicted)	Rrs1_predicted	2372	763	NM_001079699 /// XM_001066738 /// XM_232622	5q11	3.11	0.001
1376868_at	Cobl-like 1 (predicted)	Cobll1_predicted	2972	957	NM_001107733 /// XM_001056368 /// XM_229988	3q21	3.11	0.000
1385788_at	Eph receptor B3 (predicted)	Ephb3_predicted	1036	334	NM_001105868 /// XM_001059403 /// XM_221311	11q23	3.10	0.001
1384565_at	---	---	2919	940	BM387706	---	3.10	0.001
1368669_at	uncoupling protein 2 (mitochondrial, proton carrier)	Ucp2	1103	356	NM_019354	1q32	3.10	0.001
1393684_at	TBC1D12: TBC1 domain family, member 12 (predicted)	Tbc1d12_predicted	2099	677	NM_001106371 /// XM_001080666 /// XM_220062	1q53	3.10	0.002
1389009_at	arginine/serine-rich coiled-coil 1	Rsrc1	1438	464	NM_001014172	2q31	3.10	0.001
1368387_at	3-hydroxybutyrate dehydrogenase, type 1	Bdh1	978	317	NM_053995	11q22	3.09	0.000
1398732_at	hypothetical protein LOC688273	LOC688273	1774	574	XM_001081726	---	3.09	0.005
1377833_at	similar to Rap1-interacting factor 1 (predicted)	RGD1562474_predicted	2739	888	XM_001065608 /// XM_215736	3q12	3.09	0.000
1367983_at	flap structure-specific endonuclease 1	Fen1	2248	731	NM_053430	1q43	3.08	0.001

Table S2. Trophoblast stem-associated genes

Affymetrix ID	Gene Title	Gene Symbol	Stem Ave	Dif Ave	GenBank	romosomal Locat	Stem/ Di	t-test
1376483_at	LSM5 homolog, U6 small nuclear RNA associated (S. cerevisiae) (predicted)	Lsm5_predicted	4520	1472	NM_001107289 /// XM_001059585 /// XM_224630	4q24	3.07	0.000
1371839_at	similar to splicing factor, arginine/serine-rich 2	Sfrs2	4840	1581	NM_001009720	10q32.3	3.06	0.000
1387268_at	RNA polymerase 1-2	Rpo1-2	1838	605	NM_031773	3q36	3.04	0.001
1393203_at	similar to RIKEN cDNA 2010309E21 (predicted)	RGD1304825_predicted	4595	1514	NM_001106605 /// XM_001074564 /// XM_216186	4q34	3.04	0.000
1391555_at	nuclear receptor coactivator 3	Ncoa3	2094	691	XM_001072953 /// XM_215947	3q42	3.03	0.004
1386864_at	phosphoglycerate mutase 1	Pgam1	19832	6545	NM_053290 /// XM_001053034	2q32	3.03	0.001
1380143_at	Transcribed locus	---	804	265	AI029786	---	3.03	0.026
1388154_at	E2F transcription factor 5	E2f5	1911	636	XM_001053974 /// XM_574892	2q23	3.00	0.000
1384068_at	cytoskeleton associated protein 2 (predicted)	Ckap2_predicted	3029	1009	XM_001073489 /// XM_224970	16q12.5	3.00	0.000
1379375_at	Platelet derived growth factor, alpha	Pdgfa	972	324	NM_012801	12q11	3.00	0.000
1374531_at	Transcribed locus	---	1419	474	AA926305	---	2.99	0.001
1397644_at	Methylthioadenosine phosphorylase (predicted)	Mtap_predicted	2360	789	NM_001047867 /// XM_001055425 /// XM_001058325	5q32	2.99	0.000
1398401_at	tudor domain containing 3	Tdrd3	2357	789	NM_001012043	15q12	2.99	0.000
1392534_at	transmembrane, prostate androgen induced RNA (predicted)	Tmepai_predicted	1092	366	NM_001107807 /// XM_001054581 /// XM_230899	3q42	2.98	0.000
1371480_at	RGD1561797 (predicted)	RGD1561797_predicted	5993	2014	XM_579997	2q34	2.98	0.001
1371982_at	AIP1	Aip1	7702	2589	NM_173117	6q13	2.98	0.001
1381298_at	minichromosome maintenance deficient 10 (S. cerevisiae) (predicted)	Mcm10_predicted	1155	392	NM_001107366 /// XM_001071383 /// XM_225570	17q12.3	2.95	0.008
1373054_at	CDW92 antigen	Cdw92	961	326	NM_001033852 /// NM_053492	5q24	2.95	0.003
1377616_at	similar to RIKEN cDNA 6720467C03 (predicted)	RGD1310681_predicted	2120	720	XM_001053730 /// XM_216357	5q13	2.95	0.000
1371879_at	leucine rich repeat containing 42	Lrrc42	1528	519	NM_001025653	5q34	2.94	0.000
1370314_at	solute carrier family 20 (phosphate transporter), member 1	Slc20a1	8015	2725	NM_031148	3q36	2.94	0.000
1370910_at	replication factor C (activator 1) 2	Rfc2	2252	767	NM_053786	12q12	2.94	0.001
1387062_a_at	checkpoint kinase 1 homolog (S. pombe)	Chek1	2151	734	NM_080400	8q21	2.93	0.000
1372156_at	transmembrane protein 97	Tmem97	3766	1285	NM_001008334	10q25	2.93	0.002
1392589_at	replication factor C (activator 1) 5 (predicted)	Rfc5_predicted	2251	772	NM_001107146 /// XM_001080107 /// XM_222214	12q16	2.92	0.000
1393982_at	polymerase (DNA directed), epsilon 2 (p59 subunit) (predicted)	Pole2_predicted	1193	409	XM_001076651 /// XM_216727	6q24	2.92	0.000
1382216_at	gem (nuclear organelle) associated protein 6	Gemin6	1978	678	NM_001009466	6q11	2.92	0.000
1371449_at	protein (peptidyl-prolyl cis/trans isomerase) NIMA-interacting 1 (predicted)	Pin1_predicted	5297	1824	NM_001106701 /// XM_001077069 /// XM_216609	8q13	2.90	0.000
1387878_at	glutamate dehydrogenase 1	Glud1	4675	1614	NM_012570	16p16	2.90	0.000
1374577_at	transforming growth factor beta regulated gene 4	Tbrg4	3111	1076	NM_001012154	14q21	2.89	0.000

Table S2. Trophoblast stem-associated genes

Affymetrix ID	Gene Title	Gene Symbol	Stem Ave	Dif Ave	GenBank	romosomal Locat	Stem/ Dif	t-test
1387726_at	caudal type homeo box 2	Cdx2	2687	930	NM_023963	12p11	2.89	0.000
1374777_at	hypothetical protein LOC502894	LOC502894	1326	459	NM_001025064	4q42	2.89	0.000
1375259_at	eukaryotic translation initiation factor 4E binding protein 2	Eif4ebp2	2903	1006	NM_001033069	20q11	2.88	0.012
1390831_at	similar to KIAA1582 protein (predicted)	RGD1310027_predicted	1422	493	XR_006923 /// XR_009495	10q32.3	2.88	0.000
1371615_at	diacylglycerol O-acyltransferase homolog 2 (mouse)	Dgat2	1647	571	NM_001012345	1q32	2.88	0.000
1381933_at	replication factor C (activator 1) 5 (predicted)	Rfc5_predicted	3286	1140	NM_001107146 /// XM_001080107 /// XM_222214	12q16	2.88	0.027
1380668_at	Hypothetical LOC316976	MGC72974	899	312	NM_198772	4q44	2.88	0.000
1377797_at	EMG1 nucleolar protein homolog (S. cerevisiae) (predicted)	Emg1_predicted	1384	481	NM_001107888 /// XM_001062108 /// XM_232345	4q42	2.88	0.001
1393119_at	Transcribed locus	---	6344	2204	BM388725	---	2.88	0.001
1368632_at	forkhead box G1	Foxg1	1226	426	NM_012560	6q22	2.88	0.003
1377872_at	similar to chromosome 7 open reading frame 30 (predicted)	RGD1306936_predicted	4545	1586	NM_001106593 /// XM_001055149 /// XM_216139	4q24	2.87	0.000
1367683_at	karyopherin (importin) alpha 2	Kpna2	9211	3217	NM_053483	10q32.1	2.86	0.002
1372556_at	hypothetical protein LOC502374	LOC502374	2346	820	XR_007265 /// XR_009623	1q41	2.86	0.004
1375686_at	peptidylprolyl isomerase (cyclophilin)-like 3	Ppil3	3221	1126	NM_175707	9q31	2.86	0.003
1376240_at	similar to MYST histone acetyltransferase monocytic leukemia 4 (predicted)	RGD1566399_predicted	910	319	XR_008228	15p16	2.86	0.001
1389425_at	similar to 5(3)-deoxyribonucleotidase, cytosolic type (Cytosolic 5,3-pyrimidine nucleotidase) (Deoxy-5-nucleotidase 1) (dNT-1)	LOC688261	923	323	XM_001081691	10	2.86	0.009
1393987_s_at	G protein-coupled receptor kinase 6	Gprk6	5740	2010	NM_001112712 /// NM_001112713 /// NM_031657	17p14	2.85	0.003
1379835_at	coxsackie virus and adenovirus receptor	Soat1	972	340	BF558512	13	2.85	0.004
1370323_at	thimet oligopeptidase 1	Thop1	1767	619	NM_172075	7q11	2.85	0.001
1387865_at	deoxyuridine triphosphatase	Dut	7201	2524	NM_001040271 /// NM_053592	3q36	2.85	0.000
1373004_at	DnaJ (Hsp40) homolog, subfamily C, member 2	Dnajc2	1910	670	NM_053776	4q11	2.85	0.009
1374953_at	similar to CG12279-PA	LOC500420	1305	458	XM_001058924 /// XM_575783	5q21	2.85	0.000
1398448_at	FUS interacting protein (serine-arginine rich) 1	Tscot_predicted	806	283	BE108346	---	2.85	0.017
1368674_at	liver glycogen phosphorylase	Pygl	3267	1147	NM_022268	6q24	2.85	0.000
1396205_at	Transcribed locus	---	840	295	BI303419	---	2.85	0.036
1384465_at	paraspeckle protein 1	Pspc1	4450	1565	NM_001025672	15p12	2.84	0.000
1378117_at	Transcribed locus	---	2221	782	BF396269	---	2.84	0.001
1370127_at	polymerase (DNA directed), delta 1, catalytic subunit	Pold1	1143	405	NM_021662	1q22	2.82	0.004
1389385_at	endothelial precursor protein B9 (predicted)	Eppb9_predicted	1683	596	NM_001105786 /// XM_001076733 /// XM_213314	10q23	2.82	0.002

Table S2. Trophoblast stem-associated genes

Affymetrix ID	Gene Title	Gene Symbol	Stem Ave	Dif Ave	GenBank	romosomal Locat	Stem/ Di	t-test
1393627_at	similar to ovary-specific acidic protein	LOC689931	1198	425	NM_001109556 /// XM_001056112 /// XM_001072574	2q26	2.82	0.001
1389537_at	Treacher Collins Franceschetti syndrome 1, homolog (predicted)	Tcof1_predicted	1510	536	NM_001106143 /// XM_001061846 /// XM_214552	18q12.1	2.82	0.001
1386669_at	similar to Protein KIAA0690	LOC679127	812	288	XM_001054805	---	2.82	0.009
1372611_at	similar to RIKEN cDNA 2010305A19 (predicted)	RGD1305274_predicted	853	303	NM_001106674 /// XM_001066987 /// XM_216484	5q35	2.81	0.004
1392670_at	similar to BH3-only member B protein (predicted)	RGD1559427_predicted	850	302	NM_001109111 /// XM_001059192 /// XM_573911	16q11	2.81	0.001
1390823_at	similar to S-phase kinase-associated protein 2 (F-box protein Skp2) (F-box/WD-40 protein 1) (predicted)	RGD1562456_predicted	1889	674	NM_001106416 /// XM_001057222 /// XM_226817	2q16	2.80	0.000
1378753_at	occludin	Ocln	1282	458	NM_031329	2q12	2.80	0.000
1392385_at	nuclear receptor coactivator 3	Ncoa3	2611	934	XM_001072953 /// XM_215947	3q42	2.80	0.000
1374857_at	similar to nucleolar protein family A, member 1	LOC499709	4078	1458	NM_001024306	2q43	2.80	0.000
1368436_at	nuclear distribution gene C homolog (Aspergillus)	Nudc	7465	2676	NM_017271	5q36	2.79	0.000
1376828_at	G protein-coupled receptor, family C, group 5, member A	Gprc5a	4364	1565	NM_001079890 /// XM_001071868 /// XM_232477	4q43	2.79	0.000
1377760_at	nucleolar complex associated 4 homolog (S. cerevisiae)	Noc4l	2101	753	NM_001014129	12q16	2.79	0.001
1388321_at	IMP3, U3 small nucleolar ribonucleoprotein, homolog (yeast) (predicted)	Imp3_predicted	2528	907	NM_001108152 /// XM_001074037 /// XM_236276	8q24	2.79	0.000
1372108_at	similar to RIKEN cDNA 2810422B04	LOC500199	3933	1411	XM_001063161 /// XM_001065864	4q33	2.79	0.000
1377708_at	hypothetical protein LOC499339	LOC499339	2771	997	NM_001025036	1q51	2.78	0.000
1367817_at	hepatoma-derived growth factor	Hdgf	4880	1761	NM_053707	2q34	2.77	0.017
1374748_at	serine hydroxymethyltransferase 1 (soluble)	Shmt1	1054	381	NM_001047842 /// XM_001076217 /// XM_213324	10q22	2.77	0.000
1367926_at	prohibitin	Phb	913	332	NM_031851 /// XM_001053030 /// XM_001053031	Xq14	2.75	0.000
1386870_at	glutamate-ammonia ligase (glutamine synthase)	Glul	961	350	NM_017073 /// XM_001053012	13q22	2.74	0.000
1383601_at	THO complex 4 (predicted)	Dufd1_predicted	1247	454	BI295614	1	2.74	0.003
1367604_at	cysteine-rich protein 2	Crip2	8761	3194	NM_022501	6q32	2.74	0.000
1371608_at	mitochondrial ribosomal protein S34 (predicted)	Mrps34_predicted	1730	631	NM_001105771 /// XM_001057977 /// XM_213234	10q12	2.74	0.003
1384224_at	---	---	890	325	BE120549	---	2.74	0.018
1373280_at	RuvB-like 2 (E. coli)	Ruvbl2	6971	2545	NM_001025405	1q22	2.74	0.000
1390218_at	similar to hypothetical protein (predicted)	RGD1310440_predicted	1433	523	XM_001057055 /// XM_342912	5q36	2.74	0.000
1372043_at	similar to ribosomal protein P0-like protein; 60S acidic ribosomal protein PO; ribosomal protein, large, P0-like (predicted)	RGD1311709_predicted	3555	1299	NM_001106697 /// XM_001071573 /// XM_216567	5q36	2.74	0.000

Table S2. Trophoblast stem-associated genes

Affymetrix ID	Gene Title	Gene Symbol	Stem Ave	Dif Ave	GenBank	romosomal Locat	Stem/ Di	t-test
1398358_a_at	integrin, beta 5	Itgb5	4150	1516	NM_147139 /// XM_001064622	11q22	2.74	0.000
1379422_at	similar to RIKEN cDNA 4833435D08	LOC300284	1462	535	NM_001013951	Xq14	2.73	0.001
1369962_at	5-aminoimidazole-4-carboxamide ribonucleotide formyltransferase/IMP cyclohydrolase	Atic	6392	2340	NM_031014	9q33	2.73	0.001
1390163_at	---	---	905	332	BF282174	---	2.73	0.000
1393104_at	Similar to hypothetical protein FLJ33977 (predicted)	RGD1306423_predicted	1533	562	NM_001108431 /// XM_001054113 /// XM_341614	18q11	2.73	0.000
1375430_at	hypothetical LOC303211	RGD1311260	954	350	NM_001013967	10q23	2.73	0.000
1377042_at	similar to polycomb group ring finger 5	LOC681178 /// LOC687730	2173	797	XM_001060464 /// XM_001060520 /// XM_001080537 /// XM_001080543 /// XM_001080549	1q53	2.72	0.000
1388338_at	protein phosphatase 2A, regulatory subunit B (PR 53) (predicted)	Ppp2r4_predicted	1087	399	NM_001108577 /// XM_001077588 /// XM_342405	3p12	2.72	0.001
1383845_at	---	---	1010	371	AI059929	---	2.72	0.004
1383685_at	HEAT repeat containing 1 (predicted)	Heatr1_predicted	2760	1015	NM_001108418 /// XM_001061994 /// XM_341546	17q12.1	2.72	0.000
1398994_at	tyrosylprotein sulfotransferase 2	Tpst2	2705	995	NM_001008508	19p11	2.72	0.000
1378056_at	geminin (predicted)	Gmnn_predicted	2924	1077	NM_001106112 /// XM_001063272 /// XM_214477	17p11	2.72	0.000
1383106_at	cullin 2 (predicted)	Cul2_predicted	1651	609	NM_001108417 /// XM_001057445 /// XM_341542	17q12.1	2.71	0.000
1389228_at	similar to RIKEN cDNA 2010309E21 (predicted)	RGD1304825_predicted	11325	4186	NM_001106605 /// XM_001074564 /// XM_216186	4q34	2.71	0.001
1392906_at	ubiquitin-like 4a (predicted)	Ubl4a_predicted	1130	418	NM_001106346 /// XM_001055202 /// XM_215228	Xq37	2.70	0.001
1377846_a_at	protein kinase, cAMP-dependent, regulatory, type 2, alpha	Msh6_predicted	3408	1261	AA819391	6	2.70	0.000
1393719_at	Transcribed locus	---	1386	513	BF412036	---	2.70	0.028
1370136_at	lamin B receptor	Lbr	1913	710	NM_134453	13q26	2.70	0.000
1368318_at	homer homolog 1 (Drosophila)	Homer1	870	323	NM_031707 /// XM_001068911	2q12	2.69	0.000
1370213_at	Y box protein 1 /// similar to Nuclease sensitive element-binding protein 1 (Y-box-binding protein 1) (Y-box transcription factor) (YB-1) (CCAAT-binding transcription factor I subunit A) (CBF-A) (Enhancer factor I subunit A) (EFI-A) (DNA-binding protein B) (DBPB)...	LOC686530 /// Ybx1	14927	5541	NM_031563 /// XM_001055707 /// XM_001074611	5q36	2.69	0.006
1383305_at	---	---	1895	703	AW530081	10	2.69	0.001
1367960_at	ADP-ribosylation factor-like 4A	Arl4a	2286	851	NM_019186	6q21	2.69	0.001
1368404_at	drebrin 1	Dbn1	3542	1318	NM_031024	17p14	2.69	0.002
1382117_at	similar to hypothetical protein FLJ21148 (predicted)	RGD1560538_predicted	1069	398	NM_001106167 /// XM_001056961 /// XM_214633	19p13	2.69	0.004

Table S2. Trophoblast stem-associated genes

Affymetrix ID	Gene Title	Gene Symbol	Stem Ave	Dif Ave	GenBank	romosomal Locat	Stem/ Di	t-test
1377727_at	bromodomain adjacent to zinc finger domain, 1A (predicted)	Baz1a_predicted	2109	785	XM_001079067 /// XM_234156	6q23	2.69	0.000
1372331_at	similar to eukaryotic translation initiation factor 1A, Y-linked (predicted)	RGD1560198_predicted	2641	985	NM_001106963 /// XM_001054716 /// XM_217622	Xq21	2.68	0.000
1372076_at	hepatitis B virus x interacting protein (predicted)	Hbxip_predicted	8606	3210	NM_001106462 /// XM_001070674 /// XM_215674	2q34	2.68	0.000
1376206_at	Transcribed locus	---	2575	961	BF398576	---	2.68	0.001
1378127_at	cullin 2 (predicted)	Cul2_predicted	1539	574	NM_001108417 /// XM_001057445 /// XM_341542	17q12.1	2.68	0.001
1398349_at	adenylate kinase 2	Ak2	5669	2120	NM_001033967 /// NM_030986	5q36	2.67	0.001
1392732_at	nucleolar and spindle associated protein 1 (predicted)	Nusap1_predicted	1546	578	NM_001107762 /// XM_001075591 /// XM_230478	3q35	2.67	0.002
1372697_at	mitochondrial ribosomal protein S15	Mrps15	1705	638	NM_001007653	5q36	2.67	0.000
1371246_at	nuclear transport factor 2	NTF2	4854	1818	NM_001007629	19q12	2.67	0.000
1370964_at	argininosuccinate synthetase	Ass	19682	7375	NM_013157	3p12	2.67	0.001
1371365_at	ubiquitin-conjugating enzyme E2S (predicted)	Ube2s_predicted	11552	4336	NM_001106224 /// XM_001071810 /// XM_214806	1q12	2.66	0.002
1391050_at	metal response element binding transcription factor 2	Mtf2	2229	838	XM_001056894 /// XM_341180	14p22	2.66	0.001
1383238_at	queuine tRNA-ribosyltransferase 1	Qtrt1	1119	421	NM_022250	8q13	2.66	0.001
1383202_at	similar to U6 snRNA-associated Sm-like protein LSm6 (Sm protein F) (predicted)	RGD1561937_predicted	4984	1877	XM_001070778 /// XM_001070822 /// XM_001070871 /// XM_574220	19q11	2.66	0.000
1373980_at	similar to XPA binding protein 1	LOC688393	864	326	XM_001066740	6q13	2.65	0.001
1391045_at	diacylglycerol O-acyltransferase homolog 2 (mouse)	Dgat2	1401	530	NM_001012345	1q32	2.65	0.002
1395394_at	golgi associated, gamma adaptin ear containing, ARF binding protein 2	Gga2	2895	1095	XM_001072617 /// XM_001076216 /// XM_001076241 /// XM_215045	1q36	2.64	0.003
1388750_at	transferrin receptor	Tfrc	15757	5961	XM_001072774 /// XM_340999	11q22	2.64	0.001
1399047_at	mitochondrial ribosomal protein L27 (predicted)	Mrpl27_predicted	4746	1796	NM_001105831 /// XM_001081298 /// XM_213439	10q31	2.64	0.000
1367818_at	coenzyme Q3 homolog, methyltransferase (yeast)	Coq3	1621	614	NM_019187	5q21	2.64	0.000
1391573_at	tumor necrosis factor receptor superfamily, member 21 (predicted)	Tnfrsf21_predicted	1681	639	NM_001108207 /// XM_001067689 /// XM_236992	9q12	2.63	0.001
1370006_at	NADH dehydrogenase (ubiquinone) Fe-S protein 6	LOC679739 /// LOC692052 /// Ndufs6	4613	1754	NM_019223 /// XM_001053017 /// XM_001053018	17p14 /// 2q12	2.63	0.000
1369931_at	pyruvate kinase, muscle /// similar to Pyruvate kinase isozyme M2 /// similar to pyruvate kinase (EC 2.7.1.40) isozyme M2 - rat (predicted) /// similar to Pyruvate kinase isozymes M1/M2 (Pyruvate kinase muscle isozyme)	LOC499261 /// LOC681135 /// Pkm2 /// RGD1561179_predicted	15366	5847	NM_053297 /// XM_001060445 /// XM_574742 /// XR_007180 /// XR_009310	1q36 /// 20p11 /// 4	2.63	0.001

Table S2. Trophoblast stem-associated genes

Affymetrix ID	Gene Title	Gene Symbol	Stem Ave	Dif Ave	GenBank	romosomal Locat	Stem/ Di	t-test
1384185_at	similar to RIKEN cDNA 2410016O06 (predicted)	RGD1307704_predicted	1995	760	NM_001108040 /// XM_001057487 /// XM_234396	6q31	2.62	0.000
1367997_at	ClpB caseinolytic peptidase B homolog (E. coli)	Clpb	1013	386	NM_022947	1q32	2.62	0.001
1378104_at	similar to RIKEN cDNA 8430406I07	RGD1307465	1289	492	NM_001009655	3q41	2.62	0.001
1372408_at	golgi associated, gamma adaptin ear containing, ARF binding protein 2	Gga2	6454	2463	XM_001072617 /// XM_001076216 /// XM_001076241 /// XM_215045	1q36	2.62	0.000
1388959_at	---	---	2138	817	BI297049	7	2.62	0.000
1373400_at		Prkar2a	2801	1071	AW918535	8	2.61	0.000
1387151_at	nucleoporin 107	Nup107	1680	644	NM_053830	7q22	2.61	0.000
1388960_at	pyrophosphatase	Pyp	11909	4567	XM_001055932 /// XM_215416	20q11	2.61	0.000
1379348_at	BCL2-associated transcription factor 1	Exosc2_predicted	1218	467	BF420491	3	2.61	0.000
1377676_at	nuclear ubiquitous casein kinase and cyclin-dependent kinase substrate	Nucks	9138	3508	NM_022799	6q33	2.61	0.000
1374802_at	similar to chromosome 14 open reading frame 94	RGD1305288	1558	598	NM_001013995	15p13	2.60	0.000
1371935_at	transmembrane protein 9 (predicted)	Tmem9_predicted	931	357	NM_001105953 /// XM_001063617 /// XM_213883	13q13	2.60	0.000
1398997_at	phosphohistidine phosphatase 1 (predicted)	Phpt1_predicted	2189	841	NM_001106558 /// XM_001074086 /// XM_216013	3p13	2.60	0.000
1390335_at	Transcribed locus	---	2257	868	AW252589	8	2.60	0.010
1372398_at	similar to potassium channel tetramerisation domain containing 15	LOC499129	2168	834	NM_001109141 /// XM_001077040 /// XM_001077060 /// XM_001077071 /// XM_574422	1q21	2.60	0.000
1374072_at	homolog of zebrafish ES1	Vezf1_predicted	4488	1728	AI010272	---	2.60	0.002
1370200_at	glutamate dehydrogenase 1	Glud1	2412	929	NM_012570	16p16	2.60	0.001
1389758_at	transcriptional adaptor 2 (ADA2 homolog, yeast)-like	Tada2l	1023	394	NM_001012141 /// XM_001081090	10q26	2.60	0.000
1398303_s_at	tropomyosin 3, gamma	Tpm3	7427	2864	NM_057208 /// NM_173111	2q34	2.59	0.007
1370353_at	translocase of inner mitochondrial membrane 22 homolog (yeast)	Timm22	1416	547	XM_001080619 /// XM_340856	10q26	2.59	0.000
1388528_at	fibrillarin	Fbl	13455	5200	NM_001025643	1q21	2.59	0.000
1378264_at	Nuclear autoantigenic sperm protein (histone-binding)	Nasp	2371	917	NM_001005543	5q36	2.58	0.000
1374713_at	transforming growth factor, beta receptor II	Hrmt1l6_predicted	1972	764	AI228970	---	2.58	0.001
1398753_at	aldo-keto reductase family 1, member A1	Akr1a1	4402	1707	NM_031000	5q36	2.58	0.000
1371777_at	poly A binding protein, cytoplasmic 4	Pabpc4	7615	2964	XM_001054606 /// XM_216517	5q36	2.57	0.000
1371853_at	mitochondrial ribosomal protein L42 (predicted)	Mrpl42_predicted	2290	892	NM_001106782 /// XM_001077569 /// XM_216882	7q13	2.57	0.000
1379541_at	similar to hypothetical protein FLJ14624 (predicted)	RGD1560183_predicted	1146	446	XM_001079115 /// XM_224552	15q25	2.57	0.000
1367548_at	similar to FLJ00052 protein (predicted)	RGD1305986_predicted	1804	703	XM_001080131 /// XM_341092	12q16	2.56	0.001

Table S2. Trophoblast stem-associated genes

Affymetrix ID	Gene Title	Gene Symbol	Stem Ave	Dif Ave	GenBank	romosomal Locat	Stem/ Di	t-test
1367575_at	enolase 1, alpha	Eno1	19853	7744	NM_001109908 /// NM_012554	5q36	2.56	0.000
1373138_at	nudix (nucleoside diphosphate linked moiety X)-type motif 5	Nudt5	3049	1189	NM_001007733	17q12.3	2.56	0.000
1376055_at	minichromosome maintenance deficient 5, cell division cycle 46 (S. cerevisiae) (predicted)	Mcm5_predicted	2234	873	NM_001106170 /// XM_001064207 /// XM_226316	19p11	2.56	0.000
1389869_at	similar to C184L-22	LOC684594 /// LOC689397	1581	618	NM_001109537 /// XM_001073780 /// XM_001074570	1q43	2.56	0.000
1373823_at	similar to Cyclin-dependent kinases regulatory subunit 2 (CKS-2) (predicted)	RGD1562047_predicted	17206	6725	XM_001054024 /// XM_573992	17p14	2.56	0.004
1397647_at	solute carrier family 25 (mitochondrial carrier; ornithine transporter) member 15	Slc25a15	1056	413	NM_001047880 /// XM_001064456 /// XM_001064514 /// XM_001064627 /// XM_001064682 /// XM_224969	16q12.5	2.55	0.000
1389983_at	similar to cDNA sequence AF155546	RGD1309387	1789	700	NM_001033897	5q36	2.55	0.000
1389625_at	coiled-coil-helix-coiled-coil-helix domain containing 4	Chchd4	1224	479	NM_001013431	4q34	2.55	0.000
1389337_at	Transcribed locus	---	2159	846	AA946032	---	2.55	0.000
1367798_at	S-adenosylhomocysteine hydrolase	Ahcy	4817	1889	NM_017201	3q42	2.55	0.001
1371754_at	solute carrier family 25 (mitochondrial carrier, phosphate carrier), member 25	Slc25a25	1174	461	NM_145677	3p11	2.55	0.006
1370303_at	solute carrier family 35, member A4	Slc35a4	1428	561	NM_147140	18p11	2.55	0.000
1372730_at	similar to Mitochondrial 39S ribosomal protein L39 (L39mt) (MRP-L39)	LOC684304	3121	1226	XM_001069799	19	2.55	0.000
1369775_at	nuclear ubiquitous casein kinase and cyclin-dependent kinase substrate	Nucks	4326	1703	NM_022799	6q33	2.54	0.006
1372893_at	Yip1 domain family, member 1	Yipf1	1877	740	NM_199383	5q34	2.54	0.000
1370057_at	cysteine and glycine-rich protein 1	Csrp1	2701	1064	NM_017148	13q13	2.54	0.008
1371850_at	3'-phosphoadenosine 5'-phosphosulfate synthase 1 (predicted)	Papss1_predicted	1574	621	NM_001106471 /// XM_001077181 /// XM_215701	2q43	2.54	0.000
1399143_at	ubiquitin-conjugating enzyme E2N	Ube2n	6512	2568	NM_053928 /// XM_001077606	7q13	2.54	0.002
1367479_at	similar to nucleolar protein family A, member 3	LOC691534	9673	3816	XM_001078710 /// XM_001080488	3q34	2.53	0.003
1377627_at	similar to down-regulated in metastasis (predicted)	RGD1560606_predicted	1096	434	XM_235023 /// XR_009342	7q13	2.53	0.000
1373654_at	annexin A8	Anxa8	3488	1380	NM_001031654	16p16	2.53	0.013
1390902_at	Transcribed locus	---	1670	661	BI275056	---	2.53	0.001
1383116_at	similar to RIKEN cDNA 4833435D08	LOC300284	1643	651	NM_001013951	Xq14	2.52	0.012
1374393_at	protein tyrosine phosphatase-like (proline instead of catalytic arginine), member b (predicted) /// similar to protein tyrosine phosphatase-like (proline instead of catalytic arginine), member b	LOC690040 /// Ptplb_predicted	2081	824	XM_001071670 /// XM_001073002 /// XM_213610	11q22	2.52	0.000
1376579_at	leucine aminopeptidase 3	Lap3	1343	533	NM_001011910	14q21	2.52	0.005
1389765_at	notchless homolog 1 (Drosophila) (predicted)	Nle1_predicted	1364	542	XM_001081007 /// XM_220770	10q26	2.52	0.001
1389279_at	similar to Hypothetical UPF0080 protein KIAA0186 (predicted)	Ran	3050	1212	BM386204	10	2.52	0.000

Table S2. Trophoblast stem-associated genes

Affymetrix ID	Gene Title	Gene Symbol	Stem Ave	Dif Ave	GenBank	romosomal Locat	Stem/ Dif	t-test
1388641_at	phosphoribosylglycinamide formyltransferase	Gart	1913	761	XM_001068637 /// XM_573258	11q11	2.51	0.000
1395967_at	similar to LDL receptor adaptor protein (predicted)	RGD1563417_predicted	863	344	NM_001109271 /// XM_001067557 /// XM_575931	5q36	2.51	0.002
1389265_at	glucan (1,4-alpha-), branching enzyme 1	Gbe1	1717	685	XM_001064453 /// XM_221747	11p11	2.51	0.000
1374415_at	polymerase (RNA) III (DNA directed) polypeptide E (predicted)	Polr3e_predicted	1077	431	NM_001108503 /// XM_001075970 /// XM_341919	1q35	2.50	0.001
1367663_at	proteasome (prosome, macropain) 28 subunit, alpha	Psme1	1410	565	NM_017264	15p13	2.50	0.000
1379626_at	Special AT-rich sequence binding protein 1	Satb1	16935	6786	NM_001012129	9q11	2.50	0.001
1395560_at	Transcribed locus	---	2234	895	BF522151	---	2.50	0.001
1389544_at	similar to translocase of outer mitochondrial membrane 7 homolog	LOC685620	6826	2736	XM_001064547	4q11	2.50	0.000
1390272_at	DPH5 homolog (S. cerevisiae)	Dph5	1204	483	NM_001017449	2q41	2.49	0.000
1380062_at	membrane protein, palmitoylated 6 (MAGUK p55 subfamily member 6) (predicted)	Mpp6_predicted	1727	693	XM_001056864 /// XM_342682	4q24	2.49	0.012
1393241_at	protease, serine, 32 (predicted)	Prss32_predicted	3991	1605	NM_001106983 /// XM_001056587 /// XM_220209	10q12	2.49	0.000
1368054_at	lamin A	Lmna	808	325	NM_001002016	2q31-q34	2.49	0.011
1381672_at	similar to LDL receptor adaptor protein (predicted)	RGD1563417_predicted	1734	699	NM_001109271 /// XM_001067557 /// XM_575931	5q36	2.48	0.000
1379592_at	similar to citrin (predicted)	RGD1565889_predicted	974	393	XM_001054092 /// XM_342640	4q13	2.48	0.002
1368302_at	homeo box, msh-like 1	Msx1	1797	725	NM_031059	14q21	2.48	0.010
1375268_at	Transcribed locus, strongly similar to XP_996369.1 PREDICTED: similar to 40S ribosomal protein S27-like protein [Mus musculus]	---	1694	683	AA858591	---	2.48	0.014
1382471_at	similar to hypothetical protein MGC10993	RGD1310320	846	342	NM_001037776	13q11	2.48	0.000
1382975_at	CEA-related cell adhesion molecule 1	Ceacam1	1176	475	NM_001033860 /// NM_001033861 /// NM_001033862 /// NM_031755	1q21-q22	2.48	0.000
1396013_at	leucine zipper-EF-hand containing transmembrane protein 1	Letm1	921	372	NM_001005884	14q21	2.48	0.005
1370030_at	glutamate cysteine ligase, modifier subunit	Gclm	3435	1389	NM_017305	2q41	2.47	0.000
1376407_a_at	LSM7 homolog, U6 small nuclear RNA associated (S. cerevisiae) (predicted)	Lsm7_predicted	2025	820	NM_001108732 /// XM_001076086 /// XM_343157	7q11	2.47	0.002
1370699_a_at	epidermal growth factor receptor /// peptidase D	Egfr /// Pepd	1524	618	NM_001009641 /// NM_031507	14q22 /// 1q21	2.47	0.000
1389408_at	ribonucleotide reductase M2	Rrm2	20710	8415	NM_001025740	6q16	2.46	0.003
1382443_at	similar to Pabpc4_predicted protein (predicted) /// poly A binding protein, cytoplasmic 4	Pabpc4 /// RGD1562451_predicted	3223	1313	XM_001054606 /// XM_216517 /// XR_008296	12p12 /// 5q36	2.46	0.000
1371390_at	tubulin, beta 2c	Tubb2c	16301	6640	NM_199094	3p13	2.45	0.002
1388560_at	WD repeat domain 77	Wdr77	6672	2723	NM_001008771	2q34	2.45	0.000

Table S2. Trophoblast stem-associated genes

Affymetrix ID	Gene Title	Gene Symbol	Stem Ave	Dif Ave	GenBank	romosomal Locat	Stem/ Di	t-test
1370008_at	proteasome (prosome, macropain) 26S subunit, ATPase 3, interacting protein	Psmc3ip	1061	433	NM_134458	10q32.1	2.45	0.000
1391466_at	mutS homolog 6 (E. coli) (predicted)	Nucks	1978	808	AI170347	15	2.45	0.000
1374844_at	RNA binding motif protein 14	Rbm14	2344	958	XM_001072105	---	2.45	0.004
1378638_a_at	similar to 2700029M09Rik protein (predicted)	RGD1311747_predicted	2523	1031	XM_001053568 /// XM_214341	16p12	2.45	0.001
1389622_at	similar to citrin (predicted)	RGD1565889_predicted	2305	945	XM_001054092 /// XM_342640	4q13	2.44	0.001
1370309_a_at	heterogeneous nuclear ribonucleoprotein A/B	Hnrpab	13579	5566	NM_031330	10q22	2.44	0.000
1373452_at	RNA terminal phosphate cyclase-like 1	Rcl1	2048	840	NM_001013152	1q52	2.44	0.000
1399005_at	protein phosphatase 2, regulatory subunit B (B56), alpha isoform (predicted)	Ppp2r5a_predicted	1193	490	NM_001107891 /// XM_001066948 /// XM_232413	13q27	2.44	0.001
1390237_at	translocase of inner mitochondrial membrane 8 homolog a (yeast)	Timm8a	2135	877	NM_053370	Xq34	2.44	0.006
1388482_at	similar to RIKEN cDNA 9130404D14	RGD1307018	2983	1225	NM_001109885 /// XM_001078459 /// XM_342417	3p11	2.44	0.000
1371962_at	Tu translation elongation factor, mitochondrial (predicted)	Tufm_predicted	3502	1438	NM_001106295 /// XM_001077188 /// XM_215069	1q36	2.44	0.000
1382679_at	similar to WD-repeat protein 43	LOC362703	2751	1130	XM_001059356 /// XM_001061088	6q13	2.43	0.011
1371632_at	similar to Coronin, actin binding protein 1C (predicted)	RGD1564490_predicted	7727	3176	NM_001109327 /// XM_001080440 /// XM_577254	12q16	2.43	0.013
1371752_at	similar to Ran-interacting protein MOG1 (predicted)	RGD1563195_predicted	1576	648	NM_001105790 /// XM_001078960 /// XM_213367	10q24	2.43	0.001
1371548_at	mitochondrial ribosomal protein S25	Mrps25	1959	806	NM_001025408	4q34	2.43	0.000
1388178_at	nuclear receptor coactivator 3	Ncoa3	1741	717	XM_001072953 /// XM_215947	3q42	2.43	0.002
1399161_a_at	type 1 tumor necrosis factor receptor shedding aminopeptidase regulator	Arts1	1631	672	NM_030836	2q11	2.43	0.001
1382592_at	similar to RIKEN cDNA 2600001J17	RGD1306908	1394	574	NM_001034138	10q12	2.43	0.000
1376606_a_at	similar to e(y)2 protein	LOC682575 /// LOC685258	3324	1370	XM_001062411 /// XM_001062468 /// XM_001062808 /// XM_001062869	7q31	2.43	0.000
1388744_at	minichromosome maintenance deficient 7 (S. cerevisiae)	Mcm7	5481	2262	NM_001004203	12q11	2.42	0.000
1385020_at	similar to DNA segment, Chr 19, Brigham & Womens Genetics 1357 expressed (predicted) /// hypothetical protein LOC499339 /// similar to D19Bwg1357e protein	LOC499339 /// LOC683670 /// RGD1308750_predicted	1625	671	NM_001025036 /// XM_001066994 /// XM_215276	1q51 /// Xq37	2.42	0.000
1379296_at	Transcribed locus	---	926	382	AI137435	---	2.42	0.002
1396086_at	transmembrane protein 97	Tmem97	2427	1004	NM_001008334	10q25	2.42	0.000
1375246_at	hypothetical protein LOC679747 /// hypothetical protein LOC685117	LOC679747 /// LOC685117	10244	4243	XM_001055857 /// XM_001055910 /// XM_001062375 /// XM_001076275 /// XM_001076301	4q24 /// 8q32	2.41	0.004

Table S2. Trophoblast stem-associated genes

Affymetrix ID	Gene Title	Gene Symbol	Stem Ave	Dif Ave	GenBank	romosomal Locat	Stem/ Di	t-test
1393972_at	metal response element binding transcription factor 2	Mtf2	860	356	XM_001056894 /// XM_341180	14p22	2.41	0.004
1372554_at	similar to RW1 protein (predicted)	RGD1309266_predicted	1256	521	XM_001056771 /// XM_237056	9q21	2.41	0.000
1392717_at	exosome component 2 (predicted)	Kif1b	831	345	BE109171	5	2.41	0.000
1371350_at	similar to S-adenosylmethionine synthetase isoform type-2 (Methionine adenosyltransferase 2) (AdoMet synthetase 2) (Methionine adenosyltransferase II) (MAT-II)	LOC683283	4877	2027	XM_001065243	---	2.41	0.000
1376202_at	UDP-GlcNAc:betaGal beta-1,3-N-acetylglucosaminyltransferase 6 (predicted)	B3gnt6_predicted	804	334	NM_001106324 /// XM_001072516 /// XM_219693	1q43	2.41	0.000
1373705_at	similar to 39S ribosomal protein L28, mitochondrial precursor (L28mt) (MRP-L28)	LOC497876	2378	992	XM_001061401 /// XM_573060	10q12	2.40	0.001
1371693_at	similar to AHA1, activator of heat shock 90kDa protein ATPase homolog 1	LOC681996	4975	2075	XM_001062110 /// XM_001062172	---	2.40	0.000
1389288_at	NADH dehydrogenase (ubiquinone) 1 alpha subcomplex, 2 (predicted)	Ndufa2_predicted	3394	1418	NM_001106153 /// XM_001064098 /// XM_214570	18p11	2.39	0.000
1367586_at	lactate dehydrogenase A	Ldha	18791	7858	NM_017025 /// XM_001080828	1q22	2.39	0.001
1371967_at	mitochondrial ribosomal protein L16	Mrpl16	2451	1026	NM_001009647	1q43	2.39	0.000
1375630_at	similar to NHP2-like protein 1 (High mobility group-like nuclear protein 2 homolog 1) ([U4/U6.U5] tri-snRNP 15.5 kDa protein) (OTK27)	MGC72932	9084	3811	NM_212515	7q34	2.38	0.001
1390587_x_at	sprouty protein with EVH-1 domain 1, related sequence	Musk	3632	1525	AW525862	---	2.38	0.000
1385853_at	bromodomain adjacent to zinc finger domain, 1A (predicted)	Baz1a_predicted	1049	441	XM_001079067 /// XM_234156	6q23	2.38	0.000
1367883_at	survival of motor neuron 1, telomeric	Smn1	2995	1260	NM_022509	2q12	2.38	0.000
1373870_at	similar to RIKEN cDNA 2810405J04	RGD1305486	2779	1169	NM_001014073	6q12	2.38	0.000
1388129_at	structure specific recognition protein 1	Ssrp1	4805	2022	NM_031121	3q24	2.38	0.001
1371662_at	lysyl-tRNA synthetase	Kars	7801	3283	NM_001006967	19q12	2.38	0.000
1377745_at	similar to hypothetical protein FLJ20331	LOC310946	1652	696	NM_001034926 /// XM_001078514 /// XM_227795	2q45	2.37	0.000
1397255_at	similar to hypothetical protein MGC2574	MGC94720	939	396	NM_001006974	1q43	2.37	0.000
1391205_at	similar to hypothetical protein FLJ20397	MGC125214	1805	761	XM_001055205 /// XM_001065453	12q11	2.37	0.020
1372185_at	similar to RIKEN cDNA 2610205E22	RGD1306582	3503	1479	NM_001025019	3p12	2.37	0.000
1372644_at	similar to CG14286-PA	LOC686765	1850	781	XM_001075602	7	2.37	0.001
1371743_at	guanylate kinase 1	Guk1	3132	1324	NM_001013115	10q22	2.37	0.000
1388709_at	similar to WD-repeat protein 43	LOC362703	2806	1186	XM_001059356 /// XM_001061088	6q13	2.37	0.001
1373538_at	ubiquitin specific peptdiase 1	Usp1	1864	789	NM_001015015	5q33	2.36	0.001
1383477_at	ubiquitin carboxyl-terminal hydrolase L5	Uchl5	5344	2264	NM_001012149	13q21	2.36	0.002

Table S2. Trophoblast stem-associated genes

Affymetrix ID	Gene Title	Gene Symbol	Stem Ave	Dif Ave	GenBank	romosomal Locat	Stem/ Dif	t-test
1389964_at	NADH dehydrogenase (ubiquinone) 1, alpha/beta subcomplex, 1 (predicted) /// similar to Acyl carrier protein, mitochondrial precursor (ACP) (NADH-ubiquinone oxidoreductase 9.6 kDa subunit) (CI-SDAP)	LOC683884 /// Ndufab1_predicted	6280	2663	NM_001106294 /// XM_001067919 /// XM_001079490 /// XM_215044	1q36	2.36	0.000
1377706_x_at	---	---	1508	640	BF549971	---	2.36	0.010
1374836_at	RNA, U3 small nucleolar interacting protein 2 (predicted)	Rnu3ip2_predicted	1952	828	NM_001108778 /// XM_001071554 /// XM_343469	8q32	2.36	0.001
1395404_at	Similar to Hypothetical protein 9030012M21 (predicted)	RGD1304570_predicted	800	340	NM_001107077 /// XM_001081841 /// XM_221214	10q32.3	2.35	0.000
1396250_at	similar to Coronin, actin binding protein 1C (predicted)	RGD1564490_predicted	868	369	NM_001109327 /// XM_001080440 /// XM_577254	12q16	2.35	0.020
1389738_at	uracil-DNA glycosylase	Ung	1840	783	NM_001013124	12q16	2.35	0.002
1372631_at	thymidine kinase 1	Tk1	898	382	NM_052800 /// XM_001081763	---	2.35	0.003
1388532_at	similar to hypothetical protein	RGD1310571	1167	497	NM_001014147	18p12	2.35	0.003
1385428_at	exportin 4 (predicted)	Xpo4_predicted	1714	730	NM_001106042 /// XM_001063318 /// XM_214191	15p12	2.35	0.000
1372430_at	similar to RIKEN cDNA 1700037H04	RGD1311739	1161	495	NM_001025691	3q36	2.35	0.005
1376098_a_at	myosin IG	Myo1g	3387	1443	XM_001069724 /// XM_573653	14q21	2.35	0.001
1379330_s_at	similar to Pterin-4-alpha-carbinolamine dehydratase 2 (PHS 2) (4-alpha-hydroxy-tetrahydropterin dehydratase 2) (DcoH-like protein DCoHm) (Dimerization cofactor of hepatocyte nuclear factor 1 from muscle) (HNF1-alpha dimerization cofactor)...	LOC683319	1929	822	XM_001063921	---	2.35	0.000
1368032_at	nucleolar and coiled-body phosphoprotein 1	Nolc1	6136	2615	NM_022869	1q54	2.35	0.000
1374537_at	carbohydrate (chondroitin) synthase 1 (predicted)	Chsy1_predicted	5370	2291	NM_001106268 /// XM_001059243 /// XM_218759	1q22	2.34	0.000
1389393_at	similar to RIKEN cDNA 2210412D01	RGD1308210	2689	1148	XM_001068403 /// XM_341879	1q31	2.34	0.000
1368042_a_at	high mobility group box 1 /// similar to High mobility group protein 1 (HMG-1) (predicted) /// similar to Hmgb1 protein (predicted) /// hypothetical protein LOC678705 /// similar to High mobility group protein 1 (HMG-1) (High mobility group protein B1) (Amphoterin) (Heparin-binding protein p30)	Hmgb1 /// LOC678705 /// LOC679451 /// LOC679571 /// LOC680054 /// LOC680765 /// LOC680968 /// LOC681718 /// LOC685520 /// RGD1562312_predicted /// RGD1563012_predicted /// RGD1563786_predicted	13965	5971	NM_001109373 /// NM_012963 /// XM_001053005 /// XM_001053260 /// XM_001053326 /// XM_001055841 /// XM_001056424 /// XM_001058093 /// XM_001058770 /// XM_001059167 /// XM_001059688 /// XM_001067678 /// XM_001072913 /// XR_008108 /// XR_008751	12p11 /// 16p11 ///	2.34	0.001

Table S2. Trophoblast stem-associated genes

Affymetrix ID	Gene Title	Gene Symbol	Stem Ave	Dif Ave	GenBank	romosomal Locat	Stem/ Di	t-test
1375639_at	Transcribed locus, strongly similar to XP_947674.1 PREDICTED: similar to E2F transcription factor 6 isoform 1 [Homo sapiens]	---	1590	680	AA850881	---	2.34	0.001
1393584_at	tumor necrosis factor receptor superfamily, member 21 (predicted)	Tnfrsf21_predicted	2821	1207	NM_001108207 /// XM_001067689 /// XM_236992	9q12	2.34	0.002
1389815_at	protein phosphatase 1, regulatory (inhibitor) subunit 14B	Ppp1r14b	5331	2281	NM_172045 /// XM_001074638	1q43	2.34	0.000
1376395_at	Transcribed locus	---	2623	1123	AI145359	---	2.34	0.000
1373585_at		RGD1310781_predicted	1621	695	BE116774	6	2.33	0.001
1373017_at	succinate-Coenzyme A ligase, GDP-forming, beta subunit	Suclg2	2706	1160	XM_001064568 /// XM_001074487 /// XM_001074521 /// XM_001074551 /// XM_001074572	4q34	2.33	0.000
1370462_at	hyaluronan mediated motility receptor (RHAMM)	Hmmr	972	417	NM_012964	10q12	2.33	0.001
1384281_at	Transcribed locus	---	3159	1358	BI295209	---	2.33	0.000
1383251_at	poly (ADP-ribose) polymerase family, member 2 (predicted)	Parp2_predicted	1571	675	NM_001106030 /// XM_001063825 /// XM_214157	15p14	2.33	0.000
1388988_at	abhydrolase domain containing 14b	Abhd14b	1025	441	NM_001007664	8q32	2.33	0.000
1390805_at	hypothetical protein LOC687491 /// hypothetical protein LOC689755	LOC687491 /// LOC689755	985	424	NM_001109548 /// XM_001071892 /// XM_001078820	20p12	2.32	0.001
1391415_at	putatative 28 kDa protein	LOC289809	5165	2225	NM_199083	14q22	2.32	0.003
1367751_at	dolichol-phosphate (beta-D) mannosyltransferase 2	Dpm2	1277	550	NM_019252	---	2.32	0.000
1395719_at	Tu translation elongation factor, mitochondrial (predicted)	Tufm_predicted	2030	875	NM_001106295 /// XM_001077188 /// XM_215069	1q36	2.32	0.000
1372188_at	similar to hypothetical protein D15Ertd785e	MGC114417	1071	462	NM_001024877 /// XM_001055014	7q34	2.32	0.001
1367870_at	thioredoxin-like 2	Txnl2	12009	5194	NM_032614	1q41	2.31	0.001
1373516_at	similar to RIKEN cDNA 2600005C20 (predicted)	RGD1305633_predicted	1555	673	XM_001071108 /// XM_228076	20p12	2.31	0.011
1373954_at	similar to FLJ00052 protein (predicted)	RGD1305986_predicted	4349	1886	XM_001080131 /// XM_341092	12q16	2.31	0.001
1371887_at	similar to high mobility group protein homolog HMG4 (predicted)	RGD1564407_predicted	1266	549	XM_001067329 /// XM_223440	Xq37	2.30	0.001
1383353_at	ephrin B2 (predicted)	Efnb2_predicted	1354	588	NM_001107328 /// XM_001067856 /// XM_225050	16q12.5	2.30	0.000
1368647_at	G protein-coupled receptor kinase 6	Gprk6	1147	498	NM_001112712 /// NM_001112713 /// NM_031657	17p14	2.30	0.000
1373965_at	similar to hypothetical protein BC013949 (predicted)	RGD1310931_predicted	1353	588	NM_001106071 /// XM_001066312 /// XM_214300	16p14	2.30	0.000
1371847_at	---	---	1494	649	BI274357	8	2.30	0.000
1374877_at	similar to translocase of inner mitochondrial membrane 50 homolog	LOC687295	3425	1488	XM_001073281 /// XM_001073346	---	2.30	0.001
1390384_at	similar to Histone H2A.x (H2a/x) (predicted)	RGD1566119_predicted	2443	1062	NM_001109291 /// XM_001066891 /// XM_576399	8q22	2.30	0.000

Table S2. Trophoblast stem-associated genes

Affymetrix ID	Gene Title	Gene Symbol	Stem Ave	Dif Ave	GenBank	romosomal Locat	Stem/ Di	t-test
1392668_at	recombining binding protein suppressor of hairless (Drosophila) (predicted) /// similar to Recombining binding protein suppressor of hairless (J kappa-recombination signal binding protein) (RBP-J kappa)	LOC679028 /// Rbpsuh_predicted	1303	566	NM_001106631 /// XM_001053717 /// XM_001053777 /// XM_001053835 /// XM_001064167 /// XM_232595	5q11	2.30	0.010
1388410_at	UDP-glucose pyrophosphorylase 2	Ugp2	8462	3681	NM_001024743	14q22	2.30	0.007
1368055_a_at	lamin A	Lmna	4609	2005	NM_001002016	2q31-q34	2.30	0.000
1386973_a_at	mitogen activated protein kinase 8 interacting protein	Mapk8ip	913	398	NM_053777	3q24	2.30	0.000
1392747_at	caspase 8 associated protein 2 (predicted)	Fbxo30	891	388	AI030920	---	2.29	0.000
1394397_at	sterol O-acyltransferase 1	Gspt1	5170	2256	BF522734	10	2.29	0.000
1386954_at	adenylate kinase 2	Ak2	2615	1141	NM_001033967 /// NM_030986	5q36	2.29	0.000
1392454_at	similar to hypothetical protein FLJ20729 (predicted)	RGD1308723_predicted	1467	640	NM_001106203 /// XM_001079278 /// XM_217694	2q44	2.29	0.001
1389658_at	NOL1/NOP2/Sun domain family, member 2 (predicted)	Nsun2_predicted	6305	2753	NM_001108403 /// XM_001057743 /// XM_341474	17p14	2.29	0.000
1372001_at	similar to Protein FAM96B	LOC680987	1343	587	XM_001058416	19p14	2.29	0.000
1370461_at	hyaluronan mediated motility receptor (RHAMM)	Hmmr	968	423	NM_012964	10q12	2.29	0.000
1386586_at	similar to PAK/PLC-interacting protein 1	MGC125015	5299	2317	NM_001037356	17p12	2.29	0.000
1375059_at	similar to zinc finger protein 652 (predicted)	RGD1566329_predicted	1305	571	NM_001080207 /// XM_001081321 /// XM_573180	10q31	2.28	0.000
1376684_at	discs, large homolog 7 (Drosophila) (predicted)	Dlg7_predicted	1914	839	XM_001073486 /// XM_223937	15p14	2.28	0.001
1368977_a_at	fractured callus expressed transcript 1	Fxc1	847	372	NM_053371	1q33	2.28	0.002
1372406_at	minichromosome maintenance deficient 3 (S. cerevisiae) (predicted) /// similar to DNA replication licensing factor MCM3 (DNA polymerase alpha holoenzyme-associated protein P1) (P1-MCM3)	LOC367976 /// Mcm3_predicted	2268	996	XM_001070728 /// XM_236988 /// XM_346381	9q13	2.28	0.000
1371838_at	similar to splicing factor, arginine/serine-rich 2	Sfrs2	10034	4406	NM_001009720	10q32.3	2.28	0.000
1373668_at	polymerase (RNA) II (DNA directed) polypeptide I (predicted)	Polr2i_predicted	3281	1442	NM_001106244 /// XM_001075773 /// XM_214895	1q21	2.28	0.000
1381885_at	Transcribed locus	---	1604	705	BE102910	---	2.28	0.001
1382144_at	mitochondrial ribosomal protein L47	Mrpl47	2123	933	NM_001037183	2q25	2.28	0.000
1393431_at	similar to Splicing factor U2AF 35 kDa subunit (U2 auxiliary factor 35 kDa subunit) (U2 snRNP auxiliary factor small subunit)	LOC687575	8603	3783	NM_001044291 /// XM_001079206	7	2.27	0.000
1371448_at	CDNA clone IMAGE:7303896	---	2178	958	AI599295	---	2.27	0.009
1398608_at	similar to hypothetical gene supported by BC007071	RGD1311868	1666	733	NM_001033061	13q22	2.27	0.000
1387085_at	phosphoribosyl pyrophosphate synthetase 1	Prps1	2275	1003	NM_017243	Xq35	2.27	0.000
1371503_at	nucleotide binding protein 1	Nubp1	1993	880	NM_001009619	10q11	2.26	0.000
1389587_at	uridine monophosphate synthetase	Umps	4132	1826	NM_001025402	11q22	2.26	0.000

Table S2. Trophoblast stem-associated genes

Affymetrix ID	Gene Title	Gene Symbol	Stem Ave	Dif Ave	GenBank	romosomal Locat	Stem/ Dif	t-test
1371908_at	NTF2-related export protein 1 (predicted)	Nxt1_predicted	3025	1338	NM_001106521 /// XM_001057228 /// XM_215858	3q41	2.26	0.002
1388567_at	THUMP domain containing 1	Thumpd1	2013	890	NM_001009688 /// XM_001079074	1q35	2.26	0.000
1374199_at	similar to Disco-interacting protein 2 homolog	LOC687682 /// LOC690211	922	408	XM_001070487 /// XM_001070526 /// XR_009314	20p12	2.26	0.009
1375915_at	interleukin-1 receptor-associated kinase 1 binding protein 1 (predicted)	Irak1bp1_predicted	1217	539	NM_001106843 /// XM_001061980 /// XM_217210	8q31	2.26	0.001
1391432_at	WD repeat domain 75	Wdr75	2055	911	NM_001047889 /// XM_001060763 /// XM_234768	9q22	2.26	0.000
1395246_at	aquarius (predicted)	Prkar2a	872	386	AI104783	8	2.26	0.000
1373744_at	anaphase promoting complex subunit 1 (predicted)	Anapc1_predicted	1938	860	NM_001107771 /// XM_001078787 /// XM_230589	3q36	2.25	0.001
1394884_s_at	single-stranded DNA binding protein 1	Ssbp1	3475	1543	NM_183328	4q22	2.25	0.002
1377299_at	nuclear autoantigenic sperm protein (histone-binding)	Nasp	3622	1610	NM_001005543	5q36	2.25	0.001
1374288_at	FtsJ homolog 3 (E. coli)	Ftsj3	3341	1488	NM_001012014 /// XM_001081570	10q32.1	2.25	0.000
1392494_at	similar to hypothetical protein D030056L22	LOC499331	1857	827	NM_001024293	1q43	2.24	0.000
1398894_at	COMM domain containing 3	Commd3	7264	3238	NM_198732 /// XM_001073695	17q12.3	2.24	0.001
1388473_at	Transcribed locus	---	956	427	BF418041	---	2.24	0.016
1389450_at	similar to Williams-Beuren syndrome critical region protein 22 /// similar to Putative methyltransferase WBSCR22 (Williams-Beuren syndrome chromosome region 22 protein homolog)	LOC360830 /// LOC368084	4106	1834	XM_001071583 /// XM_341103 /// XM_347254	12q12	2.24	0.003
1367617_at	aldolase A	Aldoa	15478	6926	NM_012495	1q36	2.23	0.001
1382435_at	fizzy/cell division cycle 20 related 1 (Drosophila) (predicted)	Fzr1_predicted	959	429	NM_001108074 /// XM_001075601 /// XM_243390	7q11	2.23	0.001
1375895_at	growth factor receptor bound protein 2-associated protein 1 (predicted)	Srr	6256	2805	BI275908	10	2.23	0.000
1388783_at	high mobility group box 1	Hmgb1	5329	2391	NM_012963 /// XM_001072913	12p11	2.23	0.003
1398877_at	stress-induced phosphoprotein 1	Stip1	5241	2351	NM_138911	1q43	2.23	0.000
1388163_at	solute carrier family 25 (mitochondrial carrier; adenine nucleotide translocator), member 5	Slc25a5	16999	7627	NM_057102	Xq36	2.23	0.004
1367661_at	S100 calcium binding protein A6 (calcyclin)	S100a6	6024	2709	NM_053485	2q34	2.22	0.001
1376703_at	nucleoporin 37 (predicted)	Nup37_predicted	3291	1481	NM_001106775 /// XM_001076503 /// XM_001076538 /// XM_216872	7q13	2.22	0.000
1367927_at	prohibitin	Phb	8571	3857	NM_031851 /// XM_001053030 /// XM_001053031	Xq14	2.22	0.000

Table S2. Trophoblast stem-associated genes

Affymetrix ID	Gene Title	Gene Symbol	Stem Ave	Dif Ave	GenBank	romosomal Locat	Stem/ Dif	t-test
1371907_at	ADP-ribosylation factor-like 6 interacting protein 4	Arl6ip4	2423	1090	NM_001025630	12q15	2.22	0.002
1386096_at	metal response element binding transcription factor 2	Mtf2	2364	1065	XM_001056894 /// XM_341180	14p22	2.22	0.000
1388954_at	similar to hypothetical protein MGC25461 (predicted)	RGD1306717_predicted	1233	556	NM_001107756 /// XM_001072521 /// XR_006980	3q32	2.22	0.007
1373682_at	DEAD (Asp-Glu-Ala-Asp) box polypeptide 51 (predicted)	Ddx51_predicted	1566	706	NM_001107150 /// XM_001080674 /// XM_222252	12q16	2.22	0.005
1375551_at	similar to hypothetical protein MGC2574	MGC94720	1013	457	NM_001006974	1q43	2.22	0.002
1389312_at	similar to RIKEN cDNA 0610016J10 gene	RGD1309929	3453	1557	NM_001029917	6q13	2.22	0.000
1399067_at	Guanine nucleotide binding protein-like 3 (nucleolar)-like	Gnl3l	1942	876	NM_001081958	Xq14-q21	2.22	0.002
1372709_at	B-cell receptor-associated protein 29	Bcap29	3365	1521	NM_001006980	6q16	2.21	0.000
1371074_a_at	minichromosome maintenance deficient 6 (MIS5 homolog, S. pombe) (S. cerevisiae)	Mcm6	5760	2603	NM_017287 /// XM_001055953 /// XM_344135	13q12	2.21	0.008
1383144_at	tRNA nucleotidyl transferase, CCA-adding, 1	Trnt1	3070	1388	NM_001024261	4q41	2.21	0.000
1371094_at	LIM homeobox protein 2 (predicted)	Lhx2_predicted	1112	504	NM_001106571 /// XM_001054855 /// XM_216050	3q11	2.21	0.014
1390356_at	Transcribed locus	---	5307	2407	AW535401	---	2.20	0.000
1382275_at	similar to PAK/PLC-interacting protein 1	MGC125015	5297	2404	NM_001037356	17p12	2.20	0.000
1393043_at	WD repeat domain 50 (predicted)	Wdr50_predicted	1449	658	XM_001081275 /// XM_220851	10q26	2.20	0.001
1374161_at	importin 11 (predicted)	Ipo11_predicted	1627	739	XM_001064746 /// XM_001064798 /// XM_001064902 /// XM_226752	2q14	2.20	0.000
1383323_at	similar to RIKEN cDNA 1700022L09	LOC499933	835	379	NM_001025768	3q42	2.20	0.001
1368201_at	natriuretic peptide receptor 1	Npr1	1807	821	NM_012613	2q34	2.20	0.046
1388304_at	NADH dehydrogenase (ubiquinone) 1 beta subcomplex, 5 (predicted)	Ndufb5_predicted	4935	2247	NM_001106426 /// XM_001066565 /// XM_215544	2q25	2.20	0.000
1394003_at	polymerase (DNA directed), epsilon 3 (p17 subunit)	Pole3	1607	732	NM_001007652	5q24	2.19	0.001
1371735_at	Transcribed locus, strongly similar to XP_579758.1 PREDICTED: hypothetical protein XP_579758 [Rattus norvegicus]	---	9213	4205	AI105202	---	2.19	0.004
1372688_at	exosome component 7	Exosc7	4198	1922	XM_001078932 /// XM_236745	8q32	2.18	0.000
1377945_at	DEAD (Asp-Glu-Ala-Asp) box polypeptide 18	Ddx18	3534	1621	NM_001006996	13q11	2.18	0.000
1388126_at	multiple inositol polyphosphate histidine phosphatase 1	Minpp1	2529	1160	NM_019263 /// XM_001079775 /// XM_342044	1q52	2.18	0.000
1370386_at	RuvB-like protein 1	Ruvbl1	2183	1001	NM_147177	4q34	2.18	0.001
1392983_at	proteasome (prosome, macropain) 26S subunit, non-ATPase, 12	Psmd12	4779	2194	NM_001005875	10q32.1	2.18	0.000
1371443_at	similar to RIKEN cDNA A430005L14	RGD1304567	1308	601	NM_001009711	5q36	2.18	0.000

Table S2. Trophoblast stem-associated genes

Affymetrix ID	Gene Title	Gene Symbol	Stem Ave	Dif Ave	GenBank	romosomal Locat	Stem/ Di	t-test
1373454_at	similar to RIKEN cDNA 0610037P05	RGD1305823	2528	1162	NM_001108261 /// XM_001053257 /// XM_340737	10q11	2.18	0.000
1372715_at	sideroflexin 1	Sfxn1	3005	1383	NM_001012213	17p14	2.17	0.000
1398308_at	replication protein A3 (predicted)	Rpa3_predicted	4149	1910	NM_001106584 /// XM_001054662 /// XM_216097	4q21	2.17	0.000
1388397_at	EBNA1 binding protein 2	Ebna1bp2	6415	2956	NM_001008721	5q36	2.17	0.000
1389117_at	O-sialoglycoprotein endopeptidase	Osgep	1132	522	XM_001064067 /// XM_214163	15p14	2.17	0.001
1371296_at	succinate dehydrogenase complex, subunit D, integral membrane protein	Sdhd	2391	1103	NM_198788	8q23	2.17	0.000
1371813_at	HIRA interacting protein 3	Hirip3	1205	556	NM_001025725	1q36	2.17	0.001
1391461_at	similar to hypothetical protein (predicted)	RGD1306576_predicted	1259	581	NM_001106648 /// XM_001068867 /// XM_216375	5q22	2.17	0.000
1398867_at	PRP19/PSO4 pre-mRNA processing factor 19 homolog (S. cerevisiae)	Prpf19	2383	1100	NM_139333	1q43	2.17	0.000
1388683_at	similar to hypothetical protein MGC14151 (predicted)	RGD1559617_predicted	3194	1474	NM_001105794 /// XM_001079364 /// XM_213347	10q24	2.17	0.001
1379542_at	similar to Small nuclear ribonucleoprotein F (snRNP-F) (Sm protein F) (Sm-F) (SmF)	LOC680737 /// LOC687845	7198	3323	XM_001058626 /// XM_001080328	7q13	2.17	0.001
1371421_at	similar to 3-oxoacid CoA transferase 1	LOC678860 /// LOC690109	1248	576	XM_001053611 /// XM_001053666 /// XM_001073299	2q16	2.17	0.012
1367687_a_at	peptidylglycine alpha-amidating monooxygenase	Pam	3620	1672	NM_013000	9q36	2.17	0.005
1367766_at	expressed in non-metastatic cells 2	Nme2	11911	5504	NM_031833	10q26	2.16	0.000
1398944_at	apoptotic chromatin condensation inducer 1	Acin1	1621	749	XM_001054451 /// XM_001065227 /// XM_240178	15p13	2.16	0.019
1378259_at	lamin B receptor	Tardbp_predicted	1817	841	BF396301	5	2.16	0.000
1372645_at	presenilin associated, rhomboid-like	Psarl	2487	1152	NM_001035249 /// XM_001055224 /// XM_001061991 /// XM_001062047 /// XM_001062100	11q23	2.16	0.002
1372693_at	heterogeneous nuclear ribonucleoprotein A1	Hnrpa1	4392	2034	NM_017248	7q36	2.16	0.003
1374390_at	similar to Protein UNQ655/PRO1286 homolog precursor	LOC686324 /// LOC690344	826	383	XM_001073547 /// XM_001074161	1q43	2.16	0.024
1389021_at	similar to HCV NS3-transactivated protein 1 (predicted)	RGD1306332_predicted	3044	1411	NM_001106763 /// XM_001060870 /// XM_216815	9q22	2.16	0.001
1371782_at	nipsnap homolog 3A (C. elegans)	Nipsnap3a	1766	820	NM_001009422 /// XM_001055152 /// XM_001055213	5q24	2.15	0.001
1389200_at	bystin-like	Bysl	2188	1016	NM_182674	---	2.15	0.000
1378020_at	Transcribed locus	---	4701	2184	AI060205	---	2.15	0.002
1372142_at	arsA arsenite transporter, ATP-binding, homolog 1 (bacterial)	Asna1	2962	1377	XM_001066031 /// XM_213848	19q11	2.15	0.007
1395642_at	nucleolar protein 9	Nol9	1952	907	XM_001076353 /// XM_233702	5q36	2.15	0.003
1370421_a_at	cullin-associated and neddylation-dissociated 2 (putative)	Cand2	812	378	NM_181362	4q42	2.15	0.000

Table S2. Trophoblast stem-associated genes

Affymetrix ID	Gene Title	Gene Symbol	Stem Ave	Dif Ave	GenBank	romosomal Locat	Stem/ Di	t-test
1393583_at	similar to RIKEN cDNA 5730466H23 (predicted)	RGD1560957_predicted	1020	474	NM_001109094 /// XM_001058183 /// XM_573669	14q22	2.15	0.000
1376316_at	Origin recognition complex, subunit 6-like (S. cerevisiae)	Orc6l	902	420	NM_001033690	19q11	2.15	0.000
1392702_at	GH regulated TBC protein 1	Musk	917	427	BM387858	5	2.15	0.001
1372488_at	similar to RIKEN cDNA 2310057D15	RGD1309016	1576	734	NM_001009391	14p22	2.15	0.001
1386951_at	NADH dehydrogenase (ubiquinone) 1 alpha subcomplex 5	Ndufa5	1459	680	NM_012985	4q22	2.15	0.000
1367497_at	phosphatidylserine synthase 1	Ptdss1	3148	1469	NM_001012113	7q22	2.14	0.000
1390692_at	cytidine 5'-triphosphate synthase (predicted)	Ctps_predicted	1918	896	XM_001053648 /// XM_233467	5q36	2.14	0.000
1371391_at	thioredoxin domain containing 5 (predicted)	Txndc5_predicted	2320	1084	BM390196	17	2.14	0.021
1371567_at	aldehyde dehydrogenase family 7, member A1	Aldh7a1	3636	1699	XM_001059375 /// XM_214535	18q12.1	2.14	0.000
1371663_at	similar to expressed sequence AW556797 (predicted)	RGD1305138_predicted	2199	1028	XM_235689 /// XR_008462	7q36	2.14	0.002
1374051_at	similar to hypothetical protein D15Ertd785e	MGC114417	1290	604	NM_001024877 /// XM_001055014	7q34	2.14	0.001
1367999_at	aldehyde dehydrogenase 2	Aldh2	4884	2285	NM_032416	12q16	2.14	0.003
1374532_at	prostaglandin E synthase 2 (predicted)	Ptges2_predicted	1384	648	NM_001107832 /// XM_001078154 /// XM_231144	3p11	2.14	0.000
1372793_at	single-stranded DNA binding protein 1	Ssbp1	1561	731	NM_183328	4q22	2.14	0.004
1389582_at	similar to Nucleoporin Nup43	LOC683983	3165	1483	XM_001068349	11	2.13	0.000
1398809_at	nuclear distribution gene E homolog 1 (A nidulans)	Nde1	1485	696	NM_053347	10q11	2.13	0.002
1375197_at	similar to ubiquinol-cytochrome c reductase subunit	LOC686951	3562	1670	XM_001076456	---	2.13	0.000
1370976_at	Ras-GTPase-activating protein SH3-domain binding protein	G3bp	7665	3596	XM_001074757 /// XM_340802	10q22	2.13	0.001
1368204_at	ligase I, DNA, ATP-dependent	Lig1	1660	779	NM_001024268 /// NM_030855	1q21	2.13	0.002
1371387_at	cytochrome c oxidase subunit VIIb	Cox7b	12196	5731	NM_182819	Xq31	2.13	0.003
1371591_at	mitochondrial ribosomal protein S18A	Mrps18a	2363	1111	NM_198756	9q12	2.13	0.001
1367772_at	chloride channel, nucleotide-sensitive, 1A	Clns1a	5443	2558	NM_031719	1q32	2.13	0.000
1389344_at	ubiquitin specific protease 39 (predicted)	Usp39_predicted	1940	913	NM_001106597 /// XM_001063779 /// XM_216173	4q33	2.12	0.000
1391481_at	---	---	6780	3199	BE104424	---	2.12	0.026
1388564_at	similar to hypothetical protein FLJ20436	RGD1303127	3638	1717	NM_001004244	7q36	2.12	0.000
1372401_at	N-acetylneuraminic acid synthase (sialic acid synthase) (predicted)	Nans_predicted	2331	1100	NM_001106655 /// XM_001066033 /// XM_216398	5q22	2.12	0.000
1375579_at	hypothetical protein LOC688300 /// hypothetical protein LOC690871	LOC688300 /// LOC690871	1500	708	XM_001075950 /// XM_001081779	10q32.3	2.12	0.000
1392446_at	kelch-like 2, Mayven (Drosophila) (predicted)	Klhl2_predicted	2191	1034	XM_001073589 /// XM_214331	16p13	2.12	0.001

Table S2. Trophoblast stem-associated genes

Affymetrix ID	Gene Title	Gene Symbol	Stem Ave	Dif Ave	GenBank	romosomal Locat	Stem/ Di	t-test
1385133_at	similar to spermatogenesis associated 5-like 1	LOC691729	887	419	NM_001109647 /// XM_001073008 /// XM_001073038 /// XM_001073066 /// XM_001073099 /// XM_001077237 /// XM_001077255	3q35	2.12	0.008
1375411_at	NADH dehydrogenase (ubiquinone) 1 alpha subcomplex, 7 (B14.5a) (predicted)	Ndufa7_predicted	3057	1445	NM_001106772 /// XM_001079223 /// XM_216859	7q12	2.12	0.003
1389050_at	similar to Dual specificity protein phosphatase 3 (T-DSP11) (predicted)	RGD1560049_predicted	1439	681	XM_001081500 /// XM_573199	10q32.1	2.11	0.000
1372271_at	RNA-binding region (RNP1, RRM) containing 1 (predicted)	Rnpc1_predicted	1525	722	NM_001108965 /// XM_001054287 /// XM_345477	3q42	2.11	0.018
1370393_at	coiled-coil domain containing 5	Ccdc5	1347	638	NM_138864	18q12.3	2.11	0.000
1388911_at	DNA primase, p58 subunit	Prim2	1245	590	NM_001024762	9q21	2.11	0.000
1398458_at	similar to protein kinase, lysine deficient 1; kinase deficient protein (predicted)	RGD1307284_predicted	1661	788	XM_225204 /// XR_007501	17p14	2.11	0.002
1372173_at	similar to U5 snRNP-specific protein (Prp8-binding) (predicted)	RGD1309198_predicted	1978	939	XM_232775 /// XR_008002	5q36	2.11	0.001
1374063_at	splicing factor, arginine/serine-rich 3 (SRp20) (predicted)	Sfrs3_predicted	11543	5478	NM_001047907 /// XM_001069454 /// XM_342107	20p12	2.11	0.001
1389274_at	dephospho-CoA kinase domain containing	Dcakd	1433	680	NM_001007724	10q32.1	2.11	0.002
1373263_at	similar to WD repeat domain 74	LOC686449	3076	1460	XM_001077353	---	2.11	0.000
1382135_at	similar to CG9346-PA (predicted)	RGD1307882_predicted	6733	3201	XM_001064961 /// XM_001065014 /// XM_236501	8q31	2.10	0.000
1373824_at	craniofacial development protein 1	Cfdp1	5223	2485	NM_199378	19q12	2.10	0.000
1385534_at	nerve growth factor receptor (TNFRSF16) associated protein 1	Ngfrap1	998	475	NM_053401	Xq35	2.10	0.001
1398910_at	STIP1 homology and U-Box containing protein 1	Stub1	2687	1279	NM_001025625	10q12	2.10	0.000
1389055_at	peptidylprolyl isomerase E (cyclophilin E)	Ppie	1852	882	NM_001047868 /// XM_001054833 /// XM_216524	5q36	2.10	0.002
1370235_at	diazepam binding inhibitor	Dbi	5833	2778	NM_031853 /// XM_001054001 /// XM_001055495	13q13	2.10	0.000
1369785_at	phosphoribosyl pyrophosphate amidotransferase	Ppat	2979	1419	XM_001076038 /// XM_579602	14p11	2.10	0.000
1385592_at	Bcl6 interacting corepressor (predicted)	Bcor_predicted	5144	2451	XR_009547	Xq13	2.10	0.000
1398943_at	similar to Eso3 protein (predicted)	RGD1562476_predicted	1543	735	NM_001106345 /// XM_001056585 /// XM_215227	Xq37	2.10	0.000
1393035_at	---	---	1650	786	AI502529	---	2.10	0.000
1373869_at	sterol O-acyltransferase 1	Soat1	2982	1422	NM_031118	13q21	2.10	0.000
1379715_at	similar to CG9346-PA (predicted)	RGD1307882_predicted	1809	863	XM_001064961 /// XM_001065014 /// XM_236501	8q31	2.10	0.001

Table S2. Trophoblast stem-associated genes

Affymetrix ID	Gene Title	Gene Symbol	Stem Ave	Dif Ave	GenBank	romosomal Locat	Stem/ Dil	t-test
1389367_at	schwannomin interacting protein 1	Schip1	1430	682	XM_001061765 /// XM_001061820 /// XM_001069584 /// XM_215570	2q31	2.10	0.000
1372324_at	similar to thyroid hormone receptor interactor 3	LOC497975	1780	850	XM_001081104 /// XM_573169	10q26	2.09	0.000
1371896_at	growth arrest and DNA-damage-inducible, gamma interacting protein 1	Gadd45gip1	1900	908	XM_001071308 /// XM_213842	19q11	2.09	0.000
1382316_at	similar to histone cell cycle regulation defective homolog A isoform 1	LOC363849	1605	767	XM_001062924 /// XM_344057	11	2.09	0.018
1392144_at	5'-nucleotidase domain containing 1 (predicted)	Nt5dc1_predicted	920	440	NM_001106393 /// XM_001060836 /// XM_215405	20q12	2.09	0.002
1381130_at	minichromosome maintenance deficient 8 (S. cerevisiae) (predicted)	Mcm8_predicted	817	391	NM_001106514 /// XM_001079873 /// XM_215825	3q36	2.09	0.008
1372999_at	DCN1, defective in cullin neddylation 1, domain containing 5 (S. cerevisiae)	Dcun1d5	3559	1704	NM_001009696	8q11	2.09	0.001
1376100_at	tubulin, beta 6	Tubb6	4216	2019	NM_001025675	18q12.1	2.09	0.000
1386910_a_at	apurinic/apyrimidinic endonuclease 1	Apex1	3501	1677	NM_024148	15	2.09	0.000
1374446_at	TCDD-inducible poly(ADP-ribose) polymerase (predicted)	Tiparp_predicted	903	432	NM_001107679 /// XM_001060648 /// XM_227217	2q31	2.09	0.003
1372116_at	mitochondrial ribosomal protein S2 (predicted)	Mrps2_predicted	4390	2103	NM_001108576 /// XM_001079091 /// XM_342398	3p12	2.09	0.000
1398891_at	Rho GTPase activating protein 5	Mrpl15_predicted	2658	1274	AI103129	5	2.09	0.001
1371342_at	cytochrome c-1 (predicted)	Cyc1_predicted	7425	3561	XM_001059652 /// XM_001072177 /// XM_001072221 /// XM_216944	7q34	2.08	0.001
1387872_at	heterogeneous nuclear ribonucleoprotein A1	Hnrpa1	10677	5123	NM_017248	7q36	2.08	0.003
1378180_at	similar to 6030466N05Rik protein	LOC290741	1239	594	NM_001013882	16q11	2.08	0.001
1371467_at	similar to RIKEN cDNA 0610007P06	LOC293103	3843	1845	NM_001013897	1q32	2.08	0.003
1388870_at	similar to RNA-binding protein Musashi2-S (predicted)	RGD1560397_predicted	3118	1499	XM_001081205 /// XM_340876	10q26	2.08	0.003
1388405_at	cytoskeleton associated protein 5	Ckap5	3140	1509	XM_001068339 /// XM_001068589 /// XM_001068641 /// XM_001068694 /// XM_001068742 /// XM_001068790 /// XM_001068836 /// XM_230282	3q24	2.08	0.001
1371553_at	mitochondrial ribosomal protein L36 (predicted)	Mrpl36_predicted	2116	1018	NM_001108879 /// XM_001061033 /// XM_344560	17p14	2.08	0.004
1374196_at	lanC (bacterial lantibiotic synthetase component C)-like 1	Lancl1	3457	1663	NM_053723	9q32	2.08	0.005
1389010_at	leukotriene A4 hydrolase	Lta4h	2536	1221	NM_001030031 /// XM_001080297	7q13	2.08	0.001
1374323_at	angiopoietin 1	Bccip_predicted	5407	2605	BM389139	1	2.08	0.000
1376401_at	Transcribed locus	---	902	434	AI412569	---	2.08	0.002
1388930_at	transmembrane protein 123	Tmem123	1220	588	NM_001014205	8q11	2.07	0.004

Table S2. Trophoblast stem-associated genes

Affymetrix ID	Gene Title	Gene Symbol	Stem Ave	Dif Ave	GenBank	romosomal Locat	Stem/ Dif	t-test
1376212_at	similar to Zinc finger protein RP-8 CG3260-PA	LOC687670 /// LOC689637	1114	537	NM_001109544 /// XM_001073061 /// XM_001073097 /// XM_001076951 /// XM_001076979	1q21	2.07	0.000
1377735_at	pleckstrin homology domain-containing, family A (phosphoinositide binding specific) member 3	Plekha3	4755	2294	NM_001013077	3q23	2.07	0.020
1372286_at	tetraspanin 6	Tspan6	1650	797	XM_001067945 /// XM_217563	Xq34	2.07	0.000
1383429_at	similar to T-Brain-1	LOC311078	9352	4516	XM_001054967 /// XM_001059730	3q21	2.07	0.002
1372259_at	DEK oncogene (DNA binding)	Dek	4960	2396	NM_001004255	17p13	2.07	0.000
1379234_a_at	similar to cell division cycle 45 homolog (S. cerevisiae)-like	LOC287961	1048	506	NM_001105866 /// XM_001061676 /// XM_213587	11p12	2.07	0.001
1371915_at	poly(rC) binding protein 4 (predicted)	Pcbp4_predicted	1480	715	XM_001071111 /// XM_343468	8q32	2.07	0.001
1394854_at	telomeric repeat binding factor 1	Terf1	918	444	NM_001012464	5q11	2.07	0.003
1368033_at	nucleolar and coiled-body phosphoprotein 1	Nolc1	2227	1077	NM_022869	1q54	2.07	0.023
1383261_at	Transcribed locus	---	894	432	BF560105	---	2.07	0.007
1375417_at	protein kinase C binding protein 1	Prkcbp1	3309	1600	NM_001100838 /// XM_001061666 /// XM_001071923 /// XM_001071994 /// XM_215942	3q42	2.07	0.001
1373174_at	similar to 1110001K21Rik protein	LOC497867	1534	743	XM_001076872 /// XM_573047	10q12	2.07	0.000
1392499_at	phosphoribosylformylglycinamidine synthase (FGAR amidotransferase) (predicted)	Pfas_predicted	2230	1080	NM_001105791 /// XM_001080147 /// XM_213373	10q24	2.07	0.005
1367603_at	triosephosphate isomerase 1	Tpi1	10098	4891	NM_022922	4q42	2.06	0.002
1388328_at	similar to Eukaryotic translation initiation factor 3 subunit 2 (eIF-3 beta) (eIF3 p36) (eIF3i) (TGF-beta receptor-interacting protein 1) (TRIP-1)	LOC682390	6695	3247	XM_001061306	2	2.06	0.001
1369950_at	cyclin-dependent kinase 4	Cdk4	3197	1552	NM_053593	7q22	2.06	0.001
1371649_at	mitochondrial ribosomal protein S24 (predicted)	Mrps24_predicted	2732	1326	NM_001077659 /// XM_001068903 /// XM_573648	14q21	2.06	0.000
1368184_at	proteasome (prosome, macropain) 26S subunit, non-ATPase, 9	Psmd9	1778	864	NM_130430	12q16	2.06	0.001
1371399_at	hypothetical protein LOC681123	LOC681123	7748	3766	XM_001060392	9q12	2.06	0.000
1383618_at	mitochondrial ribosomal protein L38	Mrpl38	1362	662	NM_001009369	10q32.3	2.06	0.006
1385783_at	similar to BH3-only member B protein (predicted)	RGD1559427_predicted	810	394	NM_001109111 /// XM_001059192 /// XM_573911	16q11	2.06	0.001
1373003_at	squamous cell carcinoma antigen recognized by T-cells 3 (predicted)	Sart3_predicted	1992	969	NM_001107156 /// XM_001080462 /// XM_222279	12q16	2.05	0.004
1373135_at	similar to hypothetical protein MGC2744	LOC619440	891	434	NM_001034109	10q32	2.05	0.002
1388634_at	phosphoglucomutase 1	Pgm1	1042	507	NM_017033	5q33	2.05	0.000

Table S2. Trophoblast stem-associated genes

Affymetrix ID	Gene Title	Gene Symbol	Stem Ave	Dif Ave	GenBank	romosomal Locat	Stem/ Di	t-test
1383324_at	membrane protein, palmitoylated 6 (MAGUK p55 subfamily member 6) (predicted)	Mpp6_predicted	1045	508	XM_001056864 /// XM_342682	4q24	2.05	0.000
1379302_at	recombining binding protein suppressor of hairless (Drosophila) (predicted) /// similar to Recombining binding protein suppressor of hairless (J kappa-recombination signal binding protein) (RBP-J kappa)	LOC679028 /// Rbpsuh_predicted	1469	715	NM_001106631 /// XM_001053717 /// XM_001053777 /// XM_001053835 /// XM_001064167 /// XM_232595	5q11	2.05	0.001
1391427_at	similar to RIKEN cDNA 1500031M22 (predicted)	RGD1311752_predicted	2585	1259	NM_001106408 /// XM_001065350 /// XM_215477	2q14	2.05	0.000
1383182_at	serine/arginine-rich protein specific kinase 1	Srpk1	1727	842	NM_001025726	20p12	2.05	0.000
1373640_at	pyrroline-5-carboxylate reductase family, member 2	Pycr2	1103	537	NM_001012208	13q26	2.05	0.000
1396185_at	RNA binding motif protein 17	Rbm17	1889	921	NM_001013058	17q12.3	2.05	0.002
1371737_at	tripartite motif protein 27 (predicted)	Trim27_predicted	8213	4005	NM_001106115 /// XM_001059726 /// XM_214485	17q11	2.05	0.000
1398960_at	chaperonin subunit 6a (zeta) /// similar to chaperonin subunit 6a (zeta)	Cct6a /// LOC303526 /// LOC316484 /// LOC688183	10896	5316	NM_001033684 /// XR_005482 /// XR_005679 /// XR_007966 /// XR_009465	10q32.1 /// 12q13 /	2.05	0.000
1374287_at	paladin	Pald	829	404	XM_001055668 /// XM_001073719	20q11	2.05	0.002
1376985_at	---	---	1087	531	AA866482	X	2.05	0.004
1372711_at	similar to cDNA sequence BC005537	LOC498750	2398	1171	NM_001017510	17p11	2.05	0.007
1391062_at	similar to elongation protein 4 homolog	LOC687694	1033	506	XM_001079792	---	2.04	0.000
1397708_at	hypothetical LOC288978	LOC288978	817	400	XM_001054392 /// XM_213864	13q11	2.04	0.002
1374113_at	bromodomain adjacent to zinc finger domain, 1A (predicted)	Baz1a_predicted	4784	2343	XM_001079067 /// XM_234156	6q23	2.04	0.000
1372939_at	NudC domain containing 2	Nudcd2	1709	837	NM_001009621	10q12	2.04	0.001
1389497_at	SET and MYND domain containing 5 (predicted)	Smyd5_predicted	1038	509	NM_001107870 /// XM_001073891 /// XM_232134	4q34	2.04	0.003
1389389_at	DEAD (Asp-Glu-Ala-Asp) box polypeptide 56	Ddx56	1638	803	NM_001004211	14q21	2.04	0.001
1378220_at	similar to RIKEN cDNA 4930517K11	LOC497860	3669	1800	AI111983	10q11	2.04	0.004
1372815_at	mago-nashi homolog, proliferation-associated (Drosophila) (predicted)	Magoh_predicted	8582	4213	XM_001066689 /// XM_216485	5q35	2.04	0.000
1373222_at	hexosaminidase A	Hexa	1794	881	NM_001004443	8q24	2.04	0.000
1372150_at	ubiquitin specific protease 10	Usp10	3201	1572	NM_001034146	19q12	2.04	0.001
1379853_at	similar to Placental protein 25 homolog (PP25)	LOC685284	959	471	XM_001060588	5q34	2.04	0.000
1372182_at	phosphofructokinase, platelet	Pfkp	13681	6729	NM_206847	17q12.2	2.03	0.000
1388728_at	lysosomal-associated protein transmembrane 4B	Laptm4b	1395	686	NM_001013174	7q22	2.03	0.005
1390141_at	breast cancer 1	Fthfsdc1_predicted	916	451	BF281848	1	2.03	0.007
1369617_at	ubiquitin-conjugating enzyme E2N	Ube2n	844	416	NM_053928 /// XM_001077606	7q13	2.03	0.004

Table S2. Trophoblast stem-associated genes

Affymetrix ID	Gene Title	Gene Symbol	Stem Ave	Dif Ave	GenBank	romosomal Locat	Stem/ Di	t-test
1382419_at	SoxLZ/Sox6 leucine zipper binding protein in testis (predicted)	Solt_predicted	2353	1160	NM_001106407 /// XM_001072494 /// XM_215471	2q13	2.03	0.000
1392530_at	similar to NADH dehydrogenase (ubiquinone) 1, subcomplex unknown, 1	LOC689938	2850	1405	XM_001072602	2q26	2.03	0.001
1388141_at	centrin 3	Cetn3	2157	1064	XM_001061358 /// XM_342168	2q11	2.03	0.000
1379406_at	similar to chromosome 14 open reading frame 21 (predicted)	RGD1308396_predicted	995	491	NM_001106040 /// XM_001061204 /// XM_214175	15p13	2.03	0.003
1398921_at	mitochondrial ribosomal protein L37	Mrpl37	2238	1105	NM_001004235	5q34	2.03	0.004
1372362_at	galactokinase 2	Galk2	938	463	NM_001013919	3q36	2.03	0.000
1369984_at	cytochrome c oxidase, subunit XVII assembly protein homolog (yeast)	Cox17	3377	1668	NM_053540	11q21	2.03	0.001
1371323_at	similar to NADH-ubiquinone oxidoreductase MLRQ subunit (Complex I-MLRQ) (CI-MLRQ)	LOC681024	12638	6241	XM_001059983	4q21	2.02	0.000
1375767_at	similar to common-site lymphoma leukemia guanine nucleotide exchange factor like (5N754) (predicted)	RGD1308089_predicted	916	452	XM_001080822 /// XM_234320	6q24	2.02	0.006
1374620_at	CEA-related cell adhesion molecule 1	Ceacam1	2120	1048	NM_001033860 /// NM_001033861 /// NM_001033862 /// NM_031755	1q21-q22	2.02	0.004
1383068_at	deoxythymidylate kinase (predicted)	Dtymk_predicted	3221	1595	NM_001106925 /// XM_001071259 /// XM_217478	9q36	2.02	0.021
1381969_at	similar to Recombining binding protein suppressor of hairless (J kappa-recombination signal binding protein) (RBP-J kappa)	LOC679028	3246	1609	XM_001053717 /// XM_001053777 /// XM_001053835	---	2.02	0.000
1398974_at	chromosome segregation 1-like (S. cerevisiae) (predicted)	Cse1l_predicted	4472	2219	NM_001108607 /// XM_001072176 /// XM_342581	3q42	2.02	0.000
1372876_at	selenophosphate synthetase 2	Sephs2	2072	1028	NM_001079889 /// XM_001080177 /// XM_219347	1q36	2.02	0.001
1371620_at	similar to px19-like protein	RGD1308082	4354	2162	NM_001009636	17p14	2.01	0.000
1379235_x_at	similar to cell division cycle 45 homolog (S. cerevisiae)-like	LOC287961	1874	931	NM_001105866 /// XM_001061676 /// XM_213587	11p12	2.01	0.000
1393114_at	alpha-2,6-sialyltransferase ST6GalNAc IV	siat7D	922	458	XM_001080040	20	2.01	0.003
1384878_at	N-myristoyltransferase 2	Nmt2	3199	1591	NM_207590	17q12.3	2.01	0.000
1395058_at	WD repeat domain 75	Wdr75	2027	1008	NM_001047889 /// XM_001060763 /// XM_234768	9q22	2.01	0.037
1367495_at	similar to prefoldin 4 (predicted)	RGD1560211_predicted	7938	3949	XR_009562	14q21	2.01	0.000
1371597_at	ring finger protein 187 (predicted)	Rnf187_predicted	2094	1042	XM_001076738 /// XM_340805	10q22	2.01	0.002
1379312_at	peroxisome proliferative activated receptor, gamma, coactivator-related 1 (predicted)	Pprc1_predicted	2958	1472	NM_001106363 /// XM_001065637 /// XM_215259	1q54	2.01	0.000
1372677_at	similar to RIKEN cDNA 1110033C18	RGD1304783	1810	901	NM_001025006	7q11	2.01	0.000

Table S2. Trophoblast stem-associated genes

Affymetrix ID	Gene Title	Gene Symbol	Stem Ave	Dif Ave	GenBank	romosomal Locat	Stem/ Di	t-test
1376572_a_at	supervillin (predicted)	Svil_predicted	1050	523	NM_001108416 /// XM_001056293 /// XM_341540	17q12.1	2.01	0.001
1385711_at	similar to RIKEN cDNA 1500031M22 (predicted)	RGD1311752_predicted	1277	636	NM_001106408 /// XM_001065350 /// XM_215477	2q14	2.01	0.002
1371899_at	protein kinase, interferon inducible double stranded RNA dependent activator	Prkra	1255	626	NM_001024780	3q23	2.00	0.005
1372290_at	RD RNA-binding protein	Rdbp	1504	751	NM_212548	20p12	2.00	0.005
1375230_at	Endogenous retrovirus mRNA, partial sequence	---	5919	2957	AA800192	---	2.00	0.021
1369940_at	transaldolase 1	Taldo1	4314	2156	NM_031811	1q41	2.00	0.001
1393009_at	similar to RIKEN cDNA 2410002F23	RGD1309326	1223	611	NM_001014018	1q22	2.00	0.003
1387737_at	methionine adenosyltransferase II, alpha	Mat2a	1956	978	NM_134351	4q33	2.00	0.014
1383867_at	fizzy/cell division cycle 20 related 1 (Drosophila) (predicted)	Eif5a2_predicted	2042	1022	BE102803	2	2.00	0.002
1393154_at	Transcribed locus	---	2450	1228	BM384240	---	2.00	0.003
1371470_at	Alcohol dehydrogenase 4 (class II), pi polypeptide	Adh4	3046	1527	NM_017270	2q44	1.99	0.000
1383208_at	polymerase (DNA directed), epsilon 3 (p17 subunit)	Pole3	1438	721	NM_001007652	5q24	1.99	0.000
1371581_at	similar to splicing factor, arginine/serine-rich 1 (ASF/SF2)	LOC688114 /// LOC689890	8848	4440	NM_001109552 /// XM_001072402 /// XM_001081187	10q26	1.99	0.000
1372143_at	ubiquitin-conjugating enzyme E2 variant 2	Ube2v2	5153	2588	NM_183052	11q23	1.99	0.001
1373557_at	minichromosome maintenance deficient 4 homolog (S. cerevisiae)	Mcm4	2654	1333	XM_001068436 /// XM_344048	11	1.99	0.000
1373458_at	brain expressed X-linked 4	Bex4	7577	3810	NM_001037554 /// XM_001053534	Xq35	1.99	0.003
1374733_at	symplekin	Sympk	914	460	XM_001075350 /// XM_214843	1q21	1.99	0.009
1371345_at	methyl-CpG binding domain protein 3 (predicted)	Mbd3_predicted	2430	1223	NM_001108735 /// XM_001076468 /// XM_343162	7q11	1.99	0.000
1370159_at	SWI/SNF related, matrix associated, actin dependent regulator of chromatin, subfamily d, member 2	Smarcd2	1406	707	NM_031983	10q32.1	1.99	0.035
1395338_at	leucine-rich PPR-motif containing	Lrpprc	1138	573	NM_001008519	6q12	1.99	0.010
1386946_at	carnitine palmitoyltransferase 1a, liver	Cpt1a	1279	644	NM_031559	1q43	1.99	0.003
1372501_at	splicing factor 3b, subunit 3 (predicted)	Sf3b3_predicted	5281	2660	NM_001106187 /// XM_001077410 /// XM_214697	19q12	1.99	0.001
1398902_at	similar to mKIAA0664 protein (predicted)	RGD1307222_predicted	1717	865	XM_001080615 /// XM_220693	10q24	1.99	0.024
1397740_at	sideroflexin 1	Sfxn1	2193	1105	NM_001012213	17p14	1.98	0.000
1373489_at	similar to Chromatin assembly factor 1 subunit A (CAF-1 subunit A) (Chromatin assembly factor I p150 subunit) (CAF-I 150 kDa subunit) (CAF-Ip150)	LOC363333	1440	726	XM_001061702 /// XM_343671	---	1.98	0.001
1379476_at	nucleoporin 35	Nup35	2747	1385	NM_001004229 /// XM_001068076	3q24	1.98	0.001

Table S2. Trophoblast stem-associated genes

Affymetrix ID	Gene Title	Gene Symbol	Stem Ave	Dif Ave	GenBank	romosomal Locat	Stem/ Di	t-test
1388326_at	NADH dehydrogenase (ubiquinone) Fe-S protein 8 (predicted)	Ndufs8_predicted	2074	1047	NM_001106322 /// XM_001069669 /// XM_215197	1q42	1.98	0.002
1390787_at	phosphoribosylglycinamide formyltransferase	Gart	2650	1339	XM_001068637 /// XM_573258	11q11	1.98	0.005
1372498_at	cytokine induced apoptosis inhibitor 1	Ciapin1	3892	1968	NM_001007689	19p12	1.98	0.001
1379862_at	similar to CG33331-PA	LOC362419	1961	992	NM_001108642 /// XM_001053478 /// XM_001056640	4q42	1.98	0.002
1372902_at	asparagine-linked glycosylation 12 homolog (yeast, alpha-1,6-mannosyltransferase) (predicted)	Alg12_predicted	1061	537	NM_001108104 /// XM_235555	7q34	1.98	0.001
1378112_at	Transcribed locus	---	2282	1155	BI304019	---	1.98	0.022
1387771_a_at	mitogen activated protein kinase 3	Mapk3	936	474	NM_017347 /// XM_001079997	1q36	1.98	0.000
1371869_at	proteasome (prosome, macropain) subunit, alpha type 7	Psma7	10336	5236	NM_001008217	3q43	1.97	0.001
1372336_at	Unknown sequence	---	6543	3315	BE115454	---	1.97	0.000
1376687_at	ubiquitin specific peptdiase 1	Usp1	3735	1893	NM_001015015	5q33	1.97	0.000
1389126_at	coiled-coil-helix-coiled-coil-helix domain containing 1 (predicted)	Chchd1_predicted	4468	2264	NM_001108369 /// XM_001064763 /// XM_341280	15p16	1.97	0.000
1367833_at	peptidase (prosome, macropain) 26S subunit, ATPase 5	Psmc5	4737	2402	NM_031149	10q32.1	1.97	0.000
1372339_at	cell division cycle 26	Cdc26	1362	691	NM_001013240	5q24	1.97	0.000
1372128_at	mitochondrial ribosomal protein S12 (predicted)	Mrps12_predicted	2401	1220	NM_001106239 /// XM_001078443 /// XM_214890	1q21	1.97	0.000
1389518_at	hypothetical protein LOC680080	LOC680080	6874	3492	XM_001055588	5q22	1.97	0.001
1368330_at	apoptosis antagonizing transcription factor	Aatf	1983	1008	NM_053720	10q26	1.97	0.001
1388294_at	succinate dehydrogenase complex, subunit D, integral membrane protein	Sdhd	4718	2399	NM_198788	8q23	1.97	0.000
1388370_at	cyclin I (predicted)	Ccni_predicted	5316	2704	NM_001105998 /// XM_001070498 /// XM_214007	14p22	1.97	0.000
1390305_at	protein kinase C binding protein 1	Prkcbp1	1173	597	NM_001100838 /// XM_001061666 /// XM_001071923 /// XM_001071994 /// XM_215942	3q42	1.97	0.008
1383067_a_at	deoxythymidylate kinase (predicted)	Dtymk_predicted	1636	832	NM_001106925 /// XM_001071259 /// XM_217478	9q36	1.97	0.003
1393058_at	similar to CREBBP/EP300 inhibitor 2	LOC685691 /// LOC687359	2065	1051	XM_001064863 /// XM_001078141	1q21	1.97	0.000
1388402_at	similar to 2410001H17Rik protein (predicted)	RGD1305824_predicted	1276	649	NM_001078647 /// NM_001078648 /// XM_001070736 /// XM_341978	1q42	1.96	0.003
1371698_at	elongation factor Tu GTP binding domain containing 2	Eftud2	3777	1923	XM_001081526 /// XM_213492	10q32.1	1.96	0.000

Table S2. Trophoblast stem-associated genes

Affymetrix ID	Gene Title	Gene Symbol	Stem Ave	Dif Ave	GenBank	romosomal Locat	Stem/ Di	t-test
1371622_at	similar to Dph2l1 protein (predicted) /// similar to candidate tumor suppressor OVCA2 (predicted)	RGD1562694_predicted /// RGD1564623_predicted	2161	1100	NM_001105809 /// NM_001109036 /// XM_001080453 /// XM_001080669 /// XM_213396 /// XM_573140	10q24	1.96	0.000
1375186_at	similar to zinc finger, CSL domain containing 2	LOC680594 /// LOC682119	3316	1689	XM_001057483 /// XM_001057532 /// XM_001058283 /// XM_001058351	16p16	1.96	0.005
1370443_at	deoxyribonuclease II	Dnase2	1421	725	NM_138539	19q11	1.96	0.012
1375859_a_at	Transcribed locus, strongly similar to XP_218473.3 PREDICTED: similar to zinc finger protein 565 [Rattus norvegicus]	---	4976	2539	BM386823	---	1.96	0.001
1377689_at	kinetochore associated 1 (predicted)	Kntc1_predicted	886	452	NM_001107140 /// XM_001074897 /// XM_222157	12q15-q16	1.96	0.000
1389363_at	acireductone dioxygenase 1	Adi1	1577	807	NM_199097	6q16	1.96	0.000
1390168_a_at	zinc finger, CSL-type containing 3 (predicted)	Zcsl3_predicted	1114	570	XM_001079802 /// XM_342483	3q33	1.95	0.004
1372087_at	hypertrophic agonist responsive protein B64	Harpb64	2890	1479	XM_001071149 /// XM_216665	6q16	1.95	0.006
1373658_at	Rac GTPase-activating protein 1 (predicted)	Racgap1_predicted	5590	2860	NM_001108112 /// XM_001062413 /// XM_235650	7q36	1.95	0.000
1384762_at	Transcribed locus	---	1577	807	BE103873	---	1.95	0.003
1392972_at	triple functional domain (PTPRF interacting)	Trio	2139	1096	NM_001107658 /// XM_001058824 /// XM_226888	2q22	1.95	0.007
1368309_at	thioredoxin reductase 2	Txnrd2	802	411	NM_022584	11	1.95	0.002
1368101_at	calmodulin 3	Calm3	1789	917	NM_012518	1q22	1.95	0.001
1369013_a_at	mitochondrial ribosomal protein L17	Mrpl17	6636	3402	NM_133539	1q33	1.95	0.002
1390709_at	triple functional domain (PTPRF interacting)	Trio	2026	1041	NM_001107658 /// XM_001058824 /// XM_226888	2q22	1.95	0.014
1393238_at	similar to DKFZP434B168 protein (predicted)	RGD1308014_predicted	1039	534	XM_001067002 /// XM_001067053 /// XM_223075	13q27	1.95	0.000
1371525_at	solute carrier family 12 (potassium/chloride transporters), member 7	Slc12a7	3091	1588	XM_001060536 /// XM_001071999	1p11	1.95	0.002
1375701_at	similar to Hypothetical UPF0049 protein ZK1128.2 in chromosome III	LOC360568	2897	1488	XM_001080623 /// XM_340844	10q24	1.95	0.001
1398837_at	transcription elongation factor B (SIII), polypeptide 2	Tceb2	11683	6003	NM_031129 /// XM_001056409	10q12	1.95	0.002
1372052_at	budding uninhibited by benzimidazoles 3 homolog (S. cerevisiae)	Bub3	7691	3952	NM_001047906 /// XM_001053400 /// XM_001053470 /// XM_341943	1q41	1.95	0.000
1389551_at	lactamase, beta 2	Lactb2	849	436	NM_001024247	5q11	1.95	0.006
1371658_at	COX4 neighbor	Cox4nb	2269	1167	NM_001012165	19q12	1.94	0.001
1372021_at	nucleoporin 188	Nup188	1028	529	XM_001077334 /// XM_345335	3p12	1.94	0.005
1373273_at	PRP38 pre-mRNA processing factor 38 (yeast) domain containing A (predicted)	Prpf38a_predicted	2171	1117	NM_001106672 /// XM_001060413 /// XM_216475	5q35	1.94	0.001

Table S2. Trophoblast stem-associated genes

Affymetrix ID	Gene Title	Gene Symbol	Stem Ave	Dif Ave	GenBank	romosomal Locat	Stem/ Di	t-test
1367831_at	tumor protein p53	Tp53	1371	706	NM_030989	10q24	1.94	0.004
1391905_at	similar to PWP2 periodic tryptophan protein homolog	LOC687601 /// LOC690297	1258	648	XM_001074000 /// XM_001079336	20p12	1.94	0.006
1372705_at	calcium homeostasis endoplasmic reticulum protein (predicted)	Cherp_predicted	1894	975	NM_001106064 /// XM_001063423 /// XM_001063484 /// XM_001063542 /// XM_001063602 /// XM_214307	16p14	1.94	0.011
1373121_at	similar to Mitochondrial 28S ribosomal protein S28 (S28mt) (MRP-S28)	LOC679330	939	484	XM_001054178 /// XM_001054311	2	1.94	0.001
1373351_at	ankyrin 2, neuronal	Ank2	1313	676	XM_001076082 /// XM_342337	2q42	1.94	0.009
1373500_at	leucine-rich PPR-motif containing	Lrpprc	3504	1805	NM_001008519	6q12	1.94	0.001
1398411_at	---	---	12921	6659	BI278952	---	1.94	0.004
1367820_at	barrier to autointegration factor 1	Banf1	5522	2850	NM_053631	1q43	1.94	0.000
1376250_at	nuclear fragile X mental retardation protein interacting protein 1	Nufip1	3478	1795	NM_001007758	15q11	1.94	0.009
1384256_at	similar to multi sex combs CG12058-PA	LOC686883	4574	2362	NM_001044290 /// XM_001076161	---	1.94	0.000
1386983_at	hydroxymethylbilane synthase	Hmbs	1422	735	NM_013168	8q22	1.93	0.002
1395297_at	similar to hypothetical protein FLJ10652	RGD1309621	1904	985	XM_001075984 /// XM_229579	4q44	1.93	0.000
1389832_at	glutathione S-transferase omega 1	Gsto1	6141	3176	NM_001007602	1q54	1.93	0.002
1374622_at	Transcribed locus	---	3720	1925	BE107309	---	1.93	0.000
1388309_at	high mobility group AT-hook 1	Hmga1	2421	1253	NM_139327	20p12	1.93	0.005
1379521_at	asparagine-linked glycosylation 3 homolog (yeast, alpha-1,3-mannosyltransferase)	Alg3	1203	623	NM_001011897	11q23	1.93	0.000
1368234_at	prolyl endopeptidase	Prep	1366	708	NM_031324	20q13	1.93	0.000
1376201_at	similar to Hypothetical UPF0049 protein ZK1128.2 in chromosome III	LOC360568	1639	850	XM_001080623 /// XM_340844	10q24	1.93	0.000
1389326_at	replication factor C (activator 1) 3	Rfc3	1459	756	NM_001009629	12p12	1.93	0.001
1370313_at	acyl-CoA thioesterase 7	Acot7	4483	2326	NM_013214	5q36	1.93	0.026
1376196_a_at	mediator of RNA polymerase II transcription, subunit 4 homolog (yeast)	Med4	1321	685	NM_001024256	15p11	1.93	0.001
1390650_at	pericentrin 1	Pcnt1	2009	1043	NM_001025401	10q32.3	1.93	0.008
1368588_at	DEAD (Asp-Glu-Ala-Asp) box polypeptide 52	Ddx52	2152	1117	NM_053525	10q26	1.93	0.001
1372181_at	Replication protein A1	Rpa1	3069	1594	NM_001047843 /// XM_001080467 /// XM_213389	10q24	1.92	0.003
1372059_at	similar to RIKEN cDNA 2610528E23	RGD1309437	1389	722	NM_001013866	11q12	1.92	0.000
1370191_at	antizyme inhibitor 1	Azin1	2575	1338	NM_022585	7q22	1.92	0.002
1367613_at	peroxiredoxin 1	Prdx1	20194	10503	NM_057114	5q36	1.92	0.004
1371634_at	similar to RIKEN cDNA 1810020E01	RGD1305677	3422	1785	NM_001011557	1q32	1.92	0.000
1377461_at	hypothetical protein LOC680485	LOC680485	2048	1068	XM_001057391	3q42	1.92	0.012
1398385_at	similar to RIKEN cDNA 1500006O09 (predicted)	RGD1305475_predicted	4435	2314	XM_001069423 /// XM_223828	15p16	1.92	0.006
1390285_at	similar to BC026645 protein (predicted)	RGD1560911_predicted	896	468	XM_001062704 /// XM_219486	1q41	1.92	0.003
1388514_at	protein phosphatase 1G (formerly 2C), magnesium-dependent, gamma isoform	Ppm1g	5843	3050	NM_147209	6q14	1.92	0.000

Table S2. Trophoblast stem-associated genes

Affymetrix ID	Gene Title	Gene Symbol	Stem Ave	Dif Ave	GenBank	romosomal Locat	Stem/ Dif	t-test
1390124_at	similar to hypothetical protein FLJ38426 (predicted)	RGD1564603_predicted	1605	838	XM_001080684 /// XM_575208	3q35	1.91	0.000
1371463_at	PHD finger protein 5A	Phf5a	5158	2695	NM_138888	7q34	1.91	0.000
1389184_at	similar to Ribonuclease P protein subunit p30 (RNaseP protein p30) (RNase P subunit 2)	LOC685332 /// LOC687772	1275	666	XM_001063377 /// XM_001080083	1q53	1.91	0.001
1380794_at		Aqr_predicted	1567	819	BE106342	---	1.91	0.001
1386073_at	Similar to T-Brain-1	LOC311078	1420	743	XM_001054967 /// XM_001059730	3q21	1.91	0.004
1375645_at	similar to DNA segment, Chr 4, ERATO Doi 22, expressed (predicted)	RGD1560286_predicted	1464	766	XM_001072503 /// XM_575942	5q36	1.91	0.003
1371612_at	tRNA splicing endonuclease 34 homolog	TSEN34	7330	3836	NM_001006968	1q12	1.91	0.001
1372519_at	nucleoporin 93	Nup93	2823	1478	NM_001011925	19p12	1.91	0.001
1374897_at	similar to THO complex subunit 4 (Tho4) (RNA and export factor binding protein 1) (REF1-I) (Ally of AML-1 and LEF-1) (Aly/REF)	LOC688305	7160	3748	XM_001081820 /// XM_001081821	10	1.91	0.008
1382923_at	synaptotagmin binding, cytoplasmic RNA interacting protein	Syncrip	3068	1606	NM_001047916 /// XM_001065902 /// XM_343446	8q31	1.91	0.000
1392522_at	similar to hypothetical protein D10Ertd438e (predicted)	RGD1307879_predicted	1765	924	XM_001058252 /// XM_215387	20q11	1.91	0.001
1398730_at	similar to arsenate resistance protein 2	LOC686980	907	475	XM_001076594	3	1.91	0.024
1367670_at	fumarate hydratase 1	Fh1	8998	4716	NM_017005	13q25	1.91	0.001
1388344_at	similar to RIKEN cDNA 1110005A23	RGD1305692	4173	2187	NM_001033070	7q11	1.91	0.005
1367941_at	transcription factor A, mitochondrial	Tfam	3239	1699	NM_031326	20p11	1.91	0.001
1371739_at		Letm1	1251	656	AI104605	14	1.91	0.000
1369948_at	nerve growth factor receptor (TNFRSF16) associated protein 1	Ngfrap1	6189	3246	NM_053401	Xq35	1.91	0.018
1390681_at	Transcribed locus	---	1432	751	AA964074	17	1.91	0.012
1372543_at	similar to RIKEN cDNA 2610029G23 (predicted)	RGD1562502_predicted	2944	1545	NM_001108818 /// XM_001053847 /// XM_343808	Xq31	1.91	0.000
1398439_a_at	origin recognition complex, subunit 6-like (S. cerevisiae)	Orc6l	935	491	NM_001033690	19q11	1.90	0.002
1373383_at	MTERF domain containing 1	Mterfd1	3989	2096	NM_199387	7q22	1.90	0.000
1390390_at	transcription factor 20	Tcf20	2022	1062	XM_001077683 /// XM_345861	7q34	1.90	0.002
1389535_at	death associated protein 3	Dap3	1200	630	NM_001011950	2q34	1.90	0.000
1377060_at	methylcrotonoyl-Coenzyme A carboxylase 2 (beta)	Mccc2	1148	604	NM_001012177	2q12	1.90	0.002
1390048_at	serine/arginine repetitive matrix 2 (predicted)	Srrm2_predicted	4978	2618	XM_001056343 /// XM_220207	10q12	1.90	0.047
1398980_at	suppressor of Ty 16 homolog (S. cerevisiae) (predicted)	Supt16h_predicted	5037	2651	NM_001107261 /// XM_001075690 /// XM_223981	15p14	1.90	0.000
1382521_at	glutaminase	Gls	2936	1545	NM_001109968 /// NM_012569	9q31-q32	1.90	0.007
1373260_at		Lims2_predicted	3377	1777	AI412606	---	1.90	0.001
1379849_at	THO complex 3 (predicted)	Thoc3_predicted	1398	736	NM_001106059 /// XM_001068440 /// XM_237957	17p14	1.90	0.018
1398847_at	nudix (nucleoside diphosphate linked moiety X)-type motif 4	Nudt4	1372	723	NM_053598	7q13	1.90	0.007

Table S2. Trophoblast stem-associated genes

Affymetrix ID	Gene Title	Gene Symbol	Stem Ave	Dif Ave	GenBank	romosomal Locat	Stem/ Di	t-test
1371848_at	similar to small nuclear ribonucleoprotein D3	LOC687711	4557	2404	XM_001079870	---	1.90	0.001
1390489_at	Synaptotagmin binding, cytoplasmic RNA interacting protein	Syncrip	1532	808	NM_001047916 /// XM_001065902 /// XM_343446	8q31	1.90	0.004
1395152_at	RNA binding motif protein 13	Rbm13	1155	610	NM_001014002	16q12.3	1.89	0.001
1376681_at	similar to RIKEN cDNA 1500003O22	RGD1308302	1181	624	NM_001008346	1q33	1.89	0.001
1372732_at	inosine triphosphatase (nucleoside triphosphate pyrophosphatase)	Itpa	3047	1613	NM_001107774 /// XM_001081181 /// XM_230604	3q36	1.89	0.000
1370298_at	similar to hypothetical protein D11Ertd99e (predicted)	RGD1564337_predicted	3764	1993	NM_001109047 /// XM_001081477 /// XM_573196	10q32.1	1.89	0.001
1385066_a_at	Transcribed locus	---	844	447	BM390527	12	1.89	0.002
1372932_at	novel nuclear protein 1	Nnp1	2591	1372	NM_001012073 /// XM_001079295	20p12	1.89	0.001
1372402_at	N-acetylneuraminic acid synthase (sialic acid synthase) (predicted)	Nans_predicted	1726	914	NM_001106655 /// XM_001066033 /// XM_216398	5q22	1.89	0.002
1387850_at	transmembrane protein with EGF-like and two follistatin-like domains 1	Tmeff1	1017	538	NM_023020 /// XM_001055768	5q22	1.89	0.003
1367837_at	proteasome (prosome, macropain) subunit, alpha type 4	Psma4	3600	1907	NM_017281	8q24	1.89	0.000
1371955_at	mitochondrial ribosomal protein L35 (predicted)	Mrpl35_predicted	2042	1082	NM_001106596 /// XM_001063053 /// XM_216169	4q33	1.89	0.005
1370295_at	expressed in non-metastatic cells 1	Nme1	4089	2168	NM_138548	10q26	1.89	0.004
1371830_at	ubiquitin-like 1 (sentrin) activating enzyme E1A	Uble1a	2287	1212	NM_001012063	1q21	1.89	0.002
1383577_at	stratifin (predicted)	Rabl3_predicted	2878	1527	AA859277	---	1.88	0.010
1383945_at	uridine-cytidine kinase 2	Uck2	3077	1633	NM_001102408 /// XM_001076056 /// XM_222855	13q23	1.88	0.001
1377952_at	ADP-dependent glucokinase	Adpgk	923	490	XM_001072870 /// XM_236306	8q24	1.88	0.000
1393388_at	Zinc finger, DHHC domain containing 3	Zdhhc3	978	519	NM_001039014	8q32	1.88	0.002
1370230_at	ATP synthase, H+ transporting, mitochondrial F0 complex, subunit F6	Atp5j	7327	3889	NM_053602	11q11	1.88	0.001
1368079_at	pyruvate dehydrogenase kinase, isoenzyme 1	Pdk1	836	444	NM_053826 /// XM_001059583 /// XM_001059645	3q22	1.88	0.003
1374289_at	RNA polymerase 1-1	Rpo1-1	4883	2592	NM_001008330	9q12	1.88	0.000
1373741_at	pseudouridine synthase 1	Pus1	1143	607	NM_001025563	12q16	1.88	0.017
1383380_at	ribonuclease P/MRP 38 subunit (human)	Rpp38	1873	995	NM_001033063	17q12.3	1.88	0.001
1371914_at	SWI/SNF related, matrix associated, actin dependent regulator of chromatin, subfamily b, member 1	Smarcb1	1320	701	NM_001025728	20p12	1.88	0.003
1388158_at	HLA-B-associated transcript 1A	Bat1a	7855	4175	NM_133300	20p12	1.88	0.001
1372272_at	hypothetical protein LOC692032	LOC692032	828	440	XM_001081273	15q21	1.88	0.010
1398884_at	prefoldin 5 (predicted)	Pfdn5_predicted	3151	1676	NM_001106794 /// XM_001067687 /// XM_217061	7q36	1.88	0.001
1393753_at	NudC domain containing 1 (predicted)	Nudcd1_predicted	874	465	XM_001063815 /// XM_343235	7q31	1.88	0.001

Table S2. Trophoblast stem-associated genes

Affymetrix ID	Gene Title	Gene Symbol	Stem Ave	Dif Ave	GenBank	romosomal Locat	Stem/ Di	t-test
1389844_at	FK506 binding protein 4	Fkbp4	3353	1784	XM_001066628 /// XM_342763	4q42	1.88	0.000
1370931_at	X-ray repair complementing defective repair in Chinese hamster cells 5	Xrcc5	1072	570	NM_177419	9q34	1.88	0.000
1367676_at	high mobility group box 2 /// similar to High mobility group protein 2 (HMG-2) (predicted) /// similar to High mobility group protein 2 (HMG-2)	Hmgb2 /// LOC680704 /// RGD1559962_predicted /// RGD1561694_predicted /// RGD1564519_predicted	14981	7975	NM_017187 /// XM_001054623 /// XM_001058504 /// XM_001060826 /// XM_001063065 /// XM_001074365 /// XM_573272 /// XM_573625 /// XM_573903 /// XM_574279	11q12 /// 14q21 ///	1.88	0.000
1387779_at	MYB binding protein (P160) 1a	Mybbp1a	4385	2334	NM_031668	10	1.88	0.003
1386963_at	thyroid hormone receptor interactor 10	Trip10	1863	993	NM_053920	9q11	1.88	0.005
1370316_at	hsp70-interacting protein	Hspbp1	1520	810	NM_139261	1q12	1.88	0.003
1383986_at	tropomyosin 3, gamma	Tpm3	8620	4602	NM_057208 /// NM_173111	2q34	1.87	0.001
1388576_at	eukaryotic translation initiation factor 3, subunit 9 (eta)	Eif3s9	5576	2977	NM_001031640	12q11	1.87	0.000
1370915_s_at	deoxynucleotidyltransferase, terminal, interacting protein 1	Dnttip1	1588	848	NM_134400 /// XM_001072013 /// XM_579637	3q42	1.87	0.019
1388884_at	similar to RIKEN cDNA 1810022C23	RGD1310224	907	485	NM_001009275	17p12	1.87	0.019
1383962_at	Cd27 binding protein (Hindu God of destruction) (predicted)	Siva_predicted	1805	965	NM_001100982 /// XM_001072396 /// XM_001072975 /// XM_343117	6q32	1.87	0.003
1385109_at	Josephin domain containing 3	Josd3	1873	1003	NM_001014207	8q12	1.87	0.002
1369635_at	sorbitol dehydrogenase	Sord	1345	720	NM_017052	3q35	1.87	0.001
1389065_at	RNA binding motif protein 34	Rbm34	966	517	NM_001014015	19q12	1.87	0.003
1371712_at	similar to snRNP core protein SMX5	LOC684148	3448	1850	XM_001060812 /// XM_001060866	19	1.86	0.000
1367463_at	prohibitin 2	Phb2	9292	4984	NM_001013035	4q42	1.86	0.003
1398316_at	similar to LEYDIG CELL TUMOR 10 KD PROTEIN	LOC288913	1187	638	NM_198728	19q11	1.86	0.000
1376317_at	origin recognition complex, subunit 6-like (S. cerevisiae)	Orc6l	1877	1008	NM_001033690	19q11	1.86	0.000
1367756_at	G elongation factor	Gfm	1091	586	NM_053625	2q31	1.86	0.003
1380185_at	similar to hypothetical protein FLJ22965 (predicted)	RGD1564541_predicted	1101	591	NM_001107950 /// XM_001062744 /// XM_233309	Xq12	1.86	0.000
1377772_at	transmembrane protein with EGF-like and two follistatin-like domains 1	Tmeff1	2545	1368	NM_023020 /// XM_001055768	5q22	1.86	0.001
1367493_at	similar to DNA segment, Chr 18, Wayne State University 98, expressed (predicted)	RGD1560212_predicted	4050	2178	NM_001077676 /// XM_001057945 /// XM_574178	18q12.3	1.86	0.001
1384588_at	similar to 4931433E08Rik protein (predicted)	RGD1565838_predicted	1183	637	NM_001108518 /// XM_001076558 /// XM_342008	1q43	1.86	0.000
1389063_at	exportin 6	Xpo6	3153	1697	XM_001076854 /// XM_574559	1q36	1.86	0.002

Table S2. Trophoblast stem-associated genes

Affymetrix ID	Gene Title	Gene Symbol	Stem Ave	Dif Ave	GenBank	romosomal Locat	Stem/ Dif	t-test
1389338_at	hypothetical LOC293114 (predicted)	RGD1308371_predicted	2033	1094	NM_001106280 /// XM_001062219 /// XM_214995	1q32	1.86	0.000
1376488_at	similar to KIAA1582 protein (predicted)	RGD1310027_predicted	1115	601	XR_006923 /// XR_009495	10q32.3	1.86	0.001
1374632_at	jumonji domain containing 6	Jmjd6	1292	697	NM_001012143	10q32.3	1.85	0.000
1376091_at	adenylosuccinate lyase (predicted)	Adsl_predicted	1756	947	XM_001076341 /// XM_235496	7q34	1.85	0.001
1376005_at	similar to RIKEN cDNA 1190017O12 (predicted)	Kif1b	1112	600	BE109334	5	1.85	0.002
1397551_at	DEAD (Asp-Glu-Ala-Asp) box polypeptide 27	Ddx27	2243	1210	XM_001075340 /// XM_342582	3q42	1.85	0.004
1376986_at	methyltransferase-like 3	Mettl3	934	504	NM_001024794	15p14	1.85	0.002
1388414_at	NADH dehydrogenase (ubiquinone) Fe-S protein 5b, 15kDa (NADH-coenzyme Q reductase)	Ndufs5b	5005	2702	NM_001030052	5q36	1.85	0.001
1369002_at	sterol O-acyltransferase 1	Soat1	3883	2096	NM_031118	13q21	1.85	0.011
1398763_at	translocase of inner mitochondrial membrane 23 homolog (yeast)	Timm23	9569	5168	NM_019352	16p16	1.85	0.001
1390149_at	transforming, acidic coiled-coil containing protein 2	Tacc2	2573	1390	NM_001004415 /// NM_001004418	1q37	1.85	0.006
1379829_at	baculoviral IAP repeat-containing 6 (predicted)	Mycbp_predicted	1557	842	BF418799	5	1.85	0.001
1383434_at	pyrroline-5-carboxylate reductase 1 (predicted)	Pycr1_predicted	1213	656	NM_001105857 /// XM_001081808 /// XM_221200	10q32.3	1.85	0.010
1379665_at	peptidylprolyl isomerase domain and WD repeat containing 1 (predicted)	Ppwd1_predicted	1624	879	NM_001106406 /// XM_001056962 /// XM_001064128 /// XM_001064235 /// XM_215474	2q13	1.85	0.000
1370258_at	basic leucine zipper and W2 domains 2	Bzw2	1357	734	NM_134402	6q16	1.85	0.023
1399098_at	glyoxylase 1	Glo1	5348	2894	NM_207594	20p12	1.85	0.005
1372028_at	similar to Protein CGI-117 (Protein HSPC111) (predicted)	RGD1305727_predicted	3823	2070	NM_001047095 /// XM_001067951 /// XM_225176	17p14	1.85	0.005
1388458_at	replication factor C (activator 1) 4 (predicted)	Rfc4_predicted	3500	1896	NM_001105869 /// XM_001056509 /// XM_213598	11q23	1.85	0.000
1398875_at	polymerase (RNA) III (DNA directed) polypeptide K	Polr3k	3064	1661	NM_001014259	3q43	1.84	0.001
1373046_at	DAZ associated protein 1	Dazap1	2164	1174	NM_001025742	7q11	1.84	0.008
1371352_at	high mobility group nucleosomal binding domain 2	Hmgn2	9747	5287	NM_001025624	5q36	1.84	0.001
1379424_at	similar to DKFZP434B168 protein (predicted)	RGD1308014_predicted	844	458	XM_001067002 /// XM_001067053 /// XM_223075	13q27	1.84	0.024
1399077_at	similar to Metaxin 1, isoform 2	LOC295241	2439	1323	XM_001074374 /// XM_227413	2q34	1.84	0.000
1377998_at	coproporphyrinogen oxidase	Cpox	1820	989	NM_001037095	11q12	1.84	0.006
1371980_at	ATPase family, AAA domain containing 3A	Atad3a	817	444	NM_001034922	5q36	1.84	0.006
1374527_at	enoyl Coenzyme A hydratase domain containing 2 (predicted)	Echdc2_predicted	3618	1970	NM_001106675 /// XM_001059951 /// XM_216479	5q35	1.84	0.001

Table S2. Trophoblast stem-associated genes

Affymetrix ID	Gene Title	Gene Symbol	Stem Ave	Dif Ave	GenBank	romosomal Locat	Stem/ Dif	t-test
1389286_at	glutaredoxin 5 homolog (S. cerevisiae) (predicted)	Glrx5_predicted	2649	1444	NM_001108722 /// XM_001069239 /// XM_343103	6q32	1.84	0.003
1398259_at	nucleoporin 155	Nup155	3676	2004	NM_053952	2q16	1.83	0.001
1372100_at	transmembrane protein 50A (predicted)	Tmem50a_predicted	1259	687	XM_001067840 /// XM_216545	5q36	1.83	0.000
1372073_at	GATA zinc finger domain containing 2A	Gatad2a	2545	1388	NM_001013881	16p14	1.83	0.008
1388797_at	U2 small nuclear ribonucleoprotein auxiliary factor (U2AF) 2	U2af2	4844	2645	XM_001059746 /// XM_001059801 /// XM_001059867 /// XM_001060001 /// XM_001060064 /// XM_001060115 /// XM_001077658	1q12	1.83	0.004
1391014_at	zinc finger, MYND domain containing 19	Zmynd19	956	522	NM_198770	3p13	1.83	0.000
1388701_at	myeloid/lymphoid or mixed lineage-leukemia translocation to 6 homolog (Drosophila) (predicted)	Mllt6_predicted	1441	788	XM_001081378 /// XM_239329	10q31	1.83	0.033
1379417_at	midasin homolog (yeast)	Mdn1	1773	969	AA956668	5q21	1.83	0.017
1393242_at	similar to chromosome 16 open reading frame 33; minus -99 protein (predicted)	RGD1310922_predicted	992	542	XM_001062061 /// XM_213260	10q12	1.83	0.004
1393365_at	chromodomain helicase DNA binding protein 1 (predicted)	Chd1_predicted	990	541	NM_001107465 /// XM_001056703 /// XM_238731	1q12	1.83	0.028
1372218_at	WD repeat domain 12	Wdr12	3267	1786	NM_199410	9q31	1.83	0.000
1398860_at	neural precursor cell expressed, developmentally down-regulated gene 8	Nedd8	12592	6887	NM_138878	15p13	1.83	0.002
1379269_at	CD2-associated protein	Cd2ap	3030	1658	NM_181475	9q12	1.83	0.000
1372347_at	Transcribed locus	---	2149	1176	BI289462	---	1.83	0.002
1376321_at	family with sequence similarity 38, member A (predicted)	Fam38a_predicted	1762	964	NM_001077200	19q12	1.83	0.044
1369978_at	phosphoribosyl pyrophosphate synthetase-associated protein 2	Prpsap2	1468	804	NM_057131	10q23	1.83	0.000
1390035_at	similar to hypothetical protein MGC29875; novel putative protein similar to YIL091C yeast hypothetical 84 kD protein from SGA1-KTR7	LOC305076	1576	864	NM_001013986	13q27	1.82	0.002
1387031_at	endoplasmic reticulum protein 29	Erp29	1045	573	NM_053961	12q16	1.82	0.000
1371437_at	SEC13-like 1 (S. cerevisiae)	Sec13l1	6260	3436	NM_001006978	4q42	1.82	0.003
1384259_at	Rac GTPase-activating protein 1 (predicted)	Racgap1_predicted	1233	677	NM_001108112 /// XM_001062413 /// XM_235650	7q36	1.82	0.001
1394082_at	similar to T-Brain-1	LOC311078	6175	3392	XM_001054967 /// XM_001059730	3q21	1.82	0.000
1370919_at	heterogeneous nuclear ribonucleoprotein M	Hnrpm	3856	2120	NM_001109911 /// NM_053876	7q12	1.82	0.006
1371486_at	U1 small nuclear ribonucleoprotein 1C (predicted) /// similar to U1 small nuclear ribonucleoprotein C (U1 snRNP protein C) (U1C protein) (U1-C)	LOC682020 /// LOC685273 /// Snrp1c_predicted	6284	3458	XM_001059425 /// XM_001063116 /// XM_001078182 /// XM_342101	10q12 /// 20p12	1.82	0.001
1381217_at	similar to CGI-09 protein (predicted)	RGD1308877_predicted	1081	595	NM_001107779 /// XM_001081240 /// XM_230548	3q36	1.82	0.002

Table S2. Trophoblast stem-associated genes

Affymetrix ID	Gene Title	Gene Symbol	Stem Ave	Dif Ave	GenBank	romosomal Locat	Stem/ Di	t-test
1377705_at	---	---	1423	784	BF549971	---	1.82	0.049
1384804_at	DNA methyltransferase 3A	Dnmt3a	1234	680	NM_001003957 /// NM_001003958	6q14	1.81	0.001
1384087_at	---	---	1196	659	AA925049	---	1.81	0.000
1374036_at	minichromosome maintenance deficient 2 mitotin (S. cerevisiae) (predicted)	Mcm2_predicted	2558	1410	NM_001107873 /// XM_001072364 /// XM_232168	4q34	1.81	0.000
1372698_at	similar to CG2662-PA (predicted)	RGD1305094_predicted	814	449	XR_008807	19q11	1.81	0.000
1388478_at	transducin (beta)-like 1 X-linked (predicted)	Tbl1x_predicted	4133	2280	NM_001106964 /// XM_001057805 /// XM_217623	Xq22	1.81	0.005
1388517_at	mitochondrial ribosomal protein L40	Mrpl40	1047	578	NM_001024865 /// XM_001061518	11p12	1.81	0.007
1387019_at	ATP synthase, H+ transporting, mitochondrial F0 complex, subunit e	Atp5i	1777	982	NM_080481	14p22	1.81	0.000
1372260_at	leucine zipper domain protein	MGC116147	806	445	NM_001024864	10q12	1.81	0.022
1373387_at	similar to CG33714-PB, isoform B	LOC688717	4822	2665	NM_001109507 /// XM_001059213 /// XM_001068036	6q31	1.81	0.000
1390419_a_at	tumor suppressor candidate 3	Tusc3	2021	1117	NM_001004212	16q12.1	1.81	0.005
1384330_at	similar to hypothetical protein FLJ20546	LOC303067	1674	925	NM_001013966	10q21	1.81	0.002
1384136_at	similar to mKIAA0704 protein (predicted)	RGD1564287_predicted	1561	863	XM_001055606 /// XM_342683	4q24	1.81	0.000
1374631_at	oligonucleotide/oligosaccharide-binding fold containing 2B	Obfc2b	1186	656	NM_001034939	7q11	1.81	0.003
1388327_at	similar to DNA segment, Chr 10, ERATO Doi 214, expressed (predicted)	RGD1309529_predicted	6720	3725	NM_001106385 /// XM_001073750 /// XM_215378	20p11	1.80	0.001
1367911_at	isocitrate dehydrogenase 3 (NAD+) alpha	Idh3a	1760	976	NM_053638	8q24	1.80	0.007
1371551_at	Tnf receptor associated factor 4 (predicted)	Traf4_predicted	808	448	NM_001107017 /// XM_001080843 /// XM_220640	10q25	1.80	0.003
1376849_at	ubiquitin specific protease 48	Usp48	951	528	NM_198785	5q36	1.80	0.004
1370378_at	ATP synthase, H+ transporting, mitochondrial F1 complex, alpha subunit, isoform 1	Atp5a1	10505	5835	NM_023093	18q12.3	1.80	0.000
1383250_at	UTP14, U3 small nucleolar ribonucleoprotein, homolog A (yeast)	Utp14a	1254	697	NM_001014113	Xq35	1.80	0.002
1399034_at	pecanex homolog (Drosophila)	Pcnx	1138	633	XM_001055794 /// XM_234385	6q24	1.80	0.001
1389101_at	ubiquitin-like 1 (sentrin) activating enzyme E1B	Ccnc	1042	580	BE120340	5	1.80	0.000
1368136_at	thymopoietin	Tmpo	1389	773	NM_012887	7q13	1.80	0.011
1393158_at	similar to 2610528M18Rik protein	LOC363198	932	519	NM_001014215	9q12	1.80	0.003
1387113_at	C-terminal binding protein 2	Ctbp2	3896	2170	NM_053335	1q41	1.80	0.007
1377480_at	similar to RIKEN cDNA D030060M11	RGD1308076	1359	757	NM_001025277	8q23	1.80	0.000
1371367_at	TAR DNA binding protein	Tardbp	6252	3483	NM_001011979	5q36	1.80	0.011
1368049_at	t-complex protein 1	Tcp1	16713	9320	NM_012670	1q12	1.79	0.003
1377619_at	WD repeat domain 36 (predicted)	Wdr36_predicted	1216	678	XM_001060069	18p12	1.79	0.000
1376298_at	UPF3 regulator of nonsense transcripts homolog B (yeast) (predicted)	Upf3b_predicted	902	503	XM_001058104 /// XM_233312	Xq12	1.79	0.002
1398963_at	N-myristoyltransferase 2	Taf10_predicted	2465	1377	BM392226	1	1.79	0.002

Table S2. Trophoblast stem-associated genes

Affymetrix ID	Gene Title	Gene Symbol	Stem Ave	Dif Ave	GenBank	romosomal Locat	Stem/ Di	t-test
1374467_at	TNF receptor-associated protein 1	Trap1	2751	1537	NM_001039001	10q12	1.79	0.002
1376723_a_at	Transcribed locus	---	2268	1268	AW142765	---	1.79	0.007
1378501_at	similar to gem (nuclear organelle) associated protein 5	LOC686616	838	469	XM_001074875	10	1.79	0.030
1389519_at	proteasome (prosome, macropain) 26S subunit, non-ATPase, 8	Psmd8	5155	2884	XM_001075072 /// XM_214888	1q21	1.79	0.004
1398301_at	ribosomal protein L36 /// similar to ribosomal protein L36 (predicted) /// hypothetical LOC364105 /// hypothetical gene supported by NM_022504 (predicted) /// similar to ribosomal protein L36	LOC364105 /// LOC689021 /// RGD1563135_predicted /// RGD1563687_predicted /// RGD1563867_predicted /// Rpl36	1524	853	NM_022504 /// XM_001068869 /// XM_575935 /// XR_005868 /// XR_006544 /// XR_008399 /// XR_008905 /// XR_008955	14p22 /// 14q11 ///	1.79	0.007
1378346_at	Transcribed locus	---	1446	809	AI385237	---	1.79	0.025
1383491_at	interferon stimulated exonuclease gene 20-like 1 (predicted)	Isg20l1_predicted	973	545	NM_001108487 /// XM_001064302 /// XM_341874	1q31	1.79	0.002
1385620_at	heat shock 105kDa/110kDa protein 1	Hsph1	1892	1060	NM_001011901	12p11	1.79	0.000
1382166_at	signal recognition particle receptor, B subunit	Srprb	1182	662	NM_001013252	8q32	1.79	0.001
1375516_at	NADH dehydrogenase (ubiquinone) 1, subcomplex unknown, 2	Ndufc2	4647	2604	NM_001009290	1q32	1.78	0.005
1373319_at	DEAD (Asp-Glu-Ala-Asp) box polypeptide 1	Ddx1	10461	5865	NM_053414	6	1.78	0.001
1369929_at	prosaposin	Psap	12821	7191	NM_013013	20q11	1.78	0.000
1377594_at	Src homology 2 domain-containing transforming protein C1	Shc1	2924	1641	NM_053517	2q34	1.78	0.001
1367480_at	similar to DEAD (Asp-Glu-Ala-Asp) box polypeptide 48	rCG_33565	7844	4406	NM_001100158 /// XM_001081786	---	1.78	0.000
1383961_a_at	DnaJ (Hsp40) homolog, subfamily C, member 11 (predicted)	Dnajc11_predicted	1073	603	NM_001108694 /// XM_001076236 /// XM_342983	5q36	1.78	0.010
1372207_at	bromodomain containing 8	Brd8	1191	669	NM_001008509	18p12	1.78	0.002
1377137_at	tyrosyl-DNA phosphodiesterase 1	Tdp1	800	450	NM_001031657	6q32	1.78	0.016
1375641_at	actin related protein 2/3 complex, subunit 5-like /// similar to actin related protein 2/3 complex, subunit 5-like (predicted)	Arpc5l /// RGD1560362_predicted	3800	2139	NM_001037767 /// XM_001059654 /// XM_576552	3q11 /// 9q22	1.78	0.000
1387790_at	phosphoribosylaminoimidazole carboxylase, phosphoribosylaminoimidazole succinocarboxamide synthetase	Paics	10439	5876	NM_080910	14p11	1.78	0.007
1368083_at	cyclin H	Ccnh	3505	1973	NM_052981	2	1.78	0.037
1388199_at	tumor-associated calcium signal transducer 1	Tacstd1	3655	2058	NM_138541	6q12	1.78	0.016
1387077_at	cAMP-regulated phosphoprotein 19	Arpp19	3272	1843	NM_031660	8q24	1.78	0.001
1388364_at	NADH dehydrogenase (ubiquinone) Fe-S protein 3 (predicted)	Ndufs3_predicted	3928	2213	NM_001106489 /// XM_001077876 /// XM_215776	3q24	1.78	0.000
1370062_at	HIG1 domain family, member 1A	Higd1a	2949	1662	NM_080902	8q32	1.77	0.008
1398784_at	complement component 1, q subcomponent binding protein	C1qbp	13939	7856	NM_019259	10q24	1.77	0.001
1388837_at	Solute carrier family 44, member 2 (predicted)	Slc44a2_predicted	1030	581	XM_001077428 /// XM_343355	8q13	1.77	0.031
1371971_at	---	---	2768	1560	AI599676	18	1.77	0.002

Table S2. Trophoblast stem-associated genes

Affymetrix ID	Gene Title	Gene Symbol	Stem Ave	Dif Ave	GenBank	romosomal Locat	Stem/ Di	t-test
1392937_at	cyclin I (predicted)	Ccni_predicted	1182	666	NM_001105998 /// XM_001070498 /// XM_214007	14p22	1.77	0.006
1368233_at	general transcription factor IIF, polypeptide 2	Gtf2f2	1462	825	NM_031042	15q11	1.77	0.005
1377577_at	guanine monphosphate synthetase	Gmps	3867	2184	NM_001024754	2q31	1.77	0.000
1378357_at	Transcribed locus	---	1521	859	AI556522	---	1.77	0.003
1373568_at	Polycomb group ring finger 4 (predicted)	Pcgf4_predicted	1878	1061	NM_001107368 /// XM_001073733 /// XM_225606	17q12.3	1.77	0.001
1367557_s_at	glyceraldehyde-3-phosphate dehydrogenase /// similar to glyceraldehyde-3-phosphate dehydrogenase /// similar to glyceraldehyde-3-phosphate dehydrogenase (predicted)	Gapdh /// LOC366623 /// LOC682005 /// LOC685186 /// LOC686275 /// LOC689689 /// RGD1564688_predicted /// RGD1565368_predicted	29358	16591	NM_017008 /// XM_001059327 /// XM_001062726 /// XM_001065101 /// XM_001070609 /// XM_001070653 /// XM_001073242 /// XM_001073321 /// XM_573304 /// XM_576394 /// XR_006294 /// XR_009270	11q22 /// 16p13 ///	1.77	0.008
1392979_at	calcyclin binding protein	Cacybp	4801	2716	NM_001004208	13q22	1.77	0.017
1367808_at	translocase of inner mitochondrial membrane 8 homolog b (yeast)	Timm8b	5729	3242	NM_022541	8q23	1.77	0.010
1374712_at	proteasome (prosome, macropain) 26S subunit, non-ATPase, 11 (predicted)	Psmd11_predicted	7964	4506	NM_001107027 /// XM_001080984 /// XM_220754	10q26	1.77	0.007
1373609_at	similar to Mitochondrial 28S ribosomal protein S21 (S21mt) (MRP-S21)	LOC689432	2737	1549	XM_001057820 /// XM_001057872 /// XM_001068942 /// XM_001068993	2q34	1.77	0.001
1377827_at	serum response factor binding protein 1	Srfbp1	2158	1222	NM_001005536	18q11	1.77	0.000
1392425_x_at	LUC7-like (S. cerevisiae)	Luc7l	1020	577	NM_001024269	10q12	1.77	0.001
1388898_at	heat shock 105kDa/110kDa protein 1	Hsph1	4392	2491	NM_001011901	12p11	1.76	0.000
1371785_at	tumor necrosis factor receptor superfamily, member 12a	Tnfrsf12a	2101	1192	NM_181086	10q12	1.76	0.000
1388132_at	splicing factor proline/glutamine rich (polypyrimidine tract binding protein associated)	Sfpq	3709	2104	NM_001025271 /// XM_001058863	5q36	1.76	0.009
1382093_at	Similar to DKFZP434B168 protein (predicted)	RGD1308014_predicted	803	456	XM_001067002 /// XM_001067053 /// XM_223075	13q27	1.76	0.004
1388742_at	---	---	984	559	AA945877	---	1.76	0.004
1390178_at	signal recognition particle receptor, B subunit	Srprb	2254	1280	NM_001013252	8q32	1.76	0.000
1379043_at	transmembrane protein 8 (five membrane-spanning domains) (predicted)	Grtp1	1335	758	BI284837	---	1.76	0.001
1373649_at	similar to COX10 homolog, cytochrome c oxidase assembly protein, heme A: farnesyltransferase	LOC687381 /// LOC691853	933	530	XM_001077717 /// XM_001079869	10q24	1.76	0.000
1388695_at	serine hydroxymethyltransferase 2 (mitochondrial)	Shmt2	5493	3122	NM_001008322	7q22	1.76	0.010
1367467_at	similar to nitrogen fixation cluster-like (predicted)	RGD1309562_predicted	2767	1573	NM_001105936 /// XM_001078081 /// XM_213811	12q16	1.76	0.002

Table S2. Trophoblast stem-associated genes

Affymetrix ID	Gene Title	Gene Symbol	Stem Ave	Dif Ave	GenBank	romosomal Locat	Stem/ Di	t-test
1374299_at	DEAH (Asp-Glu-Ala-His) box polypeptide 9 (predicted)	Dhx9_predicted	2862	1627	NM_001107184 /// XM_001066523 /// XM_239780	13q21	1.76	0.008
1371371_at	NADH dehydrogenase (ubiquinone) 1 beta subcomplex, 4, 15kDa	Ndufb4	4663	2652	NM_001037338 /// XM_001056198	11q21	1.76	0.002
1368835_at	signal transducer and activator of transcription 1	Stat1	908	516	NM_001034164 /// NM_032612	9q31-q32	1.76	0.010
1371348_at	proteasome (prosome, macropain) subunit, beta type 5	Psmb5	9441	5370	NM_001105727 /// XM_001054724 /// XM_341314	15p13	1.76	0.006
1367695_at	quinoid dihydropteridine reductase	Qdpr	2291	1304	NM_022390	14q21	1.76	0.003
1371498_at	JTV1	MGC125271	4333	2466	NM_001037348 /// XM_001071883	12p11	1.76	0.000
1385458_a_at	similar to C11orf17 protein (predicted)	RGD1306959_predicted	1191	678	NM_001108497 /// XM_001073151 /// XM_341902	1q33	1.76	0.000
1371395_at	chromobox homolog 3 (HP1 gamma homolog, Drosophila)	Cbx3	9180	5227	NM_001008313	4q24	1.76	0.000
1383466_at	Transcribed locus	---	1042	593	BG667930	3	1.76	0.020
1372596_at	similar to RIKEN cDNA 1110007L15 (predicted)	RGD1311660_predicted	1545	880	XM_001074468 /// XM_213726	12q11	1.76	0.004
1388355_at	RNA binding motif protein 17	Rbm17	7694	4384	NM_001013058	17q12.3	1.75	0.000
1376816_at	mitochondrial ribosomal protein S28	Brca1	865	493	BF284903	10	1.75	0.004
1369971_a_at	heterogeneous nuclear ribonucleoprotein D	Hnrpd	5627	3208	NM_001082539 /// NM_001082540 /// NM_001082541 /// NM_024404	14p22	1.75	0.001
1371478_at	similar to RIKEN cDNA 1110008F13	RGD1307752	8671	4943	NM_001013922	3q42	1.75	0.001
1373850_at	sphingomyelin phosphodiesterase, acid-like 3B	Smpdl3b	2601	1483	NM_001025737	5q36	1.75	0.004
1375546_at	cyclin-dependent kinase inhibitor 3 (predicted)	Fzr1_predicted	1162	663	AI009616	7	1.75	0.006
1388495_at	Transcribed locus	---	1075	613	BI303583	---	1.75	0.037
1373445_at	nucleolar protein 8 (predicted)	Nol8_predicted	1389	793	NM_001108408 /// XM_001056049 /// XM_341503	17p14	1.75	0.003
1372261_at	Transcribed locus	---	1149	657	AI409067	---	1.75	0.001
1388640_at	similar to Ubiquitin-like protein SMT3A precursor (Ubiquitin-related protein SUMO-2)	LOC499417	7946	4543	NM_001024295	20p12	1.75	0.000
1398756_at	nucleophosmin 1 /// similar to Nucleophosmin (NPM) (Nucleolar phosphoprotein B23) (Numatrin) (Nucleolar protein NO38)	LOC300303 /// Npm1	11610	6637	NM_012992 /// XM_001057198 /// XM_213108	10q12 /// Xq14	1.75	0.002
1372550_at		Kpnb1	5979	3422	AI169278	10	1.75	0.000
1389252_at	Transcribed locus	---	1427	817	AI102190	---	1.75	0.034
1371037_at	protein S (alpha)	Pros1	5533	3168	NM_031086	11q21	1.75	0.006
1374976_a_at	Sterol O-acyltransferase 1	Soat1	7519	4306	NM_031118	13q21	1.75	0.006
1387860_at	calpain 2	Capn2	5477	3137	NM_017116	13q26	1.75	0.000
1389387_at	similar to Proteasome inhibitor PI31 subunit	LOC682071 /// LOC689852	2072	1186	NM_001101005 /// XM_001058229 /// XM_001058291 /// XR_006739	3q41	1.75	0.001
1390558_at	staufen, RNA binding protein, homolog 2 (Drosophila)	Stau2	1409	807	NM_001007149 /// NM_001007150 /// NM_134466	5q11	1.75	0.011

Table S2. Trophoblast stem-associated genes

Affymetrix ID	Gene Title	Gene Symbol	Stem Ave	Dif Ave	GenBank	romosomal Locat	Stem/ Dif	t-test
1371638_at	ring finger protein 7 (predicted)	Rnf7_predicted	4222	2421	NM_001106848 /// XM_001065291 /// XM_217235	8q31	1.74	0.005
1370340_x_at	tropomyosin 3, gamma	Tpm3	7023	4027	NM_057208 /// NM_173111	2q34	1.74	0.011
1371845_at	processing of precursor 4, ribonuclease P/MRP family, (S. cerevisiae)	Pop4	1590	912	NM_001009642	1q22	1.74	0.002
1367933_at	S-adenosylmethionine decarboxylase 1	Amd1	2331	1341	NM_031011	20q12	1.74	0.007
1368507_at	proteasome (prosome, macropain) subunit, alpha type 3 /// proteasome subunit alpha type 3-like	Psma3 /// Psma3l	13252	7626	NM_001004094 /// NM_017280	10q25 /// 6q24	1.74	0.028
1383107_at	small nuclear ribonucleoprotein D1 (predicted)	Snrpd1_predicted	3122	1797	NM_001106163 /// XM_001060646 /// XM_214621	18p13	1.74	0.001
1371398_at	similar to ATP synthase, H+ transporting, mitochondrial F0 complex, subunit f, isoform 2	LOC684567 /// LOC690441	9891	5694	XM_001071017 /// XM_001074466	12p11	1.74	0.000
1374945_at	GCD14/PCMT domain containing protein RGD1359191	RGD1359191	1273	734	NM_001007706	6q32	1.73	0.000
1384913_at	Transcribed locus	---	1293	746	AI030882	---	1.73	0.002
1371923_at	acyltransferase like 2 (predicted)	Aytl2_predicted	1515	874	XM_001057012 /// XM_001057066 /// XM_341747	1p11	1.73	0.010
1388794_at	RNA binding motif protein, X chromosome retrogene (predicted)	Rbmxrt_predicted	4676	2700	NM_001107420 /// XM_001071004 /// XM_226369	19q11	1.73	0.000
1375368_at	similar to RIKEN cDNA 1700012G19 gene (predicted)	RGD1307773_predicted	1809	1045	XM_001055264 /// XM_213235	10q12	1.73	0.003
1376648_at	v-myc myelocytomatosis viral related oncogene, neuroblastoma derived (avian)	Mycn	4165	2406	NM_001013096	6q14	1.73	0.002
1368195_at	Hspb associated protein 1	Hspbap1	2313	1336	NM_134419	11q22	1.73	0.002
1370365_at	glutathione synthetase	Gss	1296	750	NM_012962	3q42	1.73	0.003
1388913_at	phosphatidic acid phosphatase type 2c	Ppap2c	2396	1387	NM_139252	7q11	1.73	0.000
1371546_at	similar to TR4 orphan receptor associated protein TRA16	LOC361128	1575	912	NM_001047104	16p14	1.73	0.000
1373169_at	1-acylglycerol-3-phosphate O-acyltransferase 5 (lysophosphatidic acid acyltransferase, epsilon) (predicted)	Agpat5_predicted	1498	867	XM_001074443 /// XM_224993	16q12.5	1.73	0.011
1373620_at	similar to hypothetical protein D10Ertd438e (predicted)	RGD1307879_predicted	3214	1862	XM_001058252 /// XM_215387	20q11	1.73	0.000
1376598_at	PAX interacting (with transcription-activation domain) protein 1 (predicted)	Paxip1_predicted	919	533	NM_001107844 /// XM_001058453 /// XM_231271	4q11	1.73	0.004
1373758_at	flavin adenine dinucleotide synthetase	Flad1	929	538	NM_001110138	---	1.73	0.008
1383643_at	similar to UPF0197 protein C11orf10 homolog (predicted)	RGD1560328_predicted	3436	1993	XM_001074464 /// XM_574622	1q43	1.72	0.001
1373768_at	BMS1-like, ribosome assembly protein (yeast)	Bms1l	1950	1132	XM_001057343 /// XM_342746	4q42	1.72	0.001
1371576_at	mitochondrial ribosomal protein S36 (predicted)	Mrps36_predicted	2690	1563	XM_001071159 /// XM_215468	2q12	1.72	0.001
1373583_at	Transcribed locus	---	1989	1155	BF396317	---	1.72	0.005

Table S2. Trophoblast stem-associated genes

Affymetrix ID	Gene Title	Gene Symbol	Stem Ave	Dif Ave	GenBank	romosomal Locat	Stem/ Dil	t-test
1388136_at	translocase of inner mitochondrial membrane 9 homolog (yeast)	Timm9	2418	1406	XM_001072892 /// XM_001080501	6q24	1.72	0.000
1388489_at	similar to NADH-ubiquinone oxidoreductase B9 subunit (Complex I-B9) (CI-B9)	LOC683547 /// LOC691001	2561	1490	XM_001066473 /// XM_001076462	1q12	1.72	0.008
1399002_at	mitochondrial ribosomal protein S17 (predicted)	Mrps17_predicted	4972	2894	NM_001105923 /// XM_001078418 /// XM_213762	12q13	1.72	0.022
1375967_a_at	dual specificity phosphatase 22 (predicted)	Dusp22_predicted	1275	742	NM_001108412 /// XM_001061713 /// XM_341523	17p12	1.72	0.021
1367709_at	CD63 antigen	Cd63	12715	7409	NM_017125	7q11	1.72	0.000
1371321_at	similar to Cytochrome c oxidase polypeptide VIb (Cytochrome c oxidase subunit AED) (predicted)	RGD1565270_predicted	8197	4777	XM_001071713 /// XM_578078	2q34	1.72	0.002
1389343_at	similar to Hypothetical UPF0327 protein (predicted)	RGD1560187_predicted	7498	4372	XM_001071412 /// XM_342957	5q36	1.72	0.005
1380373_at	guanine monphosphate synthetase	Gmps	2048	1194	NM_001024754	2q31	1.71	0.003
1388555_at	thioredoxin-like 5 (predicted)	Txnl5_predicted	4886	2851	NM_001105805 /// XM_001080157 /// XM_213382	10q24	1.71	0.000
1398432_at	similar to Ankyrin repeat domain protein 28 (predicted)	RGD1559931_predicted	1704	995	XM_001057585 /// XM_001057687 /// XM_001057751 /// XM_224620	16p16	1.71	0.002
1377967_at	retroviral integration site 2 (predicted)	Ris2_predicted	3711	2167	NM_001106192 /// XM_001079374 /// XM_226545	19q12	1.71	0.000
1388362_at	NADH dehydrogenase (ubiquinone) 1 alpha subcomplex, 13 (predicted)	Ndufa13_predicted	4555	2662	XR_008769	16p14	1.71	0.000
1383502_at	similar to mKIAA0259 protein (predicted)	RGD1562949_predicted	1585	927	XM_001072207 /// XM_236578	8q32	1.71	0.000
1392131_at	---	---	834	488	AI012870	3	1.71	0.000
1387805_at	BCL2/adenovirus E1B 19 kDa-interacting protein 3	Bnip3	1500	877	NM_053420	1q41	1.71	0.002
1374886_at	BCS1-like (yeast)	Bcs1l	1203	704	NM_001007666	9q33	1.71	0.006
1371641_at	chaperonin subunit 7 (eta) (predicted)	Cct7_predicted	10540	6170	NM_001106603 /// XM_001073942 /// XM_216180	4q34	1.71	0.000
1388601_at	activator of basal transcription 1	Abt1	2196	1286	NM_001025674 /// XM_001072491 /// XM_225368	17q11	1.71	0.011
1392938_s_at	similar to C11orf17 protein (predicted)	RGD1306959_predicted	1661	972	NM_001108497 /// XM_001073151 /// XM_341902	1q33	1.71	0.001
1372364_a_at	similar to N-terminal asparagine amidohydrolase	RGD1305160	2524	1478	NM_001025124	10q11	1.71	0.001
1375554_at	DEAD (Asp-Glu-Ala-Asp) box polypeptide 47	Ddx47	2677	1568	NM_001015005	4q43	1.71	0.003
1388716_at	euchromatic histone lysine N-methyltransferase 2	Ehmt2	1915	1123	NM_212463	20p12	1.71	0.033
1388931_at	mitochondrial ribosomal protein L13	Mrpl13	2622	1537	NM_001006985	7q32	1.71	0.000
1371617_at	proteasome (prosome, macropain) 26S subunit, non-ATPase, 13 (predicted)	Psmd13_predicted	2183	1280	NM_001108925 /// XM_001058704 /// XM_344976	1q41	1.71	0.001
1367506_at	mitochondrial ribosomal protein L11	mrpl11	2118	1242	NM_001006973	1q43	1.71	0.005

Table S2. Trophoblast stem-associated genes

Affymetrix ID	Gene Title	Gene Symbol	Stem Ave	Dif Ave	GenBank	romosomal Locat	Stem/ Di	t-test
1373941_at	similar to YEATS domain containing 2 (predicted)	RGD1566176_predicted	1286	755	NM_001109057 /// XM_001060129 /// XM_573318	11q23	1.70	0.001
1371239_s_at	tropomyosin 3, gamma	Tpm3	7750	4549	NM_057208 /// NM_173111	2q34	1.70	0.037
1373182_at	claudin 12	Cldn12	1086	637	NM_001100813 /// XM_001067932 /// XM_575355	4q12	1.70	0.001
1372466_at		Tgfbr2	966	568	AI408571	8	1.70	0.033
1367671_at	proliferating cell nuclear antigen	Pcna	16664	9793	NM_022381	3q36	1.70	0.003
1387055_at	amyloid beta precursor protein binding protein 1	Appbp1	2045	1202	NM_032072	19p14	1.70	0.001
1374793_at	WD repeat domain 3 (predicted)	Wdr3_predicted	1943	1144	NM_001107705 /// XM_001065786 /// XM_227527	2q34	1.70	0.021
1369946_at	PDRP protein	Pdrp	948	558	NM_024384	10q12	1.70	0.007
1367471_at	similar to DNA-directed RNA polymerase II 23 kDa polypeptide (RPB25) (RPB5) (RPABC1)	LOC687055 /// LOC690966	2346	1382	NM_001109614 /// XM_001076333 /// XM_001076884	7q11	1.70	0.002
1382287_at	ribonucleic acid binding protein S1	Rnps1	3864	2276	NM_001011890	10q12	1.70	0.003
1398890_at	similar to 2310044H10Rik protein	MGC93975	943	555	NM_001004221	1q22	1.70	0.010
1395595_at	Mki67 (FHA domain) interacting nucleolar phosphoprotein	Mki67ip	3784	2230	NM_139186	13q11	1.70	0.005
1371451_at	ribonuclease H2, large subunit	Rnaseh2a	1320	778	NM_001013234	19q11	1.70	0.001
1390788_a_at	WD repeat domain 36 (predicted)	Wdr36_predicted	2722	1605	XM_001060069	18p12	1.70	0.001
1373731_at	similar to phosphoseryl-tRNA kinase (predicted)	RGD1564300_predicted	806	476	XM_001053680	1q41	1.69	0.000
1370304_at	translocator of inner mitochondrial membrane 17a	Timm17a	8435	4981	NM_019351	13q13	1.69	0.003
1390717_at	similar to chromosome 20 open reading frame 155	RGD1311037	1790	1058	NM_001014258	3q36	1.69	0.000
1372214_at	mitochondrial ribosomal protein S33 (predicted)	Mrps33_predicted	2525	1493	NM_001047863 /// XM_001070269 /// XM_216135	4q22	1.69	0.001
1388645_at	similar to RIKEN cDNA 2810409H07	RGD1307982	13361	7904	NM_001033927	3q22	1.69	0.005
1391577_at	phosphoglycerate mutase family member 5	Pgam5	2597	1537	NM_001025272	12q16	1.69	0.003
1375460_at	similar to hypothetical protein FLJ30656 (predicted)	RGD1309441_predicted	909	538	NM_001105843 /// XM_001081506 /// XM_213467	10q32.1	1.69	0.022
1372312_at	LTV1 homolog (S. cerevisiae)	Ltv1	1745	1034	NM_001014157	1p13	1.69	0.000
1367819_at	glutamate oxaloacetate transaminase 2, mitochondrial	Got2	10108	5986	NM_013177	19p13	1.69	0.000
1372721_at	---	---	4660	2763	AI175447	---	1.69	0.002
1373103_at	metastasis-associated gene family, member 2	Mta2	1292	766	XM_001077908 /// XM_342015	1q43	1.69	0.020
1398772_at	NSFL1 (p97) cofactor (p47)	Nsfl1c	5656	3355	NM_031981	3q41	1.69	0.001
1380030_at	zinc finger protein 593 (predicted)	Znf593_predicted	914	543	NM_001106689 /// XM_001067913 /// XM_216542	5q36	1.68	0.014
1371428_at	similar to Protein transport protein SEC61 gamma subunit	LOC689134	12115	7194	XM_001069684	14q22	1.68	0.003
1377021_at	similar to CGI-09 protein (predicted)	RGD1308877_predicted	1106	657	NM_001107779 /// XM_001081240 /// XM_230548	3q36	1.68	0.002

Table S2. Trophoblast stem-associated genes

Affymetrix ID	Gene Title	Gene Symbol	Stem Ave	Dif Ave	GenBank	romosomal Locat	Stem/ Dif	t-test
1372506_at	proteaseome (prosome, macropain) 28 subunit, 3	Psme3	6151	3658	NM_001011894	10q32.1	1.68	0.009
1392629_a_at	zinc finger CCHC-type and RNA binding motif 1	Zcrb1	1161	690	NM_001034940	7q35	1.68	0.002
1374443_at	immature colon carcinoma transcript 1 (predicted)	Ict1_predicted	1898	1129	XM_001081688 /// XM_221110	10q32.3	1.68	0.001
1388736_at	coiled-coil domain containing 43	Ccdc43	1017	605	XM_001081522 /// XM_340915	10q32.1	1.68	0.001
1382782_at	mitochondrial ribosomal protein S2 (predicted)	Mrps2_predicted	870	517	NM_001108576 /// XM_001079091 /// XM_342398	3p12	1.68	0.002
1383175_a_at	similar to C11orf17 protein (predicted)	RGD1306959_predicted	1025	610	NM_001108497 /// XM_001073151 /// XM_341902	1q33	1.68	0.012
1367795_at	interferon-related developmental regulator 1	Ifrd1	4735	2820	NM_019242	6q16	1.68	0.007
1384295_at	heat shock 70kDa protein 14	Hspa14	4370	2602	NM_001004257	17q12.3	1.68	0.000
1372642_at	Transcribed locus	---	3545	2111	BE113397	---	1.68	0.000
1377506_at	longevity assurance homolog 1 (S. cerevisiae) (predicted) /// growth differentiation factor 1 (predicted)	Gdf1_predicted /// Lass1_predicted	2750	1638	NM_001044230 /// NM_001044240 /// XM_001071020 /// XM_224733	16p14	1.68	0.006
1367578_at	peroxiredoxin 2	Prdx2	13440	8008	NM_017169	19q11	1.68	0.002
1372085_at	ADP-ribosylation factor-like 6 interacting protein 2	Arl6ip2	3330	1985	XM_001063138 /// XM_216629	6q11	1.68	0.007
1372460_at	SET translocation (predicted) /// similar to SET protein (Phosphatase 2A inhibitor I2PP2A) (I-2PP2A) (Template-activating factor I) (TAF-I) (Liver regeneration-related protein LRRGR00002)	LOC502130 /// LOC681893 /// Set_predicted	10867	6481	NM_001012504 /// NM_001110495 /// XM_001058855 /// XR_005945 /// XR_007741	17q11 /// 19q12	1.68	0.000
1382114_at	tousled-like kinase 1 (predicted)	Tlk1_predicted	1673	998	NM_001107734 /// XM_001059845 /// XM_242032	3q21	1.68	0.012
1377889_at	similar to Hypothetical protein KIAA0539 (predicted)	RGD1304621_predicted	1129	674	XM_001073237 /// XM_221669	11q11	1.68	0.007
1372827_at	peptidylprolyl isomerase D (cyclophilin D)	Ppid	9716	5802	NM_001004279	2q32	1.67	0.000
1394136_at	PAP associated domain containing 1 (predicted)	Papd1_predicted	1567	936	NM_001107359 /// XM_001056452 /// XM_225468	17q12.1	1.67	0.001
1388529_at	RNA terminal phosphate cyclase domain 1	Rtcd1	5030	3005	NM_001004227	2q41	1.67	0.002
1393014_at	similar to hypothetical protein FLJ30656 (predicted)	RGD1309441_predicted	838	501	NM_001105843 /// XM_001081506 /// XM_213467	10q32.1	1.67	0.001
1378568_a_at	similar to chromosome 14 open reading frame 138 (predicted)	RGD1306228_predicted	2412	1441	XM_001080392 /// XM_343073	6q24	1.67	0.000
1389608_at	ATP-binding cassette, sub-family F (GCN20), member 2 (predicted)	Abcf2_predicted	1521	909	NM_001109666 /// XM_001057136 /// XM_231307	4q11	1.67	0.002
1388134_at	eukaryotic translation elongation factor 1 delta (guanine nucleotide exchange protein)	Eef1d	10171	6084	NM_001013104	7q34	1.67	0.002
1370861_at	cytochrome c oxidase, subunit VIa, polypeptide 1	Cox6a1	9588	5737	NM_012814	12q16	1.67	0.001

Table S2. Trophoblast stem-associated genes

Affymetrix ID	Gene Title	Gene Symbol	Stem Ave	Dif Ave	GenBank	romosomal Locat	Stem/ Dif	t-test
1385806_at	Retinitis pigmentosa 9 homolog (human) (predicted)	Rp9h_predicted	1087	650	NM_001108756 /// XM_001074462 /// XM_343363	8q13	1.67	0.012
1382059_at	F-box protein 30	Fbxo30	1624	972	NM_001007690	1p13	1.67	0.011
1395044_at	hypothetical protein LOC679298 /// hypothetical protein LOC688832	LOC679298 /// LOC688832	1649	988	XM_001055692 /// XM_001068496	13q24	1.67	0.034
1382567_at	phosphatidylserine receptor	Nmt2	2432	1456	AI013639	17	1.67	0.001
1386711_a_at	DnaJ (Hsp40) homolog, subfamily C, member 11 (predicted)	Dnajc11_predicte d	931	558	NM_001108694 /// XM_001076236 /// XM_342983	5q36	1.67	0.005
1389336_at	processing of precursor 5, ribonuclease P/MRP family (S. cerevisiae) (predicted)	Pop5_predicted	911	546	NM_001105752 /// XM_001077549 /// XM_213794	12q16	1.67	0.001
1367907_a_at	clathrin, light polypeptide (Lcb)	Cltb	2336	1401	NM_053835	17p14	1.67	0.008
1388180_at	phosphorylated adaptor for RNA export	Phax	2587	1551	NM_173133	18q12.1	1.67	0.001
1398899_at	polymerase (RNA) II (DNA directed) polypeptide C	Polr2c	3298	1979	NM_001012473	19p12	1.67	0.001
1373143_at	similar to hypothetical protein FLJ10652	RGD1309621	4037	2422	XM_001075984 /// XM_229579	4q44	1.67	0.004
1390257_at	vesicle-associated membrane protein, associated protein B and C	Vapb	2236	1342	NM_021847	3q42	1.67	0.019
1372311_at	---	---	2729	1639	BG380795	---	1.67	0.002
1371714_at	Serine/arginine-rich protein specific kinase 1	Srpk1	800	480	NM_001025726	20p12	1.67	0.023
1399029_at	ubiquitin specific protease 48	Usp48	1480	889	NM_198785	5q36	1.66	0.001
1377202_at	SMC6 structural maintenance of chromosomes 6-like 1 (yeast) (predicted)	Smc6l1_predicted	1076	647	NM_001108014 /// XM_001068591 /// XM_233970	6q14	1.66	0.001
1384005_at	down-regulator of transcription 1	Dr1	805	484	NM_001011914	14p22	1.66	0.003
1389510_at	Ly1 antibody reactive clone	Lyar	5073	3048	NM_001011911	14q21	1.66	0.007
1373155_at	mitochondrial ribosomal protein L46	Mrpl46	1303	783	NM_001013068	1q31	1.66	0.004
1374711_at	cleavage and polyadenylation specificity factor 3	Cpsf3	3684	2215	NM_001030030	6q16	1.66	0.000
1376599_at	ATPase family, AAA domain containing 2 (predicted)	Atad2_predicted	877	527	XM_001068382 /// XM_235326	7q33	1.66	0.009
1398786_at	proteasome (prosome, macropain) subunit, beta type 2	Psmb2	7293	4388	NM_017284	5q36	1.66	0.001
1390019_at	H3 histone, family 3B	H3f3b	10958	6595	NM_053985	10q32.3	1.66	0.014
1371403_at	chaperonin subunit 3 (gamma)	Cct3	11933	7186	NM_199091	2q34	1.66	0.000
1379262_at	similar to acyl-CoA thioesterase	LOC302640	1381	832	NM_001013960	Xq22	1.66	0.033
1392473_at	family with sequence similarity 29, member A (predicted)	Nmt2	4842	2917	AW525353	17	1.66	0.000
1390039_at	zinc finger, DHHC domain containing 3	Zdhhc3	921	555	NM_001039014	8q32	1.66	0.005
1372634_at	ADP-ribosylhydrolase like 2 (predicted)	Adprhl2_predicted	1174	708	NM_001108680 /// XM_001057958 /// XM_342918	5q36	1.66	0.004
1383703_at	Inhibitor of DNA binding 2, dominant negative helix-loop-helix protein	Fusip1	1437	867	BE109151	5	1.66	0.005
1387773_at	cytochrome c, somatic	Cycs	8307	5012	NM_012839	4q24	1.66	0.005
1373552_at	LAS1-like (S. cerevisiae) (predicted)	Las1l_predicted	1074	648	XM_001067427 /// XM_216095	Xq31	1.66	0.012
1372628_at		Ap4s1_predicted	3740	2257	AA800277	6	1.66	0.010
1370356_at	RNA binding motif protein 10	Rbm10	1356	819	NM_152861	Xq12	1.66	0.022
1386894_at	heat shock protein 1 (chaperonin)	Hspd1	23644	14287	NM_022229	9q22	1.65	0.015

Table S2. Trophoblast stem-associated genes

Affymetrix ID	Gene Title	Gene Symbol	Stem Ave	Dif Ave	GenBank	romosomal Locat	Stem/ Dif	t-test
1368031_at	nucleolar and coiled-body phosphoprotein 1	Nolc1	1634	987	NM_022869	1q54	1.65	0.000
1368230_a_at	nuclear protein E3-3	RGD708545	1262	763	NM_001033971 /// NM_020080	8q32	1.65	0.001
1383073_at	ubiquitin specific protease 14	Usp14	8774	5303	NM_001008301	18p13	1.65	0.000
1382052_at		Fusip1	1385	837	BF284373	---	1.65	0.007
1373389_at	acyl-Coenzyme A dehydrogenase family, member 9	Acad9	1791	1083	XM_001059349 /// XM_001061665 /// XM_001061715 /// XM_574921	2q25	1.65	0.000
1389646_at	CDC23 (cell division cycle 23, yeast, homolog)	Cdc23	1322	799	NM_001100659 /// XM_001061467 /// XM_214588	18p12	1.65	0.000
1382131_at	SKB1 homolog (S. pombe) (predicted)	Arhgap5	1025	620	AW523674	---	1.65	0.015
1369636_at	sorbitol dehydrogenase	Sord	1079	653	NM_017052	3q35	1.65	0.020
1372517_at	peptidylprolyl isomerase (cyclophilin)-like 1	Ppil1	1646	996	NM_001034188 /// XM_001069671	20p12	1.65	0.005
1392464_at	RAE1 RNA export 1 homolog (S. pombe)	Rae1	3367	2038	NM_001033708	3q42	1.65	0.000
1394371_at	nuclear fragile X mental retardation protein interacting protein 1	Nufip1	853	517	NM_001007758	15q11	1.65	0.004
1383752_at	nucleolar protein 1 (predicted)	Nol1_predicted	1947	1179	XM_001065060 /// XM_235295	4q42	1.65	0.004
1382043_at	unc-93 homolog B1 (C. elegans)	Unc93b1	1441	872	NM_001108513 /// XM_001069535 /// XM_341972	1q42	1.65	0.002
1389577_at	cirrhosis, autosomal recessive 1A (human)	Cirh1a	5281	3199	NM_001009640	19q12	1.65	0.004
1379810_at	small inducible cytokine subfamily E, member 1	Scye1	3145	1905	NM_053757	2q43	1.65	0.001
1373294_at	COMM domain containing 8 (predicted)	Commd8_predicted	3705	2245	NM_001106004 /// XM_001069497 /// XM_214033	14p11	1.65	0.004
1374804_at	guanine nucleotide binding protein-like 2 (nucleolar)	Gnl2	1655	1002	NM_001025736	5q36	1.65	0.004
1389760_at	polycomb group ring finger 6	Pcgf6	3357	2034	NM_001013154	1q54	1.65	0.000
1372867_at	RNA (guanine-7-) methyltransferase	Rnmt	1020	618	NM_001008299	18q12.1	1.65	0.007
1371958_at	poly(A) binding protein, nuclear 1	Pabpn1	1169	708	XM_001055786 /// XM_214172	15p13	1.65	0.013
1373633_at	Transcribed locus	---	857	520	BF399791	---	1.65	0.001
1392829_at	similar to cleavage stimulation factor, 3 pre-RNA subunit 2	LOC683927	882	535	XM_001068092	---	1.65	0.010
1373653_at	PAP associated domain containing 1 (predicted)	Papd1_predicted	1928	1170	NM_001107359 /// XM_001056452 /// XM_225468	17q12.1	1.65	0.000
1388642_at	etoposide induced 2.4 mRNA	Ei24	1967	1193	NM_001025660	8q21	1.65	0.002
1371790_at	mitochondrial ribosomal protein L45 (predicted)	Mrpl45_predicted	2476	1503	NM_001105834 /// XM_001081368 /// XM_213446	10q31	1.65	0.000
1389222_at	similar to Serum amyloid A-3 protein precursor	LOC687992 /// LOC691143	1594	968	XR_007025 /// XR_009408	1q22	1.65	0.000
1393042_at	Ubiquitin specific protease 7 (herpes virus-associated)	Usp7	2685	1631	NM_001024790	10q12	1.65	0.005

Table S2. Trophoblast stem-associated genes

Affymetrix ID	Gene Title	Gene Symbol	Stem Ave	Dif Ave	GenBank	romosomal Locat	Stem/ Dif	t-test
1384390_at	similar to jumonji protein	LOC681740	1660	1009	XM_001056690 /// XM_001056762 /// XM_001056826 /// XM_001056897 /// XM_001056960	---	1.65	0.004
1388793_at	phosphatidylinositol glycan, class Q	Pigq	1431	869	NM_001007607	10q12	1.65	0.013
1382009_at	chromodomain helicase DNA binding protein 1 (predicted)	Chd1_predicted	2226	1352	NM_001107465 /// XM_001056703 /// XM_238731	1q12	1.65	0.001
1372942_at	exosome component 5 (predicted)	Exosc5_predicted	820	499	NM_001107493 /// XM_218343	1q21	1.64	0.017
1398762_at	syndecan binding protein	Sdcbp	12589	7658	NM_031986	5q12	1.64	0.039
1374227_at	similar to hypothetical protein FLJ20272 (predicted)	RGD1305264_predicted	1775	1080	NM_001106706 /// XM_001065384 /// XM_216644	6q12	1.64	0.002
1367931_a_at	polypyrimidine tract binding protein 1	Ptbp1	7160	4359	NM_022516	7q11	1.64	0.000
1389291_at	coiled-coil-helix-coiled-coil-helix domain containing 3 (predicted)	Chchd3_predicted	2962	1804	NM_001106588 /// XM_001066573 /// XM_238346	4q22	1.64	0.003
1375439_at	WD repeat domain 18	Wdr18	1094	667	NM_001039027	7q11	1.64	0.000
1388366_at	mitochondrial ribosomal protein L4 (predicted)	Mrpl4_predicted	2008	1224	NM_001108754 /// XM_001071006 /// XM_343354	8q13	1.64	0.000
1371888_at	mitochondrial ribosomal protein L24	mrpl24	2255	1375	NM_001007637	2q34	1.64	0.001
1379028_at	sperm associated antigen 7 (predicted)	Spag7_predicted	1209	737	NM_001107016 /// XM_001079933 /// XM_220574	10q24	1.64	0.002
1385471_at	DEAD (Asp-Glu-Ala-Asp) box polypeptide 27	Ddx27	916	558	XM_001075340 /// XM_342582	3q42	1.64	0.008
1370844_at	heterogeneous nuclear ribonucleoprotein F	Hnrpf	8182	4992	NM_001037285 /// NM_001037286 /// NM_001037287 /// NM_022397	4q42	1.64	0.000
1374326_at	peter pan homolog (Drosophila)	Ppan	1835	1120	NM_001011980	8q13	1.64	0.012
1389215_at	selenophosphate synthetase 1	Sephs1	1411	861	NM_001104630 /// XM_001066465 /// XM_214509	17q12.3	1.64	0.004
1399020_at	similar to family with sequence similarity 40, member A	LOC362012	812	496	XM_001068196 /// XM_001068248 /// XM_001068288 /// XM_001075052 /// XM_342311	2q34	1.64	0.002
1370939_at	acyl-CoA synthetase long-chain family member 1	Acsl1	849	518	NM_012820	16q11	1.64	0.028
1373868_at		Bclaf1	1800	1100	AI717547	1	1.64	0.006
1383271_at	coiled-coil domain containing 59 (predicted)	Ccdc59_predicted	859	525	NM_001108090 /// XM_001080741 /// XM_235154	7q21	1.64	0.009
1379320_at	Small nuclear ribonucleoprotein D1 (predicted)	Snrpd1_predicted	1754	1072	NM_001106163 /// XM_001060646 /// XM_214621	18p13	1.64	0.003
1372575_at	WW domain binding protein 11	Wbp11	2360	1442	NM_001009661	4q43	1.64	0.001
1372017_at	diablo homolog (Drosophila)	Diablo	1706	1044	NM_001008292	12q16	1.63	0.002
1389237_at	zinc finger, C3HC-type 1 (predicted)	Zc3hc1_predicted	1083	662	XM_001063052 /// XM_216113	4q22	1.63	0.002
1390333_at	protein phosphatase 4, catalytic subunit	Ppp4c	2055	1257	NM_134359	1q36	1.63	0.002

Table S2. Trophoblast stem-associated genes

Affymetrix ID	Gene Title	Gene Symbol	Stem Ave	Dif Ave	GenBank	romosomal Locat	Stem/ Di	t-test
1374279_at	small inducible cytokine subfamily E, member 1	Scye1	3123	1912	NM_053757	2q43	1.63	0.000
1382661_at	nucleoporin 160 (predicted)	Nup160_predicted	3595	2204	NM_001107744 /// XM_001077803 /// XM_230286	3q24	1.63	0.003
1395328_at	similar to hypothetical protein FLJ20003 (predicted)	RGD1309025_predicted	966	592	NM_001106301 /// XM_001080533 /// XM_215089	1q37	1.63	0.001
1375906_at	hypothetical protein LOC683034	LOC683034	830	509	XM_001061331 /// XM_001061393	20	1.63	0.016
1396187_at	DEAD (Asp-Glu-Ala-Asp) box polypeptide 21a	Ddx21a	4770	2926	NM_001037201 /// XM_001053000	20q11	1.63	0.014
1393033_at	tyrosyl-tRNA synthetase 2 (mitochondrial)	Yars2	1579	969	NM_001009627	11q23	1.63	0.005
1383160_at	cysteine and histidine-rich domain (CHORD)-containing, zinc-binding protein 1 (predicted)	Chordc1_predicted	4313	2648	NM_001108128 /// XM_001075005 /// XM_235878	8q12	1.63	0.000
1385765_at	similar to lin-9 homolog (C. elegans)	LOC360888	1705	1047	XM_001061895	13q26	1.63	0.002
1388976_at	similar to BolA domain-containing protein like (11.4 kD) (1P25) (predicted)	RGD1305975_predicted	1095	673	NM_001106601 /// XM_001072676 /// XM_216181	4q34	1.63	0.004
1374082_at	similar to PRO1853 homolog	RGD1311578	1293	795	NM_001008318	6q11	1.63	0.003
1373913_at	polyribonucleotide nucleotidyltransferase 1	Pnpt1	2591	1593	XM_001065076 /// XM_341266	14q22	1.63	0.000
1388582_at	proteaseome (prosome, macropain) 28 subunit, 3	Psme3	4396	2703	NM_001011894	10q32.1	1.63	0.000
1367678_at	succinate dehydrogenase complex, subunit A, flavoprotein (Fp)	Sdha	5088	3132	NM_130428	1p11	1.62	0.001
1371699_at	Transcribed locus, moderately similar to NP_005132.2 fibrinogen, beta chain preproprotein [Homo sapiens]	---	3415	2103	BI295739	---	1.62	0.002
1399130_at	similar to RIKEN cDNA 2610022G08	LOC502782	821	506	NM_001024370	4q24	1.62	0.016
1372918_at		RGD1566291_predicted	877	541	BM385785	---	1.62	0.001
1382921_at	SDA1 domain containing 1	Sdad1	1256	775	NM_001006958	14p22	1.62	0.001
1374927_at	Transcribed locus	---	1017	627	AI714124	---	1.62	0.001
1372496_at	similar to hypothetical protein MGC19604 (predicted)	RGD1561264_predicted	1660	1024	NM_001108002 /// XM_001077345 /// XM_233740	8q13	1.62	0.015
1371505_at	heterogeneous nuclear ribonucleoprotein C	Hnrpc	10716	6614	NM_001025633	15p14	1.62	0.003
1370243_a_at	prothymosin alpha	Ptma	23845	14726	NM_021740	9q35	1.62	0.000
1385073_at	---	---	5174	3195	AI145639	6	1.62	0.017
1368573_at	karyopherin (importin) beta 1	Kpnb1	2756	1703	NM_017063 /// XM_001081365	10q32.1	1.62	0.038
1371789_at	similar to Putative ATP-dependent Clp protease proteolytic subunit, mitochondrial precursor (Endopeptidase Clp)	LOC301117	1922	1188	XM_001055676 /// XM_217313	9q11	1.62	0.000
1383028_at	similar to CDV-3B	LOC315970	1038	641	NM_001014097	8q32	1.62	0.034
1387899_at	collapsin response mediator protein 1	Crmp1	800	494	NM_012932	14q21	1.62	0.005
1395455_at	heterogeneous nuclear ribonucleoprotein H3 (2H9) (predicted)	Hnrph3_predicted	1531	947	NM_001108532 /// XM_001074684 /// XM_342131	20p11	1.62	0.016

Table S2. Trophoblast stem-associated genes

Affymetrix ID	Gene Title	Gene Symbol	Stem Ave	Dif Ave	GenBank	romosomal Locat	Stem/ Di	t-test
1388368_at	mediator of RNA polymerase II transcription, subunit 28 homolog (yeast) (predicted)	Med28_predicted	3200	1979	NM_001107217 /// XM_001059017 /// XM_223463	14q21	1.62	0.022
1370253_at	ribosomal protein L22	Rpl22	22344	13818	NM_031104	5q36	1.62	0.017
1390040_at	brain and reproductive organ-expressed protein	Bre	1461	904	NM_199270	6q13	1.62	0.000
1372151_at	transcription elongation regulator 1 (CA150) (predicted)	Tcerg1_predicted	3745	2318	NM_001107390 /// XM_001068365 /// XM_225983	18p11	1.62	0.001
1372077_at	serine/threonine kinase receptor associated protein	Strap	6724	4161	NM_001011969	4q44	1.62	0.002
1382051_at	similar to J-type co-chaperone HSC20 (predicted)	RGD1311005_predicted	1132	700	NM_001108340 /// XM_001080654 /// XM_341099	12q16	1.62	0.001
1383625_a_at	similar to zinc finger protein (predicted)	RGD1562173_predicted	3346	2071	XM_001064960 /// XM_576402	8q23	1.62	0.004
1389108_at	similar to tubulin-specific chaperone d	LOC363309	1106	686	XM_001081857 /// XM_343649	---	1.61	0.003
1383051_at	Synaptotagmin binding, cytoplasmic RNA interacting protein	Syncrip	3142	1948	NM_001047916 /// XM_001065902 /// XM_343446	8q31	1.61	0.009
1371311_at	succinate dehydrogenase complex, subunit C, integral membrane protein	Sdhc	5857	3632	NM_001005534	13q24	1.61	0.012
1374186_at	Transcribed locus, weakly similar to XP_001475219.1 PREDICTED: similar to Lrrc47 protein [Mus musculus]	---	1519	942	BE109362	5	1.61	0.001
1371586_at	mitochondrial ribosomal protein L48 (predicted)	Mrpl48_predicted	1160	719	NM_001106282 /// XM_001067066 /// XM_215009	1q32	1.61	0.001
1388182_at	DNA primase, p49 subunit	Prim1	10518	6524	NM_001008768	7q11	1.61	0.000
1391201_at	WD repeat and HMG-box DNA binding protein 1 (predicted)	Wdhd1_predicted	1144	710	NM_001107255 /// XM_001062583 /// XM_223933	15p14	1.61	0.013
1372461_at	SET translocation (predicted)	Set_predicted	16307	10118	NM_001012504	19q12	1.61	0.002
1388833_at	polymerase (DNA directed), epsilon 3 (p17 subunit)	Pole3	1175	729	NM_001007652	5q24	1.61	0.010
1376811_a_at	cleavage and polyadenylation specific factor 6, 68kDa (predicted)	Cpsf6_predicted	2279	1415	NM_001106785 /// XM_001080973 /// XM_216893	7q22	1.61	0.041
1379858_at	methyltransferase like 2 (predicted)	Mettl2_predicted	1479	918	NM_001108839 /// XM_001081558 /// XM_343972	10q32.1	1.61	0.040
1389247_at	similar to polymerase (RNA) III (DNA directed) (155kD)	RGD1305574	1199	744	XM_001056048 /// XM_341388	16p16	1.61	0.011
1372529_at	Transcribed locus	---	1677	1042	BE116914	---	1.61	0.043
1372962_at	TAR (HIV) RNA binding protein 2	Tarbp2	987	613	NM_001034941	7q36	1.61	0.026
1389442_at	Transcribed locus	---	1852	1151	AI406804	---	1.61	0.009
1371544_at	similar to Enhancer of rudimentary homolog	LOC681415	12287	7637	NM_001109442 /// XM_001053649 /// XM_001061649	6q24	1.61	0.008
1394120_a_at	Transcribed locus	---	987	614	AI176775	---	1.61	0.028
1388398_at	similar to 40S ribosomal protein S4, X isoform	LOC690845	8263	5137	NM_001109612 /// XM_001074729 /// XM_001076735	4q44	1.61	0.000
1389182_at	similar to hypothetical protein FLJ37953 (predicted)	RGD1311269_predicted	833	518	XM_001067847 /// XM_217397	9q31	1.61	0.013
1387299_at	Msx-interacting-zinc finger	Miz1	3273	2036	NM_053337	18q12.3	1.61	0.011

Table S2. Trophoblast stem-associated genes

Affymetrix ID	Gene Title	Gene Symbol	Stem Ave	Dif Ave	GenBank	romosomal Locat	Stem/ Dif	t-test
1367543_at	similar to Protein SYS1 homolog	LOC685079	1429	889	XM_001062200	3q42	1.61	0.002
1375901_at	DEAD (Asp-Glu-Ala-Asp) box polypeptide 21a	Ddx21a	9023	5614	NM_001037201 /// XM_001053000	20q11	1.61	0.002
1392830_at	chromodomain helicase DNA binding protein 1 (predicted)	Chd1_predicted	1188	739	NM_001107465 /// XM_001056703 /// XM_238731	1q12	1.61	0.006
1367509_at	similar to general transcription factor IIH, polypeptide 5 (predicted)	RGD1560991_predicted	2380	1481	XM_001067341 /// XM_577689	1q11	1.61	0.001
1373437_at	protein phosphatase 1, regulatory (inhibitor) subunit 8 (predicted)	Ppp1r8_predicted	2072	1290	NM_001107911 /// XM_001064335 /// XM_232739	5q36	1.61	0.000
1388417_at	anaphase-promoting complex subunit 5 (predicted)	Anapc5_predicted	4522	2817	NM_001080147 /// XM_001079423 /// XM_213783	12q16	1.61	0.001
1376941_at	Williams-Beuren syndrome chromosome region 16 homolog (human) (predicted)	Wbscr16_predicted	993	619	NM_001108332 /// XM_001077926 /// XM_341066	12q12	1.60	0.002
1371657_at	ubiquitin-like 1 (sentrin) activating enzyme E1B	Uble1b	8499	5299	XM_001076929 /// XM_218502	1q21	1.60	0.003
1382101_at	heparan sulfate 2-O-sulfotransferase 1	Hs2st1	1691	1055	XM_001079125 /// XM_214740	2q44	1.60	0.012
1374316_at	similar to arsenate resistance protein 2	LOC686980	2901	1810	XM_001076594	3	1.60	0.015
1389633_at	polycystic kidney and hepatic disease 1-like 1	E2f4_predicted	2211	1379	BI290172	19	1.60	0.001
1372882_at	similar to CG12379-PA (predicted)	RGD1308635_predicted	866	540	XM_001078337 /// XM_216011	3p13	1.60	0.004
1374714_at	---	---	2209	1379	BI284781	---	1.60	0.005
1394095_at	similar to RIKEN cDNA 4930429M06Rik	RGD1307449	1923	1200	NM_001025012	9q36	1.60	0.000
1382161_at	M-phase phosphoprotein 10 (U3 small nucleolar ribonucleoprotein) (predicted)	Mphosph10_predicted	1576	984	NM_001106340 /// XM_001058593 /// XM_238166	1q22	1.60	0.001
1382755_at	Transcribed locus	---	3011	1882	AI058343	---	1.60	0.001
1376357_at	similar to Ankyrin repeat and IBR domain-containing protein 1	LOC368062	1758	1099	XM_001064190 /// XM_347227	4q13	1.60	0.000
1369996_at	polymerase (RNA) II (DNA directed) polypeptide F	Polr2f	2762	1728	NM_031335	7q34	1.60	0.001
1373384_at	Similar to Serine/threonine-protein phosphatase 2A 56 kDa regulatory subunit gamma isoform (PP2A, B subunit, B gamma isoform) (PP2A, B subunit, B56 gamma isoform) (PP2A, B subunit, PR61 gamma isoform) (PP2A, B subunit, R5 gamma isoform) (PP2A, B su...	LOC691318	842	528	XM_001077680	6q32	1.60	0.000
1398946_at	similar to mitochondrial ribosomal protein S16	LOC688912	1981	1241	NM_001109518 /// XM_001064095 /// XM_001068806	15p16	1.60	0.004
1373075_at	similar to Cell division protein kinase 8 (Protein kinase K35) (predicted)	RGD1560888_predicted	3861	2420	NM_001109061 /// XM_001061556 /// XM_573350	12p11	1.60	0.002
1368890_at	glyceronephosphate O-acyltransferase	Gnpat	910	571	NM_053410	19q12	1.59	0.020
1370238_at	upregulated during skeletal muscle growth 5	Usmg5	11802	7400	NM_133544	1q54	1.59	0.000
1371341_at	similar to small nuclear ribonucleoprotein D2	LOC680309	8855	5553	NM_001109399 /// XM_001056581	1q21	1.59	0.041

Table S2. Trophoblast stem-associated genes

Affymetrix ID	Gene Title	Gene Symbol	Stem Ave	Dif Ave	GenBank	romosomal Locat	Stem/ Di	t-test
1373077_at	similar to hypothetical protein D11Ertd497e	RGD1308696	2514	1576	NM_001008278	10q22	1.59	0.000
1370284_at	ATP synthase, H+ transporting, mitochondrial F1 complex, epsilon subunit	Atp5e	8511	5339	NM_139099	14q21	1.59	0.003
1371720_at	similar to mitochondrial ribosomal protein L20	LOC680747	2254	1414	NM_001109428 /// XM_001058684 /// XM_001077713	5q36	1.59	0.001
1372141_at	similar to Prefoldin subunit 2	LOC678975 /// LOC685607	5961	3740	NM_001109476 /// XM_001055322 /// XM_001055378 /// XM_001061711 /// XM_001061762	13q24	1.59	0.001
1398343_at	similar to DnaJ (Hsp40) homolog, subfamily A, member 4	LOC498996	1215	762	XR_006633 /// XR_007683	1p11	1.59	0.001
1398804_at	MAK10 homolog, amino-acid N-acetyltransferase subunit, (S. cerevisiae)	Mak10	1948	1222	NM_133324	17p14	1.59	0.007
1392999_at	neuropilin (NRP) and tolloid (TLL)-like 2 (predicted)	Neto2_predicted	1199	753	NM_001107417 /// XM_001068307 /// XM_226349	19q11	1.59	0.002
1374716_at	similar to RIKEN cDNA 2810430M08	RGD1306106	1271	798	NM_001009702	13q26	1.59	0.006
1395758_at	solute carrier family 25 (mitochondrial carrier, ornithine transporter) member 15	Syncrip	929	583	AI548569	8	1.59	0.032
1367720_at	aminolevulinate, delta-, dehydratase	Alad	3543	2224	NM_012899	5	1.59	0.005
1376037_at	achalasia, adrenocortical insufficiency, alacrimia (Allgrove, triple-A) (predicted)	Aaas_predicted	816	513	NM_001106795 /// XM_001067255 /// XM_217063	7q36	1.59	0.029
1388622_at	nucleolar protein 5A	Nol5a	7764	4877	NM_001025732	3q36	1.59	0.001
1389154_at	lipoic acid synthetase	Lias	1467	922	NM_001012037	14p11	1.59	0.003
1391786_at	Similar to Murine homolog of human ftp-3	LOC308650	1294	813	NM_001014019	Xq34	1.59	0.013
1388306_at	similar to RIKEN cDNA 1810042K04 (predicted)	RGD1305593_predicted	1157	727	XM_001065112	12q11	1.59	0.004
1388953_at	guanine nucleotide binding protein-like 3 (nucleolar)	Gnl3	8635	5430	NM_175580	16p16	1.59	0.004
1371655_at	mannose-P-dolichol utilization defect 1	Mpdu1	1266	796	NM_001107011 /// XM_001078776 /// XM_220606	10q24	1.59	0.018
1383965_at	similar to SMP3 mannosyltransferase	LOC684506	6105	3840	XM_001066087 /// XM_001066140 /// XM_001066197 /// XM_001066307	11	1.59	0.001
1398883_at	hypothetical protein LOC685548	LOC685548	8176	5144	XM_001064266	4q24	1.59	0.025
1390408_at	holocytochrome c synthetase (predicted)	Hccs_predicted	984	619	XM_001065465 /// XM_228867	Xq21	1.59	0.023
1371255_at	Harvey rat sarcoma viral (v-Ha-ras) oncogene homolog	Hras	1371	864	XM_001061671 /// XM_001062236	1q41	1.59	0.003
1367754_s_at	argininosuccinate lyase	Asl	883	557	NM_021577	12q13	1.59	0.007
1392936_at	RNA binding motif protein 25 (predicted) /// similar to RNA binding motif protein 25 (predicted)	Rbm25_predicted /// RGD1565486_predicted	1301	820	NM_001108984 /// XM_001055193 /// XM_001079014 /// XM_345703 /// XM_573045	10q12 /// 6q24-q31	1.59	0.011
1373707_at	Transcribed locus	---	1737	1095	BM382865	---	1.59	0.025
1368063_a_at	splicing factor YT521-B	Yt521	3505	2210	NM_133423	14p21	1.59	0.002

Table S2. Trophoblast stem-associated genes

Affymetrix ID	Gene Title	Gene Symbol	Stem Ave	Dif Ave	GenBank	romosomal Locat	Stem/ Dif	t-test
1370911_at	A kinase (PRKA) anchor protein 8	Akap8	3820	2409	XM_001074708 /// XM_343176	7q11	1.59	0.009
1383520_at	solute carrier family 25 (mitochondrial carrier, ornithine transporter) member 15	Sfrs12	1675	1057	AA859127	2	1.58	0.009
1370888_at	cytochrome c oxidase, subunit Va	Cox5a	13237	8352	NM_145783	8q24	1.58	0.003
1373875_at	similar to RIKEN cDNA 1190005P17 (predicted) /// hypothetical protein LOC690089	LOC690089 /// RGD1308261_predicted	1020	644	XM_001081031 /// XM_216898 /// XR_006788	6q32 /// 7q22	1.58	0.002
1395354_at	lanC (bacterial lantibiotic synthetase component C)-like 1	Lancl1	1195	755	NM_053723	9q32	1.58	0.022
1372167_at	similar to small nuclear ribonucleoprotein polypeptide G	LOC681031 /// LOC687679	7422	4689	XM_001058102 /// XM_001058164 /// XM_001058230 /// XM_001069500 /// XM_001069539 /// XM_001069586 /// XM_001079708	4q34	1.58	0.000
1398583_at	similar to small nuclear RNA activating complex, polypeptide 5	LOC691501	1173	742	NM_001109643 /// XM_001076046 /// XM_001078552	8q24	1.58	0.007
1386918_a_at	opioid receptor, sigma 1	Oprs1	1103	697	NM_030996	5q22	1.58	0.004
1392460_at	---	---	821	519	BF283685	---	1.58	0.002
1367769_at	polymerase (RNA) II (DNA directed) polypeptide G	Polr2g	5415	3424	NM_053948	1q43	1.58	0.023
1374182_at	SWI/SNF related, matrix associated, actin dependent regulator of chromatin, subfamily c, member 1 (predicted)	Smarcc1_predicted	1619	1024	NM_001106861 /// XM_001076931 /// XM_001076956 /// XM_001076982 /// XM_001077002 /// XM_001077020 /// XM_001077042 /// XM_236644	8q32	1.58	0.014
1389569_at	brix domain containing 2	Bxdc2	5623	3556	NM_001029915	2q16	1.58	0.006
1371745_at	hypothetical LOC300361	RGD1310669	2973	1880	NM_001024987	8q11	1.58	0.001
1373475_at	coiled-coil domain containing 58 (predicted)	Ccdc58_predicted	5177	3275	NM_001105875 /// XM_001071079 /// XM_213612	11q22	1.58	0.000
1372764_at	similar to density-regulated protein	LOC687565 /// LOC689601	4753	3006	XM_001067744 /// XM_001067805 /// XM_001079167	12q15	1.58	0.001
1371302_at	similar to Small EDRK-rich factor 2 (4F5rel)	LOC502663	8849	5597	NM_001098782 /// XM_001080969 /// XM_578157	3q35	1.58	0.000
1375029_at	hypothetical LOC288447 (predicted)	RGD1310835_predicted	1981	1253	NM_001105906 /// XM_001059748 /// XM_213702	12p11	1.58	0.000
1398834_at	mitogen activated protein kinase kinase 2	Map2k2	1238	783	NM_133283	7q11	1.58	0.004
1371582_at	interleukin enhancer binding factor 2	Ilf2	3712	2352	NM_001047886	2q34	1.58	0.003
1383011_at	eukaryotic translation initiation factor 2A	Eif2a	3988	2527	NM_001109339 /// XM_001058163 /// XM_578017	2q31	1.58	0.025
1379353_at	aminoadipate-semialdehyde dehydrogenase-phosphopantetheinyl transferase (predicted)	Aasdhppt_predicted	2550	1617	NM_001106798 /// XM_001070860 /// XM_217078	8q11	1.58	0.001

Table S2. Trophoblast stem-associated genes

Affymetrix ID	Gene Title	Gene Symbol	Stem Ave	Dif Ave	GenBank	romosomal Locat	Stem/ Dif	t-test
1388354_at	activating signal cointegrator 1 complex subunit 3-like 1	Ascc3l1	3134	1987	BE349703	3q36	1.58	0.009
1368004_at	mitochondrial ribosomal protein L23	Mrpl23	1592	1010	NM_022529	1q41	1.58	0.012
1371324_at	similar to Splicing factor 3B subunit 5 (SF3b5) (Pre-mRNA splicing factor SF3b 10 kDa subunit)	LOC680891 /// LOC684491	3959	2514	XM_001059359 /// XM_001070659	1p13	1.58	0.039
1367740_at	creatine kinase, brain	Ckb	3362	2135	NM_012529	6q32	1.57	0.011
1371822_at	polymerase (RNA) III (DNA directed) polypeptide D	Polr3d	2515	1598	NM_001031653	15p11	1.57	0.015
1373037_at	ubiquitin-conjugating enzyme E2L 6	Ube2l6	1071	681	NM_001024755	3q24	1.57	0.040
1380447_a_at	mitochondrial ribosomal protein S18C (predicted)	Mrps18c_predicted	955	607	NM_001105996 /// XM_001067441 /// XM_214003	14p22	1.57	0.046
1391604_at	similar to mKIAA0056 protein	LOC315508	1084	689	NM_001034000	8q13	1.57	0.029
1370842_at	branched chain ketoacid dehydrogenase kinase	Bckdk	1267	806	NM_019244	1q36	1.57	0.037
1388516_at	LRRGT00141	LOC499235	7180	4568	XM_001076383 /// XM_574528	1q33	1.57	0.010
1383341_at	calcium binding protein 39-like	Cab39l	856	544	NM_001011917	15p12	1.57	0.008
1388803_at	deoxyhypusine synthase	Dhps	1588	1011	NM_001004207	19q11	1.57	0.001
1372174_at	PEF protein with a long N-terminal hydrophobic domain	Peflin	1339	852	NM_001007651	5q36	1.57	0.001
1382139_at	similar to hypothetical protein FLJ12994 (predicted)	RGD1308795_predicted	1025	653	XM_001053787 /// XM_236392	8q24	1.57	0.002
1382783_at	Bloom syndrome homolog (human) (predicted)	Blm_predicted	1608	1024	NM_001107526 /// XM_001066539 /// XM_218837	1q31	1.57	0.012
1388562_at	serine racemase	Stard7_predicted	4584	2919	BI282011	3	1.57	0.042
1372723_at	Importin 9 (predicted)	Ipo9_predicted	4707	2999	NM_001107180 /// XM_001063316 /// XM_222661	13q13	1.57	0.000
1389031_at	similar to Coatomer gamma-2 subunit (Gamma-2 coat protein) (Gamma-2 COP) (predicted)	RGD1566215_predicted	1083	691	NM_001106929 /// XM_001065495 /// XM_217496	4q22	1.57	0.002
1378181_at	ribonuclease P 40 subunit (human)	Rpp40	1302	830	NM_001013055	17p12	1.57	0.005
1395640_at	CDC23 (cell division cycle 23, yeast, homolog)	Cdc23	1069	682	NM_001100659 /// XM_001061467 /// XM_214588	18p12	1.57	0.034
1372931_at	PRA1 domain family 2 (predicted)	Praf2_predicted	801	511	NM_001109013 /// XM_001058767 /// XM_346274	Xq13	1.57	0.002
1382598_at	heat shock factor 2	Hsf2	1782	1137	NM_031694	20q11	1.57	0.001
1373502_at	dymeclin (predicted)	Dym_predicted	1228	784	NM_001106133 /// XM_001054727 /// XM_214529	18q12.3	1.57	0.001
1397697_at	Eukaryotic translation initiation factor 4A2	Eif4a2	1430	913	NM_001008335	11q23	1.57	0.007
1367938_at	UDP-glucose dehydrogenase	Ugdh	4034	2577	NM_031325	14p11	1.57	0.001
1370075_at	dihydrofolate reductase	Dhfr	1063	679	NM_130400	2q12	1.57	0.029
1373119_at	potassium channel tetramerisation domain containing 3	Kctd3	1082	691	NM_001107199 /// XM_001066967 /// XM_223057	13q26	1.57	0.003
1384442_at	claudin 6 (predicted)	Cldn6_predicted	1803	1152	NM_001102364 /// XM_001055688 /// XM_220202	10q12	1.57	0.020
1387039_at	glypican 1	Gpc1	990	632	NM_030828	9q36	1.57	0.000

Table S2. Trophoblast stem-associated genes

Affymetrix ID	Gene Title	Gene Symbol	Stem Ave	Dif Ave	GenBank	romosomal Locat	Stem/ Dif	t-test
1391410_at	Transcribed locus	---	1098	702	BM392039	---	1.56	0.017
1386890_at	S100 calcium binding protein A10 (calpactin)	S100a10	5076	3249	NM_031114	2	1.56	0.034
1373408_at	tubulin cofactor a	Tbca	10349	6626	NM_001013245	7q36	1.56	0.013
1393881_at	NMDA receptor-regulated gene 1 (predicted)	Narg1_predicted	2951	1889	NM_001107674 /// XM_001053949 /// XM_001054009 /// XM_001054056 /// XM_001054116 /// XM_001054179 /// XM_241375	2q26	1.56	0.037
1369939_at	cytochrome c, somatic	Cycs	11102	7111	NM_012839	4q24	1.56	0.020
1399062_at	CDNA clone IMAGE:7317308	---	2160	1384	BI301008	---	1.56	0.010
1367642_at	succinate-CoA ligase, GDP-forming, alpha subunit	Suclg1	2441	1564	NM_053752	4q33	1.56	0.000
1386856_a_at	sterile alpha motif domain containing 4B	Samd4b	2175	1394	NM_001107498 /// XM_001073474 /// XM_001073503 /// XM_001073537 /// XM_218384	1q21	1.56	0.012
1371396_at	tropomyosin 3, gamma	Tpm3	8584	5504	AI600035	2	1.56	0.000
1393088_at	RNA (guanine-9-) methyltransferase domain containing 1	Rg9mtd1	2449	1570	NM_001008337	11q12	1.56	0.003
1392544_at	rcd1 (required for cell differentiation) homolog 1 (S. pombe)	Rqcd1	1607	1031	NM_001009357	9q33	1.56	0.021
1397767_at	Transcribed locus	---	830	532	BG377356	---	1.56	0.012
1372879_at	AKT1 substrate 1 (proline-rich) (predicted)	Akt1s1_predicted	1629	1045	NM_001106259 /// XM_001078095 /// XM_238103	1q22	1.56	0.007
1387918_at	tropomyosin 3, gamma	Tpm3	6470	4153	NM_057208 /// NM_173111	2q34	1.56	0.017
1388956_at	exportin 5 (predicted)	Xpo5_predicted	1327	852	NM_001108789 /// XM_001064242 /// XM_343534	9q12	1.56	0.004
1381224_at	similar to Zinc finger protein 551 (Zinc finger protein KOX23)	LOC684878 /// LOC691396	1195	767	XM_001072292 /// XM_001078065	1q12	1.56	0.044
1370339_at	tropomyosin 3, gamma	Tpm3	13014	8358	NM_057208 /// NM_173111	2q34	1.56	0.019
1390382_at	similar to RIKEN cDNA 2310003F16 (predicted)	RGD1311457_predicted	6367	4090	XM_001080972 /// XM_230510	3q35	1.56	0.003
1372522_at	similar to hypothetical protein FLJ10154	RGD1310061	4640	2982	NM_001024972	16q12.5	1.56	0.019
1372155_at	tripartite motif protein 28	Trim28	7109	4571	XM_001062234 /// XM_344861	1q21	1.56	0.003
1371892_at	ATP synthase mitochondrial F1 complex assembly factor 2 (predicted)	Atpaf2_predicted	860	553	NM_001107006 /// XM_001075912 /// XM_220522	10q22	1.56	0.047
1389547_at	periphilin 1 (predicted)	Pphln1_predicted	1591	1023	NM_001108992 /// XM_001057366 /// XM_345865	7q35	1.55	0.001
1370171_at	heterogeneous nuclear ribonucleoprotein U	Hnrpu	6578	4231	NM_057139	13q26	1.55	0.040
1389980_at	similar to Protein HSPC163 (predicted)	RGD1559740_predicted	4186	2693	NM_001105981 /// XM_001062581 /// XM_213960	13q26	1.55	0.001
1367591_at	peroxiredoxin 3	Prdx3	4309	2773	NM_022540	1q55	1.55	0.001

Table S2. Trophoblast stem-associated genes

Affymetrix ID	Gene Title	Gene Symbol	Stem Ave	Dif Ave	GenBank	romosomal Locat	Stem/ Dif	t-test
1389327_at	mitochondrial ribosomal protein L32 (predicted)	Mrpl32_predicted	3689	2374	NM_001106116 /// XM_001054992 /// XM_214491	17q12.1	1.55	0.004
1387950_at	nuclear import 7 homolog (S. cerevisiae)	Nip7	4006	2583	NM_138847	19q12	1.55	0.002
1392912_at	calcyclin binding protein	Cacybp	7518	4848	NM_001004208	13q22	1.55	0.004
1393451_at	similar to RIKEN cDNA 2610510J17	RGD1310953	2544	1641	NM_001008366	19q12	1.55	0.002
1385988_at	H6 homeo box 1 (predicted)	Hmx1_predicted	827	534	NM_001108363 /// XM_001064149 /// XM_341238	14q21	1.55	0.004
1388638_at	sirtuin 6 (silent mating type information regulation 2, homolog) 6 (S. cerevisiae)	Sirt6	843	544	NM_001031649	7q11	1.55	0.039
1384149_at	similar to hypothetical protein FLJ38348 (predicted)	RGD1311424_predicted	1506	972	NM_001108700 /// XM_001064113 /// XM_343001	6q11	1.55	0.001
1393118_at	armadillo repeat containing 1 (predicted)	Armc1_predicted	1056	681	NM_001106425 /// XM_001055081 /// XM_215537	2q24	1.55	0.005
1392736_at	---	---	6163	3979	AW532939	---	1.55	0.008
1371430_at	dystroglycan 1	Dag1	1484	959	XM_001074892 /// XM_343483	8q32	1.55	0.047
1385553_at	similar to SMP3 mannosyltransferase /// similar to nuclear cap binding protein subunit 2	LOC684506 /// LOC689116	4171	2694	NM_001109525 /// XM_001064395 /// XM_001064452 /// XM_001064512 /// XM_001064621 /// XM_001066087 /// XM_001066140 /// XM_001066197 /// XM_001066307	11q22	1.55	0.002
1370899_at	splicing factor proline/glutamine rich (polypyrimidine tract binding protein associated)	Sfpq	1366	883	NM_001025271 /// XM_001058863	5q36	1.55	0.018
1371881_at	zinc finger, HIT domain containing 2 (predicted)	Znhit2_predicted	864	558	NM_001107574 /// XM_001071296 /// XM_219526	1q43	1.55	0.000
1389289_at	Ewing sarcoma breakpoint region 1	Ewsr1	3676	2375	XM_001065285 /// XM_001065346 /// XM_001068194 /// XM_001068285 /// XM_001068438 /// XM_001068487 /// XM_001068534	14q21	1.55	0.000
1372453_at	down-regulator of transcription 1	Dr1	3092	1998	NM_001011914	14p22	1.55	0.005
1380009_at	vaccinia related kinase 1	Vrk1	2727	1763	NM_001012194	6q32	1.55	0.001
1373185_at	signal sequence receptor, beta (predicted)	Ssr2_predicted	3107	2011	NM_001106442 /// XM_001070197 /// XM_215619	2q34	1.55	0.000
1389197_at	similar to RIKEN cDNA 9630046K23	RGD1306248	1674	1084	XM_001069417 /// XM_221437	11q21	1.54	0.001
1372109_at	---	---	1010	654	AI102715	---	1.54	0.009
1371904_at	SET and MYND domain containing 2	Smyd2	1342	869	XM_001066311 /// XM_213972	13q27	1.54	0.010
1398810_at	PDGFA associated protein 1	Pdap1	9271	6005	NM_022595	12p11	1.54	0.001
1371418_at	chaperonin containing TCP1, subunit 2 (beta)	Cct2	12509	8103	NM_001005905	7q22	1.54	0.002

Table S2. Trophoblast stem-associated genes

Affymetrix ID	Gene Title	Gene Symbol	Stem Ave	Dif Ave	GenBank	romosomal Locat	Stem/ Di	t-test
1374517_at	RecQ protein-like	Recql	1763	1142	NM_001012098	4q44	1.54	0.003
1372563_at	similar to D330021B20 protein	RGD1308143	1898	1229	AA799488	9q36	1.54	0.010
1378493_at	similar to RIKEN cDNA 1810020G14 (predicted)	Fam29a_predicted	1246	807	BE117901	5	1.54	0.003
1370007_at	protein disulfide isomerase associated 4	Pdia4	1772	1148	NM_053849	4q24	1.54	0.014
1371633_at	beta catenin-like 1	Ctnnbl1	2486	1613	NM_001024870	3q42	1.54	0.000
1385521_at	Transcribed locus	---	1306	848	AI603505	---	1.54	0.016
1369976_at	dynein light chain LC8-type 1	Dynll1	12259	7960	NM_053319	12q16	1.54	0.037
1371381_at	similar to ubiquinol-cytochrome c reductase complex 7.2kDa protein isoform b	LOC685322	9460	6145	XM_001063336 /// XM_001067698	14q21	1.54	0.007
1389866_at	similar to 6.8 kDa mitochondrial proteolipid	LOC691427	4630	3008	XM_001072279 /// XM_001078225	6q32	1.54	0.004
1376704_a_at	necdin-like 2	Ndnl2	1060	689	XM_219708	1q22	1.54	0.002
1374301_at	component of oligomeric golgi complex 1 (predicted)	Cog1_predicted	950	618	NM_001107062 /// XM_001081639 /// XM_239373	10q32.1	1.54	0.000
1394027_at	similar to Nucleoporin 62 (predicted)	RGD1564353_predicted	5421	3524	XM_001053696 /// XM_217225	Xq35	1.54	0.002
1370785_s_at	translocase of outer mitochondrial membrane 20 homolog (yeast)	Tomm20	8061	5242	NM_152935	19q12	1.54	0.013
1376069_at	Proteasome (prosome, macropain) 26S subunit, non-ATPase, 11 (predicted)	Psmd11_predicted	4079	2652	NM_001107027 /// XM_001080984 /// XM_220754	10q26	1.54	0.023
1379438_at	SWI/SNF-related, matrix-associated actin-dependent regulator of chromatin, subfamily a, containing DEAD/H box 1` (predicted)	Smarcad1_predicted	1078	701	NM_001107864 /// XM_001066395 /// XM_231860	4q31	1.54	0.014
1387080_at	chondroitin sulfate proteoglycan 6	Cspg6	5598	3641	NM_031583	1q55	1.54	0.000
1372215_at	similar to mitochondrial ribosomal protein S11 (predicted)	RGD1559901_predicted	884	575	NM_001109148 /// XM_001059288 /// XM_574478	1q31	1.54	0.010
1367714_at	eukaryotic translation initiation factor 2B, subunit 2 beta	Eif2b2	1818	1183	NM_032058	6q31	1.54	0.000
1392627_x_at	thiopurine methyltransferase	Zfp84_predicted	16155	10517	BI282114	---	1.54	0.024
1370873_at	calmodulin 3	Calm3	1918	1249	NM_012518	1q22	1.54	0.003
1371585_at	G1 to S phase transition 1	Gspt1	7789	5073	NM_001003978	10q11	1.54	0.001
1371876_at	tumor necrosis factor superfamily, member 5-induced protein 1 (predicted)	Tnfsf5ip1_predicted	3347	2180	NM_001106138 /// XM_001067079 /// XM_214550	18q12.1	1.54	0.000
1388423_at	hypothetical protein MGC:15854	RGD1302996	1323	863	NM_213610	20p12	1.53	0.006
1398778_at	proteasome (prosome, macropain) subunit, alpha type 1	Psma1	6683	4356	NM_017278	1q34	1.53	0.002
1373371_a_at	similar to RIKEN cDNA 1110001J03	MGC112899	1263	824	NM_001037795	4q22	1.53	0.010
1371806_at	DiGeorge syndrome critical region gene 8 (predicted)	Dgcr8_predicted	1079	704	NM_001105865 /// XM_001064513 /// XM_221273	11q23	1.53	0.006
1388316_at	RGD1566320 (predicted)	RGD1566320_predicted	1240	810	NM_001106520 /// XM_001054551 /// XM_238286	3q41	1.53	0.005
1392908_at	hypothetical protein LOC595134	LOC595134	1198	782	NM_001030054	5q36	1.53	0.010
1389041_at	Vac14 homolog (S. cerevisiae)	Vac14	831	543	NM_177930	19q12	1.53	0.004
1373063_at	huntingtin interacting protein 2 (predicted)	Hip2_predicted	7730	5051	NM_001106006 /// XM_001071289 /// XM_214043	14p11	1.53	0.002
1371609_at	homolog of zebrafish ES1	RGD1303003	1119	732	NM_001004225	20p12	1.53	0.005

Table S2. Trophoblast stem-associated genes

Affymetrix ID	Gene Title	Gene Symbol	Stem Ave	Dif Ave	GenBank	romosomal Locat	Stem/ Dif	t-test
1371073_at	UDP-Gal:betaGlcNAc beta 1,4-galactosyltransferase, polypeptide 1	B4galt1_predicted	4104	2683	NM_053287 /// XM_001058948 /// XM_342820	5q22	1.53	0.003
1388752_at	kinesin family member 1B	Bclaf1	3291	2152	AI408078	1	1.53	0.007
1389061_at	NOL1/NOP2/Sun domain family, member 5 (predicted)	Nsun5_predicted	1477	966	XM_001071177 /// XM_213749	12q12	1.53	0.020
1395479_at	brix domain containing 2	Bxdc2	1019	666	NM_001029915	2q16	1.53	0.007
1388990_at	Mki67 (FHA domain) interacting nucleolar phosphoprotein	Mki67ip	4281	2801	NM_139186	13q11	1.53	0.022
1387366_at	interleukin enhancer binding factor 3	Ilf3	1475	965	NM_053412	8q13	1.53	0.035
1391440_at	testis expressed gene 10 (predicted)	Tex10_predicted	1799	1178	NM_001106653 /// XM_001054011 /// XM_001054184 /// XM_001054251 /// XM_216393	5q22	1.53	0.008
1388112_at	solute carrier family 25 (mitochondrial carrier; adenine nucleotide translocator), member 4	Slc25a4	13635	8935	NM_053515	16q11	1.53	0.002
1390340_a_at	eukaryotic translation initiation factor 4 gamma, 1	Eif4g1	907	594	XM_001060756 /// XM_213569	11q23	1.53	0.021
1371651_at	similar to Vacuolar protein sorting protein 25 (ELL-associated protein of 20 kDa)	LOC681059 /// LOC688199	2259	1481	XM_001060127 /// XM_001081476	10q32.1	1.53	0.000
1393193_at	Transcribed locus	---	1183	776	BF522381	---	1.53	0.011
1399065_at	Similar to RNA binding protein with multiple splicing 2 (predicted)	RGD1561222_predicted	974	639	XM_001076681 /// XM_578738	8q24	1.52	0.009
1373704_at	aquarius (predicted)	Aqr_predicted	2171	1424	NM_001100987 /// XM_001074830 /// XM_345418	3q35	1.52	0.002
1367877_at	solute carrier family 11 (proton-coupled divalent metal ion transporters), member 2	Slc11a2	839	551	NM_013173	7q36	1.52	0.031
1398932_at	histidine triad nucleotide binding protein 1 /// histidine triad nucleotide binding protein 1 (predicted) /// similar to Histidine triad nucleotide-binding protein 1 (Adenosine 5-monophosphoramidase) (Protein kinase C inhibitor 1) (Protein kinase C-interacting protein 1) (PKCI-1)	Hint1 /// Hint1_predicted /// LOC690660	10550	6923	NM_001109607 /// NM_022192 /// XM_001073678 /// XM_001074494	10q22 /// 4q24	1.52	0.001
1367943_at	nuclear factor of kappa light chain gene enhancer in B-cells inhibitor, beta	Nfkbib	1283	842	NM_030867	1q21	1.52	0.008
1393045_at	Rho-associated coiled-coil forming kinase 2	Rock2	1012	664	NM_013022	6q15-q16	1.52	0.021
1388160_a_at	isocitrate dehydrogenase 3 (NAD+) beta	Idh3B	3791	2490	NM_053581	3q36	1.52	0.000
1374135_at	importin 4 (predicted)	Ipo4_predicted	1903	1250	NM_001106038 /// XM_001056047 /// XM_214199	15p13	1.52	0.000
1386891_at	phosphatidylethanolamine binding protein 1	Pebp1	4786	3144	NM_017236	12q16	1.52	0.009
1375019_at	heterogeneous nuclear ribonucleoprotein H3 (2H9) (predicted)	Hnrph3_predicted	1253	824	NM_001108532 /// XM_001074684 /// XM_342131	20p11	1.52	0.024
1390848_at	RNA binding motif protein 19 (predicted) /// similar to RNA binding motif protein 19	LOC687727 /// Rbm19_predicted	845	556	NM_001107143 /// XM_001079940 /// XM_222200	12q16	1.52	0.004

Table S2. Trophoblast stem-associated genes

Affymetrix ID	Gene Title	Gene Symbol	Stem Ave	Dif Ave	GenBank	romosomal Locat	Stem/ Dif	t-test
1367551_a_at	similar to CG14980-PB	RGD1306410	3175	2088	NM_001014126	12p11	1.52	0.008
1371424_at	similar to RIKEN cDNA 3110005O21	RGD1304823	882	580	NM_001037196	1q36	1.52	0.031
1389979_at	transportin 3	Tnpo3	991	652	NM_001106587 /// XM_001064267 /// XM_216112	4q22	1.52	0.024
1381364_at	transportin 1	Tnpo1	1945	1280	XM_001070389 /// XM_219500	2q12	1.52	0.023
1374009_at	similar to RIKEN cDNA 5830415L20	RGD1306302	1696	1116	NM_001024799	4q13	1.52	0.001
1393807_at	Transcribed locus	---	1496	985	BF396605	---	1.52	0.026
1388614_at	splicing factor 3b, subunit 2 (predicted)	Sf3b2_predicted	3385	2228	NM_001106326 /// XM_001073034 /// XM_215182	1q43	1.52	0.012
1371704_at	DEAD (Asp-Glu-Ala-Asp) box polypeptide 23 (predicted)	Ddx23_predicted	1824	1201	NM_001106793 /// XM_001060655 /// XM_217050	7q36	1.52	0.013
1398977_at	special AT-rich sequence binding protein 1	Ide	894	589	BF558116	1	1.52	0.010
1388476_at	Tial1 cytotoxic granule-associated RNA binding protein-like 1	Tial1	2650	1746	NM_001013193	1q36	1.52	0.004
1372255_at	arginyl-tRNA synthetase (predicted)	Rars_predicted	7509	4948	NM_001105777 /// XM_001065238 /// XM_213276	10q12	1.52	0.003
1376567_at	similar to Mitotic spindle assembly checkpoint protein MAD1 (Mitotic arrest deficient-like protein 1) (MAD1-like 1)	LOC680006 /// LOC686419	1075	708	NM_001109387 /// XM_001055286 /// XM_001074022	12q11	1.52	0.013
1377551_at	Transcribed locus	---	1069	704	BE118580	---	1.52	0.012
1374876_at	leptin receptor overlapping transcript-like 1	Leprotl1	1259	830	NM_001013188	16q12.2	1.52	0.034
1398572_at	RNA (guanine-7-) methyltransferase	Rnmt	4805	3168	NM_001008299	18q12.1	1.52	0.001
1389319_at	similar to Endoplasmic reticulum-Golgi intermediate compartment protein 1 (ER-Golgi intermediate compartment 32 kDa protein) (ERGIC-32)	LOC287177 /// LOC682838	1158	764	XM_001063333 /// XM_001066818	10q12	1.52	0.000
1389168_at	McKusick-Kaufman syndrome protein	Mkks	1436	947	NM_001008353	3q36	1.52	0.034
1371423_at	mitochondrial ribosomal protein L41	Mrpl41	3737	2465	NM_001013426	3p13	1.52	0.003
1367653_a_at	malate dehydrogenase 1, NAD (soluble)	Mdh1	4263	2813	NM_033235	14q22	1.52	0.002
1371488_at	similar to SPBPJ4664.02 (predicted) /// RGD1562079 (predicted)	RGD1559845_predicted /// RGD1562079_predicted	2696	1780	XM_001079026 /// XM_579917	1q21	1.51	0.029
1375520_at	coatomer protein complex, subunit zeta 1 (predicted)	Copz1_predicted	2435	1608	NM_001108117 /// XM_001069027 /// XM_235705	7q36	1.51	0.022
1379671_at	major facilitator superfamily domain containing 1 (predicted)	Mfsd1_predicted	1519	1004	XM_001061503 /// XM_001061558 /// XM_342258	2q31	1.51	0.001
1373556_at	similar to CG14903-PA (predicted)	RGD1561792_predicted	1923	1271	XM_001070434 /// XM_216648	6q14	1.51	0.000
1389655_at	similar to A230072I16Rik protein	RGD1306819	3611	2389	NM_001013860	10q26	1.51	0.004
1374597_at	Transcribed locus, strongly similar to XP_574009.1 PREDICTED: similar to CDNA sequence BC034664 [Rattus norvegicus]	---	812	537	BI275770	---	1.51	0.015

Table S2. Trophoblast stem-associated genes

Affymetrix ID	Gene Title	Gene Symbol	Stem Ave	Dif Ave	GenBank	romosomal Locat	Stem/ Di	t-test
1367500_at	similar to 26 proteasome complex subunit DSS1 (Deleted in split hand/split foot protein 1) (Split hand/foot deleted protein 1 homolog)	LOC680532	5470	3621	XM_001054221 /// XM_001057613	4q13	1.51	0.004
1393458_s_at	similar to PHD finger protein 14 isoform 1 (predicted)	RGD1563764_predicted	1938	1283	NM_001110492 /// XM_001055735 /// XM_001055792 /// XM_001057632 /// XM_575385	4q21	1.51	0.024
1379322_at	proteasome (prosome, macropain) 26S subunit, non-ATPase, 7 (predicted)	Psmd7_predicted	2351	1557	NM_001107426 /// XM_001076658 /// XM_226439	19q12	1.51	0.005
1368199_at	nucleoporin 88	Nup88	3556	2354	NM_053616	10q24	1.51	0.007
1367502_at	mitochondrial ribosomal protein L21 (predicted)	Mrpl21_predicted	2159	1430	NM_001107567 /// XM_001067014 /// XM_219576	1q42	1.51	0.000
1373200_at	eukaryotic translation elongation factor 1 epsilon 1 (predicted) /// similar to eukaryotic translation elongation factor 1 epsilon 1 (predicted)	Eef1e1_predicted /// RGD1564159_predicted	5548	3676	NM_001106106 /// XM_001063564 /// XM_214451 /// XR_007759	17p12 /// 4q42	1.51	0.020
1392678_a_at	DNA methyltransferase 3A	Dnmt3a	1322	876	NM_001003957 /// NM_001003958	6q14	1.51	0.001
1390572_at	THO complex 3 (predicted)	Thoc3_predicted	811	537	NM_001106059 /// XM_001068440 /// XM_237957	17p14	1.51	0.008
1371726_at	Transcribed locus	---	1629	1080	AW433870	---	1.51	0.028
1378080_at	similar to SCO cytochrome oxidase deficient homolog 1 (yeast) (predicted)	RGD1559538_predicted	1796	1191	XM_001078805 /// XM_573117	10q24	1.51	0.001
1399131_at	similar to ribosomal protein, mitochondrial, S22	LOC683519	1309	868	NM_001077530 /// XM_001066036	---	1.51	0.001
1370908_at	histone deacetylase 2	Hdac2	4025	2669	XM_001061582 /// XM_342149	20q12	1.51	0.008
1367484_at	ubiquitin-conjugating enzyme E2E 2 (UBC4/5 homolog, yeast)	Ube2e2	3804	2522	NM_001108371 /// XM_001067024 /// XM_341288	15p16	1.51	0.022
1374848_at	Similar to RIKEN cDNA 2410016F19 (predicted)	RGD1307434_predicted	1238	821	NM_001108537 /// XM_001063347 /// XM_342153	20q12	1.51	0.001
1371999_at	Transcribed locus	---	1126	747	BI303641	3	1.51	0.021
1398858_at	proteasome (prosome, macropain) 26S subunit, non-ATPase, 2	Psmd2	6379	4235	NM_001031639	11q23	1.51	0.000
1371987_at	polymerase (DNA directed) sigma (predicted)	Pols_predicted	1107	735	NM_001107333 /// XM_001062703 /// XM_225072	17p14	1.51	0.001
1371557_at	THAP domain containing 4	Thap4	2077	1379	NM_001005564	9q36	1.51	0.002
1388441_at	hypothetical protein LOC689574	LOC689574	1381	918	XM_001068230 /// XM_001071240	4q22	1.51	0.011
1372581_at	SNF8, ESCRT-II complex subunit, homolog (S. cerevisiae)	Snf8	1608	1068	NM_001007804	10q31	1.50	0.005
1372665_at	phosphoserine aminotransferase 1	Psat1	10633	7071	NM_198738	1q43	1.50	0.006
1388428_at	histidyl tRNA synthetase 2 (predicted)	Hars2_predicted	1933	1285	NM_001108594 /// XM_001054635 /// XM_342532	3q41	1.50	0.000
1399142_at	Transcribed locus	---	1780	1184	BF398045	3	1.50	0.002
1388570_at	similar to RIKEN cDNA 2310005O14	LOC498909	1881	1252	NM_001035257	19p12	1.50	0.014

Table S2. Trophoblast stem-associated genes

Affymetrix ID	Gene Title	Gene Symbol	Stem Ave	Dif Ave	GenBank	romosomal Locat	Stem/ Di	t-test
1384131_at	ADP-ribosylation factor-like 6 interacting protein 2	Arl6ip2	2180	1450	XM_001063138 /// XM_216629	6q11	1.50	0.015
1388668_at	Transcribed locus	---	3237	2155	BM384972	---	1.50	0.002
1398916_at	aurora kinase A interacting protein 1	Aurkaip1	2502	1666	NM_001004237	5q36	1.50	0.015
1373394_at	similar to RIKEN cDNA 4122402O22	RGD1310674	1218	812	XM_001058775 /// XM_001063553	11q23	1.50	0.005
1374806_at	similar to 14-3-3 protein sigma /// stratifin (predicted)	LOC298795 /// Sfn_predicted	6985	4656	NM_001013941 /// XM_001065279 /// XM_001065560 /// XM_232745	5q36 /// 6q13	1.50	0.013
