# Supplementary material for: Phosphatidylinositol 3 kinase modulation of trophoblast cell differentiation
Source: BMC Dev Biol. 2010 Sep 14;10:97. doi: 10.1186/1471-213X-10-97 (PMC2944162; doi:10.1186/1471-213X-10-97)
Supplement: Additional file 3 — Table S3: Gene functions. [file 1471-213X-10-97-S3.PDF]

Table S3. Gene functions

Function	Group	Genes
Cell Cycle	Trophoblast stem cell-associated	BMP4, PIAS2, MAPK3, NOLC1, CCNB2, DSN1, BAZ1A, HRAS, HES1, GPSM2, RECQL, CHAF1A, CD2AP, BMI1, CSE1L, SMARCB1, CDKN3, POLR3D, TP53, CALM3, CDCA8, ESPL1, PCGF6, CKS2 (includes EG:1164), ZC3HC1, HIRA, HMGA1, FGFR2, CKAP2 (includes EG:26586), FOXG1, RAE1, SKP2, CCDC5, PCNA, CCND3, HELLS, TARDBP, H2AFX, EHMT2, ANAPC5, TFRC, EBNA1BP2, SGOL1, BRE, XRCC5, UBE2C, KIF20A, NCAPD3, CD320, AATF, AKAP8, CDT1, SUV39H2, KPNA2, DKC1, GAPDH (includes EG:2597), DTD1, NUF2, CDX1, CHEK1, PTMA, ID1, PSMG2, PROS1, DAP3, TOP2A, E2F5, MCM10, KIF2C, EGFR, CDC45L, MYCN, CCNB1, DBI, NAE1, CDK2AP1, PSMD13, SPC25, TIAL1, PLK1, NCAPD2, POLD1, ID3, FZR1, CEACAM1, KPNB1, KIF22, BUB1 (includes EG:699), MAD2L1, GPI, CCNE1, MCM2, TLK1, MSH2, CUL2, NDE1, NOL1, MPHOSPH1, CHFR, CCT7, EDG1, PRMT5, NHP2L1, PSAP, PIN1, SFN, LIG1, KIF23, DLG7, SFRS2, SRPK1, CCNI, MLLT6, PTTG1, PARP2, SMC4, UBE2V2, NUDC, TAF10, KNTC1, PCBP4, AURKB, SCYE1, BIRC5, GSPT1, MAP2K2, MYBL2, CAST, ECT2, NUCKS1, KIFC1, MCM5, SMC3, MIF, PSMB5, EWSR1, HDAC2, NUBP1, MTA2, ORC6L, LMNA, PAXIP1, ZRF1, BUB1B, SSRP1, NCOA3, ERCC6L, PCAF, NASP, SMC2, CDK4, HIRIP3, CAPN2, GNL3, DIABLO, TMPO, POLE3, BUB3, ID2, PA2G4, CDC20, DTYMK, PTPRF, SHC1, CCNA2, ASPM, GMNN, DR1, BLM, STAT1, TERF1, TACC3, NUSAP1, UHRF1, KLHL2, RACGAP1, PRC1, CKAP5, TBRG4, PPM1G, NCAPH2, JOSD3, CKS1B, PNPT1, PPAP2C, MCM7, DDX11
	Differentiation-associated	MAGED1, CAMK1, CREM, HBP1, SOD2, TCEB3, TSC2, FOSL1, VCPIP1, IRS3, SNAI1, JUNB, STAT3, IER3, CSH1, ATF2, DAXX, HELLS, JDP2, FOXN3, BTG2, CDC2L6, MORC3, ADM, FN1, DDIT3, ILK, ZNF655, CRK, DMTF1, NOTCH2, JUN, HK2, GADD45A, PCTK2, PDGFRA, RB1CC1, CITED2, ITGB1, GRB2, EGR1, XBP1, PPP1R15A (includes EG:23645), CEACAM1, GRN, FIS1, GNAI3, LYN, APBB2, PLAU, NCOR2, CSH2, PSEN1, HSPA1A, CUL4A, KLF6, C13ORF15, PIP5K3, GATA2, PPM1D, MYC, USP8, TOP1, CAMK2D, PLAC1, RHOB, EZH2, ITGAV, CDKN1C, ZMYM2, FGFR1, PAWR, BCAR3, KLF4, SEL1L, BCL2L1, IGF2, CCNG2, MDM4, MNT, DUSP1, CEBPD, CASP2, MDK, SOCS5, VHL, JARID1B, ATF5, RBBP8, HIF1A, ING4, NFKBIA, CLK1, SP1, FLNA (includes EG:2316), PLK2, ANXA1, SPHK1, ARID3A, PBEF1, PPARD, PLAUR, CEBPB, IVNS1ABP, GNAI2, PIK3CB, CDKN1B, ING1, MMP9, LGALS1
	Positively regulated by PI3K	AKAP12, CUL5, CREM, DDIT3, BAZ1A, CDC123, SCIN, AURKA, CSH1, MYC, BCL2L1, DAXX, HMOX1, IGF2, SREBF1, GATA3, CSH2, MMP9
	Negatively regulated by PI3K	AMACR, TPM1, ATF3, ING5, HSPA1A, EGR1, TAF10, LATS2, CYP26B1, CD2AP, H2AFX, SPRY2, PRMT5, SPHK1, UBE2D3, PRKCH, CAST, TCF7L2, ID4, PPP3CA
Cell Survival	Trophoblast stem cell-associated	LIG1, NPM1 (includes EG:18148), PRPF19, PIAS2, BMP4, DNMT3A, MSX1, TRAP1, HRAS, HES1, BIRC5, BMI1, TP53, TNFRSF21, PRKRA, HDAC2, RRM2, HMGA1, PAXIP1, LMNA, BUB1B, PCNA, CCND3, DIABLO, XRCC5, PRDX2, S100A6, ID2, NME1, SMN1, EMILIN2, CDC20, NUF2, PTMA, CHEK1, ID1, SHC1, MT1E, HMGB1 (includes EG:25459), MCM10, PDK1, BLM, EGFR, DHX9, MYCN, RBM17, TACC3, CCNB1, UCP2, UHRF1, SPC25, PLK1, MAD2L1, CCNE1, TPM3, MSH2, CHFR, COX5A, EDG1, SFN, TXNDC17
	Differentiation-associated	MAGED1, TGFBR1, CREM, MAP3K7IP2, GCLC, TEGT, ODC1, C16ORF5, MAML1, POR, PKN2, NSF, SOD2, FHL2, TSC2, VAMP3, ADA, ATF4, FOSL1, SERPINE1, DNM2, DDIT4, CSH1, STAT3, ATF2, DAXX, CDH5, JDP2, HELLS, BTG2, CTSB, ZYX, SQSTM1, HTATIP2, ARMC10, HSPA1B, DDIT3, ILK, HSPB8, GLIPR1, TGM2, CASP6, APPL1, NOTCH2, CD47, GADD45A, BBC3, ZFAND5, RNF19A, NFE2L2, B4GALT5, TMSB4X, OPTN, GRB2, TNFRSF1A, ABCG2, TRIB3, SLC25A24, GRN, LDLR, BFAR, LYN, HAP1, PSEN1, PINK1, DAD1, JAK1, KLF6, BAG3, GATA2, CEBPG, PDCD7, PPM1D, MYC, FDFT1, CTSD, UBE2B, RHOB, SNN, PRR13, CDKN1C, KLF11, STK17B, ATG12, STXBP1, IL2RG, ZMYM2, FEM1B, RDX, BTG1, KLF4, BCL2L1, IGF2, MDM4, SERINC3, GCNT1, DUSP1, SGPP1, MDK, IRF8, HFE, VHL, HSPB1, B2M, KLF10, MAP2K1IP1, NFAT5, PBEF1, CD24, PXN, SLC12A2, G6PD, NSD1, HERPUD1, PLAUR, ALS2, NFE2L1, IVNS1ABP, PRKCI, NRTN, CDKN1B, ING1, CTSL2, A4GALT, BCL2L2, SMG1, IRS3, FASLG, PEG3 (includes EG:5178), TNKS, JUNB, IER3, LDOC1, CD9, RTN4, CDC2L6, ADM, CA2, TCF4, FN1, ZFP36, CRK, PSENEN, NFIL3, JUN, HK2, TFAP2A, RB1CC1, PDGFRA, ACSL4, MAP1LC3B, CITED2, ITGB1, EGR1, ELL, XBP1, ANXA4, PPP1R15A (includes EG:23645), F3, RABGEF1, CEACAM1, ZNF148, ITGB2, FIS1, KRT8, DHCR24, APBB2, PLAU, NCOR2, SLC31A1, NCOA6, CLN8, HSPA1A, TICAM2, CUL4A, MAP1B, TCF7, TOP1, CAMK2D, SCARB1, DPP3, ITGAV, JUP, DSP, PTPRE, FGFR1, PAWR, RALBP1, STAMBP, EIF2AK4, ARRB2, MNT,
	Positively regulated by PI3K	ADM, CA2, HTATIP2, TCF4, CUL5, CREM, DDIT3, ATF5, GADD45G, HSPB8, GCLC, GLIPR1, MYC, CTSD, HMOX1, HSP90B1, GCLM, FASLG, PLSCR1, DDIT4, SLC2A1, HSPA9, HERPUD1, NSD1, TRIB3, SCIN, AURKA, CSH1, HCLS1, LDOC1, CEACAM1, BCL2L1, DAXX, IGF2, YARS, RIPK3, CYB5A, DNAJB6, SQSTM1, MMP9, HSPB1
	Negatively regulated by PI3K	TPM1, S100A6, HSPA1B, ING5, SGK1, HSPA1A, DUSP22, TAF10, LATS2, TNFRSF12A, NCK2, FDFT1, CYP26B1, CD2AP, PPP3CB, CLDN4, STUB1, A4GALT, SPHK1, CYB5R3, PRR13, DAG1, CAST, DNAJB1, DUSP14, SERPINE1, PPM2C, KLF2, PPP3CA, S100A10, PRNP, STXBP1, ATF3, EGR1, PERP (includes EG:64065), ITGA6, PSIP1, AKAP13, MBTPS1, BNIP3L, PRKCH, ZFP36L2, HFE, CLDN3, ID4
Cell Signaling	Trophoblast stem cell-associated	

Table S3. Gene functions

Function	Group	Genes
Cell Signaling (cont)	Differentiation-associated	LITAF, JAK1, ZFP36, TICAM2, CRK, AKAP11, TGM2, GNB4, MAP2K1IP1, NLK, JUN, GADD45A, FLNA (includes EG:2316), TNIP2, PLK2, ITGAV, RB1CC1, MAPKAPK2, CD24, TMEM9B, HGS, GNG12, STK17B, ITGB1, GRB2, FGFR1, DAPK3, MKNK2, STAT3, STAMBP, ATF2, GNAI2, DAXX, STK38, NRTN, NDFIP1, WNK1, PIK3CB, STAT2, DOK1, MAP4K5, SQSTM1, SOCS5, ECM1, PINK1, IRAK2, LGALS1
	Positively regulated by PI3K	
	Negatively regulated by PI3K	CD2AP, SLC9A3R1, DAG1, PARD3, HFE
Cell-To-Cell Signaling and Interaction	Trophoblast stem cell-associated	LAMA5, PHB2, TFRC, L1CAM, HES1, S100A10
	Differentiation-associated	NCOA6, MYC, PKN2, CTSL2, ESAM, FHL2, SOD2, RHOB, SCARB1, TSC2, ITGAV, JUP, MGAT1, SERPINE1, LCP1, IL2RG, FERMT2, STAT3, IGF2, CD9, CDH5, ITGA1, IRF8, TFPI, C1GALT1, VHL, SPN, FN1, PVRL3, PTPN14, ILK, UGCG, CRK, PTPN12, TGM2, CD47, ANXA1, STX4, CD24, ITGB1, CD59, EGR1, PLAUR, ITGA3, F3, ITGB2, ADAM10, LYN, CALD1, PIK3CB, PLAU, ITGAX
	Positively regulated by PI3K	ADM, CEACAM1, MYC, HMOX1, BCL2L1, IGF2, HSP90B1, PBX3, ENTPD2, PRL4A1, GATA3, MMP9, FASLG
	Negatively regulated by PI3K	NCK2, F11R, STXBP1, HSPA1A, PIP5K1C, SPRY2, EGR1, ITGA6, TACSTD1, CAST, SERPINE1
Cellular Assembly and Organization	Trophoblast stem cell-associated	KIF23, DLG7, SRPK1, PTTG1, PARP2, SMC4, TXNL4A, CCNB2, DSN1, BAZ1A, GPSM2, AURKB, BIRC5, CHAF1A, CKB, SNRPD1, ECT2, KIFC1, TP53, PPID, ESPL1, NUBP1, MTA2, CKAP2 (includes EG:26586), BUB1B, ERCC6L, SKP2, RAE1, PRDX3, TFAM, SMC2, HELLS, H2AFX, EHMT2, HMGN2, EBNA1BP2, CAPN2, HIRIP3, SGOL1, XRCC5, BUB3, POLE3, SMN1, NCAPD3, CDT1, UBE2N, SUV39H2, CKMT1B, NUF2, SLC25A5, USP39, ID1, CCNA2, GMNN, DR1, TOP2A, KIF2C, BLM, TERF1, MYCN, NUSAP1, TACC3, CCNB1, SLC25A4, UHRF1, MRPL15, PRC1, SPC25, CKAP5, PLK1, MRPL17, NCAPD2, NCAPH2, KIF22, KPNB1, BUB1 (includes EG:699), MAD2L1, CCNE1, B4GALT1, TLK1, NDE1, NHP2L1, PIN1, SFN, KIF18A, DDX11
	Differentiation-associated	EPS15, TOM1, RABEP1, MAP1B, PIP5K3, LIMK2, MYC, NSF, SOD2, RHOB, BAIAP2, ITGAV, FASLG, DSP, PTP4A3, RDX, CD9, CDH5, SQSTM1, ELMO1, CTTN, VHL, ENAH, FN1, PICALM, PTPN14, UGCG, CRK, REPS1, PTPN12, FLNA (includes EG:2316), TINAGL1, ARHGEF2, GOSR1, CD24, HGS, ITGB1, STX12, PXN, LOC290704, RAB5A, GRB2, DDEF1, STX5, PLAUR, ALS2, RABGEF1, ITGB2, EPB41, WIPF1, PRKCI, SYNJ1, STX7, HAP1, CALD1, PLAU, CDKN1B, MSN
	Positively regulated by PI3K	ADM, MYC, BCL2L1, IGF2, GCLC, BAZ1A, AURKA, GCLM, GATA3, FASLG, PLSCR1
	Negatively regulated by PI3K	FLNB, TPM1, MYH10, HSPA1B, HSPA1A, CSRP1, TNFRSF12A, NCK2, DHCR7, CD2AP, A4GALT, STMN4, BAIAP2, CYB5R3, SPHK1, PLDN, CAST, DAG1, SERPINE1, KLF2, PPP3CA, LASP1, STXBP1, ATF3, ARHGEF12, DDEF1, ITGA6, AKAP13, CORO1C, CHRAC1, PIP5K1C, H2AFX, SPRY2, HMGN2, PARD3
Cellular Growth and Proliferation	Trophoblast stem cell-associated	NPM1 (includes EG:18148), DBN1, PIAS2, HMMR, PLK4, ELF3, SMARCB1, TRAF4, RUVBL2, MYBBP1A, AHCY, SART3, TNFRSF21, TP53, ESPL1, CDCA8, HMGA1, GFM1, RRM2, FGFR2, ANP32A, SKP2, NDNL2, ANAPC5, TFRC, DHFR, HNRPAB, XRCC5, UBE2C, KIF20A, NME1, AATF, CD320, PHLDA2, GADD45GIP1, TMEFF1, CLDN4, BCCIP, HNRNPU, THG1L, UCP2, CDK2AP1, LASS1, WNK2, CDCA7, HNRPD, ID3, MCM3, MAD2L1, CCNE1, ENO1, CCT7, PSAP, SHMT1, PIN1, SFN, CXADR, DLG7, SFRS2, ILF3, MLLT6, PTTG1, SF3B2, GNPAT, CCT2, NME2, SCYE1, GGA2, SAE1, MAP2K2, MYBL2, CAST, EIF5A2, PGK1, SMC3, HNRPF, LAPTM4B, EWSR1, HDAC2, CDX2, BAMBI, PFDN5, ZRF1, NCOA3, TACSTD1, DIABLO, PPAT, TMEPAI, ID2, HDGF, SHC1, CCNA2, STUB1, STAT1, TERF1, LAMA5, SLC9A3R1, ADAM15, HSPD1 (includes EG:3329), MTHFD1, PPM1G, ATIC, JOSD3, PSMD2, CKS1B, EIF3I, BMP4, MAPK3, TRAP1, HRAS, HES1, GSS, SET, EEF1D, OCLN, ACIN1, BNIP3 (includes EG:664), BMI1, NFKBIB, NOL8, ITGB5, CALM3, METTL3, TNK2, FOXG1, CCND3, PSMB2, CCT3, SPINT2, LDHA, EMILIN2, CDT1, DUSP6, GPX1, RUVBL1, SYMPK, EIF4G1, CDX1, SF3B3, EIF2B2, CHEK1, BYSL, PTMA, ID1, MT1E, TCOF1 (includes EG:6949), DAP3, EGFR, MYCN, CCNB1, TRIM28, DDX56, TIAL1, RRM1, FOLR1, CEACAM1, MCM2, NDE1, MPHOSPH1, CHFR, RSL1D1, EDG1, FKBP4, C6ORF108, PRPF19, CCNI, PCBP4, PBK, CD63, PSMC5, BIRC5, USP7, SIVA1, AKT1S1, MCM5, MIF, ATP5A1, MTA2, PAXIP1, LMNA, HNRPM, EI24, SFRS3, PCAF, CDK4, AK2,

Table S3. Gene functions

Function	Group	Genes
Cellular Growth and Proliferation (cont)	Differentiation-associated	MAGED1, TGFBR1, CREM, RAB2A, MAP3K7IP2, ODC1, MAML1, LAMC1, POR, SOD2, FHL2, TSC2, ADA, ATF4, FOSL1, SERPINE1, MGAT1, DNM2, LCP1, FLOT1, STAT3, CSH1, ATF2, CDC73, DAXX, CDH5, BTG2, CTSB, ZYX, ITGA1, DOK1, ECM1, ARMC10, PIM3, DDIT3, PTPN14, SEMA6A, ILK, HSPB8, TGM2, APPL1, DMTF1, HADHB, CD47, NOTCH2, GADD45A, BBC3, TMSB4X, OPTN, ENTPD1, TNFRSF1A, GRB2, GRN, WIPF1, TMEM49, LYN, NRIP1, RNF14, CSH2, PSEN1, JAK1, ARL6IP5, KLF6, GATA2, PPM1D, GPNMB, HDAC6, MYC, CTNNBIP1, CTSD, FDFT1, USP8, UBE2B, ANXA11, RHOB, CDKN1C, STK17B, KLF11, TOP3B, IL2RG, ZMYM2, BTG1, KLF4, PRL7A2, SLC3A2, BCL2L1, MDM4, IGF2, SERINC3, DUSP1, GCNT1, IRF8, MDK, TFPI, S100A11 (includes EG:6282), VHL, LITAF, YPEL3, KLF10, DHCR7, NFAT5, ARID3A, PBEF1, CD24, G6PD, ERO1L, PLAUR, IVNS1ABP, SHB, PRKCI, ING1, CDKN1B, ITGAX, TCIRG1, HBP1, CTSL2, TCEB3, BCL2L2, FASLG, TNKS, PTP4A3, USP9X, JUNB, IER3, BMYC, LDOC1, CD9, CDC2L6, RAP1B, ADM, TCF4, RALA, FN1, B3GNT2, CRK, DNAJA1, HK2, JUN, TFAP2A, PDGFRA, RB1CC1, HGS, CITED2, BMP1, GLMN, ITGB1, RGS2, EGR1, ELL, XBP1, PFN2, PPP1R15A (includes EG:23645), NAP1L1, ITGA3, RABGEF1, ZNF148, CEACAM1, CUX1, FIS1, ITGB2, NUB1, KRT8, DHCR24, SREBF1, ADAM10, APBB2, NCOR2, PLAU, EPS15, NCOA6, INSIG1, CUL4A, CDA, TCF7, TOP1, PLAC1, SCARB1, EZH2, ITGAV, UBE2E3, JUP, DSP, SOX4, PTPRE, FGFR1, TSNAX, PAWR, BCAR3, RALBP1, STAMBP, DDX17, ELF1,
	Positively regulated by PI3K	AKAP12, ADM, CREM, DDIT3, GADD45G, HSPB8, DNAJC3, MYC, CTSD, HMOX1, FASLG, PLSCR1, LCP1, TSNAX, CSH1, AURKA, ATIC, SLC3A2, CEACAM1, BCL2L1, IGF2, FABP5, SREBF1, DNAJB6, CSH2, MMP9
	Negatively regulated by PI3K	GBX2, FLNB, MYH10, S100A6, ING5, HSPA1A, DUSP22, IGBP1, TNFRSF12A, NCK2, FDFT1, DHCR7, CLDN4, STUB1, SPHK1, UBE2D3, SERPINE1, IRX3 (includes EG:79191), KLF2, PPP3CA, S100A10, PRNP, AMACR, ATF3, EGR1, ITGA6, F11R, AKAP13, SPRY2, PRMT5, BNIP3L, TACSTD1, ZFP36L2, HMGCR, ID4
Cellular Movement	Trophoblast stem cell-associated	KIF23, TP53, KIF20A, NUSAP1, CCNB1, CDC20, RACGAP1, PRC1, HRAS, PLK1, AURKB, ID3, ID1, CD2AP, ELF3, MAP2K2, MPHOSPH1, CHFR, TOP2A, ECT2, EGFR
	Differentiation-associated	TGFBR1, TCIRG1, HBP1, ODC1, LAMC1, CTSL2, LGMN, SOD2, FHL2, A4GALT, TSC2, CBLL1, FOSL1, SERPINE1, GNG12, DNM2, FASLG, LCP1, PTP4A3, CREB3, FERMT2, SNAI1, JUNB, STAT3, CSH1, ARHGDIB, CDH5, CD9, RTN4, CTSB, ZYX, ITGA1, DOK1, ADM, ENAH, FLNB, RALA, FN1, PTPN14, ILK, TPM4, CRK, DNAJA1, PTPN12, TGM2, PRKX, CD47, ACTR3, JUN, GADD45A, PPAP2B, PDGFRA, SARS, NFE2L2, B4GALT5, TMSB4X, ITGB1, TNFRSF1A, EGR1, ITGA3, F3, GRN, CEACAM1, GNAI3, ITGB2, PEX5, LDLR, SREBF1, ADAM10, LYN, APBB2, PLAU, PSEN1, JAK1, EPS15, MAP1B, HEBP1, HDAC6, MYC, CTSD, ESAM, RHOB, PLAC1, EZH2, ITGAV, JUP, FXYD5, FGFR1, BCAR3, TMOD3, RALBP1, KLF4, SLC3A2, SEL1L, IGF2, ARRB2, PREX1, GCNT1, CXCR7, DUSP1, SEMA6D, SGPP1, MDK, TFPI, CTTN, ELMO1, VHL, HSPB1, PAFAH1B2, SPN, PICALM, ING4, HIF1A, DNMBP, PDCD4, NFKBIA, NFAT5, SP1, FLNA (includes EG:2316), ANXA1, SPHK1, CCRL1, MAPKAPK2, CD24, PRNP, CD59, PXN, DDEF1, PPARD, DAPK3, G6PD, PLAUR, GNAI2, PRKCI, NRTN, SYNJ1, MYLIP, CALD1, PIK3CB, CDKN1B, HMGCR, MMP9, MSN, LGALS1, ITGAX
	Positively regulated by PI3K	ADM, LCP1, CUL5, AURKA, CSH1, SLC3A2, CEACAM1, MYC, CTSD, ESAM, NARS, WARS, IGF2, FABP5, YARS, SEMA6D, PPAP2B, GATA3, SARS, ELMO1, FASLG, MMP9
	Negatively regulated by PI3K	GBX2, MYH10, FLNB, TPM1, S100A6, IGBP1, LATS2, TNFRSF12A, NCK2, CD2AP, CLDN4, A4GALT, SPHK1, CAST, SERPINE1, KLF2, LASP1, S100A10, PRNP, ATF3, SLC9A3R1, DDEF1, EGR1, APLP2, ITGA6, F11R, PIP5K1C, MRLC2, SPRY2, HMGCR, CLDN3
Gene Expression	Trophoblast stem cell-associated	POLR2F, PRIM1, PIAS2, BMP4, MAPK3, MSX1, SMARCD2, MED21, BAZ1A, HRAS, SFPQ, HES1, PPP4C, SET, EEF1D, CHAF1A, ELF3, BMI1, GATAD2A, SMARCB1, TCERG1, MYBBP1A, NFKBIB, APEX1, TARBP2, HMX1, TP53, BCOR, PCGF6, NUFIP1, HIRA, HMGA1, MAPK8IP1, ANP32A, NDNL2, ZNF238, CCND3, SUPT16H, TARDBP, EHMT2, HMGN2, SLC44A2, POLR2I, HNRPAB, XRCC5, HMGB2, DEK, NME1, PRDX1, KPNA2, PSMD9, RUVBL1, MED28, CDX1, PTMA, VRK1, NARG1, ID1, RNPS1, TRIM27, TCOF1 (includes EG:6949), CTBP2, E2F5, TOP2A, POLR3G, PSMD14, HMGB1 (includes EG:25459), ABT1, EGFR, ORC1L, MYCN, CCNB1, NAE1, PPP1R8, PRIM2, TRIM28, MRPL12, TIAL1, PLK1, ID3, CCNE1, ZNF593, ENO1, EDG1, PIN1, MED4, GTF3A, TRAF3, ILF3, GTF3C6, MLLT6, DNMT3A, PTTG1, HSF2, NME2, CBX3, PSMC5, RDBP, TCEB2, PTGES2, PPIE, ECT2, MIF, EWSR1, HDAC2, PHF5A, CDX2, MTA2, PHB2, PFDN5, LMNA, BRD8, TBL1X, PUS1, SSRP1, NCOA3, IFRD1, PCAF, TFAM, TADA2L, NSEP1, PABPN1, TMPO, PEBP1, POU3F1, SNF8, ID2, PA2G4, GTF2F2, PDGFA, BCLAF1, CCNA2, POLR1C, SHC1, POLR2C, DNTTIP1, STUB1, NEDD8, DR1, SUDS3, STAT1, TCF20, SLC20A1, TRIP13, CCNH, VEZF1, UHRF1, CSDA, PSMC3IP, STRAP, CENPK, POLR2G, NOC2L, ZNHIT3, KLF5, SATB1, PNPT1, SMARCC1, MCM7

Table S3. Gene functions

Function	Group	Genes
Gene Expression (cont)	Differentiation-associated	MED13, MAGED1, CREM, TGFBR1, MAP3K7IP2, GCLC, GTF2E2, HBP1, ATP8B1, PKN2, MAML1, MYCBP2, FHL2, SOD2, TSC2, TCEB3, ATF4, FOSL1, SERPINE1, FASLG, JUNB, CSH1, STAT3, ZNF496, ATF2, DAXX, JDP2, CCNDBP1, FOXN3, BTG2, SQSTM1, ECM1, ADM, TCF4, HTATIP2, SCAP, FN1, DDIT3, ZFP36, ZNF143, ILK, CRK, AFF4, SMURF1, ARID4B, APPL1, DMTF1, NFIL3, NOTCH2, JUN, ACTR3, GADD45A, TFAP2A, HGS, NFE2L2, CITED2, GLMN, ITGB1, GRB2, TNFRSF1A, EGR1, ELL, XBP1, TRIB3, NAP1L1, NOSTRIN, GRN, ZNF148, CUX1, WIPF1, GPBP1, SREBF2, SREBF1, ATF7IP, LYN, UBN1, AEBP2, APBB2, NCOR2, NRIP1, RNF14, STAG2, PSEN1, JAK1, NCOA6, TICAM2, JMJD1C, CUL4A, KLF6, HAND1, UIMC1, LRRFIP2, GATA2, TCF7, CEBPG, PPM1D, MYC, CTNNBIP1, TOP1, TCF25, CAMK2D, RHOB, EZH2, ITGAV, JUP, CDKN1C, KLF11, EID1, FGFR1, BTG1, PAWR, RALBP1, STAMBP, DDX17, ELF1, EPN2, BCL2L1, MDM4, IGF2, MNT, DUSP1, CEBPD, GMCL1, TLE3, PDLIM1, STAT2, LCOR, IRF8, VHL, JARID1B, LITAF, DYNLT3, ATF5, KLF10, RBBP8, MEIS2, HIF1A, ING4, HIC1, PDCD4, NFX1, MAP2K1IP1, NFKBIA, NFAT5, SP1, MED15, FLNA (includes EG:2316), PLK2, ARID3A, MAPKAPK2, BLZF1, ARID5B, PXN, TAF6, MSL3L1, PPARD, NSD1, CIR (includes EG:9541), CEBPB, BIRC4, NFE2L1, IVNS1ABP, HTATSF1, RCOR1, ERBB2IP, PRKCI, WNK1, CDKN1B, ING1, LGALS1
	Positively regulated by PI3K	ADM, AKAP12, HTATIP2, TCF4, CREM, DDIT3, GADD45G, ATF5, BAZ1A, GCLC, ATP8B1, MYC, HSP90B1, GRINL1A, ATF4, FASLG, MED20, PCGF6, NSD1, TRIB3, CSH1, HCLS1, DAXX, BCL2L1, IGF2, FABP5, SREBF1, CYB5A, DNAJB6, SQSTM1, GATA3, ELF5
	Negatively regulated by PI3K	AKAP13, ARHGEF12, ATF3, EGR1, ITGA6, PRKCH, DNAJB1, PARD3, KLF2, TCF7L2, PRNP
Lipid Metabolism	Trophoblast stem cell-associated	PRDX3, GPX1, APEX1
	Differentiation-associated	HSD3B1, SCAP, CLN8, IDI1, INSIG1, CYP17A1, FDFT1, DHCR7, CD47, SCARB1, SMG1, PBEF1, CD24, CYP51A1, PLSCR1, CYP11A1, ITGB1, APOB48R, ABHD5, RAB5A, PPARD, STARD3, CEBPB, FDPS, LDLR, SYNJ1, DHCR24, SREBF2, SREBF1, PIK3CB, SLC27A1, DGAT1, HMGCR, LGALS1
	Positively regulated by PI3K	ADM, CYP17A1, BCL2L1, FABP5, CYB5A, CSH1, CSH2, HSD17B2, FASLG, PLSCR1
	Negatively regulated by PI3K	AMACR, SQLE, ACAT2, DGAT2, FDFT1, CYP26B1, DHCR7, A4GALT, MBTPS1, PCYT2, SPHK1, SERPINE1, HMGCR, MOGAT2, CYP51A1, PRNP
Post-Translational Modification	Trophoblast stem cell-associated	HSPBP1, DNAJA4, SCO1, SF3B3, OCLN, CHAF1A, CD2AP, TCEB2, STUB1, TCP1, RUVBL2, DAG1, VAPB, TBCD, FKBP5, PPID, TP53, BCS1L, MKKS, TBCA, SLC9A3R1, HMGA1, CCT6A, ERP29, FKBP4, CCT7, CCT3, HFE
	Differentiation-associated	RNF139, TGFBR1, JAK1, DPM1, PIP5K3, PPM1D, MYC, HDAC6, SOD2, CAMK2D, SCARB1, EZH2, TSC2, CBLL1, ITGAV, SMG1, ATG12, PTPRE, TNKS, FGFR1, ELF1, SLC3A2, BCL2L1, ARRB2, WHSC1L1, CD9, DUSP1, RNF25, UBE2G1, BACE2, TLK2 (includes EG:11011), VHL, ADM, PIM3, SPN, FN1, PTPN14, ILK, PSENEN, CRK, ING4, DNAJA1, BAG2, USP2, PTPN12, UBE4A, SMURF1, HK1, CD47, NLK, CLK1, PPP3CB, RB1CC1, CD24, PLOD3, MTMR3, ITGB1, PPARD, ERO1L, NSD1, CLPX, USP33, BIRC4, SHB, ITGB2, STK38, CMAS, MYLIP, LYN, PSEN1, IRAK2, LGALS1
	Positively regulated by PI3K	
	Negatively regulated by PI3K	SLC9A3R1, APLP2, ITGA6, DUSP22, NCK2, CD2AP, PPP3CB, STUB1, PCMT1, SPHK1, UBE2D3, DAG1, DNAJB1, PPM2C, PARD3, PPP3CA, HFE
