# Supplementary material for: Phosphatidylinositol 3 kinase modulation of trophoblast cell differentiation
Source: BMC Dev Biol. 2010 Sep 14;10:97. doi: 10.1186/1471-213X-10-97 (PMC2944162; doi:10.1186/1471-213X-10-97)
Supplement: Additional file 4 — Table S4: Trophoblast differentiation-associated genes [file 1471-213X-10-97-S4.PDF]

Table S4. Trophoblast differentiation-associated genes

Affymetrix ID	Gene Title	Gene Symbol	Stem Ave	Dif Ave	GenBank	Chromosomal Location	dif/ stem	t-test
1390511_at	LOC308394	Cgm4	10	1863	BI285801	1	193.51	0.006
1378534_at	similar to brain carcinoembryonic antigen	LOC308394	10	1746	NM_001025679	1q21	183.10	0.001
1388433_at	keratin complex 1, acidic, gene 19	Krt1-19	53	7958	NM_199498	10q32.1	149.07	0.022
1369029_at	phospholipid scramblase 1	Plscr1	20	2050	NM_057194	8q31	101.12	0.028
1389856_at	carcinoembryonic antigen gene family 4	Cgm4	57	5073	NM_012525	1q21	89.73	0.001
1368996_at	carcinoembryonic antigen-related cell adhesion molecule 3	Ceacam3	202	17879	NM_012702	1q21	88.71	0.001
1392832_at	similar to angiopoietin-like 1	LOC684489	17	1398	XM_001068284	---	83.66	0.001
1377666_at	choline dehydrogenase	Chdh	26	1571	NM_198731	16p16	60.59	0.003
1368468_at	cytochrome P450, family 11, subfamily a, polypeptide 1	Cyp11a1	196	9216	NM_017286	8q24	47.11	0.000
1376934_x_at	similar to brain carcinoembryonic antigen	Cgm4	53	2146	BI285801	1	40.22	0.000
1390525_a_at	stimulated by retinoic acid gene 6 homolog (mouse)	Stra6	28	1037	NM_001029924	8q24	37.44	0.001
1382690_at	carcinoembryonic antigen gene family 4	Cgm4	81	2729	NM_012525	1q21	33.86	0.001
1367809_at	prolactin family 4, subfamily a, member 1	Prl4a1	683	22573	NM_017036	17p11	33.05	0.004
1383458_at	calcium channel, voltage-dependent, L type, alpha 1D subunit	Cacna1d	26	802	BF403759	16	30.89	0.001
1370852_at	spleen protein 1 precursor	LOC171573	692	20968	NM_138537	8q21	30.29	0.003
1376036_at	transporter	LOC314323	29	875	NM_199109	6q31	29.86	0.000
1370227_at	prolactin family 8, subfamily a, member 7	Prl8a7	33	937	NM_022537	17p11	28.78	0.000
1374488_at	GRAM domain containing 1B (predicted)	Gramd1b_predicted	89	2514	XM_001059472 /// XM_217113	8q22	28.11	0.003
1380077_at	Transcribed locus	---	191	5186	AI412131	---	27.18	0.000
1376845_at	putative ISG12(b) protein	isg12(b)	158	4110	NM_206846 /// XM_001068101 /// XM_238467	6q32	26.02	0.017
1387180_at	interleukin 1 receptor, type II	Il1r2	81	2075	NM_053953	9q21	25.57	0.003
1385925_at	Transcribed locus	---	262	6573	AA963975	---	25.12	0.001
1387144_at	integrin alpha 1	Itga1	79	1976	NM_030994	2q16-q21	24.98	0.007
1389092_at	interleukin 2 receptor, gamma (severe combined immunodeficiency)	Il2rg	62	1490	NM_080889	Xq31	24.09	0.004
1368227_at	solute carrier family 28 (sodium-coupled nucleoside transporter), member 2	Slc28a2	240	5743	NM_031664	3q35	23.94	0.000
1383765_at	similar to RIKEN cDNA 4921520P21	MGC114388	36	863	NM_001025288	---	23.86	0.000
1377264_at	interleukin 17F	Il17f	179	4036	NM_001015011	9q13	22.49	0.000
1378483_at	prolactin family 7, subfamily b, member 1	Prl7b1	374	8240	NM_153738	17p11	22.01	0.005
1379598_at	adenylate cyclase 7	Adcy7	53	1153	AI071649	19	21.75	0.003
1380768_at	Transcribed locus	---	85	1798	BM389322	---	21.23	0.000
1370234_at	fibronectin 1	Fn1	712	14010	NM_019143	9q33	19.68	0.000
1391756_at	Transcribed locus, strongly similar to XP_344867.2 PREDICTED: similar to carcinoembryonic antigen-related cell adhesion molecule 1 (biliary glycoprotein) [Rattus norvegicus]	---	856	16683	BI277904	1	19.50	0.001
1369182_at	coagulation factor III	F3	46	857	NM_013057	2q41	18.69	0.000
1389394_at	Transcribed locus	---	129	2255	AI411809	---	17.50	0.001
1373841_at	rac/cdc42 guanine nucleotide exchange factor 6	arhgef6	129	2219	AI177431	X	17.23	0.000
1389780_at	tissue factor pathway inhibitor	Tfpi	497	8452	NM_017200	3q24	17.01	0.000
1376958_at	Similar to serine (or cysteine) proteinase inhibitor, clade B, member 9 (predicted)	RGD1562844_predicted	294	4825	XR_008337	17p12	16.40	0.000
1389474_at	myosin regulatory light chain interacting protein (predicted)	Mylip_predicted	64	1016	NM_001107344 /// XM_001059193 /// XM_225220	17p12	15.81	0.001

Table S4. Trophoblast differentiation-associated genes

Affymetrix ID	Gene Title	Gene Symbol	Stem Ave	Dif Ave	GenBank	Chromosomal Location	dif/ stem	t-test
1373900_at	keratin complex 2, basic, gene 7	Krt2-7	334	5219	NM_001047870 /// XM_001064467 /// XM_217035	7q36	15.62	0.001
1384934_at	Solute carrier family 41, member 2 (predicted)	Slc41a2_predicted	87	1354	NM_001108742 /// XM_001079703 /// XM_343191	7q13	15.56	0.000
1388471_at	t-complex 11 (mouse) like 2	Tcp11l2	145	2202	NM_001017458	7q13	15.20	0.000
1368579_at	Prolactin family 2, subfamily a, member 1	Prl2a1	2414	36672	NM_053791	17p11	15.19	0.002
1377912_at	Transcribed locus	---	149	2207	AI013104	---	14.80	0.003
1370471_at	prolactin family 6, subfamily a, member 1	Prl6a1	102	1481	NM_022176 /// XM_001069642 /// XM_341525	17p11	14.55	0.000
1368623_at	CEA-related cell adhesion molecule 9	Ceacam9	618	8892	NM_053919	1q21	14.38	0.000
1376457_at	cysteine-rich secretory protein LCCL domain containing 2	Crispld2	101	1375	NM_138518	19q12	13.59	0.000
1371298_at	H19 fetal liver mRNA	H19	329	4313	BF284168	1	13.10	0.026
1378400_at	similar to RIKEN cDNA 4632404H22 (predicted)	RGD1560129_predicted	292	3703	XM_001054191 /// XM_577043	Xq36	12.69	0.000
1391693_at	ATPase, Class I, type 8B, member 1 (predicted)	Atp8b1_predicted	195	2456	NM_001106140 /// XM_001064261 /// XM_214553	18q12.1	12.60	0.000
1378134_at	ATPase, Class I, type 8B, member 1 (predicted)	Atp8b1_predicted	338	4260	NM_001106140 /// XM_001064261 /// XM_214553	18q12.1	12.59	0.000
1395267_at	CDNA clone IMAGE:7366335	---	95	1174	BM391972	---	12.41	0.000
1369874_at	G protein-coupled receptor 1	Gpr1	77	956	NM_012961	9q31	12.37	0.016
1383474_at	interleukin-1 receptor-associated kinase 2	Irak2	103	1241	NM_001025422	4q42	12.01	0.000
1375934_at	similar to RIKEN cDNA D330045A20 (predicted)	RGD1566282_predicted	262	3127	XM_001054034 /// XM_236533	Xq35	11.93	0.004
1379390_at	ST6 (alpha-N-acetyl-neuraminyl-2,3-beta-galactosyl-1,3)-N-acetylgalactosaminide alpha-2,6-sialyltransferase 2	St6galnac2	194	2191	NM_001031652	10q32.3	11.28	0.000
1388703_at	endothelial cell adhesion molecule	Esam	160	1748	NM_001004245	8q21	10.92	0.002
1379636_at	similar to hypothetical protein FLJ32954	LOC313840	266	2880	NM_001037200	6q11	10.81	0.003
1387123_at	cytochrome P450, family 17, subfamily a, polypeptide 1	Cyp17a1	413	4444	NM_012753	1q55	10.76	0.000
1387156_at	hydroxysteroid (17-beta) dehydrogenase 2	Hsd17b2	507	5354	NM_024391	19q12	10.56	0.000
1382868_at	sema domain, transmembrane domain (TM), and cytoplasmic domain, (semaphorin) 6A (predicted)	Sema6a_predicted	582	6041	NM_001108430 /// XM_001054004 /// XM_341612	18q11	10.38	0.001
1367841_a_at	prolactin family 8, subfamily a, member 9	Prl8a9	402	4135	NM_134385	17p11	10.29	0.001
1383554_at	similar to RIKEN cDNA D330045A20 (predicted)	RGD1566282_predicted	309	3166	XM_001054034 /// XM_236533	Xq35	10.26	0.005
1387282_at	heat shock 22kDa protein 8	Hspb8	91	935	NM_053612	12q16	10.24	0.001
1390190_at	placenta-specific 1	Plac1	1268	12838	NM_001024894	Xq36	10.13	0.001
1376937_at	similar to 4631422O05Rik protein (predicted)	RGD1565927_predicted	93	933	XM_001056823 /// XM_221535	11q12	10.08	0.002
1388721_at	heat shock 22kDa protein 8	Hspb8	210	2061	NM_053612	12q16	9.80	0.000
1367577_at	heat shock 27kDa protein 1	Hspb1	97	940	NM_031970	12q12	9.73	0.000
1382949_at	retinol dehydrogenase 12 (predicted)	Rdh12_predicted	89	857	NM_001108037 /// XM_001080897 /// XM_234334	6q24	9.64	0.006

Table S4. Trophoblast differentiation-associated genes

Affymetrix ID	Gene Title	Gene Symbol	Stem Ave	Dif Ave	GenBank	Chromosomal Location	dif/ stem	t-test
1370371_a_at	CEA-related cell adhesion molecule 1 /// CEA-related cell adhesion molecule 10	Ceacam1 /// Ceacam10	514	4941	NM_001033860 /// NM_001033861 /// NM_001033862 /// NM_031755 /// NM_173339	1q21 /// 1q21-q22	9.62	0.002
1395408_at	nitric oxide synthase trafficker	Nostrin	299	2864	NM_001024260	3q21	9.59	0.000
1381428_a_at	hemochromatosis	Hfe	344	3275	AI177364	---	9.51	0.002
1385361_at	ATPase, class V, type 10A	Atp10a	585	5563	XM_001054901 /// XM_001054957 /// XM_344901	1q22	9.51	0.000
1381473_at	---	---	236	2237	BG381125	---	9.47	0.014
1369957_at	regulator of G-protein signaling 5	Rgs5	136	1287	NM_019341	13q24	9.44	0.001
1390406_at	Rho GTPase activating protein 18 (predicted)	Arhgap18_predicted	434	4093	NM_001106354 /// XM_001062453 /// XM_215247	1p12	9.42	0.000
1379356_at	similar to RIKEN cDNA C230093N12 (predicted)	RGD1310037_predicted	376	3386	AI574991	---	9.00	0.002
1370874_at	Prolactin family 3, subfamily b, member 1	Prl3b1	1556	13469	NM_012535	17p11	8.66	0.000
1382017_at	Transcribed locus, moderately similar to XP_985665.1 PREDICTED: hypothetical protein LOC72575 [Mus musculus]	---	1611	13864	AI012949	---	8.60	0.000
1375635_at	Transcribed locus	---	134	1144	AI406410	---	8.54	0.002
1384277_at	pleckstrin homology domain containing, family H (with MyTH4 domain) member 1 (predicted)	Plekhh1_predicted	525	4478	NM_001108036 /// XM_001080885 /// XM_234332	6q24	8.54	0.002
1389353_at	sema domain, transmembrane domain (TM), and cytoplasmic domain, (semaphorin) 6D (predicted)	Sema6d_predicted	496	4154	NM_001107768 /// XM_001077367 /// XM_230583	3q35	8.38	0.001
1379982_at	nuclear receptor interacting protein 1 (predicted)	Nrip1_predicted	131	1095	BE117278	---	8.35	0.000
1384406_at	similar to C-C chemokine receptor type 11 (C-C CKR-11) (CC-CKR-11) (CCR-11) (Chemokine receptor-like 1) (CCRL1) (CCX CKR)	LOC685243	355	2960	XM_001061561 /// XM_001061617 /// XM_001069795 /// XM_001069841	8q32	8.35	0.001
1385926_at	Transcribed locus	---	119	993	BF550565	---	8.32	0.000
1391871_at	similar to Serologically defined colon cancer antigen 13 (predicted)	RGD1564816_predicted	109	895	NM_001109060 /// XM_001056759 /// XM_001056824 /// XM_573341	12p12	8.25	0.000
1385251_at	hypothetical protein LOC500638	LOC500638	104	839	NM_001025051 /// XM_001075934	6q16	8.08	0.000
1391424_at	Transcribed locus	---	237	1875	AA944326	---	7.92	0.000
1369559_a_at	CD47 antigen (Rh-related antigen, integrin-associated signal transducer)	Cd47	653	5153	NM_019195	11q21	7.90	0.000
1373433_at	nucleosome binding protein 1 (predicted) /// similar to Nucleosome binding protein 1 (Nucleosome binding protein 45) (NBP-45) (GARP45 protein)	LOC680182 /// LOC681284 /// Nsbp1_predicted	1093	8590	XM_001056034 /// XM_001058107 /// XM_001061077	1q22 /// Xq31	7.86	0.000
1370695_s_at	tribbles homolog 3 (Drosophila)	Trib3	293	2284	NM_144755	3q41	7.81	0.000
1395020_at	pleckstrin homology domain containing, family H (with MyTH4 domain) member 1 (predicted)	Plekhh1_predicted	235	1824	NM_001108036 /// XM_001080885 /// XM_234332	6q24	7.75	0.000

Table S4. Trophoblast differentiation-associated genes

Affymetrix ID	Gene Title	Gene Symbol	Stem Ave	Dif Ave	GenBank	Chromosomal Location	dif/ stem	t-test
1371959_at	histone cluster 2, H2aa (predicted) /// similar to H2A histone family, member O	Hist2h2aa_predicted /// LOC682560 /// LOC690131	180	1393	XM_001062026 /// XM_001062079 /// XM_001073395 /// XM_345255	2q34	7.74	0.000
1382225_at	Transcribed locus	---	290	2220	BF284510	---	7.65	0.002
1373504_at	GLI pathogenesis-related 1 (glioma)	Glipr1	131	971	NM_001011987	7q21	7.43	0.001
1376671_at	similar to Ferritin heavy chain (Ferritin H subunit) (Proliferation-inducing gene 15 protein)	LOC681066	1965	14549	XM_001060160	Xq22	7.40	0.000
1370941_at	platelet derived growth factor receptor, alpha polypeptide	Pdgfra	314	2316	NM_012802 /// XM_001067574 /// XM_001067631 /// XM_001067681 /// XM_214030	14p11	7.38	0.000
1372084_at	protein tyrosine phosphatase 4a3 (predicted)	Ptp4a3_predicted	186	1371	XM_001069043 /// XM_001069094 /// XM_343259	7q34	7.36	0.001
1395557_at	core promoter element binding protein	Copeb	433	3134	BF565718	17	7.23	0.000
1368321_at	early growth response 1	Egr1	170	1230	NM_012551	18q	7.23	0.002
1390655_at	antisense paternally expressed gene 3	Apeg3	162	1149	BF420653	1	7.08	0.001
1368735_a_at	transient receptor potential cation channel, subfamily V, member 2	Trpv2	231	1625	NM_017207	10q23	7.03	0.000
1379396_at	engulfment and cell motility 1, ced-12 homolog (C. elegans) (predicted)	Elmo1_predicted	116	804	NM_001108415 /// XM_001059750 /// XM_341532	17q11	6.94	0.000
1375707_at	---	---	1389	9639	AA817993	---	6.94	0.000
1390021_at	histone cluster 1, H2bh	Hist1h2bh	482	3341	NM_001107352 /// XM_001054570 /// XM_001071676	17p11	6.93	0.000
1390529_at	CD83 antigen (predicted)	Cd83_predicted	425	2926	NM_001108410 /// XM_001057071 /// XM_341509	17p12	6.88	0.001
1395010_at	Transcribed locus	---	194	1326	AI102173	---	6.83	0.001
1382345_at	PCTAIRE-motif protein kinase 2	Pctk2	864	5892	NM_001108082 /// XM_001055332 /// XM_001077276 /// XM_001077300 /// XM_235049	7q13	6.82	0.000
1392547_at	hypothetical LOC302884	MGC105649	352	2391	NM_001008518	3q35	6.80	0.002
1376498_at	similar to 2900002H16Rik protein (predicted)	RGD1307973_predicted	558	3788	XM_001079000 /// XM_222150	12q15	6.79	0.000
1386059_at	Transcribed locus	---	519	3497	BF284475	---	6.74	0.000
1373881_at	Rho, GDP dissociation inhibitor (GDI) beta	Arhgdib	614	4099	NM_001009600	4q43	6.67	0.000
1383531_at	similar to adult retina protein (predicted)	RGD1310862_predicted	209	1367	AA901061	---	6.55	0.000
1368080_at	response gene to complement 32	Rgc32	206	1333	XM_001072931	15q12	6.48	0.002
1378126_at	similar to RIKEN cDNA B930096L08 (predicted)	RGD1306142_predicted	189	1215	XM_001070028 /// XM_231654	4q22	6.42	0.000
1368519_at	serine (or cysteine) peptidase inhibitor, clade E, member 1	Serpine1	349	2238	NM_012620	12q11-q12	6.41	0.000
1388432_at	optineurin	Optn	161	1027	NM_145081	17q12.3	6.39	0.000
1376117_at	solute carrier family 44, member 4	Slc44a4	521	3326	NM_212541	20p12	6.38	0.000
1394599_at	similar to cDNA sequence BC022133 (predicted)	RGD1561296_predicted	188	1177	XM_575607	4q34	6.25	0.014
1377880_at	Transcribed locus	---	292	1820	AI170633	---	6.23	0.000

Table S4. Trophoblast differentiation-associated genes

Affymetrix ID	Gene Title	Gene Symbol	Stem Ave	Dif Ave	GenBank	Chromosomal Location	dif/ stem	t-test
1395381_at	---	---	1060	6602	BF542239	---	6.23	0.000
1373826_at	yippee-like 5 (Drosophila)	Ypel5	332	2049	NM_001035221	6q13	6.17	0.006
1372308_at	CDNA clone IMAGE:7366335	---	285	1746	BM386869	---	6.12	0.000
1367970_at	profilin 2	Pfn2	411	2514	NM_030873	2q31	6.12	0.000
1388698_at	extracellular matrix protein 1	Ecm1	1255	7660	NM_053882	2q34	6.11	0.000
1379724_at	similar to pleckstrin homology-like domain, family B, member 2	LOC685611	260	1572	XM_001064525	11q21	6.06	0.000
1394931_at	muscleblind-like 2 (predicted)	Mbnl2_predicted	170	1027	AI180361	15	6.03	0.000
1389538_at	nuclear factor of kappa light chain gene enhancer in B-cells inhibitor, alpha	Nfkbia	580	3402	NM_001105720 /// XM_001075778 /// XM_343065	6q23	5.86	0.000
1387219_at	adrenomedullin	Adm	229	1327	NM_012715	1q33	5.81	0.013
1378094_at	Transcribed locus	---	364	2110	BM389654	11	5.80	0.000
1380622_at	Sil1 protein	RGD:735103	1153	6689	BI289578	18	5.80	0.000
1367733_at	carbonic anhydrase 2	Ca2	340	1935	NM_019291	2q23	5.69	0.007
1372328_at	kinesin light chain 4	Klc4	192	1090	NM_001009601	9q12	5.67	0.000
1371933_at	---	---	239	1355	AI228039	2	5.66	0.000
1368948_at	moesin	Msn	438	2476	NM_030863	Xq31	5.66	0.001
1369590_a_at	DNA-damage inducible transcript 3	Ddit3	582	3287	NM_001109986 /// NM_024134	7q22	5.65	0.003
1386895_at	melanoma antigen, family D, 1	Maged1	2198	12337	NM_053409	Xq31	5.61	0.000
1370950_at	phosphatidic acid phosphatase type 2B	Ppap2b	164	922	NM_138905	5q34	5.61	0.005
1380338_at	Transcribed locus	---	174	972	BF562910	---	5.60	0.005
1386922_at	carbonic anhydrase 2	Ca2	244	1363	NM_019291	2q23	5.59	0.002
1387630_at	ELOVL family member 5, elongation of long chain fatty acids (yeast)	Elovl5	941	5256	NM_134382 /// XM_001075440	8q31	5.58	0.001
1382118_at	Transcribed locus, strongly similar to NP_001029332.1 hypothetical protein LOC619476 [Rattus norvegicus]	---	748	4177	BE104676	---	5.58	0.001
1389502_at	---	---	322	1796	BG378715	---	5.57	0.004
1368488_at	nuclear factor, interleukin 3 regulated	Nfil3	180	995	NM_053727	17p14	5.53	0.001
1398319_at	Nclone10 mRNA	---	498	2744	AI071000	---	5.52	0.000
1376304_at	similar to AI661453 protein (predicted)	RGD1561662_predicted	194	1069	AI716243	---	5.50	0.002
1367601_at	Cbp/p300-interacting transactivator, with Glu/Asp-rich carboxy-terminal domain, 2	Cited2	1390	7635	NM_053698	1p12	5.49	0.000
1383715_at	similar to hypothetical protein D4Ertd89e	RGD1305703	352	1931	NM_001014191	5q24	5.49	0.001
1394948_at	arrestin domain containing 3	Arrdc3	181	987	BI303527	2	5.46	0.000
1386075_at	Myc induced nuclear antigen	Mina	153	830	NM_153309	11q12	5.42	0.002
1389430_at	Transcribed locus	---	230	1241	AI176172	---	5.39	0.000
1397680_at	Transcribed locus	---	198	1053	BF288560	---	5.31	0.000
1377405_at	Ral GEF with PH domain and SH3 binding motif 2	Ralgps2	299	1578	BM387133	13	5.28	0.001
1384653_at	similar to melanoma ubiquitous mutated protein	LOC684135 /// LOC690082	265	1397	XM_001069081 /// XM_001073181	Xq37	5.28	0.000
1392264_s_at	serine (or cysteine) peptidase inhibitor, clade E, member 1	Serpine1	233	1226	NM_012620	12q11-q12	5.26	0.000

Table S4. Trophoblast differentiation-associated genes

Affymetrix ID	Gene Title	Gene Symbol	Stem Ave	Dif Ave	GenBank	Chromosomal Location	dif/ stem	t-test
1384769_a_at	similar to Zinc finger X-linked protein ZXDB (predicted) /// similar to Zinc finger X-linked protein ZXDB	LOC683508 /// LOC683516 /// RGD1563355_predicted	274	1434	XM_001066253 /// XM_001066304 /// XM_234140	Xq31	5.23	0.000
1368275_at	sterol-C4-methyl oxidase-like	Sc4mol	1030	5389	NM_080886	16p13	5.23	0.000
1371575_at	moesin	Msn	1509	7862	BF281185	X	5.21	0.000
1367696_at	interferon induced transmembrane protein 2 (1-8D)	Ifitm2	292	1514	NM_030833	1q41	5.18	0.000
1387498_a_at	Fibroblast growth factor receptor 1	Fgfr1	166	859	NM_024146	16q12.4	5.17	0.001
1367602_at	Cbp/p300-interacting transactivator, with Glu/Asp-rich carboxy-terminal domain, 2	Cited2	4265	21976	NM_053698	1p12	5.15	0.000
1384037_at	signal-induced proliferation-associated 1 like 2	Sipa1l2	249	1284	NM_001009704	19q12	5.15	0.020
1368578_at	hydroxy-delta-5-steroid dehydrogenase, 3 beta- and steroid delta-isomerase 1	Hsd3b1	1636	8421	NM_001007719	2q34	5.15	0.000
1368679_a_at	Yamaguchi sarcoma viral (v-yes-1) oncogene homolog	Lyn	712	3645	NM_001111098 /// NM_030857	5q12	5.12	0.000
1384216_at	---	---	184	938	AW534148	---	5.11	0.000
1381942_at	hypermethylated in cancer 1 (predicted)	Hic1_predicted	638	3251	NM_001107021 /// XM_001080644 /// XM_220706	10q24	5.09	0.000
1374137_at	E74-like factor 1	Elf1	258	1313	AA818055	15	5.09	0.000
1382891_at	similar to ubiquitin specific protease 27, X chromosome (predicted)	RGD1564628_predicted	274	1392	XM_228777 /// XR_008155	Xq13	5.09	0.002
1372760_at	CDNA clone IMAGE:7321089	---	241	1228	AI176579	---	5.09	0.023
1387015_at	profilin 2	Pfn2	1014	5151	NM_030873	2q31	5.08	0.000
1388986_at	core promoter element binding protein	Copeb	626	3179	AI598339	6	5.08	0.000
1371951_at	four and a half LIM domains 2	Fhl2	290	1464	NM_031677	9q22	5.05	0.017
1368489_at	fos-like antigen 1	Fosl1	997	5028	NM_012953	1q43	5.04	0.000
1383137_at	SRY-box containing gene 4 (predicted)	Sox4_predicted	266	1342	XM_001068302 /// XM_344594	17p12	5.04	0.009
1389594_at	similar to Protein C20orf22 homolog	LOC499913	311	1557	NM_001024314	3q41	5.01	0.000
1373270_at	WD repeat domain, phosphoinositide interacting 1 (predicted)	Wipi1_predicted	612	3044	XM_001081631 /// XM_001081633 /// XM_221063	10q32.1	4.98	0.003
1388921_at	Transcribed locus	---	223	1109	BI279587	---	4.97	0.002
1369166_at	matrix metallopeptidase 9	Mmp9	245	1209	NM_031055	3q42	4.93	0.000
1390459_at	Transcribed locus	---	222	1095	BG670247	---	4.93	0.002
1374480_at	dishevelled associated activator of morphogenesis 1 (predicted)	Daam1_predicted	1329	6544	NM_001108030 /// XM_001080564 /// XM_234275	6q24	4.92	0.000
1367894_at	insulin induced gene 1	Insig1	780	3836	NM_022392	4q11	4.92	0.000
1375910_at	CDC42 effector protein (Rho GTPase binding) 3 (predicted)	Cdc42ep3_predicted	492	2406	NM_001048044 /// XM_001063467 /// XM_233815	6q11	4.90	0.000
1371332_at	similar to Histone H1.2 (H1 VAR.1) (H1c)	LOC684681	1284	6284	XM_001071565	---	4.89	0.000
1370688_at	glutamate-cysteine ligase, catalytic subunit	Gclc	1317	6423	NM_012815	8q31	4.88	0.000
1389554_at	nuclear receptor subfamily 2, group F, member 2	Nr2f2	532	2593	AA956784	1	4.88	0.000
1372101_at	phosphatidic acid phosphatase type 2B	Ppap2b	462	2245	NM_138905	5q34	4.86	0.001
1368262_at	PH domain and leucine rich repeat protein phosphatase	Phlpp	642	3108	NM_021657 /// XM_001063791	13p13	4.84	0.002
1368189_at	7-dehydrocholesterol reductase	Dhcr7	216	1037	NM_022389	1q41	4.81	0.001

Table S4. Trophoblast differentiation-associated genes

Affymetrix ID	Gene Title	Gene Symbol	Stem Ave	Dif Ave	GenBank	Chromosomal Location	dif/ stem	t-test
1378128_at	Transcribed locus	---	314	1509	BM383749	---	4.81	0.007
1390412_at	solute carrier family 39 (iron-regulated transporter), member 1	Slc40a1	177	850	NM_133315	9q22	4.80	0.001
1372097_at	Interferon regulatory factor 8	Irf8	465	2231	NM_001008722	19q12	4.80	0.001
1387060_at	Kruppel-like factor 6	Klf6	819	3915	NM_031642	17q12.2	4.78	0.000
1375962_at	Transcribed locus	---	775	3694	BE102925	---	4.77	0.012
1382955_at	G protein-coupled receptor 126 (predicted)	Gpr126_predicted	630	2983	XM_001071417 /// XM_218313	1p13	4.73	0.000
1368430_at	legumain	Lgmn	3873	18305	NM_022226	6q32	4.73	0.000
1395745_at	aspartate-beta-hydroxylase (predicted)	Asph_predicted	574	2693	AI113308	---	4.69	0.006
1383229_at	ATP-binding cassette, sub-family A (ABC1), member 7	Abca7	202	944	NM_207598	7q11	4.68	0.005
1371729_at	yippee-like 5 (Drosophila)	Ypel5	302	1404	NM_001035221	6q13	4.66	0.001
1372069_at	ankyrin repeat domain 15	Ankrd15	490	2279	XM_001055725 /// XM_001080106	1q51	4.66	0.000
1372447_at	Fibroblast growth factor receptor 1 /// similar to fibroblast growth factor receptor 1 isoform 9 precursor	Fgfr1 /// LOC500109	306	1426	NM_024146 /// XM_001053923 /// XM_001081961	16q12.4 /// 4q24	4.65	0.000
1369313_at	four and a half LIM domains 2	Fhl2	283	1313	NM_031677	9q22	4.64	0.000
1393050_at	hypothetical LOC302495	LOC302495	660	3062	NM_001106950 /// XM_001070285 /// XR_006183	Xq36	4.64	0.000
1376057_at	Phosphodiesterase 8A	Pde8a	340	1572	NM_198767	1q31	4.63	0.001
1372523_at	glutamate-cysteine ligase, catalytic subunit	Gclc	1617	7445	NM_012815	8q31	4.60	0.000
1369248_a_at	baculoviral IAP repeat-containing 4	Birc4	726	3337	NM_022231	Xq11	4.60	0.001
1398450_at	Transcribed locus	---	177	813	BI291373	---	4.60	0.004
1390803_at	HIV TAT specific factor 1 (predicted)	Htatsf1_predicted	4311	19468	NM_001108259 /// XM_001058638 /// XM_229199	Xq36	4.52	0.000
1389453_at	RAD52 motif 1 (predicted)	Rdm1_predicted	201	905	NM_001105842 /// XM_001081488 /// XM_220931	10q32.1	4.50	0.000
1389918_at	Transcribed locus, strongly similar to XP_979526.1 PREDICTED: similar to palladin isoform 1 [Mus musculus]	---	608	2733	BM391364	16	4.50	0.000
1383216_at	similar to mKIAA0431 protein	RGD1305781	563	2518	XM_001078346 /// XM_235378	19q12	4.48	0.002
1370351_at	tudor domain containing 7	Tdrd7	342	1530	NM_138871	5q22	4.47	0.000
1388109_at	G protein-coupled receptor 116	Gpr116	1465	6540	NM_139110	9q12	4.46	0.000
1370379_at	protease, serine, 8 (prostasin)	Prss8	264	1173	NM_138836	1q36	4.43	0.000
1372836_at	Transcribed locus	---	371	1643	AI170399	---	4.43	0.000
1368885_at	ectonucleoside triphosphate diphosphohydrolase 1	Entpd1	491	2177	NM_022587	1q54	4.43	0.000
1370694_at	tribbles homolog 3 (Drosophila)	Trib3	266	1176	NM_144755	3q41	4.42	0.000
1382535_at	potassium channel tetramerisation domain containing 12 (predicted)	Kctd12_predicted	513	2266	AI030942	---	4.42	0.003
1381353_at	similar to melanoma antigen family A, 5	MGC114427	875	3863	NM_001024893	Xq37	4.41	0.007
1375140_at	muscleblind-like 2 (predicted)	Mbnl2_predicted	209	920	AA818999	15	4.41	0.002
1367571_a_at	insulin-like growth factor 2	Igf2	3273	14329	NM_031511 /// XM_001064965	1q41	4.38	0.000
1386321_s_at	tribbles homolog 3 (Drosophila)	Trib3	695	3032	NM_144755	3q41	4.37	0.002
1368549_at	high mobility group box transcription factor 1	Hbp1	722	3146	NM_013221	6q16	4.36	0.000

Table S4. Trophoblast differentiation-associated genes

Affymetrix ID	Gene Title	Gene Symbol	Stem Ave	Dif Ave	GenBank	Chromosomal Location	dif/ stem	t-test
1379371_at	Transcribed locus	---	352	1532	BF284791	---	4.35	0.000
1372175_at	similar to DNA segment, Chr 10, University of California at Los Angeles 1 (predicted)	RGD1560401_predicted	677	2942	NM_001106386 /// XM_215383	20p11	4.35	0.000
1367741_at	homocysteine-inducible, endoplasmic reticulum stress-inducible, ubiquitin-like domain member 1	Herpud1	642	2781	NM_053523	19p12	4.33	0.001
1393346_at	similar to RIKEN cDNA 5830436D01 (predicted)	RGD1561673_predicted	384	1663	XM_001062586 /// XM_574843	2q12	4.33	0.002
1369358_a_at	huntingtin-associated protein 1	Hap1	433	1872	NM_024133 /// NM_177982	10q32.1	4.32	0.000
1371883_at	monocyte to macrophage differentiation-associated	Mmd	847	3656	NM_001007673 /// XM_001081244	10q26	4.31	0.000
1368009_at	glucosamine	Gne	514	2214	NM_053765	5q22	4.31	0.000
1388991_at	similar to SEC14 and spectrin domains 1 (predicted)	RGD1562244_predicted	417	1795	XM_001066119 /// XM_230024	3q23	4.30	0.000
1389318_at	dishevelled associated activator of morphogenesis 1 (predicted)	Daam1_predicted	2514	10742	NM_001108030 /// XM_001080564 /// XM_234275	6q24	4.27	0.000
1398312_s_at	solute carrier family 14 (urea transporter), member 2	Slc14a2	473	2020	NM_001110270 /// NM_019347 /// NM_177962	18q12.3	4.27	0.000
1377925_at	Transcribed locus	---	245	1045	BE105762	---	4.27	0.002
1372390_at	Transcribed locus	---	2908	12366	AI710604	---	4.25	0.001
1388348_at	ELOVL family member 5, elongation of long chain fatty acids (yeast)	Elovl5	5188	22056	BI278590	8	4.25	0.006
1367614_at	annexin A1	Anxa1	3856	16381	NM_012904	1q51	4.25	0.000
1389500_at	similar to ras homolog gene family, member U	LOC678766	430	1823	XM_001053087	1	4.24	0.004
1377213_at	similar to protein kinase/endoribonuclease(IRE1) alpha (predicted)	RGD1559716_predicted	543	2299	XM_001081576 /// XM_573211	10q32.1	4.23	0.000
1372475_at	PTEN induced putative kinase 1 (predicted)	Pink1_predicted	267	1128	AI410877	5	4.22	0.000
1389255_at	Cadherin 5 (predicted)	Cdh5_predicted	1488	6278	NM_001107407 /// XM_001057656 /// XM_226213	19p14	4.22	0.000
1389301_at	muscleblind-like 2 (predicted)	Mbnl2_predicted	2885	12146	AI176665	15	4.21	0.000
1389986_at	CDNA clone IMAGE:7321089	---	1401	5882	AI008409	---	4.20	0.027
1383131_at	integrin beta 2	Itgb2	719	3016	NM_001037780 /// XM_001069791 /// XM_001071845	20p12	4.19	0.000
1374379_at	dual specificity phosphatase 23 (predicted)	Dusp23_predicted	221	923	BF396810	---	4.18	0.000
1367650_at	lipocalin 7	Lcn7	736	3075	NM_053582	5q36	4.18	0.000
1367628_at	lectin, galactose binding, soluble 1	Lgals1	1807	7510	NM_019904	7q34	4.16	0.000
1373286_at	filamin binding LIM protein 1	Fblim1	1129	4688	NM_001007554	5q36	4.15	0.000
1388464_at	similar to cullin 7	LOC680835	1167	4841	XM_001056401 /// XM_001056459 /// XM_001056520	9q12	4.15	0.000
1398397_at	similar to Parg1-pending protein	RGD1306185	1049	4348	AI407483	2	4.15	0.001
1371537_at	UDP-Gal:betaGlcNAc beta 1,4-galactosyltransferase, polypeptide 5 (predicted)	B4galt5_predicted	3881	16060	NM_001108608 /// XM_001073367 /// XM_342584	3q42	4.14	0.000
1367651_at	cathepsin D	Ctsd	2606	10770	NM_134334	1q41	4.13	0.000

Table S4. Trophoblast differentiation-associated genes

Affymetrix ID	Gene Title	Gene Symbol	Stem Ave	Dif Ave	GenBank	Chromosomal Location	dif/ stem	t-test
1383528_at	similar to mKIAA0215 protein (predicted)	RGD1563945_predicted	391	1614	XM_001056752 /// XM_234528	Xq12	4.12	0.005
1398333_at	endothelial PAS domain protein 1	Epas1	209	861	AI598434	6	4.12	0.011
1382210_at	hypothetical LOC302495	LOC302495	304	1251	NM_001106950 /// XM_001070285 /// XR_006183	Xq36	4.11	0.000
1399069_at	Transcribed locus	---	241	982	AI234008	---	4.07	0.000
1372160_at	bladder cancer associated protein homolog (human)	Blcap	1090	4427	NM_133582	3q42	4.06	0.000
1390486_at	ubiquitin specific protease 32 (predicted)	Usp32_predicted	688	2788	NM_001107032 /// XM_001081109 /// XM_220798	10q26	4.05	0.000
1368254_a_at	sphingosine kinase 1	Sphk1	218	883	NM_133386	10q32.3	4.05	0.000
1374489_at	GTP binding protein 2	Gtpbp2	204	825	XM_001058711 /// XM_001064572 /// XM_001064689 /// XM_343535	9q12	4.05	0.000
1369788_s_at	Jun oncogene	Jun	459	1852	NM_021835	5q34.1	4.04	0.001
1395353_at	similar to KIAA0368 (predicted)	RGD1306148_predicted	237	956	XM_001061185 /// XM_232937	5q24	4.04	0.004
1374429_at	Transcribed locus	---	547	2194	AI598401	---	4.01	0.000
1398275_at	matrix metallopeptidase 9	Mmp9	530	2120	NM_031055	3q42	4.00	0.000
1373818_at	potassium channel tetramerisation domain containing 12 (predicted)	Kctd12_predicted	1257	5024	AI408351	---	4.00	0.001
1391672_at	Transcribed locus	---	241	961	BF399539	---	3.99	0.000
1384652_at	HIV TAT specific factor 1 (predicted)	Htatsf1_predicted	272	1082	NM_001108259 /// XM_001058638 /// XM_229199	Xq36	3.98	0.000
1368884_at	ectonucleoside triphosphate diphosphohydrolase 1	Entpd1	355	1410	NM_022587	1q54	3.98	0.000
1381705_at	pantothenate kinase 3 (predicted)	Pank3_predicted	707	2792	BE110067	---	3.95	0.001
1384508_at	Transcribed locus, moderately similar to NP_033341.1 TAF6 RNA polymerase II, TATA box binding protein (TBP)-associated factor [Mus musculus]	---	568	2237	AI576469	12	3.94	0.000
1368106_at	polo-like kinase 2 (Drosophila)	Plk2	309	1211	NM_031821	2q14	3.92	0.000
1394086_at	SUMO1/sentrin specific protease 7 (predicted)	Senp7_predicted	354	1386	NM_001105888 /// XM_001057745 /// XM_221566	11q12	3.92	0.000
1372977_at	similar to mitogen-activated protein kinase kinase kinase kinase 5 isoform 2 (predicted)	RGD1562028_predicted	712	2789	XM_001076790 /// XM_578547	6q24	3.92	0.001
1389107_at	similar to KIAA1749 protein (predicted)	RGD1304623_predicted	914	3575	NM_001108164 /// XM_001054062 /// XM_236385	8q24	3.91	0.000
1371115_at	protein tyrosine phosphatase, receptor type, E	Ptpre	253	991	XM_001056164 /// XM_341950	1q41	3.91	0.000
1369032_at	bladder cancer associated protein homolog (human)	Blcap	417	1626	NM_133582	3q42	3.90	0.000
1373019_at	epidermal growth factor receptor pathway substrate 15	Eps15	563	2190	AI172468	5	3.89	0.001
1372299_at	cyclin-dependent kinase inhibitor 1C (P57)	Cdkn1c	9367	36398	NM_001033757 /// NM_001033758 /// NM_182735	1q42	3.89	0.001
1371440_at	Beta-2 microglobulin	B2m	312	1207	NM_012512	3q35	3.87	0.000

Table S4. Trophoblast differentiation-associated genes

Affymetrix ID	Gene Title	Gene Symbol	Stem Ave	Dif Ave	GenBank	Chromosomal Location	dif/ stem	t-test
1396036_at	Ral GEF with PH domain and SH3 binding motif 2	Ralgps2	276	1068	NM_001100680 /// XM_001073076 /// XM_222773	13q22	3.87	0.000
1376600_at	Transcribed locus	---	1810	7008	BI289616	6	3.87	0.000
1380229_at	Transcribed locus	---	312	1201	AW530004	---	3.85	0.000
1371692_at	myeloid/lymphoid or mixed-lineage leukemia (trithorax homolog, Drosophila); translocated to, 11	Mllt11	208	802	NM_001013912	2q34	3.85	0.000
1367932_at	3-hydroxy-3-methylglutaryl-Coenzyme A synthase 1	Hmgcs1	511	1966	NM_017268	2q16	3.85	0.000
1373363_at	microtubule-associated protein 1b	Map1b	976	3759	NM_019217 /// XM_001061557 /// XM_215469	2q12	3.85	0.000
1373231_at	OTU domain containing 5	Otud5	1026	3947	NM_001037496	Xq13	3.85	0.000
1391495_at	Similar to RIKEN cDNA 2010012O05 (predicted)	RGD1311783_predicted	359	1378	XM_001059424 /// XM_215266	1q54	3.84	0.002
1394451_at	annexin A1	Anxa1	227	871	NM_012904	1q51	3.84	0.000
1367519_at	oxysterol binding protein-like 2	Osbpl2	536	2051	NM_001013079	3q43	3.83	0.000
1383744_at	Transcribed locus	---	567	2163	AI146250	---	3.82	0.004
1390832_at	transmembrane and coiled-coil domain family 3 (predicted)	Tmcc3_predicted	239	913	NM_001108084 /// XM_001080385 /// XM_235072	7q13	3.82	0.003
1388403_at	similar to NADP+-specific isocitrate dehydrogenase	LOC361596	260	993	NM_001014161	1q31	3.81	0.000
1392267_at	similar to RIKEN cDNA B930096L08 (predicted)	RGD1306142_predicted	253	965	XM_001070028 /// XM_231654	4q22	3.81	0.001
1393037_at	SH3 domain binding glutamic acid-rich protein like 2 (predicted)	Sh3bgrl2_predicted	281	1071	BG373089	8	3.81	0.000
1387587_at	Fas ligand (TNF superfamily, member 6)	Faslg	1816	6909	NM_012908	13q22	3.80	0.000
1382086_at	Transcribed locus	---	413	1568	AI235883	---	3.79	0.000
1377810_at	Ral GEF with PH domain and SH3 binding motif 2	Ralgps2	262	989	AI408104	13	3.78	0.003
1375548_at	similar to RIKEN cDNA 4732418C07 (predicted)	RGD1310351_predicted	368	1387	NM_001106677 /// XM_001064076 /// XM_001064133 /// XM_001064193 /// XM_216499	5q36	3.77	0.000
1370898_at	stannin	Snn	298	1121	NM_001034083 /// XM_001052997	10q11	3.76	0.031
1370156_at	prion protein	Prnp	1723	6476	NM_012631	3q36	3.76	0.000
1387502_at	serine/threonine kinase 17b (apoptosis-inducing)	Stk17b	577	2162	NM_133392	9q31	3.75	0.001
1367839_at	farnesyl diphosphate farnesyl transferase 1	Fdft1	402	1503	NM_019238	15p12	3.74	0.002
1371765_at	histone 2a	H2a	592	2215	NM_021840 /// XM_001053023	10q22	3.74	0.002
1376040_at	signal-induced proliferation-associated 1 like 2	Sipa1l2	2200	8228	NM_001009704	19q12	3.74	0.008
1375170_at	S100 calcium binding protein A11 (calizzarin)	S100a11	4687	17470	NM_001004095	2q34	3.73	0.004
1388725_at	leptin receptor overlapping transcript	Leprot	339	1261	NM_020099	5q33	3.72	0.001
1395274_at	dystonin (predicted)	Dst_predicted	641	2380	NM_001108208 /// XM_001054738 /// XM_237042	9q21	3.71	0.007
1368241_a_at	flotillin 1	Flot1	853	3152	NM_022701	20p12	3.69	0.000
1369305_at	RAB3A interacting protein (rabin3)-like 1	Rab3il1	310	1143	NM_134411	1q43	3.69	0.000

Table S4. Trophoblast differentiation-associated genes

Affymetrix ID	Gene Title	Gene Symbol	Stem Ave	Dif Ave	GenBank	Chromosomal Location	dif/ stem	t-test
1380334_at	hypothetical RNA binding protein RGD1359713	RGD1359713	1317	4830	NM_001005882	14p11	3.67	0.000
1374943_at	similar to RIKEN cDNA 2010011I20 (predicted)	RGD1311378_predicted	420	1535	NM_001106547 /// XM_001053264 /// XM_230889	3q42	3.66	0.000
1373962_at	ubiquitin specific protease 32 (predicted)	Usp32_predicted	1241	4519	NM_001107032 /// XM_001081109 /// XM_220798	10q26	3.64	0.002
1383478_at	Janus kinase 1	Jak1	780	2841	XM_001061647 /// XM_342872	5q31.3-q35	3.64	0.002
1392785_at	potassium channel tetramerisation domain containing 12 (predicted)	Kctd12_predicted	1914	6965	AA800908	---	3.64	0.000
1388169_at	jumonji domain containing 1C	Jmjd1c	1200	4369	XM_001080424 /// XM_228122	20p11	3.64	0.000
1395887_at	similar to RIKEN cDNA 1600029D21	LOC363060	3928	14289	NM_001014209	8q23	3.64	0.000
1394639_at	similar to CDC42 small effector 2 (predicted) /// hypothetical protein LOC686503 /// hypothetical protein LOC691031	LOC686503 /// LOC691031 /// RGD1563924_predicted	651	2365	XM_001074441 /// XM_001076592 /// XM_577792	10q22 /// 1q22	3.64	0.008
1382060_at	Transcribed locus	---	366	1325	BE115085	---	3.62	0.001
1375672_at	Transcribed locus	---	449	1622	BG380279	---	3.61	0.000
1387550_a_at	solute carrier family 14 (urea transporter), member 2	Slc14a2	1770	6365	NM_001110270 /// NM_019347 /// NM_177962	18q12.3	3.60	0.002
1377923_at	START domain containing 8 (predicted)	Stard8_predicted	324	1165	NM_001107849 /// XM_001068833 /// XM_231459	Xq31	3.59	0.002
1382001_at	similar to Myeloid/lymphoid or mixed-lineage leukemia protein 3 homolog (Histone-lysine N-methyltransferase, H3 lysine-4 specific MLL3)	LOC502710	778	2795	XR_006158	4q11	3.59	0.000
1391012_at	Transcribed locus	---	266	955	BF283210	---	3.59	0.000
1368025_at	DNA-damage-inducible transcript 4	Ddit4	814	2917	NM_080906	20q11	3.59	0.000
1389507_at	neural precursor cell expressed, developmentally down-regulated 4-like	Nedd4l	228	816	XM_001064485 /// XM_574161	18q12.1	3.58	0.000
1371689_at	eukaryotic translation elongation factor 1 alpha 1	Eef1a1	302	1079	BE107334	8	3.58	0.018
1372115_at	similar to ubiquitin protein ligase E3 component n-recognin 2	LOC363188	817	2920	XM_001061775 /// XM_001061828 /// XM_001061882	9q12	3.58	0.000
1398302_at	prolactin family 7, subfamily a, member 3	Prl7a3	10970	39208	NM_022530	17p11	3.57	0.002
1389791_at	ceroid-lipofuscinosis, neuronal 8	Cln8	949	3393	NM_001007686	16q12.5	3.57	0.000
1393345_at	aspartate-beta-hydroxylase (predicted)	Asph_predicted	713	2546	BF561145	---	3.57	0.000
1379934_at	lysosomal trafficking regulator	Lyst	227	811	BI285676	17	3.57	0.001
1397509_x_at	reproductive homeobox on X chromosome, 9	Rhox9	3486	12417	NM_001024874	Xq11	3.56	0.000
1388251_at	protein kinase C, iota	Prkci	250	888	XM_001064601 /// XM_342223	2q24	3.56	0.000
1374642_at	zinc finger protein 64	Zfp64	1510	5359	NM_001012093	3q42	3.55	0.000
1395770_at	Transcribed locus	---	364	1289	BM391695	---	3.55	0.001
1399152_at	epidermal growth factor receptor pathway substrate 15	Eps15	702	2486	NM_001009424	5q35	3.54	0.000
1374404_at	Jun oncogene	Jun	259	912	NM_021835	5q34.1	3.53	0.001

Table S4. Trophoblast differentiation-associated genes

Affymetrix ID	Gene Title	Gene Symbol	Stem Ave	Dif Ave	GenBank	Chromosomal Location	dif/ stem	t-test
1389315_at	G protein-coupled receptor kinase-interactor 2	Git2	705	2489	NM_001005553	12q16	3.53	0.000
1393845_a_at	transmembrane channel-like gene family 4	Tmc4	278	982	NM_001034104	1q12	3.53	0.000
1374675_at	similar to D7H11orf15 protein (predicted)	RGD1310775_predicted	295	1039	BG374992	1	3.53	0.000
1386998_at	aldolase C	Aldoc	283	997	NM_012497	10	3.53	0.001
1371431_at	peroxisome biogenesis factor 5 (predicted)	Pex5_predicted	726	2550	XM_001062081 /// XM_232343	4q42	3.51	0.000
1387260_at	Kruppel-like factor 4 (gut)	Klf4	395	1387	NM_053713	5q24	3.51	0.000
1375899_at	similar to HTPAP protein (predicted)	RGD1559978_predicted	250	878	BF283736	---	3.51	0.001
1391083_at	Rho GTPase activating protein 22 (predicted)	Arhgap22_predicted	586	2052	NM_001107297 /// XM_001056636 /// XM_224637	16p16	3.50	0.004
1391279_at	Scinderin	Scin	1137	3965	NM_198748	6q21	3.49	0.002
1393334_at	Transcribed locus	---	302	1049	AW528448	---	3.47	0.000
1380909_at	Transcribed locus	---	939	3251	BM385894	---	3.46	0.008
1373184_at	similar to chondroitin sulfate GalNAcT-2 (predicted)	RGD1563660_predicted	400	1384	NM_001106616 /// XM_001056935 /// XM_232316	4q42	3.46	0.000
1375658_at	Transcribed locus	---	1215	4200	AI412317	---	3.46	0.000
1376861_at	similar to RIKEN cDNA 1810018L05 /// similar to motile sperm domain containing 1	LOC317312 /// LOC686087	308	1062	NM_001014107 /// XM_001052988 /// XM_001066482	14q21 /// Xq36	3.44	0.000
1395579_at	DEAH (Asp-Glu-Ala-His) box polypeptide 32 (predicted)	Dhx32_predicted	417	1433	BG666347	1	3.44	0.003
1389528_s_at	Jun oncogene	Jun	1206	4145	NM_021835	5q34.1	3.44	0.000
1394346_at	mannosidase, alpha, class 1A, member 2 (predicted)	Man1a2_predicted	478	1641	NM_001106452 /// XM_001062587 /// XM_227543	2q34	3.43	0.000
1368147_at	dual specificity phosphatase 1	Dusp1	420	1441	NM_053769	10q12	3.43	0.016
1386662_at	similar to Sestrin 2 (Hi95) (predicted)	RGD1566319_predicted	318	1090	NM_001109358 /// XM_001065176 /// XM_001065235 /// XM_001065290 /// XM_578496	5q36	3.43	0.000
1385089_at	grainyhead-like 1 (Drosophila) (predicted)	Grhl1_predicted	659	2258	XM_001074769 /// XM_234006	6q16	3.43	0.000
1388945_at	similar to 1300014I06Rik protein	RGD1311307	496	1691	NM_001025719	17p12	3.41	0.000
1392542_at	similar to CDC42 small effector 2 (predicted) /// hypothetical protein LOC686503 /// hypothetical protein LOC691031	LOC686503 /// LOC691031 /// RGD1563924_predicted	1785	6058	XM_001074441 /// XM_001076592 /// XM_577792	10q22 /// 1q22	3.39	0.000
1383880_at	integrin alpha V (predicted)	Itgav_predicted	427	1449	NM_001106549 /// XM_001068715 /// XM_230950	3q24	3.39	0.001
1386893_at	granulin	Grn	3709	12577	NM_017113	10q32.1	3.39	0.000
1387703_a_at	ubiquitin specific peptidase 2	Usp2	374	1270	NM_053774	8q22	3.39	0.007
1373373_at	glutamine fructose-6-phosphate transaminase 1	Gfpt1	914	3097	BI295011	4	3.39	0.000
1387870_at	zinc finger protein 36	Zfp36	2194	7419	NM_133290	1q21	3.38	0.000
1379275_at	sorting nexin 10	Snx10	373	1259	NM_001013085	4q24	3.37	0.000

Table S4. Trophoblast differentiation-associated genes

Affymetrix ID	Gene Title	Gene Symbol	Stem Ave	Dif Ave	GenBank	Chromosomal Location	dif/ stem	t-test
1384060_at	Janus kinase 1	Jak1	318	1071	XM_001061647 /// XM_342872	5q31.3-q35	3.37	0.006
1378420_at	Transcribed locus	---	338	1137	BF408177	---	3.37	0.002
1373546_at	Putative UA20 protein	Ua20	723	2429	AI409922	16q12.5	3.36	0.000
1389401_at	---	---	309	1036	BI294956	---	3.35	0.007
1387227_at	Wiskott-Aldrich syndrome protein interacting protein	Waspip	374	1252	NM_057192	3q23	3.35	0.003
1392802_at	similar to RIKEN cDNA 4930431E10 (predicted) /// similar to Smad ubiquitination regulatory factor 1 isoform 2	LOC690516 /// RGD1309707_predicted	289	966	NM_001109598 /// XM_001071299 /// XM_001074660	12p11	3.35	0.000
1367538_at	---	---	246	821	BF284303	---	3.34	0.000
1377968_at	Transcribed locus	---	1748	5831	AI069912	---	3.34	0.000
1398287_at	plasminogen activator, urokinase	Plau	488	1623	NM_013085	15p16	3.33	0.000
1390842_at	transcription factor AP-2, alpha (predicted)	Tcfap2a_predicted	727	2419	NM_001107345 /// XM_001062658 /// XM_225238	17p12	3.33	0.006
1376728_at	protein phosphatase 1, regulatory (inhibitor) subunit 8 (predicted)	Ppp1r8_predicted	1426	4728	BM392264	5	3.31	0.000
1393234_at	Transcribed locus, moderately similar to XP_994560.1 PREDICTED: similar to Rho guanine exchange factor 16 [Mus musculus]	---	514	1704	BI285850	1	3.31	0.026
1397729_x_at	similar to RIKEN cDNA 1600029D21	LOC363060	5827	19300	NM_001014209	8q23	3.31	0.000
1395320_s_at	reproductive homeobox on X chromosome, 9	Rhox9	3386	11184	NM_001024874	Xq11	3.30	0.000
1387065_at	phospholipase C, delta 4	Plcd4	906	2990	NM_080688	9q33	3.30	0.001
1388714_at	elongation factor RNA polymerase II (predicted)	Ell_predicted	532	1754	NM_001107304 /// XM_001070626 /// XM_224727	16p14	3.30	0.000
1373066_at	Transcribed locus	---	359	1185	AI407797	---	3.30	0.000
1370930_at	---	---	402	1326	BF417285	10	3.30	0.000
1393294_at	cytoplasmic polyadenylation element binding protein 4 (predicted)	Cpeb4_predicted	569	1874	BG671389	10	3.29	0.000
1377141_at		LOC498685	257	845	AI103397	---	3.29	0.000
1392929_at	RGD1565616 (predicted)	RGD1565616_predicted	323	1054	NM_001109206 /// XM_001081185 /// XM_575237	3q36	3.27	0.000
1393683_at	similar to KIAA0368 (predicted)	RGD1306148_predicted	487	1588	XM_001061185 /// XM_232937	5q24	3.26	0.000
1377911_at	Transcribed locus	---	826	2684	AI029434	---	3.25	0.000
1389230_at	arrestin domain containing 3	Arrdc3	987	3196	AA818910	2	3.24	0.000
1387101_at	acyl-CoA synthetase long-chain family member 4	Acsl4	1107	3582	NM_053623	Xq14	3.24	0.000
1377636_at	Transcribed locus, strongly similar to XP_579796.1 PREDICTED: hypothetical protein XP_579796 [Rattus norvegicus]	---	277	894	BE109363	---	3.23	0.002
1398598_at	dystonin (predicted)	Dst_predicted	2248	7260	NM_001108208 /// XM_001054738 /// XM_237042	9q21	3.23	0.000
1383590_at	similar to chondroitin beta1,4 N-acetylgalactosaminyltransferase (predicted)	RGD1307618_predicted	480	1551	AA963863	16	3.23	0.000
1386581_at	integrin alpha V (predicted)	Itgav_predicted	492	1586	NM_001106549 /// XM_001068715 /// XM_230950	3q24	3.22	0.000

Table S4. Trophoblast differentiation-associated genes

Affymetrix ID	Gene Title	Gene Symbol	Stem Ave	Dif Ave	GenBank	Chromosomal Location	dif/ stem	t-test
1371684_at	pelota homolog	Pelo	938	3023	NM_001007634	2q14	3.22	0.000
1369954_at	isocitrate dehydrogenase 1 (NADP+), soluble	Idh1	387	1248	NM_031510	9q32	3.22	0.000
1388130_at	zyxin	Zyx	2053	6605	XM_001072096 /// XM_216124	4q23	3.22	0.001
1371071_at	guanine nucleotide binding protein beta 4 subunit	Gnb4	745	2396	NM_001013910	2q25	3.22	0.000
1377307_at	similar to mammary tumor virus receptor 2 isoform 2	RGD1309879	376	1210	NM_001011711	19q12	3.22	0.019
1393236_at	RIO kinase 3 (yeast) (predicted)	Riok3_predicted	2953	9487	NM_001108423 /// XM_001072670 /// XM_341578	18p13	3.21	0.018
1387675_at	plasminogen activator, urokinase	Plau	762	2444	NM_013085	15p16	3.21	0.000
1393159_at	glutaredoxin 2 (thioltransferase)	Glrx2	715	2293	BG671304	13	3.21	0.000
1398327_at	pleckstrin homology domain containing, family C (with FERM domain) member 1	Plekhc1	2131	6832	NM_001011915	15p14	3.21	0.000
1370128_at	heart and neural crest derivatives expressed transcript 1	Hand1	1169	3745	NM_021592	10q22	3.20	0.001
1371781_at	signal transducer and activator of transcription 3	Stat3	360	1152	NM_012747	10q32.1	3.20	0.014
1373313_at	Sel1 (suppressor of lin-12) 1 homolog (C. elegans)	Sel1h	736	2354	NM_177933	6q31	3.20	0.001
1373088_at	hypothetical protein LOC682888	LOC682888	2270	7243	XM_001063567	18	3.19	0.000
1382807_at	similar to hypothetical protein	RGD1310450	415	1322	NM_001014218	9q33	3.18	0.000
1394047_at	pregnancy specific beta-1-glycoprotein 4	Psg4	772	2444	BE107848	---	3.17	0.001
1384865_at	Transcribed locus, strongly similar to XP_346369.1 PREDICTED: similar to leucine zipper, down-regulated in cancer 1 [Rattus norvegicus]	---	1759	5568	AW535518	X	3.16	0.007
1384413_at	similar to solute carrier family 35, member A5 (predicted)	RGD1564361_predicted	290	917	XM_001066912 /// XM_573283	11q21	3.16	0.001
1374903_at	glucosaminyl (N-acetyl) transferase 2, I-branching enzyme	Gcnt2	495	1560	NM_001001511	17p12	3.15	0.001
1373872_at	Transcribed locus, strongly similar to XP_574462.1 PREDICTED: similar to hypothetical protein C230069C04 [Rattus norvegicus]	---	340	1068	BE096535	---	3.14	0.000
1384791_at	UDP-GlcNAc:betaGal beta-1,3-N-acetylglucosaminyltransferase 1 (predicted)	B3gnt1_predicted	558	1752	NM_001107240 /// XM_001061345 /// XM_223674	14q22	3.14	0.002
1371499_at	CD9 antigen	Cd9	2729	8573	NM_053018 /// XM_001063955	4q42	3.14	0.000
1383686_at	synaptogyrin 1	Syngr1	778	2435	BE111537	7	3.13	0.000
1386967_at	ras homolog gene family, member Q	Rhoq	3994	12485	NM_053522	6q12	3.13	0.001
1398719_at	chromodomain protein, Y chromosome-like 2 (predicted)	Cdyl2_predicted	266	830	NM_001106189 /// XM_001078221 /// XM_226510	19q12	3.12	0.000
1390987_at	Transcribed locus	---	316	984	AI406858	---	3.11	0.006
1392995_at	similar to zinc finger protein 322a	LOC680201 /// LOC684943	362	1127	XM_001056103 /// XM_001072600	17q11	3.11	0.001
1385052_at	RAB3A interacting protein (rabin3)-like 1	Rab3il1	275	856	BM389915	1	3.11	0.002
1384182_at	pleckstrin homology domain containing, family C (with FERM domain) member 1	Plekhc1	562	1746	NM_001011915	15p14	3.11	0.000
1368213_at	P450 (cytochrome) oxidoreductase	Por	701	2170	NM_031576	12q12	3.10	0.000

Table S4. Trophoblast differentiation-associated genes

Affymetrix ID	Gene Title	Gene Symbol	Stem Ave	Dif Ave	GenBank	Chromosomal Location	dif/ stem	t-test
1389971_at	sarcoglycan, epsilon	Sgce	1394	4316	NM_001002023	---	3.10	0.003
1387109_at	P450 (cytochrome) oxidoreductase	Por	677	2095	NM_031576	12q12	3.10	0.000
1390117_at	Transcribed locus	---	294	909	BG372455	---	3.09	0.000
1398362_at	Notch gene homolog 2 (Drosophila)	Notch2	1932	5957	NM_024358	2q34	3.08	0.001
1372854_at	tetratricopeptide repeat domain 17	Ttc17	308	947	NM_001107752 /// XM_001078737 /// XM_230313	3q31	3.08	0.001
1369958_at	ras homolog gene family, member B	Rhob	2343	7202	NM_022542	6q14	3.07	0.001
1381111_at	Transcribed locus	---	263	808	BI275744	---	3.07	0.022
1372771_at	Transcribed locus	---	329	1010	BI279659	---	3.07	0.001
1375421_a_at	praja 2, RING-H2 motif containing	Pja2	711	2181	NM_138896	9q37	3.07	0.000
1394706_at	pantothenate kinase 3 (predicted)	Pank3_predicted	716	2193	AI044545	---	3.06	0.003
1387130_at	solute carrier family 39 (iron-regulated transporter), member 1	Slc40a1	545	1669	NM_133315	9q22	3.06	0.000
1383628_at	Transcribed locus	---	324	990	BG376623	---	3.06	0.001
1387883_a_at	thymosin, beta 4	Tmsb4x	1203	3671	NM_031136 /// XM_001053027	X	3.05	0.000
1373626_at	Transcribed locus	---	2412	7341	AI102507	---	3.04	0.000
1394511_at	myb-like, SWIRM and MPN domains 1 (predicted)	Mysm1_predicted	266	808	XM_001058020 /// XM_216460	5q33	3.04	0.000
1369943_at	transglutaminase 2, C polypeptide	Tgm2	319	968	NM_019386	3q42	3.04	0.010
1389483_at	Dpy-19-like 1 (C. elegans) (predicted)	Dpy19l1_predicted	1622	4925	XM_001053132 /// XM_235970	8q13	3.04	0.002
1372888_at	ubiquitination factor E4A, UFD2 homolog (S. cerevisiae)	Ube4a	380	1151	NM_207610	8q22	3.03	0.000
1387867_at	aldehyde dehydrogenase family 9, subfamily A1	Aldh9a1	2060	6241	NM_022273	13q24	3.03	0.000
1388700_at	CDNA clone IMAGE:7317367	---	781	2359	AW253004	---	3.02	0.003
1384205_at	N-glycanase 1	Ngly1	342	1032	NM_001014136	15p16	3.02	0.001
1382818_at	similar to hypothetical protein D630010C10	RGD1309752	570	1721	NM_001025710	9q31	3.02	0.000
1374066_at	cell division cycle 2-like 6 (CDK8-like) (predicted)	Cdc2l6_predicted	298	898	NM_001107634 /// XM_001063700 /// XM_228203	20q12	3.02	0.001
1389639_at	similar to Shb protein (predicted)	RGD1565350_predicted	315	949	XR_008772	5q22	3.01	0.000
1370245_at	cathepsin L	Ctsl	3411	10278	NM_013156	17p14	3.01	0.000
1389418_at	---	---	306	920	AI410452	---	3.01	0.000
1375637_at	similar to RIKEN cDNA 1110003E01	RGD1311122	348	1044	NM_001037792	14p11	3.00	0.011
1371755_at	similar to RIKEN cDNA B230219D22 (predicted)	RGD1566359_predicted	1323	3969	AA799607	---	3.00	0.000
1382044_at	hypothetical protein LOC498796	LOC498796	397	1187	NM_001025034	17q12.3	2.99	0.001
1367857_at	fatty acid desaturase 1	Fads1	281	837	NM_053445	1q43	2.98	0.000
1372223_at	cytoplasmic polyadenylation element binding protein 4 (predicted)	Cpeb4_predicted	890	2640	BG374299	10	2.97	0.000
1389511_s_at	---	---	558	1650	BF403383	---	2.96	0.000
1372106_at	EH-domain containing 4	Ehd4	428	1265	NM_139324	3q35	2.96	0.000
1387848_at	3-hydroxy-3-methylglutaryl-Coenzyme A reductase	Hmgcr	407	1203	NM_013134	2q12	2.96	0.000
1371249_at	X-box binding protein 1	Xbp1	3233	9555	NM_001004210	14q21	2.96	0.000
1375944_at	acyl-CoA synthetase short-chain family member 2 (predicted)	Acss2_predicted	1423	4202	NM_001107793 /// XM_001064684 /// XM_230773	3q42	2.95	0.000

Table S4. Trophoblast differentiation-associated genes

Affymetrix ID	Gene Title	Gene Symbol	Stem Ave	Dif Ave	GenBank	Chromosomal Location	dif/ stem	t-test
1376489_at	son of sevenless homolog 2 (Drosophila)	Sos2	1224	3608	XM_001080400	6q24	2.95	0.001
1389617_at	ELK3, member of ETS oncogene family (predicted)	Elk3_predicted	938	2761	NM_001108743 /// XM_001080272 /// XM_343198	7q13	2.94	0.005
1396191_at	Eukaryotic translation initiation factor 5B	Eif5b	859	2527	NM_001110141 /// XM_001055509 /// XM_001057591 /// XM_001057637 /// XM_218162	9q21	2.94	0.000
1369953_a_at	CD24 antigen	Cd24	6918	20201	NM_012752	20q13	2.92	0.000
1378154_at	Transcribed locus	---	325	948	BG668499	---	2.92	0.002
1394815_at	Transcribed locus	---	304	888	BE107436	---	2.92	0.001
1377820_a_at	arginine-tRNA-protein transferase 1 (predicted)	Ate1_predicted	2862	8342	NM_001106300 /// XM_001077907 /// XM_001077921 /// XM_001079539 /// XM_001079547 /// XM_001079559 /// XM_215086	1q37	2.91	0.000
1368126_at	acetoacetyl-CoA synthetase	Aacs	304	887	NM_023104	12q14	2.91	0.011
1388448_at	Hypothetical protein LOC691031	LOC691031	2314	6710	XM_001076592	10q22	2.90	0.000
1368146_at	dual specificity phosphatase 1	Dusp1	1321	3831	NM_053769	10q12	2.90	0.000
1367646_at	cathepsin B	Ctsb	6967	20177	NM_022597	15p12	2.90	0.002
1388690_at	breast cancer metastasis-suppressor 1-like (predicted)	Brms1l_predicted	1492	4316	NM_001106731 /// XM_001075894 /// XM_216712	6q23	2.89	0.000
1393824_at	Transcribed locus	---	309	893	BG377414	---	2.89	0.000
1370129_at	meningioma expressed antigen 5 (hyaluronidase)	Mgea5	750	2164	NM_131904	1q54	2.89	0.000
1398923_at	similar to D11Bwg0434e protein (predicted)	RGD1305687_predicted	2244	6474	XM_001079783 /// XM_237786	10q24	2.88	0.000
1397417_at	Transcribed locus	---	536	1545	BG671626	---	2.88	0.000
1374433_at	similar to Ferritin light chain (Ferritin L subunit) (predicted)	RGD1563902_predicted	908	2617	BI301532	---	2.88	0.004
1367759_at	H1 histone family, member 0	H1f0	2742	7894	NM_012578	7q34	2.88	0.002
1375349_at	similar to sorbin and SH3 domain containing 1 isoform 3 /// sorbin and SH3 domain containing 1 (predicted)	LOC678826 /// Sorbs1_predicted	437	1259	XM_001053355 /// XM_001066536	1q53	2.88	0.013
1379555_at	Transcribed locus	---	782	2248	AI233902	---	2.88	0.002
1374808_at	similar to hypothetical protein FLJ21156 (predicted)	RGD1309823_predicted	313	900	XM_001067111 /// XR_006864	5q36	2.87	0.008
1373164_at	serine/threonine kinase 17b (apoptosis-inducing)	Stk17b	1294	3710	AI012590	9	2.87	0.000
1381480_at	sterile alpha and TIR motif containing 1 (predicted)	Sarm1_predicted	309	885	NM_001105817 /// XM_001080875 /// XM_213404	10q25	2.87	0.011
1375027_at	Cell cycle progression 1 (predicted)	Ccpg1_predicted	403	1150	NM_001108770 /// XM_001053133 /// XM_001053208 /// XM_001053270 /// XM_001053343 /// XM_343429	8q24	2.86	0.000
1373127_at	tripartite motif-containing 44	Trim44	683	1951	BM383645	3	2.86	0.000

Table S4. Trophoblast differentiation-associated genes

Affymetrix ID	Gene Title	Gene Symbol	Stem Ave	Dif Ave	GenBank	Chromosomal Location	dif/ stem	t-test
1382500_at	similar to Sestrin 2 (Hi95) (predicted)	RGD1566319_predicted	772	2198	NM_001109358 /// XM_001065176 /// XM_001065235 /// XM_001065290 /// XM_578496	5q36	2.85	0.000
1389488_at	---	---	5790	16463	AA800750	---	2.84	0.000
1371953_at	cyclin G2 (predicted)	Ccng2_predicted	300	851	NM_001105725 /// XM_001063045 /// XM_223270	14p22	2.84	0.000
1392955_at	Sel1 (suppressor of lin-12) 1 homolog (C. elegans)	Sel1h	429	1216	NM_177933	6q31	2.83	0.000
1393643_at	reticulocalbin 1 (predicted)	Rcn1_predicted	633	1791	NM_001108586 /// XM_001073225 /// XM_342481	3q32	2.83	0.001
1375700_at	kelch-like 22 (Drosophila) (predicted)	Klhl22_predicted	1141	3226	NM_001107079 /// XM_001063385 /// XM_221268	11q23	2.83	0.000
1372779_at	UDP-GlcNAc:betaGal beta-1,3-N-acetylglucosaminyltransferase 1 (predicted)	B3gnt1_predicted	1543	4355	NM_001107240 /// XM_001061345 /// XM_223674	14q22	2.82	0.001
1368011_at	ferredoxin reductase	Fdxr	407	1147	NM_024153	10q32.2	2.82	0.005
1368207_at	FXYD domain-containing ion transport regulator 5	Fxyd5	663	1866	NM_021909	1q21	2.81	0.000
1399030_at	WD repeat domain 45	Wdr45	306	859	NM_001013958	Xq13	2.81	0.001
1370010_at	lysosomal membrane glycoprotein 2	Lamp2	3193	8940	NM_017068	Xq11	2.80	0.000
1374525_at	Ras association (RalGDS/AF-6) and pleckstrin homology domains 1 (predicted)	Raph1_predicted	531	1483	NM_001108798 /// XM_001070281 /// XM_343579	9q31	2.79	0.004
1368947_at	growth arrest and DNA-damage-inducible 45 alpha	Gadd45a	301	840	NM_024127	4q31-q33	2.79	0.001
1385888_at	Transcribed locus	---	319	888	AA963272	---	2.78	0.017
1375174_at	dpy-19-like 1 (C. elegans) (predicted)	Dpy19l1_predicted	979	2719	XM_001053132 /// XM_235970	8q13	2.78	0.001
1390731_at	Transcribed locus	---	825	2292	BI288533	---	2.78	0.000
1376990_at	similar to HCDI protein (predicted)	RGD1309307_predicted	471	1306	BE118704	15	2.77	0.000
1375723_at	Transcribed locus	---	535	1485	AI385171	---	2.77	0.000
1390103_at	PHD finger protein 2 (predicted)	Phf2_predicted	459	1271	NM_001107342 /// XM_001056853 /// XM_225206	17p14	2.77	0.002
1388872_at	isopentenyl-diphosphate delta isomerase	Idi1	569	1575	BI290053	17	2.77	0.001
1369027_at	alpha 1,4-galactosyltransferase	A4galt	958	2643	NM_022240	7q34	2.76	0.000
1380665_at	CGI-58-like protein	LOC316122	637	1756	BF418059	8	2.76	0.000
1387847_at	phosphatidylinositol 3-kinase, catalytic, beta polypeptide	Pik3cb	916	2517	NM_053481	8q31	2.75	0.006
1367936_at	serine/threonine kinase 10	Stk10	1232	3385	XM_001064952 /// XM_001065000 /// XM_577078	10	2.75	0.000
1372129_at	similar to TBC1 domain family, member 8; BUB2-like protein 1; vascular Rab-GAP/TBC-containing (predicted)	RGD1310147_predicted	668	1836	NM_001108274 /// XM_001070650 /// XM_001070694 /// XM_001070738 /// XM_001070776 /// XM_001070820 /// XM_340793	10q22	2.75	0.000

Table S4. Trophoblast differentiation-associated genes

Affymetrix ID	Gene Title	Gene Symbol	Stem Ave	Dif Ave	GenBank	Chromosomal Location	dif/ stem	t-test
1390128_at	similar to chromatin modifying protein 1B	LOC682926 /// LOC689364	755	2073	NM_001109533 /// XM_001060811 /// XM_001067633	18q12.1	2.74	0.000
1384089_at	RAB guanine nucleotide exchange factor (GEF) 1 (predicted)	Rabgef1_predicted	399	1094	NM_001108333 /// XM_001072217 /// XM_341067	12q13	2.74	0.000
1393915_at	membrane bound O-acyltransferase domain containing 5	Mboat5	1067	2927	NM_001012189	4q42	2.74	0.000
1370071_at	adenosine deaminase	Ada	1983	5441	NM_130399	3q42	2.74	0.001
1367667_at	farnesyl diphosphate synthase	Fdps	1784	4893	NM_031840	2q34	2.74	0.000
1389282_at	Integrin alpha 3 (predicted)	Itga3_predicted	414	1134	NM_001108292 /// XM_001081309 /// XM_340884	10q31	2.74	0.000
1367874_at	ras homolog gene family, member Q	Rhoq	2717	7443	NM_053522	6q12	2.74	0.000
1387116_at	DnaJ (Hsp40) homolog, subfamily B, member 9	Dnajb9	1095	2995	NM_012699	6q16-q23	2.74	0.002
1385691_at	similar to RIKEN cDNA 5830435K17 (predicted)	RGD1560454_predicted	306	835	XM_001080836 /// XM_575496	4q24	2.73	0.001
1368878_at	isopentenyl-diphosphate delta isomerase	Idi1	1910	5214	NM_053539	17q12.1	2.73	0.001
1382901_at	growth factor independent 1B (predicted)	Gfi1b_predicted	338	924	NM_001107823 /// XM_001079155 /// XM_231109	3p12	2.73	0.002
1372781_at	WD repeat domain 47	Wdr47	484	1317	XM_001071369 /// XM_001081957	2q34	2.72	0.000
1369689_at	N-ethylmaleimide sensitive fusion protein	Nsf	659	1792	NM_021748	10q32.1	2.72	0.000
1389150_at	Transcribed locus	---	547	1488	AW524559	---	2.72	0.004
1373742_at	splA/ryanodine receptor domain and SOCS box containing 2	Spsb2	385	1046	NM_001009660	4q42	2.72	0.001
1376315_at	similar to putative alpha-mannosidase	RGD1303074	1313	3551	NM_001004230	3q42	2.70	0.000
1372638_at	Rho guanine nucleotide exchange factor 7	Arhgef7	696	1881	BG371454	16	2.70	0.005
1367715_at	tumor necrosis factor receptor superfamily, member 1a	Tnfrsf1a	1317	3548	NM_013091	4q42	2.70	0.000
1391607_at	similar to Sestrin 2 (Hi95) (predicted)	RGD1566319_predicted	1017	2739	NM_001109358 /// XM_001065176 /// XM_001065235 /// XM_001065290 /// XM_578496	5q36	2.69	0.000
1373775_at	NEDD8 ultimate buster-1	Nub1	321	862	NM_001013925 /// XM_001060617	4q11	2.69	0.002
1373650_at	cytidine monophospho-N-acetylneuraminic acid synthetase	Cmas	1946	5231	NM_001009419	4q44	2.69	0.002
1371783_at	heat shock protein	LOC290549	2201	5915	NM_207589	16p16	2.69	0.000
1392653_at	Transcribed locus	---	493	1321	BE118116	---	2.68	0.004
1383799_at	Transcribed locus, strongly similar to NP_001026482.1 phosphoinositide-3-kinase, catalytic, beta polypeptide [Gallus gallus]	---	734	1967	BF567886	---	2.68	0.014
1367869_at	oxidation resistance 1	Oxr1	319	854	XM_001061616 /// XM_001061721 /// XM_576258	7q31	2.68	0.003
1377644_at	similar to RIKEN cDNA 4921524J17 (predicted)	RGD1308706_predicted	1886	5051	XM_001068411 /// XM_214649	19q11	2.68	0.001
1375889_at	spermine synthase	Sms	504	1348	NM_001033899	Xq21	2.68	0.000
1369279_at	dehydrogenase/reductase (SDR family) member 9	Dhrs9	698	1865	NM_130819	3q21	2.67	0.007

Table S4. Trophoblast differentiation-associated genes

Affymetrix ID	Gene Title	Gene Symbol	Stem Ave	Dif Ave	GenBank	Chromosomal Location	dif/ stem	t-test
1371975_at	RNA binding motif, single stranded interacting protein 2	Rbms2	563	1504	BG377343	7	2.67	0.019
1383655_at	TNFAIP3 interacting protein 2	Tnip2	361	963	NM_001024771	14q21	2.66	0.001
1397386_at	AF4/FMR2 family, member 4 (predicted)	Aff4_predicted	528	1399	NM_001107001 /// XM_001073824 /// XM_220420	10q22	2.65	0.004
1393718_at	similar to DKFZP564P1916 protein (predicted)	RGD1311704_predicted	401	1061	BE117189	---	2.65	0.000
1390114_at	myelin protein zero-like 1	Mpzl1	3057	8085	NM_001007728	13q23	2.64	0.002
1374964_at	dipeptidylpeptidase 8 (predicted)	Dpp8_predicted	447	1182	NM_001108159 /// XM_001075075 /// XM_236345	8q24	2.64	0.000
1373996_at	Transcribed locus	---	568	1501	AI236940	---	2.64	0.000
1384399_at	ankyrin repeat domain 50 (predicted)	Ankrd50_predicted	342	905	XM_001069855 /// XM_215553	2q25	2.64	0.000
1373484_at	kelch-like 13 (Drosophila)	Klhl13	445	1173	XM_001061200 /// XM_233297	Xq12	2.64	0.021
1370812_at	Bcl2-like 1	Bcl2l1	339	892	NM_001033670 /// NM_001033671 /// NM_001033672 /// NM_031535	3q41.2	2.63	0.006
1370476_at	Stam binding protein	Stambp	305	802	NM_138531	4q34	2.63	0.001
1388426_at	sterol regulatory element binding factor 1	Srebf1	870	2288	XM_001075680 /// XM_213329	10q22	2.63	0.013
1374987_at	pantothenate kinase 3 (predicted)	Pank3_predicted	2320	6104	AI232974	---	2.63	0.000
1390422_at	PX domain containing serine/threonine kinase	Pxk	365	959	NM_182821	15p14	2.63	0.001
1372812_at	similar to mKIAA0256 protein (predicted)	RGD1559930_predicted	384	1008	XM_001077702 /// XM_215843	3q36	2.63	0.003
1367528_at	F-box and WD-40 domain protein 8 (predicted)	Fbxw8_predicted	982	2578	NM_001107145 /// XM_001076873 /// XM_001076911 /// XM_222223	12q16	2.63	0.000
1389355_at	immediate early response 5	Ier5	916	2397	NM_001025137	13q21	2.62	0.001
1377713_at	checkpoint suppressor 1 (predicted)	Ches1_predicted	340	889	NM_001108047 /// XM_001065012 /// XM_001065063 /// XM_001065120 /// XM_234377	6q32	2.62	0.022
1387269_s_at	plasminogen activator, urokinase receptor	Plaur	432	1131	NM_017350 /// NM_134352 /// XM_001059419	1q21	2.61	0.001
1368297_at	GATA binding protein 2	Gata2	556	1451	NM_033442	4q34-q41	2.61	0.042
1382254_at	phosphodiesterase 4D interacting protein (myomegalin)	Pde4dip	616	1607	BI295944	---	2.61	0.006
1369006_at	hexokinase 2	Hk2	798	2080	NM_012735	4q34	2.60	0.005
1389640_at	SH3 domain binding glutamic acid-rich protein like 2 (predicted)	Sh3bgrl2_predicted	2464	6412	BM391811	8	2.60	0.004
1372772_at	nuclear factor of activated T-cells 5 (predicted)	Nfat5_predicted	430	1119	NM_001107425 /// XM_001076581 /// XM_226436	19q12	2.60	0.003
1373208_at	Transcribed locus	---	372	968	BE113371	---	2.60	0.002
1388712_at	pleckstrin homology, Sec7 and coiled-coil domains 3	Pscd3	809	2103	NM_053912	12p11	2.60	0.001
1383770_at	Transcribed locus	---	756	1964	BE117323	---	2.60	0.008

Table S4. Trophoblast differentiation-associated genes

Affymetrix ID	Gene Title	Gene Symbol	Stem Ave	Dif Ave	GenBank	Chromosomal Location	dif/ stem	t-test
1390000_at	jumonji domain containing 3 (predicted)	Jmjd3_predicted	327	849	NM_001108829 /// XM_001079385 /// XM_343919	10q24	2.60	0.001
1379811_at	phosphodiesterase 4D interacting protein (myomegalin)	Pde4dip	312	810	AI763912	2	2.60	0.034
1376840_at	Transcribed locus	---	1276	3305	AI103040	---	2.59	0.000
1370940_at	tight junction protein 2	Tjp2	1018	2635	NM_053773	1q51	2.59	0.000
1375343_at	Transcribed locus	---	1066	2755	BE116572	---	2.59	0.005
1372601_at	activating transcription factor 5	Atf5	521	1345	NM_172336	1q22	2.58	0.000
1389392_at	Chloride channel 6 (predicted)	Clcn6_predicted	409	1056	NM_001106479 /// XM_001074014 /// XM_227870	5q36	2.58	0.024
1368177_at	acyl-CoA synthetase long-chain family member 3	Acsl3	770	1987	NM_057107	9q33	2.58	0.001
1383362_at	Transcribed locus	---	324	836	BI275850	---	2.58	0.002
1373385_at	similar to mahogunin, ring finger 1, mahoganoid	RGD1311862	703	1813	BF550271	10	2.58	0.000
1389541_at	Malignant fibrous histiocytoma amplified sequence 1 (predicted)	Mfhas1_predicted	395	1017	NM_001107316 /// XM_001058880 /// XM_224916	16q12.2	2.57	0.000
1372164_at	similar to hypothetical protein FLJ20154	RGD1305793	1159	2981	XM_001067855 /// XM_001067947 /// XM_001071072 /// XM_219958	1q54	2.57	0.000
1387074_at	regulator of G-protein signaling 2	Rgs2	902	2320	NM_053453	13q21	2.57	0.000
1384559_at	placenta specific homeobox 1 (predicted)	Psx1_predicted	1826	4694	XM_001076864 /// XM_231064	3p13	2.57	0.000
1371104_at	sterol regulatory element binding factor 1	Srebf1	336	863	XM_001075680 /// XM_213329	10q22	2.57	0.004
1389277_at	similar to KIAA0368 (predicted)	RGD1306148_predicted	1722	4419	XM_001061185 /// XM_232937	5q24	2.57	0.000
1373657_at	solute carrier family 31, member 2	Slc31a2	317	811	NM_001033693	5q24	2.56	0.003
1374696_at	---	---	810	2073	AI170182	---	2.56	0.000
1370987_at	sialophorin	Spn	3734	9556	XM_001080140 /// XM_344964	1q36	2.56	0.022
1391613_at	ELK4, member of ETS oncogene family (predicted)	Elk4_predicted	754	1929	NM_001107173 /// XM_001059832 /// XM_222630	13q13	2.56	0.001
1385108_at		RGD1309752_predicted	335	857	AW524173	---	2.56	0.017
1390825_at	solute carrier family 35, member B3 (predicted)	Slc35b3_predicted	621	1587	XM_001063451 /// XM_225253	17p12	2.56	0.000
1372462_at	acetyl-Coenzyme A acetyltransferase 2	Acat2	736	1876	NM_001006995	1q11	2.55	0.000
1389187_at	Transcribed locus, strongly similar to NP_001007709.1 hypothetical protein LOC315126 [Rattus norvegicus]	---	884	2251	BI286421	7	2.55	0.002
1369583_at	Jun dimerization protein 2	Jundp2	741	1887	NM_053894	6q31	2.55	0.000
1375921_at	Reticulocalbin 1 (predicted)	Rcn1_predicted	341	867	NM_001108586 /// XM_001073225 /// XM_342481	3q32	2.54	0.000
1387183_at	carnitine O-octanoyltransferase	Crot	488	1238	NM_031987	4q12	2.54	0.000
1377709_at	heart and neural crest derivatives expressed transcript 1	Hand1	9351	23698	AI072532	---	2.53	0.005
1369643_a_at	latrophilin 2	Lphn2	1109	2809	NM_134408	2q45	2.53	0.000

Table S4. Trophoblast differentiation-associated genes

Affymetrix ID	Gene Title	Gene Symbol	Stem Ave	Dif Ave	GenBank	Chromosomal Location	dif/ stem	t-test
1388824_at	similar to RIKEN cDNA 4930431E10 (predicted) /// similar to Smad ubiquitination regulatory factor 1 isoform 2	LOC690516 /// RGD1309707_predicted	471	1192	NM_001109598 /// XM_001071299 /// XM_001074660	12p11	2.53	0.002
1373592_at	similar to SPI6	MGC94010	5086	12866	NM_001007732	17p12	2.53	0.000
1369319_at	ADP-ribosylation factor-like 6 interacting protein 5	Arl6ip5	561	1418	NM_023972	4q34	2.53	0.003
1373219_at	snail homolog 1 (Drosophila)	Snai1	1095	2765	NM_053805	3q42	2.53	0.001
1388887_at	glycoprotein galactosyltransferase alpha 1, 3	Ggta1	1458	3679	AI178222	3	2.52	0.001
1392704_at	sorting nexin 4 (predicted)	Snx4_predicted	406	1026	AI029221	---	2.52	0.000
1367844_at	guanine nucleotide binding protein, alpha inhibiting 2	Gnai2	660	1663	NM_031035	8q32	2.52	0.004
1384292_at	docking protein 1	Dok1	347	874	NM_001025416	4q34	2.52	0.001
1388732_at	solute carrier family 35, member F5 (predicted)	Slc35f5_predicted	889	2237	NM_001105950 /// XM_001056041 /// XM_222576	13q11	2.52	0.001
1382174_at	Transcribed locus, strongly similar to NP_001026482.1 phosphoinositide-3-kinase, catalytic, beta polypeptide [Gallus gallus]	---	1503	3784	AI227996	---	2.52	0.009
1370347_at	PDZ and LIM domain 7	Pdlim7	1953	4916	NM_173125	17p14	2.52	0.000
1398340_at	cyclin D-type binding-protein 1	Ccndbp1	640	1611	NM_001013204	3q35	2.52	0.000
1379914_at	transcription factor CP2-like 2	Tcfcp2l2	476	1197	NM_001037354	6q16	2.52	0.005
1375739_at	EH-domain containing 4	Ehd4	1625	4072	NM_139324	3q35	2.51	0.002
1372030_at	zinc finger, FYVE domain containing 21 (predicted)	Zfyve21_predicted	641	1604	BI283114	6	2.50	0.001
1397866_at	serine (or cysteine) proteinase inhibitor, clade B, member 6b	Serpinb6b	2755	6896	NM_001012214	17p12	2.50	0.001
1379785_at	Transcribed locus	---	421	1052	BI275894	---	2.50	0.003
1375969_at	torsin A interacting protein 2	Tor1aip2	344	860	NM_199100	13q21	2.50	0.004
1374594_at	similar to RIKEN cDNA 1600029D21	LOC363060	8755	21865	NM_001014209	8q23	2.50	0.001
1398771_at	solute carrier family 3 (activators of dibasic and neutral amino acid transport), member 2	Slc3a2	3741	9339	NM_019283	1q43	2.50	0.000
1387446_at	core 1 UDP-galactose:N-acetylgalactosamine-alpha-R beta 1,3-galactosyltransferase	C1galt1	437	1091	NM_022950	4q21	2.49	0.015
1389439_at	---	---	457	1139	AI172618	---	2.49	0.045
1373393_at	similar to Ext1	LOC299907	510	1272	XM_001065900 /// XM_216920	7q31	2.49	0.002
1368144_at	regulator of G-protein signaling 2	Rgs2	702	1746	NM_053453	13q21	2.49	0.007
1368336_at	ferredoxin 1	Fdx1	1142	2839	NM_017126	8q24	2.49	0.002
1384186_at	similar to mKIAA0212 protein (predicted)	RGD1563633_predicted	982	2440	XM_001075914 /// XM_001075943 /// XM_238366	4q41	2.48	0.000
1397349_at	UBX domain containing 6 (predicted)	Ubxd6_predicted	403	1000	NM_001106086 /// XM_001059749 /// XM_214360	16q12.3	2.48	0.010
1383585_s_at	Transcribed locus	---	658	1630	AI043753	4	2.48	0.000
1396059_at	UBX domain containing 2	Ubxd2	769	1904	NM_001012025	13q12	2.48	0.000
1396195_at	ankyrin repeat domain 13	Ankrd13	1266	3135	NM_001012148	12q16	2.48	0.000
1372237_at	similar to testes development-related NYD-SP22 isoform 1 (predicted)	RGD1561916_predicted	572	1413	BI298306	---	2.47	0.000

Table S4. Trophoblast differentiation-associated genes

Affymetrix ID	Gene Title	Gene Symbol	Stem Ave	Dif Ave	GenBank	Chromosomal Location	dif/ stem	t-test
1388539_at	plakophilin 2	Pkp2	1752	4328	XM_001065241 /// XM_213560	11q23	2.47	0.000
1371645_at	stromal cell derived factor 2 (predicted)	Sdf2_predicted	1916	4725	NM_001105803 /// XM_001080807 /// XM_213377	10q25	2.47	0.000
1389546_at	angiomotin like 2	Amotl2	1460	3599	BF418582	8	2.46	0.004
1372733_at	Similar to intracellular membrane-associated calcium-independent phospholipase A2 gamma (predicted)	RGD1311444_predicted	686	1690	NM_001108020 /// XM_001078026 /// XM_234092	6q21	2.46	0.000
1370224_at	signal transducer and activator of transcription 3	Stat3	496	1221	NM_012747	10q32.1	2.46	0.003
1387903_at	praja 2, RING-H2 motif containing	Pja2	884	2178	NM_138896	9q37	2.46	0.000
1389482_at	similar to EST AA792894	RGD1306495	518	1275	NM_001009413	4q33	2.46	0.001
1373395_at	tripartite motif protein 41 (predicted)	Trim41_predicted	933	2298	XM_001069403 /// XM_220357	10q21	2.46	0.000
1383223_at	ubiquitin-like 3	Ubl3	404	995	NM_001015030	12p11	2.46	0.000
1371766_at	Transcribed locus	---	454	1116	BE113242	---	2.46	0.007
1367682_at	midkine	Mdk	2222	5464	NM_030859	3q24	2.46	0.001
1371587_at	mitogen-activated protein kinase kinase 1 interacting protein 1	Map2k1ip1	2729	6703	NM_001008375	2q44	2.46	0.003
1388653_at	similar to Paired amphipathic helix protein Sin3b (Transcriptional corepressor Sin3b) (Histone deacetylase complex subunit Sin3b)	LOC683381	762	1871	XM_001062859 /// XM_001062980	---	2.45	0.001
1395741_at	Meningioma expressed antigen 5 (hyaluronidase)	Mgea5	1447	3552	NM_131904	1q54	2.45	0.000
1382112_at	similar to chromatin modifying protein 1B	LOC682926 /// LOC689364	453	1112	NM_001109533 /// XM_001060811 /// XM_001067633	18q12.1	2.45	0.000
1391304_at	similar to Ubr2 protein (predicted)	RGD1561637_predicted	371	910	BM388658	9	2.45	0.004
1381335_at	---	---	3553	8710	BE349658	---	2.45	0.000
1381162_at	Similar to septin 10 isoform 1	LOC309891	434	1064	NM_001014033	20q13	2.45	0.003
1386962_at	phospholipase C, beta 4	Plcb4	414	1014	NM_024353	3q36	2.45	0.000
1373178_at	Transcribed locus	---	2062	5052	AA945183	---	2.45	0.000
1390423_at	pam, highwire, rpm 1 (predicted)	Phr1_predicted	1219	2987	NM_001106055 /// XM_001076799 /// XM_214245	15q22	2.45	0.000
1385887_at	Transcribed locus	---	383	936	BE107316	---	2.44	0.010
1384899_at	Transcribed locus	---	5406	13211	AA996921	---	2.44	0.002
1382568_at	Transcribed locus	---	640	1565	BE098457	---	2.44	0.000
1369472_a_at	activating transcription factor 2	Atf2	475	1159	NM_031018	3q23	2.44	0.000
1390430_at	nuclear receptor subfamily 1, group D, member 2	Nr1d2	360	877	BF284190	15	2.44	0.000
1383025_at	meningioma expressed antigen 5 (hyaluronidase)	Mgea5	613	1491	NM_131904	1q54	2.43	0.000
1370357_at	solute carrier family 30 (zinc transporter), member 4	Slc30a4	651	1579	NM_172066	3q35	2.43	0.000
1373624_at	Transcribed locus	---	663	1608	AA945713	---	2.43	0.002
1383306_at	similar to RIKEN cDNA 5830435K17 (predicted)	RGD1560454_predicted	1100	2665	XM_001080836 /// XM_575496	4q24	2.42	0.000
1373225_at	myeloid cell leukemia sequence 1	Mcl1	1803	4368	AI102618	2	2.42	0.000
1367915_at	diacylglycerol O-acyltransferase 1	Dgat1	824	1994	NM_053437	7q34	2.42	0.000

Table S4. Trophoblast differentiation-associated genes

Affymetrix ID	Gene Title	Gene Symbol	Stem Ave	Dif Ave	GenBank	Chromosomal Location	dif/ stem	t-test
1394363_at	RIO kinase 3 (yeast) (predicted)	Riok3_predicted	825	1997	NM_001108423 /// XM_001072670 /// XM_341578	18p13	2.42	0.001
1374399_at	EH-domain containing 4	Ehd4	997	2412	NM_139324	3q35	2.42	0.007
1373047_at	protein kinase C, iota	Prkci	1838	4439	XM_001064601 /// XM_342223	2q24	2.42	0.000
1380028_at	similar to ADP-ribosylation factor-like protein 4C (ADP-ribosylation factor-like 7)	LOC367311	339	818	XM_001057060 /// XM_001065966	9q35	2.42	0.001
1376758_at	inhibitor of growth family, member 1	Ing1	1435	3466	NM_001038591	16q12.5	2.41	0.006
1389316_at	ubiquitin specific peptidase 9, X chromosome (predicted)	Usp9x_predicted	866	2090	XM_001056701 /// XM_343766	Xq13	2.41	0.000
1392579_at	similar to 5830411E10Rik protein	RGD1306658	3490	8418	NM_001014216	9q22	2.41	0.003
1368308_at	myelocytomatosis viral oncogene homolog (avian)	Myc	1806	4355	NM_012603	7q33	2.41	0.006
1367853_at	solute carrier family 12, member 2	Slc12a2	2294	5528	NM_031798	18q12.1	2.41	0.000
1398909_at	hypothetical LOC301124	LOC301124	1215	2925	XM_001060121 /// XM_238570	9q11	2.41	0.001
1372840_at	zinc finger, BED domain containing 4 (predicted) /// similar to zinc finger, BED domain containing 4	LOC678931 /// LOC688556 /// Zbed4_predicted	621	1494	XM_001053876 /// XM_001067401 /// XM_235553	2q25 /// 7q34	2.40	0.000
1376969_at	Transcribed locus	---	627	1505	BI275077	---	2.40	0.037
1393738_s_at	malignant fibrous histiocytoma amplified sequence 1 (predicted)	Mfhas1_predicted	576	1382	NM_001107316 /// XM_001058880 /// XM_224916	16q12.2	2.40	0.002
1375852_at	3-hydroxy-3-methylglutaryl-Coenzyme A reductase	Hmgcr	1695	4067	NM_013134	2q12	2.40	0.000
1367929_at	CD59 antigen	Cd59	9949	23857	NM_012925	3q32	2.40	0.000
1374774_at	Protein tyrosine phosphatase, non-receptor type 14 (predicted)	Ptpn14_predicted	337	806	NM_001107200 /// XM_001066141 /// XM_223062	13q27	2.39	0.024
1386569_at	Transcribed locus	---	462	1105	BF563922	---	2.39	0.000
1372225_at	centrosome-associated protein 350	Cap350	479	1146	XM_001067472 /// XM_341136	13q21	2.39	0.000
1373501_at	NIMA (never in mitosis gene a)-related expressed kinase 7 (predicted)	Nek7_predicted	566	1353	NM_001108346 /// XM_001066105 /// XM_341127	13q13	2.39	0.000
1398969_at	Transcribed locus	---	2560	6118	BE104323	1	2.39	0.001
1388751_at	CAP, adenylate cyclase-associated protein, 2 (yeast)	Cap2	806	1925	AW433787	---	2.39	0.000
1387975_at	UDP-glucose ceramide glucosyltransferase	Ugcg	808	1926	NM_031795	5q24	2.38	0.005
1379283_at	Transcribed locus	---	1487	3542	BE117687	---	2.38	0.000
1373149_at	similar to yippee-like 3 (predicted)	RGD1564579_predicted	394	937	XM_001080016 /// XM_215057	1q36	2.38	0.000
1383169_at	Transcribed locus	---	1623	3853	BF563716	---	2.37	0.001
1373120_at	Transcribed locus, strongly similar to NP_446127.3 spermatogenesis associated 2 [Rattus norvegicus]	---	364	864	BM389931	3	2.37	0.000
1373459_at	serine/threonine kinase 11 interacting protein (predicted)	Stk11ip_predicted	522	1239	NM_001106922 /// XM_001058404 /// XM_237307	9q33	2.37	0.001
1369690_at	N-ethylmaleimide sensitive fusion protein	Nsf	983	2332	NM_021748	10q32.1	2.37	0.006

Table S4. Trophoblast differentiation-associated genes

Affymetrix ID	Gene Title	Gene Symbol	Stem Ave	Dif Ave	GenBank	Chromosomal Location	dif/ stem	t-test
1382884_at	Transcribed locus, strongly similar to XP_001006562.1 PREDICTED: similar to phosphatase subunit gene g4-1 [Mus musculus]	---	346	820	AI009813	---	2.37	0.000
1372040_at	calmodulin binding transcription activator 2 (predicted)	Camta2_predicted	356	845	NM_001105801 /// XM_001079889 /// XM_213362	10q24	2.37	0.011
1387035_a_at	Rho GTPase activating protein 17	Arhgap17	1371	3248	NM_022244	1q36	2.37	0.000
1383742_at	sorting nexin 7	Snx7	417	988	NM_001012083	2q41	2.37	0.000
1398332_at	Transcribed locus, strongly similar to XP_579788.1 PREDICTED: hypothetical protein XP_579788 [Rattus norvegicus]	---	780	1844	BM386294	11	2.37	0.002
1373375_at	Rab6 interacting protein 1 (predicted)	Rab6ip1_predicted	808	1910	BI275835	1	2.36	0.001
1370286_at	solute carrier family 38, member 2	Slc38a2	4742	11201	NM_181090	7q34	2.36	0.002
1377112_at	cytidine deaminase (predicted)	Cda_predicted	513	1211	NM_001108688 /// XM_001070553 /// XM_342955	5q36	2.36	0.000
1367789_at	solute carrier family 27 (fatty acid transporter), member 1	Slc27a1	489	1154	NM_053580	16p14	2.36	0.023
1383679_at	ring finger protein 31 (predicted)	Rnf31_predicted	357	842	NM_001108868 /// XM_001059460 /// XM_001059524 /// XM_344409	15p13	2.36	0.005
1367657_at	B-cell translocation gene 1, anti-proliferative	Btg1	4285	10088	NM_017258	7q13	2.35	0.011
1372592_at	histone deacetylase 6	Hdac6	503	1183	XM_001057931 /// XM_228753	Xq13	2.35	0.002
1382245_at	Clone UI-R-FJ0-cpv-n-11-0-UI unknown mRNA	---	1374	3232	BF397964	---	2.35	0.001
1377664_at	KARP-1 binding protein 1	Kab	769	1806	NM_134416	13q25	2.35	0.000
1387788_at	Jun-B oncogene	Junb	1974	4638	NM_021836	19q11	2.35	0.000
1374719_at	Transcribed locus, strongly similar to XP_228025.3 PREDICTED: similar to ICBP90 binding protein 1 [Rattus norvegicus]	---	556	1306	BF285339	---	2.35	0.001
1389728_at	Meis1, myeloid ecotropic viral integration site 1 homolog 2 (predicted)	Meis2_predicted	483	1133	NM_001107758 /// XM_001080637 /// XM_230449	3q35	2.34	0.000
1371644_at	protein tyrosine kinase 9	Ptk9	3225	7537	NM_001008521	7q35	2.34	0.000
1377410_at	E2F transcription factor 8	E2f8	684	1597	XM_001080259 /// XM_001080267 /// XM_218601	1q22	2.34	0.000
1373672_at	Similar to SPI6	MGC94010	6343	14802	NM_001007732	17p12	2.33	0.003
1389001_at	karyopherin (importin) alpha 4	Kpna4	3800	8855	AW433944	2	2.33	0.000
1393956_at	serine/threonine kinase 17b (apoptosis-inducing)	Stk17b	830	1933	NM_133392	9q31	2.33	0.004
1392368_at	---	---	506	1178	BE117273	---	2.33	0.000
1374490_at	thyroid hormone receptor interactor 12	Trip12	485	1130	NM_001031659	9q35	2.33	0.000
1395725_at	Similar to intracellular membrane-associated calcium-independent phospholipase A2 gamma (predicted)	RGD1311444_predicted	856	1993	NM_001108020 /// XM_001078026 /// XM_234092	6q21	2.33	0.000
1388330_at	vitamin K epoxide reductase complex, subunit 1	Vkorc1	787	1832	NM_203335	1q36	2.33	0.000
1377750_at	Rho guanine nucleotide exchange factor (GEF) 3 (predicted)	Arhgef3_predicted	499	1160	NM_001106061 /// XM_001055379 /// XM_224588	16p16	2.33	0.002

Table S4. Trophoblast differentiation-associated genes

Affymetrix ID	Gene Title	Gene Symbol	Stem Ave	Dif Ave	GenBank	Chromosomal Location	dif/ stem	t-test
1370192_at	syntaxin 12	Stx12	1487	3458	NM_022939	5q36	2.32	0.001
1393186_at	similar to RIKEN cDNA 2010200O16 (predicted)	RGD1564454_predicted	684	1586	XM_001071753 /// XM_575014	2q34	2.32	0.000
1373457_at	inhibitor of growth family, member 1	Ing1	767	1776	NM_001038591	16q12.5	2.32	0.015
1380408_at	Transcribed locus	---	1751	4057	BI290397	---	2.32	0.006
1373059_at	ankyrin repeat domain 13	Ankrd13	2975	6890	NM_001012148	12q16	2.32	0.000
1383062_at	glutaredoxin 2 (thioltransferase)	Glrx2	849	1966	BE105102	13	2.32	0.001
1373520_at	CDNA clone IMAGE:7367270	---	420	972	BI285960	---	2.31	0.001
1371805_at	ankyrin repeat domain 46	Ankrd46	400	925	NM_001013948	7q22	2.31	0.000
1373072_at	Transcribed locus	---	1821	4211	AI170552	---	2.31	0.000
1373305_at	sorting nexin 4 (predicted)	Snx4_predicted	879	2033	XM_001072295 /// XM_340997	11q22	2.31	0.000
1383093_at	---	---	540	1246	AI029437	---	2.31	0.005
1376650_at	golgi autoantigen, golgin subfamily a, 5	Golga5	514	1186	NM_001033065	6q32	2.31	0.000
1369737_at	cAMP responsive element modulator	Crem	412	951	NM_001110860 /// NM_013086 /// NM_017334	17q12.1	2.31	0.004
1377390_at	beta-site APP-cleaving enzyme 2	Bace2	1678	3869	NM_001002802	11q12	2.31	0.003
1389521_at	influenza virus NS1A binding protein (predicted)	Ivns1abp_predicted	2569	5922	NM_001047085 /// XM_001064851 /// XM_001065579 /// XM_001065640 /// XM_213898	13q21	2.31	0.016
1385719_at	Transcribed locus	---	349	805	AW531176	---	2.31	0.001
1390177_at	Transcribed locus	---	554	1278	AI233857	---	2.30	0.002
1367979_s_at	cytochrome P450, subfamily 51	Cyp51	1385	3190	NM_012941	4	2.30	0.002
1374652_at	solute carrier family 25, member 26 (predicted)	Slc25a26_predicted	689	1587	BF285372	4	2.30	0.008
1371547_at	similar to C10 (predicted)	RGD1309695_predicted	1291	2971	BI282112	4	2.30	0.005
1391348_at	pantothenate kinase 3 (predicted)	Pank3_predicted	373	859	NM_001108272 /// XM_001066086 /// XM_340785	10q12	2.30	0.005
1367595_s_at	beta-2 microglobulin	B2m	6965	16016	NM_012512	3q35	2.30	0.005
1388217_a_at	calumenin	Calu	2667	6129	NM_001033898 /// NM_022535	4q22	2.30	0.001
1384290_at	Retinoblastoma binding protein 6	Rbbp6	501	1151	XM_001073098 /// XM_001076339 /// XM_001076360 /// XM_219296	1q36	2.30	0.006
1371038_at	CCAAT/enhancer binding protein (C/EBP), gamma	Cebpg	481	1105	NM_012831 /// XM_001079636 /// XM_341842	1q21	2.30	0.014
1383519_at	Hexokinase 2	Hk2	1722	3949	NM_012735	4q34	2.29	0.004
1374387_at	ADP-ribosylation factor-like 6 interacting protein 5	Arl6ip5	1018	2333	NM_023972	4q34	2.29	0.000
1368076_at	von Hippel-Lindau syndrome homolog	Vhl	1350	3093	NM_052801	4q41.3-q42.1	2.29	0.000
1373533_at	---	---	759	1740	AI412460	---	2.29	0.001
1384560_x_at	placenta specific homeobox 1 (predicted)	Psx1_predicted	1973	4512	XM_001076864 /// XM_231064	3p13	2.29	0.000
1372244_at	calcium binding protein 39 (predicted)	Cab39_predicted	1164	2662	NM_001106924 /// XM_001063411 /// XM_217464	9q35	2.29	0.001

Table S4. Trophoblast differentiation-associated genes

Affymetrix ID	Gene Title	Gene Symbol	Stem Ave	Dif Ave	GenBank	Chromosomal Location	dif/ stem	t-test
1373289_at	ankyrin repeat and SOCS box-containing protein 8 (predicted)	Asb8_predicted	576	1316	NM_001108109 /// XM_001059760 /// XM_235618	7q36	2.29	0.007
1379377_at	ganglioside-induced differentiation-associated-protein 2	Gdap2	596	1362	NM_001013201	2q34	2.29	0.000
1376073_at	Sel1 (suppressor of lin-12) 1 homolog (C. elegans)	Sel1h	534	1219	NM_177933	6q31	2.28	0.002
1371382_at	filamin, alpha (predicted)	Flna_predicted	958	2185	XM_001056685 /// XM_238167	Xq37	2.28	0.030
1389571_at	signal transducer and activator of transcription 2	Stat2	358	816	NM_001011905	7q11	2.28	0.001
1397174_at		Kiaa0415	551	1256	AW520453	---	2.28	0.000
1372487_at	hypothetical protein LOC619573	LOC619573	389	886	NM_001034958	10q32	2.28	0.000
1398421_at	nuclear factor of activated T-cells 5 (predicted)	Nfat5_predicted	1037	2361	NM_001107425 /// XM_001076581 /// XM_226436	19q12	2.28	0.001
1368908_at	annexin A4	Anxa4	1620	3682	NM_024155	4q34	2.27	0.000
1390628_at	cytoplasmic polyadenylation element binding protein 2 (predicted)	Cpeb2_predicted	1195	2714	NM_001108361 /// XM_001060239 /// XM_341227	14q21	2.27	0.001
1375858_at	tumor suppressor candidate 4	Tusc4	612	1388	NM_001025744	8q32	2.27	0.010
1382420_at	similar to hypothetical protein FLJ14681 (predicted)	RGD1307907_predicted	373	845	XM_001081122 /// XM_342515	3q36	2.26	0.001
1372890_at	sphingosine phosphate lyase 1	Sgpl1	596	1348	BM389696	20	2.26	0.000
1373488_at	Transcribed locus	---	1585	3583	BF289154	---	2.26	0.000
1397618_at	sperm associated antigen 9 (predicted)	Spag9_predicted	553	1249	NM_001108290 /// XM_001081278 /// XM_340879	10q26	2.26	0.009
1388659_at	calcium regulated heat stable protein 1	Carhsp1	380	856	NM_152790	10q12	2.26	0.001
1398780_at	Rab acceptor 1 (prenylated)	Rabac1	989	2230	NM_031774	1q21	2.25	0.000
1389146_at	hypothetical protein LOC498796	LOC498796	2467	5559	NM_001025034	17q12.3	2.25	0.000
1389047_at	Similar to Bcl2-associated athanogene 2	LOC690038	1721	3879	XM_001073000	9q21	2.25	0.000
1376620_at	Transcribed locus	---	669	1505	AI137912	---	2.25	0.008
1387072_at	protein kinase, lysine deficient 1	Prkwnk1	1204	2705	NM_053794	4q42	2.25	0.000
1368842_at	transcription factor 4	Tcf4	592	1329	NM_053369	18q12.1	2.24	0.012
1398374_at	similar to RIKEN cDNA 2810002D13 gene	RGD1307128	466	1047	NM_001012354	3q35	2.24	0.000
1382070_at	activating transcription factor 7 interacting protein (predicted)	Atf7ip_predicted	467	1046	AA819758	4	2.24	0.000
1380383_at	ADP-ribosylation factor 4-like (predicted)	Arf4l_predicted	576	1291	NM_001107052 /// XM_001081491 /// XM_220933	10q32.1	2.24	0.000
1375130_at	hypothetical protein LOC679612 /// hypothetical protein LOC687408	LOC679612 /// LOC687408	396	888	XM_001053736 /// XM_001078416	1q51	2.24	0.000
1388868_at	zinc finger protein 216 (predicted)	Zfp216_predicted	3844	8611	NM_001106356 /// XM_001079764 /// XM_215251	1q51	2.24	0.000
1384584_at	Transcribed locus, moderately similar to NP_007227.1 cytochrome c oxidase subunit I [Rattus norvegicus]	---	390	873	AA892774	---	2.24	0.000
1390648_at	hect (homologous to the E6-AP (UBE3A) carboxyl terminus) domain and RCC1 (CHC1)-like domain (RLD) 2 (predicted)	Herc2_predicted	522	1168	NM_001107520 /// XM_001054649 /// XM_218720	1q22	2.24	0.002
1387432_at	ROD1 regulator of differentiation 1 (S. pombe)	Rod1	929	2076	NM_031346	5q24	2.24	0.013

Table S4. Trophoblast differentiation-associated genes

Affymetrix ID	Gene Title	Gene Symbol	Stem Ave	Dif Ave	GenBank	Chromosomal Location	dif/ stem	t-test
1392536_at	Transcribed locus	---	447	999	AI136839	---	2.23	0.000
1388947_at	eukaryotic translation initiation factor 5B /// similar to Eukaryotic translation initiation factor 5B (eIF-5B) (Translation initiation factor IF-2)	Eif5b /// LOC689581	3312	7388	NM_001110141 /// XM_001055509 /// XM_001057591 /// XM_001057637 /// XM_001071498 /// XM_001071547 /// XM_001075051 /// XM_001075073 /// XM_218162	19q12 /// 9q21	2.23	0.001
1372863_at	pam, highwire, rpm 1 (predicted)	Phr1_predicted	2187	4877	NM_001106055 /// XM_001076799 /// XM_214245	15q22	2.23	0.005
1392973_at	Transcribed locus, strongly similar to XP_579919.1 PREDICTED: hypothetical protein XP_579919 [Rattus norvegicus]	---	480	1070	AI073173	---	2.23	0.000
1368486_at	insulin receptor substrate 3	Irs3	1104	2459	NM_032074	12q12	2.23	0.000
1374884_at	protein phosphatase 1D magnesium-dependent, delta isoform (predicted)	Ppm1d_predicted	441	982	NM_001105825 /// XM_001081120 /// XM_213418	10q26	2.23	0.001
1388995_at	ring finger protein 14	Rnf14	1779	3960	XM_001066493	18p11	2.23	0.000
1388383_at	Transcribed locus	---	1135	2522	AI412740	---	2.22	0.000
1398360_at	similar to Elongation of very long chain fatty acids protein 1	LOC679532	1089	2418	NM_001044275 /// XM_001053380 /// XM_001072419	5q36	2.22	0.000
1371978_at	oxysterol binding protein-like 9 (predicted)	Osbpl9_predicted	1034	2296	XM_001061614 /// XM_001061772 /// XM_216477	5q35	2.22	0.001
1376440_at	ring finger protein 139 (predicted)	Rnf139_predicted	682	1513	XM_235338	7q33	2.22	0.000
1371664_at	paxillin	Pxn	3008	6674	NM_001012147	12q16	2.22	0.000
1384366_at	COP9 (constitutive photomorphogenic) homolog, subunit 2 (Arabidopsis thaliana)	Cops2	1173	2601	BI283477	3	2.22	0.000
1381542_at	UBX domain containing 2	Ubxd2	806	1785	NM_001012025	13q12	2.21	0.000
1373874_at	sphingosine-1-phosphate phosphatase 1	Sgpp1	2206	4880	XM_001080791 /// XM_343081	6q24	2.21	0.000
1389345_at	Transcribed locus	---	872	1929	AI231218	---	2.21	0.016
1387087_at	CCAAT/enhancer binding protein (C/EBP), beta	Cebpb	2235	4935	NM_024125	3q42	2.21	0.000
1379439_at	BTB (POZ) domain containing 15	Btbd15	375	828	NM_001034942	8q13	2.21	0.013
1398927_at	similar to 0610010K06Rik protein	RGD1307161	2366	5216	NM_001025669	13q26	2.20	0.000
1390432_at	transmembrane and coiled-coil domains 3 (predicted)	Tmco3_predicted	737	1625	XM_001075325 /// XM_225016	16q12.5	2.20	0.000
1389481_at	similar to GI:13385412-like protein splice form I	RGD735065	487	1074	NM_199379	20p12	2.20	0.008
1374682_at	SRY (sex determining region Y)-box 13 (predicted)	Sox13_predicted	1450	3193	NM_001105952 /// XM_001060642 /// XM_222636	13q13	2.20	0.000
1378896_at	solute carrier family 30 (zinc transporter), member 2	Slc30a2	634	1394	NM_001083122 /// NM_012890	5q36	2.20	0.035
1368323_at	tissue factor pathway inhibitor	Tfpi	17979	39474	NM_017200	3q24	2.20	0.006
1376347_at	similar to PLU1 (predicted)	RGD1565602_predicted	864	1896	NM_001107177 /// XM_001062221 /// XM_001062340 /// XM_239761	13q13	2.20	0.001
1370172_at	superoxide dismutase 2, mitochondrial	Sod2	1639	3598	NM_017051	1q21	2.19	0.000

Table S4. Trophoblast differentiation-associated genes

Affymetrix ID	Gene Title	Gene Symbol	Stem Ave	Dif Ave	GenBank	Chromosomal Location	dif/ stem	t-test
1375882_at	protein phosphatase 3, catalytic subunit, beta isoform	Ppp3cb	645	1416	NM_017042	15p16	2.19	0.001
1388308_at	hypothetical protein LOC687090	LOC687090	1008	2204	NM_001110318 /// XR_009131	---	2.19	0.004
1392475_at	Transcribed locus	---	638	1396	BE104169	---	2.19	0.000
1372871_at	hypothetical protein MGC:72616	RGD735175	493	1077	NM_199112	9q33	2.19	0.009
1399102_at	similar to hypothetical protein MGC15523	RGD1306356	823	1797	XM_001081798 /// XM_001081800 /// XM_221195	10q32.3	2.18	0.000
1385314_at	RAB14, member RAS oncogene family	Rab14	877	1914	AA923974	3	2.18	0.003
1392531_at	similar to 3110080A02Rik protein (predicted)	RGD1305754_predicted	667	1456	NM_001106537 /// XM_001069672 /// XM_230830	3q42	2.18	0.001
1392806_at	Wolf-Hirschhorn syndrome candidate 1-like 1 (predicted)	Whsc1l1_predicted	587	1279	AI234807	---	2.18	0.003
1382027_at	integrin beta 3	Itgb3	1478	3211	BI296880	---	2.17	0.001
1373812_at	Cyclin-dependent kinase inhibitor 1B	Cdkn1b	559	1214	NM_031762	4q43	2.17	0.029
1374413_at	ubiquitin-like 3	Ubl3	1337	2897	NM_001015030	12p11	2.17	0.000
1383197_at	junction adhesion molecule 2	Jam2	812	1759	NM_001034004	11q11	2.17	0.018
1385228_x_at	development and differentiation enhancing (predicted)	Ddef1_predicted	941	2038	AI059117	7	2.17	0.018
1374544_at	hypothetical protein LOC679150 /// hypothetical protein LOC690899	LOC679150 /// MGC112715	1398	3024	NM_001044300 /// XM_001055448 /// XM_001073575	20q11	2.16	0.048
1391572_at	cysteinyl-tRNA synthetase (predicted)	Cars_predicted	1091	2361	NM_001106319 /// XM_001065753 /// XM_215134	1q41	2.16	0.000
1395454_at	similar to osteopetrosis associated transmembrane protein 1	LOC499474	550	1190	NM_001029925	20q13	2.16	0.025
1387753_s_at	membrane associated guanylate kinase, WW and PDZ domain containing 3	Magi3	620	1337	NM_139084 /// XM_001068223	2q34	2.16	0.000
1372082_at	NECAP endocytosis associated 2	Necap2	1227	2647	NM_199096	5q36	2.16	0.003
1372528_at	N-ethylmaleimide sensitive fusion protein	Nsf	985	2124	NM_021748	10q32.1	2.16	0.001
1373509_at	NOL1/NOP2/Sun domain family, member 4 (predicted)	Nsun4_predicted	425	915	NM_001106678 /// XM_001070636 /// XM_238407	5q36	2.16	0.001
1375979_at	Transcribed locus	---	841	1813	AA945734	6	2.15	0.000
1372990_at	cAMP responsive element binding protein 3	Creb3	1115	2402	NM_001013092	5q22	2.15	0.001
1369141_at	Prolactin family 3, subfamily d, member 1 /// Prolactin family 3, subfamily d, member 2	Prl3d1 /// Prl3d2	10452	22492	NM_001083940 /// NM_017363 /// XM_001078794	17p11	2.15	0.000
1389333_at	similar to F-box only protein 3 isoform 1	LOC690634	2031	4370	NM_001109606 /// XM_001064070 /// XM_001064129 /// XM_001072621 /// XM_001072658	3q32	2.15	0.000
1382199_at	microtubule-associated proteins 1A/1B light chain 3	Map1lc3b	908	1953	BF288776	19	2.15	0.043
1374599_at	hect (homologous to the E6-AP (UBE3A) carboxyl terminus) domain and RCC1 (CHC1)-like domain (RLD) 1 (predicted)	Herc1_predicted	688	1478	XM_001075834 /// XM_236362	8q24	2.15	0.002
1375714_at	similar to Erbb2 interacting protein isoform 2 (predicted)	RGD1562952_predicted	536	1147	XM_001072232	2q12-q13	2.14	0.000
1367974_at	annexin A3	Anxa3	2177	4660	NM_012823	14p22	2.14	0.000

Table S4. Trophoblast differentiation-associated genes

Affymetrix ID	Gene Title	Gene Symbol	Stem Ave	Dif Ave	GenBank	Chromosomal Location	dif/ stem	t-test
1370174_at	myeloid differentiation primary response gene 116	Myd116	1244	2662	NM_133546	1q22	2.14	0.000
1387169_at	transducin-like enhancer of split 3, E(spl) homolog (Drosophila)	Tle3	908	1942	NM_053400	8q24	2.14	0.006
1376994_at	Transcribed locus, strongly similar to XP_988139.1 PREDICTED: similar to F-box and leucine-rich repeat protein 20 isoform 5 [Mus musculus]	---	442	946	BE098799	---	2.14	0.002
1398481_at	Transcribed locus	---	728	1555	BF402699	---	2.14	0.000
1368946_at	ADP-ribosylation factor 2	Arf2	686	1464	NM_024150	10q32.1	2.13	0.000
1372877_at	procollagen-lysine, 2-oxoglutarate 5-dioxygenase 3	Plod3	1490	3178	NM_178101	12q12	2.13	0.001
1388336_at	similar to hypothetical protein D2Ertd391e (predicted)	RGD1310685_predicted	515	1096	XM_001069036 /// XM_342465	3q24	2.13	0.002
1371872_at	Nucleosome assembly protein 1-like 1	Nap1l1	5723	12181	NM_053561	7q21	2.13	0.000
1390845_at	Transcribed locus	---	386	821	AI179288	---	2.13	0.002
1389304_at	RE1-silencing transcription factor	Rest	2866	6097	AI176323	14	2.13	0.000
1399037_at	similar to Tetratricopeptide repeat protein 19 (TPR repeat protein 19)	LOC687312 /// LOC691506	546	1161	NM_001109644 /// XM_001077297 /// XM_001077548	10q23	2.13	0.000
1397670_at	autophagy-related 12 (yeast)	Atg12	842	1790	NM_001038495	18q11	2.13	0.000
1392471_at	guanine nucleotide binding protein (G protein), gamma 12	Gng12	1190	2528	BI302750	4	2.12	0.003
1374058_at	TMEM9 domain family, member B (predicted)	Tmem9b_predicted	632	1344	NM_001106289 /// XM_001073254 /// XM_215038	1q33	2.12	0.001
1375221_at	thioredoxin domain containing 13	Txndc13	1423	3021	XM_001081259 /// XM_215853	3q36	2.12	0.001
1393341_at	Transcribed locus	---	1248	2649	AA955466	---	2.12	0.003
1384331_at	sulfiredoxin 1 homolog (S. cerevisiae)	Srxn1	615	1306	NM_001047858 /// XM_001058481 /// XM_215887	3q41	2.12	0.007
1389297_at	ERO1-like (S. cerevisiae)	Ero1l	2562	5437	NM_138528	15p14	2.12	0.001
1389478_at	kelch-like 22 (Drosophila) (predicted)	Klhl22_predicted	1923	4075	NM_001107079 /// XM_001063385 /// XM_221268	11q23	2.12	0.000
1382332_at	stromal antigen 2 (predicted)	Stag2_predicted	1824	3864	XM_001059059 /// XM_233108	Xq11	2.12	0.000
1393024_at	Transcribed locus	---	459	973	BI300589	---	2.12	0.000
1393226_at	Transcribed locus	---	883	1869	BI275559	---	2.12	0.000
1371127_at	bone morphogenetic protein 1	Bmp1	432	914	XM_001070483 /// XM_001070524 /// XM_001075543 /// XM_573814	15p11	2.12	0.002
1399153_at	RAB5B, member RAS oncogene family (predicted)	Rab5b_predicted	447	946	NM_001079936 /// XM_001071247 /// XM_213824	7q11	2.12	0.001
1387964_a_at	ERO1-like (S. cerevisiae)	Ero1l	437	924	NM_138528	15p14	2.12	0.019
1385540_at	protein inhibitor of activated STAT 1 (predicted)	Pias1_predicted	553	1169	NM_001106829 /// XM_001074210 /// XM_217188	8q24	2.11	0.001
1388874_at	metastasis suppressor 1 (predicted)	Mtss1_predicted	665	1405	XM_001064860 /// XM_343248	7q33	2.11	0.000
1387653_at	translin-associated factor X	Tsnax	606	1280	NM_022262	19	2.11	0.001
1389444_at	Similar to RIKEN cDNA 5033406L14	RGD1305755	757	1597	NM_001039336	15p12	2.11	0.027

Table S4. Trophoblast differentiation-associated genes

Affymetrix ID	Gene Title	Gene Symbol	Stem Ave	Dif Ave	GenBank	Chromosomal Location	dif/ stem	t-test
1389194_at	Transcribed locus, strongly similar to NP_001020450.1 hypothetical protein LOC306014 [Rattus norvegicus]	---	554	1168	AI406491	---	2.11	0.003
1379560_at	nuclear receptor binding SET domain protein 1 (predicted) /// similar to Histone-lysine N-methyltransferase, H3 lysine-36 and H4 lysine-20 specific (H3-K36-HMTase) (H4-K20-HMTase) (Nuclear receptor binding SET domain containing protein 1) (NR-binding SET domain containing protein)	LOC686060 /// Nsd1_predicted	594	1251	NM_001107337 /// XM_001066374 /// XM_001066409 /// XM_225168	17p14	2.11	0.006
1377964_at	similar to RIKEN cDNA 2010200O16 (predicted)	RGD1564454_predicted	789	1662	XM_001071753 /// XM_575014	2q34	2.11	0.001
1384929_at	similar to apolipoprotein B48 receptor (predicted)	RGD1565169_predicted	830	1749	NM_001109154 /// XM_001079912 /// XM_574561	1q36	2.11	0.000
1398935_at	similar to Sorting nexin-9	LOC683687	1075	2263	XM_001067064	---	2.10	0.002
1393268_at	Transcribed locus	---	688	1447	AI071071	---	2.10	0.027
1369496_at	protein tyrosine phosphatase, non-receptor type 12	Ptpn12	786	1651	NM_057115	4q11	2.10	0.000
1378987_at	Transcribed locus	---	414	870	BE119446	---	2.10	0.002
1375315_at	similar to hypothetical protein BC013035 (predicted)	RGD1560996_predicted	481	1008	BE098345	16	2.10	0.001
1367826_at	nuclear factor, erythroid derived 2, like 2	Nfe2l2	2960	6192	NM_031789	3q23	2.09	0.000
1383698_at	pyruvate dehydrogenase E1 alpha 1	Pdha1	518	1082	NM_001004072	Xq21	2.09	0.005
1374422_at	similar to Erbb2 interacting protein isoform 2 (predicted)	RGD1562952_predicted	498	1042	XM_001072232	2q12-q13	2.09	0.001
1372410_at	C1q and tumor necrosis factor related protein 6	C1qtnf6	2364	4935	NM_001034932	7q34	2.09	0.001
1381018_a_at	development and differentiation enhancing (predicted)	Ddef1_predicted	458	956	BG378709	7	2.09	0.032
1392472_at	similar to myocyte enhancer factor 2C	LOC309957	446	931	NM_001014035	2q11	2.09	0.001
1387346_at	integrin beta 1 (fibronectin receptor beta)	Itgb1	2875	5994	NM_017022	19q12	2.09	0.003
1376754_at	cysteinyl-tRNA synthetase (predicted)	Cars_predicted	920	1916	NM_001106319 /// XM_001065753 /// XM_215134	1q41	2.08	0.000
1387436_at	septin 7	Sept7	3327	6930	NM_022616	8q13	2.08	0.007
1397179_at	similar to DIP13 alpha (predicted)	RGD1309388_predicted	404	841	XR_007603	16p16	2.08	0.002
1371857_at	potassium channel tetramerization domain containing 10	Kctd10	1008	2098	NM_001009973	---	2.08	0.000
1371338_at	presenilin enhancer 2 homolog (C. elegans)	Psenen	849	1767	NM_001008764	1q21	2.08	0.001
1377999_at	tropomodulin 3 (predicted)	Tmod3_predicted	1175	2442	BE103242	8	2.08	0.020
1398512_at	nuclear transcription factor, X-box binding 1	Nfx1	774	1608	XM_001059354 /// XM_001059410 /// XM_001059469 /// XM_001064398 /// XM_001064460	5q22	2.08	0.003
1389028_at	nuclear receptor coactivator 6	Ncoa6	1491	3091	XM_001064071 /// XM_001064189 /// XM_342552	3q42	2.07	0.002
1368365_at	aldehyde dehydrogenase family 3, subfamily A2	Aldh3a2	906	1878	NM_031731	10q22	2.07	0.000
1368702_at	PRKC, apoptosis, WT1, regulator	Pawr	771	1599	NM_033485	7q21	2.07	0.002

Table S4. Trophoblast differentiation-associated genes

Affymetrix ID	Gene Title	Gene Symbol	Stem Ave	Dif Ave	GenBank	Chromosomal Location	dif/ stem	t-test
1390385_at	similar to D-glucuronyl C5-epimerase (predicted)	RGD1565253_predicted	847	1755	XM_001073932 /// XM_343404	8q24	2.07	0.000
1389534_at	ubiquitin-conjugating enzyme E2E 3, UBC4/5 homolog (yeast) (predicted)	Ube2e3_predicted	5666	11740	NM_001047857 /// XM_001067181 /// XM_215754	3q24	2.07	0.003
1388161_at	a disintegrin and metalloprotease domain 10	Adam10	569	1179	XM_001054737 /// XM_217197	8q24	2.07	0.010
1369644_at	latrophilin 2	Lphn2	2974	6158	NM_134408	2q45	2.07	0.000
1399049_at	---	---	1961	4057	AI230073	6	2.07	0.002
1374405_at	heterogeneous nuclear ribonucleoprotein A3	Hnrpa3	1498	3100	NM_001111294 /// NM_001111295 /// NM_198132	3q23	2.07	0.003
1371979_at	sterol regulatory element binding factor 2 (predicted)	Srebf2	661	1367	NM_001033694	7q34	2.07	0.001
1388733_at	bifunctional apoptosis regulator	Bfar	807	1668	NM_001013125	10q11	2.07	0.001
1376640_at	Hypothetical protein LOC680259	LOC680259	2157	4457	XM_001056358	17q12.3	2.07	0.001
1380019_at	T-cell, immune regulator 1	Tcirg1	524	1081	NM_199089	1q42	2.06	0.002
1368770_at	glucosaminyl (N-acetyl) transferase 1, core 2	Gcnt1	1044	2155	NM_022276	1q43	2.06	0.001
1367946_at	PDZ and LIM domain 1 (elfin)	Pdlim1	4580	9449	NM_017365	1q53	2.06	0.001
1385658_at	zinc finger protein 313	Zfp313	1262	2602	NM_001001517	3q42	2.06	0.000
1376658_at	Ras association (RalGDS/AF-6) and pleckstrin homology domains 1 (predicted)	Raph1_predicted	416	858	NM_001108798 /// XM_001070281 /// XM_343579	9q31	2.06	0.004
1372510_at	sulfiredoxin 1 homolog (S. cerevisiae)	Srxn1	1104	2274	NM_001047858 /// XM_001058481 /// XM_215887	3q41	2.06	0.040
1388542_at	similar to RIKEN cDNA 2010315L10 (predicted) /// similar to USE1-like protein (Hematopoietic stem/progenitor cells protein MDS032) (Putative MAPK-activating protein PM26) (Protein p31)	LOC292282 /// RGD1306660_predicted	548	1128	XM_001065114 /// XM_214304 /// XM_214765	16p14	2.06	0.001
1373115_at	similar to 2310061I09Rik protein (predicted)	RGD1304653_predicted	652	1341	BI281909	9	2.06	0.001
1375936_at	desmocollin 2	Dsc2	1659	3414	NM_001033688	18p12	2.06	0.001
1372020_at	target of myb1 homolog (chicken)	Tom1	500	1029	NM_001008365	19p11	2.06	0.000
1374075_at	N-ethylmaleimide-sensitive factor attachment protein, gamma	Napg	511	1051	NM_001107384 /// XM_001063511 /// XM_225881	18q12.1	2.06	0.000
1384163_at	Transcribed locus	---	1059	2175	BE103875	---	2.05	0.000
1374034_at	cysteinyl-tRNA synthetase (predicted)	Cars_predicted	1339	2750	NM_001106319 /// XM_001065753 /// XM_215134	1q41	2.05	0.000
1381019_x_at	development and differentiation enhancing (predicted)	Ddef1_predicted	583	1198	BG378709	7	2.05	0.007
1397634_at	ankyrin repeat and SAM domain containing 1 (predicted)	Anks1_predicted	1804	3701	NM_001107613 /// XM_001078242 /// XM_228027	20p12	2.05	0.007
1388635_at	similar to hypothetical protein FLJ20507 (predicted)	RGD1309744_predicted	531	1090	NM_001100978 /// XM_001057254 /// XM_001081091 /// XM_230574	3q36	2.05	0.010
1370141_at	myeloid cell leukemia sequence 1	Mcl1	2334	4788	NM_021846	---	2.05	0.001
1372909_at	similar to hypothetical protein FLJ36090 (predicted)	RGD1565619_predicted	612	1256	XM_001055603 /// XM_225760	18q11	2.05	0.000

Table S4. Trophoblast differentiation-associated genes

Affymetrix ID	Gene Title	Gene Symbol	Stem Ave	Dif Ave	GenBank	Chromosomal Location	dif/ stem	t-test
1398866_at	membrane associated guanylate kinase, WW and PDZ domain containing 3	Magi3	1989	4080	NM_139084 /// XM_001068223	2q34	2.05	0.001
1398375_at	metastasis associated 3 (predicted)	Mta3_predicted	1701	3486	BM392144	4	2.05	0.006
1384376_at	Transcribed locus, strongly similar to XP_980162.1 PREDICTED: similar to DnaJ (Hsp40) homolog, subfamily B, member 14 isoform 1 [Mus musculus]	---	421	862	AA818641	---	2.05	0.012
1374641_at	Transcribed locus	---	1394	2851	BF397653	---	2.05	0.003
1388407_at	similar to BC003940 protein (predicted)	RGD1311925_predicted	714	1458	NM_001108311 /// XM_001081799 /// XM_340949	10q32.3	2.04	0.000
1369912_at	v-crk sarcoma virus CT10 oncogene homolog (avian)	Crk	659	1346	NM_019302	10q24	2.04	0.004
1379500_at	similar to cDNA sequence BC018601	LOC498404	834	1702	NM_001017502	14q21	2.04	0.005
1378925_at	CAMP responsive element modulator	Crem	1912	3897	NM_001110860 /// NM_013086 /// NM_017334	17q12.1	2.04	0.001
1377697_at	similar to amyloid beta (A4) precursor protein-binding, family B, member 2 (predicted)	RGD1562438_predicted	399	813	XM_001077874 /// XM_223399	14p11	2.04	0.001
1371401_at	ing finger protein 5 pseudogene	rCG_38334	961	1958	NM_001109025	20p12	2.04	0.000
1397317_at	Transcribed locus	---	1451	2955	BI296984	---	2.04	0.004
1371322_at	laminin, gamma 1	Lamc1	1275	2596	XM_001071300 /// XM_341133	13q21	2.04	0.001
1396353_at	Transcribed locus, strongly similar to XP_001059486.1 PREDICTED: similar to Protein phosphatase 2A, 59 kDa regulatory subunit B (PP2A PR59) (PP2A B-PR59) [Rattus norvegicus]	---	454	925	BF406671	---	2.04	0.004
1380577_at	ATP-binding cassette, sub-family G (WHITE), member 2	Abcg2	732	1491	NM_181381	4q24	2.04	0.001
1374554_at	similar to HT014 (predicted)	RGD1308048_predicted	3557	7234	XM_001068778 /// XM_216543	5q36	2.03	0.004
1389305_at	annexin A4	Anxa4	2108	4285	NM_024155	4q34	2.03	0.000
1378037_at	similar to RIKEN cDNA 0610009J22 (predicted)	RGD1310738_predicted	647	1314	NM_001107234 /// XM_001065855 /// XM_223565	14q21	2.03	0.001
1388873_at	thyroid hormone receptor interactor 12	Trip12	4960	10078	NM_001031659	9q35	2.03	0.001
1371798_at	guanine nucleotide binding protein, alpha 12	Gna12	604	1228	AI013011	12	2.03	0.001
1369661_at	dynamin 2	Dnm2	406	825	NM_013199	8q13	2.03	0.000
1390250_x_at	similar to Probable phospholipid-transporting ATPase ID (ATPase class I type 8B member 2)	LOC685152	448	908	XM_001062555	2q34	2.03	0.008
1383368_at	Unknown (protein for MGC:72610)	MGC72610	4077	8267	BF550060	---	2.03	0.000
1388813_at	ADP-ribosylation factor 2	Arf2	2117	4293	NM_024150	10q32.1	2.03	0.000
1374117_at	brain-specific angiogenesis inhibitor 1-associated protein 2	Baiap2	599	1215	NM_057196	10q32.3	2.03	0.001
1372568_at	EPM2A (laforin) interacting protein 1 (predicted)	Epm2aip1_predicted	677	1371	XM_001076356 /// XM_236659	8q32	2.03	0.002
1389539_at	similar to DIP13 alpha (predicted)	RGD1309388_predicted	527	1067	XR_007603	16p16	2.03	0.019
1379567_at	thyroid hormone receptor associated protein 1 (predicted)	Thrap1_predicted	678	1372	NM_001107035 /// XM_001081130 /// XM_220813	10q26	2.02	0.036
1367878_at	syntaxin 5a	Stx5a	787	1593	NM_031704	1q43	2.02	0.003

Table S4. Trophoblast differentiation-associated genes

Affymetrix ID	Gene Title	Gene Symbol	Stem Ave	Dif Ave	GenBank	Chromosomal Location	dif/ stem	t-test
1374798_at	Transcribed locus	---	3236	6549	AA924151	---	2.02	0.000
1388218_at	low density lipoprotein receptor	Ldlr	1523	3081	NM_175762	8q13	2.02	0.000
1376727_at	Yip1 domain family, member 4	Yipf4	3098	6267	NM_001009712	6q12	2.02	0.000
1383874_at	RGD1560812 (predicted)	RGD1560812_predicted	525	1062	XM_576015 /// XR_009070	6q16	2.02	0.003
1374536_at	Transcribed locus, strongly similar to XP_579981.1 PREDICTED: hypothetical protein XP_579981 [Rattus norvegicus]	---	835	1687	BG671506	10	2.02	0.002
1377949_s_at	ankyrin repeat and SAM domain containing 1 (predicted)	Anks1_predicted	558	1127	NM_001107613 /// XM_001078242 /// XM_228027	20p12	2.02	0.005
1382196_at	similar to hypothetical protein ET (predicted)	RGD1307394_predicted	483	976	NM_001108308 /// XM_001081752	10q32.3	2.02	0.005
1388401_at	filamin, beta (predicted)	Flnb_predicted	2636	5318	NM_001107288 /// XM_001071051 /// XM_224561	15p14	2.02	0.006
1372023_at	Transcribed locus, strongly similar to XP_579847.1 PREDICTED: hypothetical protein XP_579847 [Rattus norvegicus]	---	604	1217	BI279581	---	2.02	0.000
1370905_at	dedicator of cytokinesis 9	Dock9	1695	3418	NM_001105759 /// XM_001078910 /// XM_224538	15q25	2.02	0.001
1368426_at	carnitine O-octanoyltransferase	Crot	1268	2554	NM_031987	4q12	2.01	0.000
1395165_at	PC4 and SFRS1 interacting protein 1	Psip1	735	1479	NM_175765	5q31	2.01	0.005
1392573_at	ubiquitin-conjugating enzyme E2A, RAD6 homolog (S. cerevisiae)	Ube2a	1102	2219	NM_001013933	Xq12	2.01	0.001
1391558_at	zinc finger protein 608 (predicted)	Zfp608_predicted	1063	2139	NM_001107378 /// XM_001056026 /// XM_225755	18q11	2.01	0.000
1389540_at	similar to IQ motif and Sec7 domain 1	LOC686590	490	987	XM_001072973 /// XM_001073009 /// XM_001073040	---	2.01	0.011
1373071_at	similar to RIKEN cDNA 1810054G18 (predicted)	RGD1308901_predicted	512	1030	NM_001106245 /// XM_001076215 /// XM_214898	1q21	2.01	0.002
1398549_at	Cyclin L2	Ccnl2	484	973	NM_001013094	5q36	2.01	0.001
1391671_at	Hypothetical protein LOC689165	LOC689165	451	905	XM_001069806	17p14	2.01	0.003
1392951_at	platelet-activating factor acetylhydrolase, isoform 1b, alpha2 subunit	Pafah1b2	899	1800	AW521231	---	2.00	0.005
1395147_at	excision repair cross-complementing rodent repair deficiency, complementation group 3	Ercc3	527	1052	BE116565	---	2.00	0.001
1393449_at	junction adhesion molecule 2	Jam2	585	1167	NM_001034004	11q11	2.00	0.019
1367890_at	caspase 2	Casp2	693	1383	NM_022522	4q23	1.99	0.009
1398779_at	actin related protein 2/3 complex, subunit 1A	Arpc1a	2375	4737	NM_031146	12p11	1.99	0.003
1388929_at	Clone UI-R-FJ0-cpv-n-11-0-UI unknown mRNA	---	2097	4180	AI011716	---	1.99	0.000
1372533_at	similar to mKIAA0212 protein (predicted)	RGD1563633_predicted	2023	4032	XM_001075914 /// XM_001075943 /// XM_238366	4q41	1.99	0.000
1371562_at	epsin 2	Epn2	571	1138	NM_001033914 /// NM_021852	10q23	1.99	0.003
1393162_at	solute carrier family 39 (metal ion transporter), member 6	Slc39a6	929	1849	NM_001024745	18p12	1.99	0.003

Table S4. Trophoblast differentiation-associated genes

Affymetrix ID	Gene Title	Gene Symbol	Stem Ave	Dif Ave	GenBank	Chromosomal Location	dif/ stem	t-test
1373864_at	mitogen-activated protein kinase kinase kinase kinase 4 (predicted)	Map4k4_predicted	1137	2263	NM_001106904 /// XM_001058422 /// XM_001058485 /// XM_001058541 /// XM_001058590 /// XM_001058649 /// XM_001058712 /// XM_217381	9q21	1.99	0.000
1373278_at	nuclear factor, erythroid derived 2,-like 1 (predicted)	Nfe2l1_predicted	650	1294	BM391303	10	1.99	0.001
1376465_at	Transcribed locus	---	421	838	BI295240	---	1.99	0.001
1382689_at	similar to D7Ertd743e protein	RGD1310870	1859	3696	NM_001012358	1q33	1.99	0.001
1371325_at	protective protein for beta-galactosidase	Ppgb	3857	7667	NM_001011959	3q42	1.99	0.001
1368826_at	catechol-O-methyltransferase	Comt	1421	2824	NM_012531	11q23	1.99	0.002
1389567_at	SREBP cleavage activating protein (predicted)	Scap_predicted	493	978	NM_001100966 /// XM_001077128 /// XM_217279	8q32	1.99	0.001
1398536_at	zinc finger protein 655	Zfp655	940	1866	NM_001008362	12p11	1.99	0.006
1374418_at	similar to CG8009-PA (predicted)	RGD1308816_predicted	1302	2586	BF289188	19	1.99	0.001
1368217_at	ralA binding protein 1	Ralbp1	1626	3224	NM_032067	9q37	1.98	0.000
1398335_at	tweety homolog 3 (Drosophila) (predicted)	Ttyh3_predicted	443	878	NM_001107124 /// XM_001073693 /// XM_221962	12q11	1.98	0.004
1391719_at	---	---	609	1206	BE098936	---	1.98	0.000
1389965_at	trans-golgi network protein 2	Tgoln2	2293	4540	NM_138840	4q33	1.98	0.000
1398948_at	Tax1 (human T-cell leukemia virus type I) binding protein 1	Tax1bp1	3667	7259	NM_001004199	4q24	1.98	0.000
1384380_at	zinc finger, CCHC domain containing 7 (predicted)	Zcchc7_predicted	486	962	AI535316	---	1.98	0.007
1372776_at	F-box and leucine-rich repeat protein 5 (predicted)	Fbxl5_predicted	1471	2910	NM_001107222 /// XM_001060082 /// XM_223508	14q21	1.98	0.000
1388587_at	immediate early response 3	Ier3	3290	6496	NM_212505	20p12	1.97	0.000
1383837_at	Yip1 domain family, member 3	Yipf3	473	934	NM_001007801	9q12	1.97	0.003
1398998_at	similar to CG4768-PA (predicted)	RGD1309748_predicted	1484	2925	NM_001106972 /// XM_001078661 /// XM_220139	10q12	1.97	0.001
1386994_at	B-cell translocation gene 2, anti-proliferative	Btg2	4782	9422	NM_017259	13	1.97	0.002
1388957_at	CDNA clone IMAGE:7368410	---	857	1688	AA891790	---	1.97	0.000
1374447_at	ubiquitin specific peptidase 9, X chromosome (predicted)	Usp9x_predicted	1597	3144	XM_001056701 /// XM_343766	Xq13	1.97	0.000
1371823_at	stromal antigen 2 (predicted)	Stag2_predicted	2978	5860	XM_001059059 /// XM_233108	Xq11	1.97	0.000
1372885_at	CCAAT/enhancer binding protein (C/EBP), gamma	Cebpg	1138	2238	AI230467	1	1.97	0.003
1389686_at	protein kinase, X-linked	Prkx	517	1018	NM_001033963	Xq22	1.97	0.009
1389573_at	ChaC, cation transport regulator-like 1 (E. coli) (predicted)	Chac1_predicted	422	830	XM_001080834 /// XM_342497	3q35	1.97	0.003
1375960_at	germ cell-less homolog 1 (Drosophila)	Gmcl1	1441	2833	NM_001010956 /// NM_001033931	4q34	1.97	0.000
1383942_at	similar to Sorting nexin-9	LOC683687	3997	7848	XM_001067064	---	1.96	0.001
1393363_at	similar to zinc finger protein 198	LOC305913	609	1195	XM_001060431 /// XM_224241	15p12	1.96	0.002

Table S4. Trophoblast differentiation-associated genes

Affymetrix ID	Gene Title	Gene Symbol	Stem Ave	Dif Ave	GenBank	Chromosomal Location	dif/ stem	t-test
1370928_at	LPS-induced TN factor	Litaf	10984	21526	NM_001105735 /// XM_001078236 /// XM_343856	10q11	1.96	0.014
1375870_a_at	RNA binding motif, single stranded interacting protein 1	Rbms1	1752	3431	NM_001012184	3q21	1.96	0.001
1392517_at	similar to Golgin 45 (Basic leucine zipper nuclear factor 1)	LOC498266	568	1110	NM_001017494	13q22	1.96	0.005
1389189_at	actinin, alpha 1	Actn1	3016	5899	NM_031005	6q24	1.96	0.000
1393707_at	Bcl2-like 2	Bcl2l2	790	1544	NM_021850	15p13	1.95	0.001
1390171_at	similar to hypothetical protein BC008163 (predicted)	RGD1305162_predicted	730	1426	NM_001108686 /// XM_001065867 /// XM_342937	5q36	1.95	0.001
1371353_at	sequestosome 1	Sqstm1	7025	13722	NM_175843 /// NM_181550	10q21	1.95	0.001
1388274_at	brain expressed myelocytomatosis oncogene	Bmyc	654	1278	NM_001013163	3p13	1.95	0.001
1389778_a_at	transcription elongation factor B (SIII), polypeptide 3	Tceb3	974	1899	NM_017103	5q36	1.95	0.001
1371601_at	similar to Endoplasmic reticulum mannosyl-oligosaccharide 1,2-alpha-mannosidase (ER alpha-1,2-mannosidase) (predicted)	RGD1563595_predicted	1051	2049	XM_001077558 /// XM_575086	3p13	1.95	0.000
1387090_a_at	LIM motif-containing protein kinase 2	Limk2	1784	3477	NM_024135	14q21	1.95	0.000
1382556_a_at	similar to DIP13 alpha (predicted)	RGD1309388_predicted	1015	1977	XR_007603	16p16	1.95	0.002
1367680_at	acyl-Coenzyme A oxidase 1, palmitoyl	Acox1	1840	3584	NM_017340	10q32.3	1.95	0.000
1371522_at	similar to mKIAA0945 protein (predicted)	RGD1563127_predicted	590	1150	XM_001080776 /// XM_347035	3q35	1.95	0.009
1387076_at	hypoxia inducible factor 1, alpha subunit	Hif1a	10340	20134	NM_024359	6q24	1.95	0.000
1379346_at	cell division cycle 73, Paf1/RNA polymerase II complex component, homolog (S. cerevisiae)	Cdc73	3839	7472	NM_001024769	13q21	1.95	0.000
1383819_at	COP9 (constitutive photomorphogenic) homolog, subunit 2 (Arabidopsis thaliana)	Cops2	1267	2466	BI292866	3	1.95	0.024
1396262_at	pre-B-cell colony enhancing factor 1	Pbef1	721	1403	NM_177928	6q16	1.94	0.001
1372808_at	similar to Bifunctional methylenetetrahydrofolate dehydrogenase/cyclohydrolase, mitochondrial precursor	LOC680308	1993	3873	NM_001109398 /// XM_001056154 /// XM_001056213 /// XM_001067863 /// XM_001067912	4q34	1.94	0.005
1377849_at	valosin containing protein (p97)/p47 complex interacting protein 1	Vcpip1	478	928	NM_176857	5q11	1.94	0.000
1378524_at	ring finger protein (C3HC4 type) 19 (predicted)	Rnf19_predicted	678	1317	XM_001059414 /// XM_343228	7q22	1.94	0.000
1388468_at	CDC42 small effector 1	Cdc42se1	794	1542	NM_001039044	2q34	1.94	0.000
1389590_at	Transcribed locus	---	995	1932	AA925513	---	1.94	0.000
1385168_at	similar to receptor-interacting factor 1	RGD1306520	714	1385	XM_001067954 /// XM_227573	2q34	1.94	0.001
1371996_at	AE binding protein 2 (predicted)	Aebp2_predicted	1066	2068	NM_001106626 /// XM_001071806 /// XM_216295	4q44	1.94	0.000
1377347_at	forkhead box J3 (predicted)	Foxj3_predicted	1978	3835	NM_001107971 /// XM_001074052 /// XM_233463	5q36	1.94	0.010
1391770_at	Ubiquitin specific peptidase 33	Usp33	583	1130	XM_001068666 /// XM_001080019	2q45	1.94	0.005
1374809_at	Transcribed locus	---	1099	2130	BG672598	---	1.94	0.007
1382993_at	Bcl-2 binding component 3	Bbc3	743	1439	NM_173837	---	1.94	0.000

Table S4. Trophoblast differentiation-associated genes

Affymetrix ID	Gene Title	Gene Symbol	Stem Ave	Dif Ave	GenBank	Chromosomal Location	dif/ stem	t-test
1378027_at	Pvrl3_predicted	poliovirus receptor-related 3 (predicted)	2075	4019	AW525315	11	1.94	0.001
1372497_at	neighbor of Brca1 gene 1	Nbr1	2370	4591	NM_001024765	10q32.1	1.94	0.001
1374729_at	Similar to UV radiation resistance associated	LOC308846	755	1461	NM_001107536 /// XM_001065167 /// XM_218951	1q32	1.94	0.004
1373589_at	myotubularin related protein 3	Mtmr3	713	1380	NM_001012038	14q21	1.94	0.005
1384958_at	Transcribed locus	---	566	1096	BI279112	---	1.94	0.022
1374494_at	Transcribed locus	---	1312	2537	AI137375	17	1.93	0.000
1399071_at	---	---	472	912	BI281808	---	1.93	0.000
1373473_a_at	nucleosome assembly protein 1-like 1	Nap1l1	5166	9974	NM_053561	7q21	1.93	0.000
1389139_at	similar to Ttc15 protein (predicted)	RGD1566054_predicted	648	1249	XM_001071807 /// XM_234029	6q16	1.93	0.000
1377613_at	similar to RIKEN cDNA 5031400M07	RGD1307343	1524	2937	NM_001024988	8q13	1.93	0.000
1395699_at	RIO kinase 3 (yeast) (predicted)	Riok3_predicted	1134	2186	NM_001108423 /// XM_001072670 /// XM_341578	18p13	1.93	0.005
1385158_at	similar to chromatin modifying protein 1B	LOC682926 /// LOC689364	851	1638	NM_001109533 /// XM_001060811 /// XM_001067633	18q12.1	1.93	0.002
1389210_at	lymphocyte cytosolic protein 1	Lcp1	1546	2976	NM_001012044	15q11	1.92	0.020
1381349_a_at	similar to ubiquitin protein ligase E3 component n-recognin 2	LOC363188	1141	2194	XM_001061775 /// XM_001061828 /// XM_001061882	9q12	1.92	0.002
1381100_at	Rho guanine nucleotide exchange factor (GEF) 12	Arhgef12	589	1132	BE108751	---	1.92	0.012
1381980_at	Transcribed locus	---	773	1485	BI278862	---	1.92	0.001
1382735_at	autophagy-related 12 (yeast)	Atg12	511	981	NM_001038495	18q11	1.92	0.005
1377192_a_at	caseinolytic peptidase X (E.coli)	Clpx	990	1902	NM_001007803	8q24	1.92	0.000
1368427_at	A kinase (PRKA) anchor protein 11	Akap11	544	1045	NM_012773 /// XM_573823	15q11	1.92	0.003
1373175_at	similar to RIKEN cDNA 1100001H23	RGD1308734	4260	8177	NM_001013927	4q43	1.92	0.004
1393267_at	PC4 and SFRS1 interacting protein 1	Psip1	2077	3984	NM_175765	5q31	1.92	0.018
1385070_at	similar to Opa-interacting protein 5 (predicted)	RGD1564263_predicted	922	1768	BE111420	---	1.92	0.001
1371439_at	similar to erythrocyte membrane protein band 4.1 (elliptocytosis 1, RH-linked) isoform 1 (predicted)	RGD1564762_predicted	669	1281	XM_001063302 /// XM_232771	5q36	1.92	0.010
1388896_at	ubiquitin specific peptidase 33	Usp33	2048	3924	XM_001068666 /// XM_001080019	2q45	1.92	0.000
1372509_at	Transcribed locus	---	418	800	AI172024	---	1.91	0.000
1374911_at	oxidative stress responsive gene	RGD1303142	2069	3954	NM_201560	3q42	1.91	0.003
1388847_at	positive cofactor 2, multiprotein complex, glutamine/Q-rich-associated protein (predicted)	Pcqap_predicted	524	1000	NM_001108325 /// XM_001065699 /// XM_341015	11q23	1.91	0.014
1373825_at	SCY1-like 2 (S. cerevisiae) (predicted)	Scyl2_predicted	934	1783	XM_001076749 /// XM_235050	7q13	1.91	0.002
1372004_at	heme binding protein 1 (predicted)	Hebp1_predicted	581	1108	AI102065	4	1.91	0.003
1369414_at	syntaxin binding protein 3	Stxbp3	428	816	NM_053637	2q34	1.91	0.002

Table S4. Trophoblast differentiation-associated genes

Affymetrix ID	Gene Title	Gene Symbol	Stem Ave	Dif Ave	GenBank	Chromosomal Location	dif/ stem	t-test
1390102_at	disrupted in renal carcinoma 2 homolog (human)	Dirc2	677	1288	NM_001012017	11q22	1.90	0.004
1373600_at	Similar to CG9996-PA	LOC300173	1089	2072	XM_001057023 /// XM_001057074 /// XM_217027	7q35	1.90	0.000
1399052_at	toll interacting protein (predicted)	Tollip_predicted	958	1821	NM_001109668 /// XM_001063419 /// XM_341961	1q41	1.90	0.000
1373160_at	similar to phosphatidic acid phosphatase type 2 domain containing 1A	LOC680466 /// LOC683534	1034	1967	NM_001109411 /// XM_001056392 /// XM_001062694	16q12.4	1.90	0.002
1388506_at	desmoplakin	Dsp	4061	7717	XM_001058415 /// XM_001058477 /// XM_001068439 /// XM_225259	17p12	1.90	0.001
1387777_at	integrin linked kinase	Ilk	1573	2987	NM_133409	1q33	1.90	0.009
1368650_at	Kruppel-like factor 10	Klf10	1542	2921	NM_031135	7q22	1.89	0.020
1371446_at	MAP kinase-activated protein kinase 2	Mapkapk2	740	1402	NM_178102	13q13	1.89	0.004
1376314_at	ubiquitin-conjugating enzyme E2Q (putative) 2 (predicted)	Ube2q2_predicted	3789	7167	XM_001072896	8q24	1.89	0.000
1392922_at	RAP2B, member of RAS oncogene family	Rap2b	777	1469	BE116619	---	1.89	0.001
1375929_at	max binding protein (predicted)	Mnt_predicted	599	1132	NM_001105807 /// XM_001080632 /// XM_220698	10q24	1.89	0.002
1373781_a_at	similar to suprabasal-specific protein suprabasin (predicted)	RGD1562305_predicted	12194	23036	NM_001044231 /// XM_001079264 /// XM_214902	1q21	1.89	0.003
1370305_at	Yip1 interacting factor homolog (S. cerevisiae)	Yif1	683	1289	NM_172017	1q43	1.89	0.000
1390786_at	rho/rac guanine nucleotide exchange factor (GEF) 2	Arhgef2	1730	3267	NM_001012079	2q34	1.89	0.004
1392051_at	similar to hypothetical protein FLJ14681 (predicted)	RGD1307907_predicted	487	919	XM_001081122 /// XM_342515	3q36	1.89	0.000
1379866_at	septin 6 (predicted)	Sept6_predicted	514	969	AW532098	---	1.89	0.005
1368433_at	SAC1 (suppressor of actin mutations 1, homolog)-like (S. cerevisiae)	Sacm1l	1134	2134	NM_053798	8q32	1.88	0.000
1374522_at	similar to melanoma inhibitory activity 3	LOC683007	2527	4756	XM_001064186	15	1.88	0.001
1372569_at	four and a half LIM domains 3 (predicted)	Fhl3_predicted	1000	1881	AI171676	---	1.88	0.002
1390208_at	HIV-1 tat interactive protein 2, homolog (human) (predicted)	Htatip2_predicted	753	1416	NM_001106263 /// XM_001080298 /// XM_214927	1q22	1.88	0.003
1376593_at	Transcribed locus	---	879	1653	BF283990	---	1.88	0.002
1379574_at	similar to Inner nuclear membrane protein Man1 (LEM domain containing protein 3)	LOC680066	761	1431	XM_001055542	7q22	1.88	0.003
1372221_at	Transcribed locus, strongly similar to XP_994234.1 PREDICTED: similar to Ubiquitin-conjugating enzyme E2 H (Ubiquitin-protein ligase H) (Ubiquitin carrier protein H) (UBCH2) (E2-20K) [Mus musculus]	---	1292	2429	BM389079	4	1.88	0.009
1389111_at	Transcribed locus	---	533	1002	BF396316	---	1.88	0.002
1399088_at	tousled-like kinase 2 (Arabidopsis) (predicted)	Tlk2_predicted	1467	2757	XM_001067052 /// XM_001081561 /// XM_221023	10q32.1	1.88	0.000

Table S4. Trophoblast differentiation-associated genes

Affymetrix ID	Gene Title	Gene Symbol	Stem Ave	Dif Ave	GenBank	Chromosomal Location	dif/ stem	t-test
1387926_at	sterol-C5-desaturase (fungal ERG3, delta-5-desaturase) homolog (S. cerevisae)	Sc5d	1138	2138	NM_053642	8q22	1.88	0.000
1380513_at	similar to Expressed sequence AW060207	RGD1309034	1057	1984	NM_001013987	14p22	1.88	0.000
1378001_at	Transcribed locus	---	2109	3953	AI548615	---	1.87	0.004
1376796_at	RAB14, member RAS oncogene family	Rab14	1621	3038	NM_053589	3p11	1.87	0.005
1383077_at	similar to RIKEN cDNA 1110063F24 (predicted)	RGD1311622_predicted	481	901	NM_001106636 /// XM_001061273 /// XM_001061326 /// XM_001061389 /// XM_216348	5q36	1.87	0.004
1372724_at	glutamate receptor, ionotropic, N-methyl D-asparate-associated protein 1 (glutamate binding)	Grina	1215	2276	NM_153308	7q34	1.87	0.009
1389322_at	programmed cell death protein 7 (predicted)	Pdcd7_predicted	676	1266	NM_001108768 /// XM_001076457 /// XM_343413	8q24	1.87	0.000
1373738_at	similar to M phase phosphoprotein 6	LOC686999	2951	5522	XM_001076650	11	1.87	0.002
1374629_at	mediator of RNA polymerase II transcription, subunit 8 homolog (yeast) (predicted)	Med8_predicted	616	1151	NM_001108673 /// XM_001072348 /// XM_342894	5q36	1.87	0.002
1372754_at	similar to DIP13 beta (predicted)	RGD1563028_predicted	488	911	NM_001108741 /// XM_001079632 /// XM_343190	7q13	1.87	0.003
1375006_at	CDNA clone IMAGE:7318427	---	2538	4741	BE121050	---	1.87	0.003
1367889_at	calcium/calmodulin-dependent protein kinase I	Camk1	448	836	NM_134468	4q42	1.87	0.000
1383884_at	Transcribed locus	---	668	1248	BF409598	---	1.87	0.001
1388999_at	transcription factor 12	Tcf12	936	1748	BF397907	8	1.87	0.000
1368247_at	heat shock 70kD protein 1A /// heat shock 70kD protein 1B (mapped)	Hspa1a /// Hspa1b	1095	2044	NM_031971 /// NM_212504	20p12	1.87	0.002
1370507_at	discs, large homolog-associated protein 4 (Drosophila)	Dlgap4	520	970	NM_173145	3q42	1.87	0.002
1374824_at	vacuolar protein sorting 53 (yeast) (predicted)	Vps53_predicted	630	1176	NM_001105813 /// XM_001080583 /// XM_213395	10q24	1.87	0.004
1395555_at	golgi SNAP receptor complex member 1	Gosr1	504	939	NM_053584	10q26	1.86	0.008
1367783_at	GABA(A) receptor-associated protein like 2	Gabarapl2	2156	4015	NM_022706	19q12	1.86	0.005
1370912_at	heat shock 70kD protein 1B (mapped)	Hspa1b	839	1562	NM_212504	20p12	1.86	0.000
1392772_at	cysteine-rich protein 3 (predicted)	Crip3_predicted	876	1628	BF285633	9	1.86	0.006
1373507_at	similar to acyl-Coenzyme A binding domain containing 5	LOC679565	1742	3238	XM_001053520	5q11	1.86	0.001
1394414_at	Yip1 domain family, member 4 /// hypothetical gene supported by BC088468; NM_001009712 (predicted)	RGD1565766_predicted /// Yipf4	2959	5493	NM_001009712 /// XM_580096	6q12 /// 6q14	1.86	0.015
1377254_a_at	Cohen syndrome homolog 1 (predicted)	Cohh1_predicted	450	835	XM_001058951 /// XM_243588	7q22	1.86	0.000
1391994_at	Transcribed locus	---	467	866	AI408264	---	1.86	0.005
1397824_at	similar to WAC (predicted)	RGD1562407_predicted	2222	4119	XM_001064372	17q12.1	1.85	0.002
1372612_at	dynein light chain LC8-type 2	Dynll2	3741	6932	NM_080697	10q26	1.85	0.000
1397363_at	Poliovirus receptor-related 3 (predicted)	Pvrl3_predicted	1659	3072	NM_001105883 /// XM_001059227 /// XM_213626	11q21	1.85	0.000

Table S4. Trophoblast differentiation-associated genes

Affymetrix ID	Gene Title	Gene Symbol	Stem Ave	Dif Ave	GenBank	Chromosomal Location	dif/ stem	t-test
1370807_at	transmembrane protein 49	Tmem49	1459	2701	NM_138839	10q26	1.85	0.000
1372500_at	tropomodulin 3	Tmod3	2781	5141	NM_001011997	8q24	1.85	0.000
1395574_at	ROD1 regulator of differentiation 1 (S. pombe)	Rod1	2534	4685	BE118358	5	1.85	0.000
1398294_at	actinin, alpha 1	Actn1	804	1486	NM_031005	6q24	1.85	0.037
1374296_at	leucine-rich repeat-containing 8	Lrrc8	1171	2161	BM387574	3	1.85	0.004
1370123_a_at	cortactin	Cttn	1563	2879	NM_021868	1q41-q42	1.84	0.001
1382040_at	glutamyl-prolyl-tRNA synthetase	Eprs	2218	4085	NM_001024238	13q26	1.84	0.021
1367855_at	scavenger receptor class B, member 1	Scarb1	444	817	NM_031541	12q15-q16	1.84	0.004
1385059_at	zinc finger protein 655	Zfp655	1201	2210	NM_001008362	12p11	1.84	0.004
1371386_at	similar to Protein C9orf10 (predicted) /// hypothetical protein LOC683420 /// similar to Protein CXorf17 homolog	LOC683420 /// LOC690938 /// RGD1306643_predicted	9838	18098	XM_001065874 /// XM_001076224 /// XM_237998	17p14 /// 1q12	1.84	0.001
1383023_at	ubiquitin-conjugating enzyme E2H (predicted)	Ube2h_predicted	614	1128	AI235658	4	1.84	0.002
1381118_at	similar to RIKEN cDNA 2700007P21	RGD1311463	461	847	NM_001014045	3q33	1.84	0.001
1372000_at	neuroepithelial cell transforming gene 1	Net1	2734	5017	NM_001039023	17q12.3	1.84	0.000
1388487_at	adducin 1 (alpha)	Add1	1363	2500	NM_016990	14q21	1.83	0.001
1389048_at	bone morphogenetic protein 1	Bmp1	821	1505	XM_001070483 /// XM_001070524 /// XM_001075543 /// XM_573814	15p11	1.83	0.001
1372650_at	similar to Dynamin-binding protein (Scaffold protein Tuba)	LOC309362	521	954	XM_001060754 /// XM_219860	1q51	1.83	0.001
1373096_at	ARP8 actin-related protein 8 homolog (S. cerevisiae) (predicted)	Actr8_predicted	1633	2992	NM_001108396 /// XM_001063449 /// XM_341393	16p16	1.83	0.000
1383076_at	similar to RIKEN cDNA 1200016B10 (predicted)	RGD1308695_predicted	1324	2427	NM_001105960 /// XM_001070454 /// XM_213894	13q21	1.83	0.001
1371028_at	trans-golgi network protein 2	Tgoln2	586	1073	NM_138840	4q33	1.83	0.017
1388983_at	similar to hypothetical protein (predicted)	RGD1305045_predicted	1703	3114	NM_001106496 /// XM_001074486 /// XM_215787	3q34	1.83	0.000
1371380_at	pyruvate dehydrogenase E1 alpha 1 /// pyruvate dehydrogenase E1 alpha 1 pseudogene	LOC685778 /// Pdha1	1065	1947	NM_001004072 /// XM_001060860 /// XM_001060918 /// XM_001067950 /// XM_001067997	16q12.5 /// Xq21	1.83	0.018
1373244_at	similar to RIKEN cDNA 2010008E23 gene	RGD1304758	868	1585	NM_001031651	10q32.1	1.83	0.000
1398879_at	transmembrane protein 66	Tmem66	1747	3192	NM_001004213	16q12.2	1.83	0.002
1367694_at	hydroxyacyl-Coenzyme A dehydrogenase/3-ketoacyl-Coenzyme A thiolase/enoyl-Coenzyme A hydratase (trifunctional protein), beta subunit	Hadhb	3697	6751	NM_133618	6q14	1.83	0.006
1367534_at	G protein-coupled receptor 21 (predicted)	Gpr21_predicted	447	816	NM_001107841 /// XM_001054010 /// XM_231251	3q11	1.83	0.023
1371627_at	Angiomotin-like 1 (predicted)	Amotl1_predicted	3352	6120	NM_001108126 /// XM_001074080 /// XM_235827	8q11	1.83	0.000

Table S4. Trophoblast differentiation-associated genes

Affymetrix ID	Gene Title	Gene Symbol	Stem Ave	Dif Ave	GenBank	Chromosomal Location	dif/ stem	t-test
1374920_at	Transcribed locus	---	1792	3269	AI228955	---	1.82	0.001
1388830_at	protein kinase N2	Pkn2	1775	3236	NM_001105755 /// XM_001076809 /// XM_215718	2q44	1.82	0.001
1375843_at	iduronate 2-sulfatase	Ids	2095	3816	XM_001054447 /// XM_579042	Xq37	1.82	0.000
1372088_at	PTPRF interacting protein, binding protein 1 (liprin beta 1) (predicted)	Ppfibp1_predicted	1355	2464	NM_001107896 /// XM_001074157 /// XM_232536	4q44	1.82	0.001
1387521_at	programmed cell death 4	Pdcd4	942	1714	NM_022265	1q55	1.82	0.006
1387641_at	RAB5A, member RAS oncogene family	Rab5a	1720	3126	NM_022692	9q11	1.82	0.008
1374947_at	breast cancer anti-estrogen resistance 3 (predicted)	Bcar3_predicted	503	914	NM_001107722 /// XM_001074184 /// XM_227657	2q42	1.82	0.002
1382065_at	Transcribed locus	---	1032	1872	BM388726	---	1.81	0.003
1382013_at	CAP, adenylate cyclase-associated protein, 2 (yeast)	Cap2	634	1148	AI071118	---	1.81	0.001
1376984_at	gene trap locus 6	Gtl6	539	976	AW915048	9	1.81	0.002
1372934_at	similar to 1700019E19Rik protein (predicted)	RGD1307392_predicted	849	1537	XM_001060869 /// XM_216750	6q31	1.81	0.002
1381564_at	glomulin, FKBP associated protein	Glmn	471	853	NM_001105993 /// XM_001057583 /// XM_213992	14p22	1.81	0.001
1399031_at	similar to CG31855-PA	LOC684106 /// LOC688813	714	1293	XM_001068407 /// XM_001068955	16p14	1.81	0.034
1373894_at	RAB31, member RAS oncogene family	Rab31	867	1568	NM_145094	9q37	1.81	0.014
1370163_at	ornithine decarboxylase 1	Odc1	10035	18146	NM_012615	6q16	1.81	0.000
1388444_at	UBX domain containing 2	Ubxd2	3259	5888	NM_001012025	13q12	1.81	0.005
1374047_at	similar to RIKEN cDNA 2400010D15	RGD1311805	484	874	NM_001009638	18p13	1.81	0.004
1368838_at	tropomyosin 4	Tpm4	5901	10655	NM_012678	16p14	1.81	0.005
1393110_at	Mpv17 transgene, kidney disease mutant-like (predicted)	Mpv17l_predicted	1150	2075	NM_001098240 /// XM_001075775 /// XM_340739	10q11	1.81	0.001
1388140_at	RAB13, member RAS oncogene family	Rab13	579	1044	NM_031092	2q34	1.80	0.024
1373346_at	similar to hypothetical protein CL25084 (predicted)	RGD1306508_predicted	1057	1905	NM_001106023 /// XM_001059292 /// XM_214121	14q22	1.80	0.049
1368819_at	integrin beta 1 (fibronectin receptor beta)	Itgb1	6048	10898	NM_017022	19q12	1.80	0.000
1368305_at	caspase 6	Casp6	2111	3804	NM_031775	2q43	1.80	0.005
1376197_at	transcription factor 7, T-cell specific (predicted)	Tcf7_predicted	543	978	XM_001073458 /// XM_343891	10q22	1.80	0.001
1391980_at	similar to ubiquitin specific protease 34	LOC360990	863	1554	XR_005456 /// XR_009611	14q22	1.80	0.008
1387454_at	niban protein	Niban	609	1096	NM_022242	13q21	1.80	0.003
1376274_at	BTB (POZ) domain containing 10	Btbd10	901	1621	NM_001014022	1q34	1.80	0.000
1382423_at	Transcribed locus	---	595	1069	AW920944	---	1.80	0.003
1372320_at	male-specific lethal-3 homolog 1 (Drosophila)	Msl31	600	1077	NM_001014111	Xq21	1.80	0.001
1372206_at	similar to chromosome 14 open reading frame 9	RGD1307475	646	1159	NM_001014233	15p14	1.79	0.003
1373561_at	chondroitin polymerizing factor	D1bwg1363e	984	1766	NM_001005906	9q33	1.79	0.002

Table S4. Trophoblast differentiation-associated genes

Affymetrix ID	Gene Title	Gene Symbol	Stem Ave	Dif Ave	GenBank	Chromosomal Location	dif/ stem	t-test
1388503_at	similar to CREBBP/EP300 inhibitory protein 1 (predicted)	RGD1562702_predicted	2434	4366	NM_001109205 /// XM_001077667 /// XM_575227	3q36	1.79	0.001
1376832_at	similar to FLJ20689 (predicted)	RGD1308907_predicted	834	1496	AA800763	---	1.79	0.004
1390063_at	microfibrillar-associated protein 3	Mfap3	674	1207	NM_001007609	10q22	1.79	0.000
1387782_at	dynein light chain LC8-type 2	Dynll2	1619	2899	NM_080697	10q26	1.79	0.000
1385741_at	similar to RIKEN cDNA 1700052N19	RGD1305235	530	948	NM_001017447	1p11	1.79	0.001
1389014_at	pre-B-cell colony enhancing factor 1	Pbef1	2638	4717	NM_177928	6q16	1.79	0.010
1374215_at	pleckstrin homology domain containing, family J member 1	Plekhj1	691	1235	NM_001108072 /// XM_001071331 /// XM_234909	7q11	1.79	0.010
1398934_at	mitogen-activated protein kinase kinase kinase 7 interacting protein 2	Map3k7ip2	2156	3853	BE107454	1	1.79	0.001
1398959_at	inositol polyphosphate-5-phosphatase E	Inpp5e	881	1574	XM_001078406 /// XM_342391	3p13	1.79	0.011
1398493_at	CDNA clone IMAGE:7318263	---	735	1313	BG377873	---	1.79	0.005
1367966_at	dipeptidylpeptidase 3 /// similar to Dipeptidyl-peptidase 3 (Dipeptidyl-peptidase III) (DPP III) (Dipeptidyl aminopeptidase III) (Dipeptidyl arylamidase III)	Dpp3 /// LOC678760	936	1671	NM_053748 /// XM_001053134	3q41	1.79	0.000
1387086_at	calcium modulating ligand	Camlg	819	1462	NM_053334	17p14	1.79	0.002
1388823_at	RAB5B, member RAS oncogene family (predicted)	Rab5b_predicted	1312	2343	NM_001079936 /// XM_001071247 /// XM_213824	7q11	1.79	0.000
1381065_at	hypothetical protein LOC499120	LOC499120	1186	2116	XM_001078762 /// XM_574414	1q21	1.78	0.013
1375955_at	zinc finger protein 313	Zfp313	1385	2469	NM_001001517	3q42	1.78	0.003
1393072_at	ubiquitin-conjugating enzyme E2Q (putative) 2 (predicted)	Ube2q2_predicted	3324	5923	XM_001072896	8q24	1.78	0.002
1387061_at	junction plakoglobin	Jup	2754	4905	NM_031047	10q32.1	1.78	0.001
1368385_a_at	growth factor receptor bound protein 2	Grb2	3053	5436	NM_030846	10q32.3	1.78	0.001
1378098_at	RGD1309748_predicted	similar to CG4768-PA (predicted)	1717	3057	AI406802	---	1.78	0.002
1390952_at	REST corepressor 1 (predicted)	Rcor1_predicted	2159	3844	NM_001108060 /// XM_001071532 /// XM_234546	6q32	1.78	0.013
1373529_at	mitochondrial carrier triple repeat 1	Mcart1	616	1097	BE117883	---	1.78	0.001
1375642_at	ubiquitin-conjugating enzyme E2B, RAD6 homolog (S. cerevisiae)	Ube2b	1216	2165	NM_031138	10q22	1.78	0.003
1389412_at	---	---	674	1200	AA800693	---	1.78	0.007
1371552_at	Transcribed locus	---	592	1054	BF400684	---	1.78	0.002
1372346_at	zinc finger protein 513	Zfp513	1038	1846	NM_001012110	6q14	1.78	0.000
1384148_at	similar to Ras-related protein Rab-20	LOC686866 /// LOC689377	635	1130	NM_001109535 /// XM_001070580 /// XM_001076108	16q12.5	1.78	0.001
1371614_at	autophagy-related 12 (yeast)	Atg12	1615	2870	NM_001038495	18q11	1.78	0.000
1370000_at	nucleobindin 2	Nucb2	675	1200	NM_021663	1q35	1.78	0.000
1369738_s_at	cAMP responsive element modulator	Crem	577	1026	NM_001110860 /// NM_013086 /// NM_017334	17q12.1	1.78	0.025
1376089_at	low density lipoprotein receptor	Ldlr	8931	15861	BI294974	8	1.78	0.001

Table S4. Trophoblast differentiation-associated genes

Affymetrix ID	Gene Title	Gene Symbol	Stem Ave	Dif Ave	GenBank	Chromosomal Location	dif/ stem	t-test
1374169_at	similar to chromosome 16 open reading frame 5	RGD1310686	622	1105	NM_001008360	10q12	1.78	0.046
1387857_at	syntaxin 7	Stx7	595	1056	NM_021869	1p12	1.78	0.001
1378257_at	three prime repair exonuclease 1	Trex1	653	1160	NM_001024989	8	1.78	0.014
1369452_a_at	phosphatidylinositol binding clathrin assembly protein	Picalm	920	1633	NM_053554	1q32	1.77	0.000
1376230_at	similar to Hypothetical protein MGC37938 (predicted)	RGD1566169_predicted	492	873	XM_001072960 /// XM_238429	5q36	1.77	0.003
1381972_at	cereblon	Crbn	1299	2304	NM_001015003	4q41	1.77	0.000
1388796_at	Golgi SNAP receptor complex member 1	Gosr1	534	945	NM_053584	10q26	1.77	0.005
1395802_at	similar to SEC24 related gene family, member C	LOC685144	689	1220	NM_001109456 /// XM_001056270 /// XM_001056391 /// XM_001056451 /// XM_001056512 /// XM_001056565 /// XM_001056635 /// XM_001061137 /// XM_001061263 /// XM_001061319 /// XM_001061382 /// XM_001061441 /// XM_001061501	15p16	1.77	0.022
1372012_at	24-dehydrocholesterol reductase	Dhcr24	503	890	NM_001080148 /// XM_001065263 /// XM_216452	5q34	1.77	0.004
1373418_at	glutamyl-prolyl-tRNA synthetase	Eprs	3871	6850	NM_001024238	13q26	1.77	0.013
1386929_at	hexokinase 1	Hk1	464	821	NM_012734	20q11	1.77	0.004
1368712_at	zinc finger protein 386 (Kruppel-like)	Znf386	776	1373	NM_019620	6q33	1.77	0.001
1375561_at	Transcribed locus	---	499	882	BG372234	---	1.77	0.017
1373476_at	hypothetical protein LOC500251	LOC500251	503	889	NM_001025047	4q34	1.77	0.001
1390524_at	ring finger protein 12	Rnf12	3383	5970	NM_001024892	Xq31	1.76	0.013
1390989_at	similar to Mospd2 protein (predicted)	RGD1563952_predicted	993	1753	XM_001071071 /// XM_343782	Xq21	1.76	0.002
1390157_at	Transcribed locus, strongly similar to XP_994234.1 PREDICTED: similar to Ubiquitin-conjugating enzyme E2 H (Ubiquitin-protein ligase H) (Ubiquitin carrier protein H) (UBCH2) (E2-20K) [Mus musculus]	---	895	1579	BI295124	4	1.76	0.001
1388474_at	Ubiquitin-conjugating enzyme E2I	Ube2i	1104	1946	NM_013050	10q12	1.76	0.002
1376813_at	membrane bound O-acyltransferase domain containing 5	Mboat5	898	1582	NM_001012189	4q42	1.76	0.002
1374687_at	extra spindle poles like 1 (S. cerevisiae) (predicted)	Espl1_predicted	3420	6024	BF283386	7	1.76	0.000
1374514_at	Transcribed locus	---	1525	2683	BG671893	---	1.76	0.000
1371810_at	Coatomer protein complex, subunit gamma	Copg	534	939	NM_001031822	4q34	1.76	0.009
1383059_a_at	G kinase anchoring protein 1	Gkap1	908	1598	NM_001012160	17p14	1.76	0.001
1377092_at	Transcribed locus	---	992	1743	BF389682	---	1.76	0.008
1372717_at	Transcribed locus	---	639	1123	BI296012	---	1.76	0.006
1374007_at	similar to step II splicing factor SLU7; DNA segment, Chr 11, ERATO Doi 730, expressed; DNA segment, Chr 3, Brigham & Womens Genetics 0878 expressed	LOC303057	1324	2326	XM_001067382 /// XM_001067430 /// XM_220315	10q21	1.76	0.005

Table S4. Trophoblast differentiation-associated genes

Affymetrix ID	Gene Title	Gene Symbol	Stem Ave	Dif Ave	GenBank	Chromosomal Location	dif/ stem	t-test
1367856_at	glucose-6-phosphate dehydrogenase X-linked	G6pdx	2116	3717	NM_017006	Xq37	1.76	0.004
1377166_at	Amyotrophic lateral sclerosis 2 (juvenile) homolog (human)	Als2	515	905	NM_001013413 /// XM_001061827 /// XM_001064519 /// XM_001064573 /// XM_343574	9q31	1.76	0.001
1373282_at	similar to mitochondrial carrier protein MGC4399	LOC691431	703	1234	XM_001078245	5q36	1.76	0.005
1391827_at	filamin, beta (predicted)	Flnb_predicted	2213	3885	NM_001107288 /// XM_001071051 /// XM_224561	15p14	1.76	0.018
1389205_at	Transcribed locus	---	640	1123	AI012582	---	1.75	0.017
1372262_at	RGD1563087 (predicted)	RGD1563087_predicted	1075	1886	BI285626	---	1.75	0.003
1374584_at	similar to Serine/threonine protein kinase 24 (predicted)	RGD1561742_predicted	1073	1881	XM_001078875 /// XM_341377	15q25	1.75	0.000
1367512_at	chromatin modifying protein 5	Chmp5	5834	10230	NM_001025410	5q22	1.75	0.011
1373918_at	retinol dehydrogenase 11	Rdh11	617	1081	NM_001012193	6q24	1.75	0.001
1386880_at	acetyl-Coenzyme A acyltransferase 2 (mitochondrial 3-oxoacyl-Coenzyme A thiolase)	Acaa2	1360	2383	NM_130433	18q12.3	1.75	0.001
1372825_at	Formin binding protein 1	Fnbp1	844	1478	NM_138914	3p12	1.75	0.009
1372400_at	similar to cullin 4A (predicted)	RGD1563853_predicted	634	1110	XM_001066808 /// XM_341464	16q12.5	1.75	0.011
1370121_at	adducin 1 (alpha)	Add1	489	855	NM_016990	14q21	1.75	0.007
1375412_at	arylsulfatase B	Arsb	963	1684	AI101331	2	1.75	0.001
1370826_at	nucleosome assembly protein 1-like 1	Nap1l1	7107	12426	NM_053561	7q21	1.75	0.000
1390152_at	transmembrane emp24 protein transport domain containing 7 /// similar to transmembrane emp24 protein transport domain containing 7	LOC679060 /// Tmed7	820	1431	NM_001105758 /// XM_001054517 /// XM_001063185	18q11	1.75	0.043
1377739_at	guanine nucleotide binding protein (G protein), gamma 12	Gng12	991	1731	XM_001067408 /// XM_578287	4q31	1.75	0.012
1370876_at	similar to proline arginine rich coiled coil 1	LOC681287	942	1644	NM_001109438 /// XM_001057635 /// XM_001057691 /// XM_001057759 /// XM_001057822 /// XM_001057873 /// XM_001058588 /// XM_001058646 /// XM_001058709 /// XM_001058764 /// XM_001058826	5q36	1.75	0.001
1374028_at	similar to CDNA sequence BC024479	LOC500974	751	1312	XR_005433 /// XR_009636	8q21	1.75	0.002
1388365_at	ATPase, H+ transporting, V0 subunit D isoform 1	Atp6v0d1	2527	4410	NM_001011927	19q11	1.75	0.000
1389685_at	zinc finger protein 655	Zfp655	3582	6248	NM_001008362	12p11	1.74	0.000
1374428_at	kinesin family member 3B (predicted)	Kif3b_predicted	548	957	NM_001106529 /// XM_001061322 /// XM_001061386 /// XM_001061444 /// XM_001061504 /// XM_215883	3q41	1.74	0.002
1399122_at	Transcribed locus	---	544	947	BI278603	---	1.74	0.001

Table S4. Trophoblast differentiation-associated genes

Affymetrix ID	Gene Title	Gene Symbol	Stem Ave	Dif Ave	GenBank	Chromosomal Location	dif/ stem	t-test
1374914_at	peroxisome proliferator activated receptor delta	Ppard	704	1226	AI230294	20	1.74	0.002
1388520_at	sperm associated antigen 9 (predicted)	Spag9_predicted	2743	4775	NM_001108290 /// XM_001081278 /// XM_340879	10q26	1.74	0.001
1394871_at	solute carrier family 31 (copper transporters), member 1	Slc31a1	553	960	BF394431	5	1.74	0.004
1368824_at	caldesmon 1	Cald1	1558	2705	NM_013146	4q22	1.74	0.029
1385595_at	similar to antigenic determinant of rec-A protein	LOC683353 /// LOC689197	582	1008	NM_001109529 /// XM_001064234 /// XM_001066201	17q12.3	1.73	0.016
1386995_at	B-cell translocation gene 2, anti-proliferative	Btg2	9996	17313	NM_017259	13	1.73	0.047
1367848_at	dynactin 1	Dctn1	859	1488	NM_024130	4q34	1.73	0.006
1373142_at	Growth hormone inducible transmembrane protein	Ghitm	2283	3952	NM_001005908	16p14	1.73	0.001
1387855_at	guanosine diphosphate dissociation inhibitor 1	Gdi1	1343	2325	NM_017088	Xq37.1-q37.2	1.73	0.009
1370244_at	cathepsin L	Ctsl	14237	24644	NM_013156	17p14	1.73	0.010
1367545_at	G protein-coupled receptor 21 (predicted)	Gpr21_predicted	596	1030	NM_001107841 /// XM_001054010 /// XM_231251	3q11	1.73	0.002
1373279_at	similar to beta-catenin-interacting protein ICAT	LOC503000	1102	1905	XM_001075213 /// XM_001078135	5q36	1.73	0.000
1375964_at	phosphoserine phosphatase	Psph	1055	1824	NM_001009679	12q13	1.73	0.001
1372862_at	RAB22A, member RAS oncogene family (predicted)	Rab22a_predicted	1112	1923	NM_001108966 /// XM_001054313 /// XM_001054385 /// XM_345479	3q42	1.73	0.002
1393055_at	protein kinase N2	Pkn2	789	1362	NM_001105755 /// XM_001076809 /// XM_215718	2q44	1.73	0.000
1382846_at	Transcribed locus	---	1220	2106	AI501459	---	1.73	0.007
1395237_at	eukaryotic translation initiation factor 5B /// similar to Eukaryotic translation initiation factor 5B (eIF-5B) (Translation initiation factor IF-2)	Eif5b /// LOC689581	1120	1933	NM_001110141 /// XM_001055509 /// XM_001057591 /// XM_001057637 /// XM_001071498 /// XM_001071547 /// XM_001075051 /// XM_001075073 /// XM_218162	19q12 /// 9q21	1.73	0.005
1379689_at	Transcribed locus	---	1015	1750	AI011930	---	1.72	0.002
1372201_at	zinc finger protein 403	Zfp403	1572	2709	NM_001004273 /// XM_001081060 /// XM_001081065	10q26	1.72	0.004
1377214_a_at	hypothetical protein LOC292764	RGD1303117	907	1560	NM_001004219	1q21	1.72	0.048
1367532_at	DAZ associated protein 2	Dazap2	2567	4414	NM_001013107	7q36	1.72	0.011
1387654_at	myosin IC	Myo1c	799	1373	NM_023092 /// XM_001080717	10q24	1.72	0.001
1371555_at	sorting nexin 12 (predicted)	Snx12_predicted	609	1047	NM_001108817 /// XM_001069787 /// XM_343799	Xq31	1.72	0.007
1384319_at	Tousled-like kinase 2 (Arabidopsis) (predicted)	Tlk2_predicted	1426	2449	XM_001067052 /// XM_001081561 /// XM_221023	10q32.1	1.72	0.020

Table S4. Trophoblast differentiation-associated genes

Affymetrix ID	Gene Title	Gene Symbol	Stem Ave	Dif Ave	GenBank	Chromosomal Location	dif/ stem	t-test
1372624_at	transmembrane protein 16F (predicted)	Tmem16f_predicted	737	1265	NM_001108108 /// XM_001057694 /// XM_001057761 /// XM_235640	7q35	1.72	0.000
1383894_at	Transcribed locus	---	1290	2215	BI289486	---	1.72	0.028
1371594_at	fission 1 (mitochondrial outer membrane) homolog (yeast)	Fis1	1274	2185	NM_001105919 /// XM_001076962 /// XM_213746	12q12	1.71	0.011
1375185_at	importin 7 (predicted)	Ipo7_predicted	4469	7659	NM_001107545 /// XM_001077641 /// XM_219265	1q33	1.71	0.000
1372038_at	MAP kinase-interacting serine/threonine kinase 2	Mknk2	524	897	NM_001011985	7q11	1.71	0.002
1388937_at	ring finger protein (C3HC4 type) 19 (predicted)	Rnf19_predicted	702	1202	XM_001059414 /// XM_343228	7q22	1.71	0.003
1376660_at	Transcribed locus	---	526	900	BG672127	---	1.71	0.012
1372758_at	Transcribed locus	---	951	1627	BI282978	---	1.71	0.004
1367624_at	activating transcription factor 4	Atf4	9715	16615	NM_024403	7q34	1.71	0.001
1388755_at	SEC23A (S. cerevisiae) (predicted)	Sec23a_predicted	966	1652	AI179139	6	1.71	0.002
1373491_at	similar to Glucosylceramidase precursor (Beta-glucocerebrosidase) (Acid beta-glucosidase) (D-glucosyl-N-acylsphingosine glucohydrolase)	LOC684536	675	1153	XM_001071921 /// XM_001071959	2	1.71	0.001
1392462_at	v-crk sarcoma virus CT10 oncogene homolog (avian)	Crk	3605	6159	AA817891	10	1.71	0.001
1377886_at	Transcribed locus, strongly similar to XP_926592.1 PREDICTED: similar to Tripartite motif protein 6 isoform 3 [Mus musculus]	---	1684	2876	BI274321	1	1.71	0.000
1370265_at	arrestin, beta 2	Arrb2	564	964	NM_012911	10q24	1.71	0.001
1398999_at	similar to es 64	LOC363675	924	1574	NM_001014229	10q31	1.70	0.008
1374548_at	AF4/FMR2 family, member 4 (predicted)	Aff4_predicted	2322	3956	NM_001107001 /// XM_001073824 /// XM_220420	10q22	1.70	0.021
1393843_at	feminization 1 homolog b (C. elegans) (predicted)	Fem1b_predicted	1723	2934	NM_001108157 /// XM_001074110 /// XM_236321	8q24	1.70	0.002
1388445_at	annexin A11	Anxa11	1796	3057	NM_001011918	16p16	1.70	0.000
1369994_at	calcitonin gene-related peptide-receptor component protein	Crcp	695	1182	NM_053670	12q13	1.70	0.019
1398905_at	ATPase, H transporting, lysosomal V1 subunit G1 (predicted)	Atp6v1g1_predicted	877	1490	NM_001106660 /// XM_001064435 /// XM_216411	5q24	1.70	0.008
1374212_at	Transcribed locus	---	1525	2592	AI012753	---	1.70	0.002
1388771_at	CGG triplet repeat binding protein 1 (predicted)	Cggbp1_predicted	984	1671	NM_001105900 /// XM_001063614 /// XM_213679	11p12	1.70	0.000
1383206_at	component of oligomeric golgi complex 3	Cog3	511	868	NM_001012157	15q11	1.70	0.030
1382721_at	zinc finger protein 403	Zfp403	2933	4978	NM_001004273 /// XM_001081060 /// XM_001081065	10q26	1.70	0.003
1372120_at	ubiquitin-activating enzyme E1-domain containing 1	Ube1dc1	1039	1762	NM_001009669	8q32	1.70	0.000
1399114_at	general transcription factor II E, polypeptide 2 (beta subunit) (predicted)	Gtf2e2_predicted	1602	2718	NM_001107318 /// XM_001067878 /// XM_224929	16q12.3	1.70	0.001

Table S4. Trophoblast differentiation-associated genes

Affymetrix ID	Gene Title	Gene Symbol	Stem Ave	Dif Ave	GenBank	Chromosomal Location	dif/ stem	t-test
1373862_at	transmembrane emp24 protein transport domain containing 7	Tmed7	6674	11320	NM_001105758 /// XM_001063185	18q11	1.70	0.002
1390868_at	similar to RIKEN cDNA 1200016B10 (predicted)	RGD1308695_predicted	842	1428	NM_001105960 /// XM_001070454 /// XM_213894	13q21	1.70	0.002
1371337_at	cytochrome c oxidase subunit VIIa polypeptide 2 like (predicted)	Cox7a2l_predicted	6498	11020	NM_001106704 /// XM_001056904 /// XM_216635	6q12	1.70	0.001
1368386_at	growth factor receptor bound protein 2	Grb2	1995	3381	NM_030846	10q32.3	1.69	0.014
1383060_at	G kinase anchoring protein 1	Gkap1	643	1089	NM_001012160	17p14	1.69	0.002
1391040_at	similar to Retinoblastoma-binding protein 8 (RBBP-8) (CtBP interacting protein) (CtIP) (Retinoblastoma-interacting protein and myosin-like) (RIM) (predicted)	RGD1308872_predicted	650	1100	XM_001072535 /// XM_214620	18p13	1.69	0.012
1372177_at	molybdenum cofactor synthesis 2	Mocs2	882	1492	NM_001007633 /// XM_001067999 /// XM_001068053	2q14	1.69	0.020
1388512_at	phosphodiesterase 6D, cGMP-specific, rod, delta (predicted)	Pde6d_predicted	640	1082	NM_001108806 /// XM_001066038 /// XM_343613	9q35	1.69	0.010
1379909_at	G kinase anchoring protein 1	Gkap1	2918	4931	NM_001012160	17p14	1.69	0.003
1376153_at	Transcribed locus	---	1355	2288	BE102621	---	1.69	0.003
1396128_at	similar to RIKEN cDNA 1700034P14 (predicted)	RGD1305492_predicted	1862	3141	NM_001106410 /// XM_001066754 /// XM_215485	2q14	1.69	0.003
1377103_at	midnolin (predicted)	Midn_predicted	1681	2836	XM_001076784 /// XM_234902	7q11	1.69	0.001
1371653_at	Tropomyosin 4	Tpm4	12657	21341	NM_012678	16p14	1.69	0.002
1393569_at	RNA binding motif protein 18 (predicted)	Rbm18_predicted	1231	2076	NM_001107838 /// XM_001080367 /// XM_231176	3p11	1.69	0.001
1368030_at	guanine nucleotide binding protein, alpha inhibiting 3 /// guanine nucleotide binding protein, alpha inhibiting 1	Gnai1 /// Gnai3	2223	3744	NM_013106 /// NM_013145	2q34 /// 4q11	1.68	0.011
1389730_at	Transcribed locus	---	1141	1921	AI176502	---	1.68	0.020
1367854_at	ATP citrate lyase	Acly	5221	8787	NM_001111095 /// NM_016987	10q32.1	1.68	0.000
1386229_at	similar to CBF1 interacting corepressor	RGD1309199	755	1270	NM_001007799	3q22	1.68	0.001
1372414_at	microtubule-associated proteins 1A/1B light chain 3	Map1lc3b	1006	1692	BI281979	---	1.68	0.003
1373301_at	protein phosphatase 1, regulatory (inhibitor) subunit 13B (predicted)	Ppp1r13b_predicted	2118	3563	NM_001108062 /// XM_001072164 /// XM_234555	6q32	1.68	0.012
1373206_at	fibronectin type III domain containing 3B (predicted)	Fndc3b_predicted	2706	4551	XM_001057133 /// XM_226988	2q24	1.68	0.001
1385889_at	Similar to hypothetical protein MGC52110 (predicted)	RGD1565095_predicted	5680	9547	XM_001057297 /// XM_578786	9q21	1.68	0.015
1372196_at	nuclear receptor co-repressor 2 (predicted)	Ncor2_predicted	647	1088	NM_001108334 /// XM_001074041 /// XM_341072	12q15	1.68	0.004
1371983_at	Josephin domain containing 1	Josd1	571	959	NM_001025009	7q34	1.68	0.015
1374048_at	neurturin	Nrtn	596	1000	NM_053399	9q11	1.68	0.000
1368834_at	calcium/calmodulin-dependent protein kinase II, delta	Camk2d	629	1055	NM_012519	2q42-q43	1.68	0.017
1372791_at	Protein kinase, lysine deficient 1	Prkwnk1	2505	4201	NM_053794	4q42	1.68	0.005
1387152_at	nuclear receptor binding factor 2	Nrbf2	607	1017	NM_022186	20p11	1.68	0.000

Table S4. Trophoblast differentiation-associated genes

Affymetrix ID	Gene Title	Gene Symbol	Stem Ave	Dif Ave	GenBank	Chromosomal Location	dif/ stem	t-test
1372283_at	trafficking protein particle complex 6B (predicted)	Trappc6b_predicted	1783	2986	NM_001106733 /// XM_001079724 /// XM_216708	6q23	1.67	0.009
1367884_at	RAB14, member RAS oncogene family	Rab14	4853	8125	NM_053589	3p11	1.67	0.016
1378500_at	AT rich interactive domain 3A (Bright like) (predicted)	Arid3a_predicted	922	1544	NM_001108066 /// XM_001076973 /// XM_234884	7q11	1.67	0.001
1387016_a_at	stromal cell derived factor receptor 1	Sdfr1	4995	8360	NM_019380	8q24	1.67	0.001
1399151_at	similar to hypothetical protein FLJ11526 (predicted)	RGD1309585_predicted	2295	3840	XM_001075034 /// XM_340803	10q22	1.67	0.002
1394993_at	Transcribed locus	---	1916	3203	AW531339	---	1.67	0.044
1369879_a_at	testis enhanced gene transcript	Tegt	4890	8177	NM_019381 /// XM_001063712 /// XM_576343	7q36	1.67	0.008
1383167_at	pregnancy-specific beta 1-glycoprotein	RGD:727932	545	912	BM392101	1	1.67	0.004
1376627_at	Transcribed locus	---	1188	1984	BF550404	---	1.67	0.032
1387800_at	Fas death domain-associated protein	Daxx	1011	1688	NM_080891	20p12	1.67	0.000
1393310_at	itchy homolog E3 ubiquitin protein ligase	Itch	552	921	BG672309	3	1.67	0.001
1398986_at	serine incorporator 3	Serinc3	4558	7603	NM_001008312	3q42	1.67	0.010
1372548_at	Transcribed locus, strongly similar to XP_999783.1 PREDICTED: similar to cryptochrome 2 (photolyase-like) isoform 5 [Mus musculus]	---	557	930	AI412018	3	1.67	0.017
1391497_at	TBC1D12: TBC1 domain family, member 12 (predicted)	Tbc1d12_predicted	651	1086	AA997515	---	1.67	0.013
1383265_at	TBC1 domain family, member 23 (predicted)	Tbc1d23_predicted	938	1563	NM_001107099 /// XM_001060935 /// XM_221532	11q12	1.67	0.002
1368984_at	septin 2	Sept2	2334	3887	NM_057148	9q36	1.67	0.035
1373867_at	similar to Retinoblastoma-binding protein 2 (RBBP-2)	LOC312678	2155	3588	XR_005442 /// XR_009628	4q42	1.66	0.000
1377807_a_at	Similar to IQ motif and WD repeats 1 (predicted)	RGD1561961_predicted	983	1636	XM_001071288 /// XM_213926	13q23	1.66	0.027
1367547_at	similar to zinc finger protein 198	LOC305913	1856	3085	XM_001060431 /// XM_224241	15p12	1.66	0.012
1374462_at	kinesin-associated protein 3 (predicted)	Kifap3_predicted	583	970	NM_001105964 /// XM_001070739 /// XM_213920	13q22	1.66	0.040
1372789_at	zinc finger protein 637	Zfp637	694	1153	XM_001056246 /// XM_342745	4q42	1.66	0.000
1389531_at	zinc finger protein 330 (predicted)	Zfp330_predicted	3956	6568	NM_001108443 /// XM_001073428 /// XM_341666	19q11	1.66	0.001
1383431_at	similar to KIAA1841 protein (predicted)	RGD1305110_predicted	668	1108	BF404209	---	1.66	0.000
1389383_at	SEC24 related gene family, member A (S. cerevisiae) (predicted)	Sec24a_predicted	898	1488	BI289855	---	1.66	0.000
1383697_at	Transcribed locus	---	661	1095	AW530905	---	1.66	0.000
1389233_at	RalBP1 associated Eps domain containing protein (predicted)	Reps1_predicted	1566	2595	NM_001106264 /// XM_001060578 /// XM_001060639 /// XM_001060697 /// XM_214954	1p12	1.66	0.007
1376262_at	UDP-glucuronate decarboxylase 1	Uxs1	1653	2738	NM_139336	9q32	1.66	0.001
1371465_at	cortactin	Cttn	2967	4914	NM_021868	1q41-q42	1.66	0.005

Table S4. Trophoblast differentiation-associated genes

Affymetrix ID	Gene Title	Gene Symbol	Stem Ave	Dif Ave	GenBank	Chromosomal Location	dif/ stem	t-test
1371723_at	Ras-related GTP binding C (predicted)	Rragc_predicted	1890	3127	NM_001048184 /// XM_001055506 /// XM_216515	5q36	1.65	0.003
1383455_at	glutamyl-prolyl-tRNA synthetase	Eprs	4207	6957	NM_001024238	13q26	1.65	0.000
1372395_at	similar to KIAA0597 protein (predicted)	RGD1565757_predicted	1326	2193	XM_001065952 /// XM_215517	2q22	1.65	0.015
1380808_at	toll interacting protein (predicted)	Tollip_predicted	753	1244	NM_001109668 /// XM_001063419 /// XM_341961	1q41	1.65	0.001
1391632_at	inositol polyphosphate-5-phosphatase E	Inpp5e	777	1283	XM_001078406 /// XM_342391	3p13	1.65	0.032
1394654_at	zinc finger protein 451	Zfp451	572	944	NM_001033705	9q21	1.65	0.030
1390852_x_at	Similar to IQ motif and WD repeats 1 (predicted)	RGD1561961_predicted	1007	1661	XM_001071288 /// XM_213926	13q23	1.65	0.004
1383327_at	programmed cell death 4	Pdcd4	820	1352	AI576297	1	1.65	0.035
1382365_at	Similar to nemo like kinase (predicted)	RGD1561602_predicted	695	1146	XM_001080888 /// XM_573152	10q25	1.65	0.039
1370025_at	phosphatidylinositol-4-phosphate 5-kinase, type II, gamma	Pip5k2c	1671	2751	NM_080480	7q22	1.65	0.001
1374608_at	similar to TatD DNase domain containing 2	LOC500295	1105	1819	NM_001109252 /// XM_001078895 /// XM_575644	4q42	1.65	0.027
1376043_at	similar to PHD zinc finger containing protein JUNE1	MGC94192	2104	3464	NM_001004272	10q24	1.65	0.000
1379412_at	Transcribed locus	---	567	933	BF559875	---	1.65	0.028
1385596_at	TBC1 domain family, member 15	Tbc1d15	1653	2716	XM_001078627 /// XM_345825	7q22	1.64	0.000
1390199_at	Transcribed locus	---	874	1434	BM389647	---	1.64	0.003
1399085_at	zinc metalloproteinase, STE24 homolog (S. cerevisiae) (predicted)	Zmpste24_predicted	628	1028	BM384770	5	1.64	0.009
1388408_at	similar to RIKEN cDNA 1110020C13	RGD1307129	3435	5624	NM_001008379	7q36	1.64	0.004
1384387_at	Transcribed locus	---	900	1473	BE110061	---	1.64	0.016
1373791_at	similar to RIKEN cDNA 2310011J03	RGD1359127	1221	1999	NM_001007657	7q11	1.64	0.008
1390115_at	SEC63-like (S. cerevisiae) (predicted)	Sec63_predicted	849	1390	NM_001107637 /// XM_001063100 /// XM_228305	20q13	1.64	0.026
1376080_at	REST corepressor 1 (predicted)	Rcor1_predicted	1431	2340	NM_001108060 /// XM_001071532 /// XM_234546	6q32	1.64	0.002
1391837_at	similar to chr2 synaptotagmin (predicted)	RGD1565705_predicted	954	1559	XR_007908	6q33	1.64	0.001
1382045_at	TBC1 domain family, member 15	Tbc1d15	2441	3988	XM_001078627 /// XM_345825	7q22	1.63	0.001
1382756_at	karyopherin (importin) alpha 1	Kpna1	617	1007	NM_198726	11q22	1.63	0.000
1375666_at	cyclin D binding myb-like transcription factor 1	Dmtf1	1108	1809	NM_053693	4	1.63	0.002
1370014_at	syntaxin 4A (placental)	Stx4a	1369	2235	NM_031125	1q36	1.63	0.004
1383328_x_at	programmed cell death 4	Pdcd4	1122	1831	NM_022265	1q55	1.63	0.034
1380110_at	Janus kinase 2	Jak2	760	1240	NM_031514	1q51-q53	1.63	0.003
1373284_at	salvador homolog 1 (Drosophila) (predicted)	Sav1_predicted	1096	1788	NM_001097581 /// XM_001080429 /// XM_216725	6q24	1.63	0.000
1367796_at	mannoside acetylglucosaminyltransferase 1	Mgat1	523	853	NM_030861	10q21	1.63	0.001

Table S4. Trophoblast differentiation-associated genes

Affymetrix ID	Gene Title	Gene Symbol	Stem Ave	Dif Ave	GenBank	Chromosomal Location	dif/ stem	t-test
1373817_at	inhibitor of growth family, member 4	Ing4	720	1173	NM_001079887 /// XM_001064956 /// XM_216265	4q42	1.63	0.010
1378637_at	nuclear transcription factor, X-box binding 1	Nfx1	3568	5813	XM_001059354 /// XM_001059410 /// XM_001059469 /// XM_001064398 /// XM_001064460	5q22	1.63	0.024
1399123_at	DEAD/H (Asp-Glu-Ala-Asp/His) box polypeptide 26	Ddx26	1498	2440	BI302415	15	1.63	0.001
1379699_at	similar to FLJ20689 (predicted)	RGD1308907_predicted	758	1231	AI549388	---	1.62	0.026
1390352_at	polycomb group ring finger 1	Pcgf1	605	983	NM_001007000	4q34	1.62	0.006
1379513_at	transmembrane protein 30B (predicted)	Tmem30b_predicted	781	1268	NM_001080380 /// XM_001080722 /// XM_234295	6q24	1.62	0.010
1379282_at	leucine rich repeat (in FLII) interacting protein 2	Lrrfip2	524	850	NM_001024761	8q32	1.62	0.050
1379259_at	Transcribed locus	---	1022	1659	AW534671	---	1.62	0.006
1369631_at	myosin IC /// similar to unconventional myosin Myr2 I heavy chain	LOC686250 /// Myo1c	1880	3051	NM_023092 /// XM_001073096 /// XM_001080717	10q24	1.62	0.003
1373416_at	fibronectin type III domain containing 3B (predicted)	Fndc3b_predicted	2831	4590	XM_001057133 /// XM_226988	2q24	1.62	0.001
1371595_at	Transcribed locus, weakly similar to XP_984565.1 PREDICTED: hypothetical protein LOC66961 [Mus musculus]	---	919	1489	BM384301	---	1.62	0.009
1379450_at	---	---	909	1472	BM383195	2	1.62	0.001
1374412_at	F-box and WD-40 domain protein 11 (predicted)	Fbxw11_predicted	1811	2931	NM_001106993 /// XM_001063835 /// XM_220281	10q12	1.62	0.000
1382537_at	Ras-related GTP binding C (predicted)	Rragc_predicted	1486	2406	NM_001048184 /// XM_001055506 /// XM_216515	5q36	1.62	0.001
1372341_at	similar to solute carrier family 25, member 36	LOC501039	559	905	XM_001065705 /// XM_576451	8q31	1.62	0.045
1382417_at	transformed mouse 3T3 cell double minute 4	Mdm4	836	1350	NM_001012026	13q13	1.61	0.002
1384901_at	zinc finger protein 451	Zfp451	586	946	BF523017	---	1.61	0.003
1372904_at	MOB1, Mps One Binder kinase activator-like 2B (yeast) (predicted)	Mobkl2b_predicted	674	1088	NM_001108734 /// XM_001071737 /// XM_343161	7q11	1.61	0.004
1390448_at	similar to 1110065L07Rik protein (predicted)	RGD1308317_predicted	592	955	NM_001107327 /// XM_001076411 /// XM_225044	16q12.5	1.61	0.009
1390131_at	serine racemase	Srr	781	1259	NM_198757	10q24	1.61	0.000
1373553_at	topoisomerase (DNA) III beta (predicted)	Top3b_predicted	649	1045	NM_001105861 /// XM_001067804 /// XM_213564	11q23	1.61	0.023
1369421_at	topoisomerase (DNA) I	Top1	1456	2347	NM_022615	3q42	1.61	0.023
1399022_at	CDC-like kinase 1	Clk1	3024	4872	NM_001106913 /// XM_001063325 /// XM_217405	9q31	1.61	0.010
1371893_at	procollagen, type IV, alpha 3 (Goodpasture antigen) binding protein (predicted)	Col4a3bp_predicted	1302	2096	NM_001108935 /// XM_001068766 /// XM_345143	2q12	1.61	0.005

Table S4. Trophoblast differentiation-associated genes

Affymetrix ID	Gene Title	Gene Symbol	Stem Ave	Dif Ave	GenBank	Chromosomal Location	dif/ stem	t-test
1370501_at	ubiquitin-conjugating enzyme E2G 1 (UBC7 homolog, C. elegans)	Ube2g1	3293	5303	NM_022690	10q24	1.61	0.011
1390142_at	microrchidia 3 (predicted)	Morc3_predicted	1194	1922	NM_001107109 /// XM_001054868 /// XM_221635	11q11	1.61	0.000
1381043_at	spermatogenesis associated, serine-rich 1	Spats1	630	1014	AI639256	9	1.61	0.044
1377262_at	similar to KIAA2010 protein (predicted)	RGD1309450_predicted	1814	2916	NM_001108367 /// XM_001053322 /// XM_001058097	14q22	1.61	0.040
1378405_at	zinc finger protein 143	Zfp143	640	1029	NM_001012169	1q33	1.61	0.000
1375423_at	hypothetical protein LOC689959	LOC689959	729	1172	NM_001109559 /// XM_001065356 /// XM_001068156	8q23	1.61	0.039
1380148_at	Wolf-Hirschhorn syndrome candidate 1 (predicted)	Whsc1_predicted	1996	3207	BE118688	14	1.61	0.013
1367865_at	limkain b1	Lkap	527	847	NM_133421 /// XM_001077357	10q11	1.61	0.041
1367940_at	chemokine orphan receptor 1	Cmkor1	1704	2736	NM_053352	9q35	1.61	0.010
1370070_at	synaptojanin 1	Synj1	740	1189	XM_001073290 /// XM_573256	11q11	1.61	0.011
1389729_at	similar to cDNA sequence BC003324	MGC94142	583	936	NM_001004205	12q15	1.60	0.020
1367840_at	HGF-regulated tyrosine kinase substrate	Hgs	1361	2183	NM_019387	10q32.3	1.60	0.001
1389717_at	similar to KIAA0157 gene product is novel. (predicted)	RGD1308918_predicted	635	1019	NM_001106307 /// XM_001054804 /// XM_219441	1q41	1.60	0.007
1397617_at	similar to Retinoblastoma-binding protein 2 (RBBP-2)	LOC312678	734	1177	XR_005442 /// XR_009628	4q42	1.60	0.001
1384439_at	peptidylprolyl isomerase D	RGD:1303174	1476	2367	BM385726	2	1.60	0.001
1372824_at	pleckstrin homology domain containing, family F (with FYVE domain) member 2 (predicted)	Plekhf2_predicted	1132	1814	NM_001108655 /// XM_001072203 /// XM_342803	5q13	1.60	0.013
1399090_at	dynein, cytoplasmic, light intermediate chain 1	Dncli1	4588	7351	AA944459	8	1.60	0.005
1389402_at	AXIN1 up-regulated 1 (predicted)	Axud1_predicted	1323	2119	NM_001108786 /// XM_001077652 /// XM_343504	8q32	1.60	0.018
1386525_at	THO complex 2 (predicted)	Thoc2_predicted	547	876	XM_001058768 /// XM_233081	Xq11	1.60	0.028
1375699_at	centaurin, beta 2	Centb2	540	865	BM389190	---	1.60	0.036
1382308_at	Transcribed locus	---	1110	1777	AI717668	---	1.60	0.002
1380560_at	Transcribed locus	---	541	866	BI289499	---	1.60	0.006
1388027_a_at	reticulon 4	Rtn4	3982	6370	NM_031831 /// XM_001065481	14q22	1.60	0.033
1392038_at	similar to PI-3-kinase-related kinase SMG-1 isoform 2 (predicted) /// similar to PI-3-kinase-related kinase SMG-1	LOC691397 /// RGD1563508_predicted	595	952	XM_001078729 /// XR_007094	1q35	1.60	0.008
1367621_at	death-associated protein kinase 3	Dapk3	796	1273	NM_022546	7q11	1.60	0.011
1383538_at	Transcribed locus, strongly similar to XP_996029.1 PREDICTED: zinc finger protein 650 [Mus musculus]	---	1011	1617	BF545850	3	1.60	0.002
1386042_at	Transcribed locus	---	624	997	AW534967	---	1.60	0.010
1390475_at	RNA guanylyltransferase and 5'-phosphatase (predicted)	Rngtt_predicted	563	900	NM_001107923 /// XM_001057191 /// XM_232865	5q21	1.60	0.006

Table S4. Trophoblast differentiation-associated genes

Affymetrix ID	Gene Title	Gene Symbol	Stem Ave	Dif Ave	GenBank	Chromosomal Location	dif/ stem	t-test
1393587_a_at	similar to RIKEN cDNA 2310047O13 (predicted)	RGD1309605_predicted	870	1391	NM_001106122 /// XM_001072337 /// XM_214513	17q12.3	1.60	0.016
1392681_at	RAB2, member RAS oncogene family	Rab2	2316	3701	NM_031718	5q13	1.60	0.022
1390777_at	sterol-C5-desaturase (fungal ERG3, delta-5-desaturase) homolog (S. cerevisae)	Sc5d	1147	1832	NM_053642	8q22	1.60	0.045
1367980_at	rabaptin, RAB GTPase binding effector protein 1	Rabep1	829	1323	NM_019124	10q24	1.60	0.025
1369986_at	hydroxyacyl glutathione hydrolase	Hagh	511	815	NM_033349	10q12	1.59	0.031
1367725_at	serine/threonine-protein kinase pim-3	Pim3	1261	2011	NM_022602	7q34	1.59	0.005
1372719_at	---	---	1052	1677	BF283702	---	1.59	0.005
1398865_at	unc-50 homolog (C. elegans)	Unc50	2724	4342	NM_138919	9q21	1.59	0.000
1371530_at	keratin complex 2, basic, gene 8	Krt2-8	14974	23855	NM_199370	7q36	1.59	0.008
1397405_at	DEAD (Asp-Glu-Ala-Asp) box polypeptide 17	Ddx17	514	818	NM_001015018 /// XM_001076554 /// XM_235480	7q34	1.59	0.041
1371878_at	mastermind like 1 (Drosophila) (predicted)	Maml1_predicted	581	925	NM_001106997 /// XM_001071703 /// XM_220373	10q22	1.59	0.008
1373535_at	enabled homolog (Drosophila)	Enah	2060	3280	NM_001012150	13q26	1.59	0.006
1382242_at	zinc finger protein 451	Zfp451	574	913	NM_001033705	9q21	1.59	0.006
1387222_at	postsynaptic protein Cript	Cript	972	1545	NM_019907	6q12	1.59	0.001
1369642_at	platelet-activating factor acetylhydrolase, isoform 1b, alpha2 subunit	Pafah1b2	1427	2268	NM_022387	8q22	1.59	0.000
1388002_at	TAO kinase 1	Taok1	759	1205	NM_173327	10q26	1.59	0.006
1385210_at	dedicator of cytokinesis 5 (predicted)	Dock5_predicted	862	1369	NM_001107274 /// XM_001067769 /// XM_224311	15p12	1.59	0.024
1373950_at	zinc finger protein 496 (predicted)	Zfp496_predicted	1169	1856	XM_001075266 /// XM_220512	10q22	1.59	0.035
1372964_at	AT rich interactive domain 5B (Mrf1 like) (predicted)	Arid5b_predicted	3086	4896	BI294751	20	1.59	0.001
1368837_at	AT rich interactive domain 4B (Rbp1 like)	Arid4b	849	1345	NM_053421	17q12.1	1.59	0.001
1394731_at	casein kinase 1, gamma 3	Csnk1g3	652	1034	NM_022855	18q11	1.59	0.000
1379449_at	RB1-inducible coiled-coil 1 (predicted)	Rb1cc1_predicted	1356	2149	NM_001107901 /// XM_001054971 /// XM_001061388 /// XM_001061446 /// XM_232667	5q12	1.58	0.008
1378096_at	kinesin family member 5B	Kif5b	889	1407	NM_057202	17q12.1	1.58	0.005
1386952_a_at	dynein, cytoplasmic, intermediate chain 2	Dncic2	2752	4355	NM_053880	3q21	1.58	0.001
1389138_at	SEC22 vesicle trafficking protein-like 1 (S. cerevisiae)	Sec22l1	710	1124	AA945574	2	1.58	0.009
1390526_at	kelch-like 9 (Drosophila) (predicted)	Klhl9_predicted	1168	1847	NM_001107944 /// XM_001054585 /// XM_233157	5q32	1.58	0.034
1369070_at	peroxisomal biogenesis factor 12	Pex12	677	1069	NM_053921	10q26	1.58	0.000
1373548_at	WD repeat domain 68 (predicted)	Wdr68_predicted	1648	2601	NM_001107057 /// XM_001081566 /// XM_221032	10q32.1	1.58	0.002
1368056_at	tuberous sclerosis 2	Tsc2	697	1099	NM_012680	10q12	1.58	0.034
1371956_at	similar to praja1, RING-H2 motif containing	LOC683077	1124	1772	NM_001101006 /// XM_001058047 /// XM_001058171	---	1.58	0.006

Table S4. Trophoblast differentiation-associated genes

Affymetrix ID	Gene Title	Gene Symbol	Stem Ave	Dif Ave	GenBank	Chromosomal Location	dif/ stem	t-test
1370949_at	myristoylated alanine rich protein kinase C substrate /// similar to Myristoylated alanine-rich C-kinase substrate (MARCKS) (ACAMP-81) /// similar to Myristoylated alanine-rich C-kinase substrate (MARCKS) (Protein kinase C substrate 80 kDa protein)	LOC294446 /// LOC681252 /// Marcks	2617	4123	XM_001060954 /// XM_001061024 /// XM_001061084	20q12	1.58	0.044
1392917_at	ADP-ribosylation factor interacting protein 1	Arfip1	1728	2722	BI302971	2	1.58	0.001
1398970_at	translocation protein 1	Tloc1	2212	3483	NM_001034129	2q24	1.57	0.001
1396692_at	similar to RIKEN cDNA 4933435A13	RGD1306402	1074	1692	NM_001025278	14q22	1.57	0.004
1399126_at	kinesin light chain 1	Klc1	1136	1788	NM_001081972 /// NM_001081973 /// NM_001081974 /// XM_001072327 /// XM_001072624 /// XM_001072695 /// XM_001072733 /// XM_001072766 /// XM_343114	6q32	1.57	0.001
1392486_at	transmembrane protein 106B	Tmem106b	1101	1732	NM_001004267	4q21	1.57	0.037
1374485_at	Transcribed locus	---	2800	4404	AI137762	---	1.57	0.012
1389162_at	histone cell cycle regulation defective interacting protein 5 (predicted)	Hirip5_predicted	826	1298	NM_001106606 /// XM_001070447 /// XM_216202	4q34	1.57	0.001
1393027_at	similar to RIKEN cDNA 1700052N19	RGD1305235	1758	2761	NM_001017447	1p11	1.57	0.004
1379271_at	similar to Suppressor of cytokine signaling 5 (predicted)	RGD1564914_predicted	1043	1638	NM_001109274 /// XM_001058928 /// XM_575987	6q12	1.57	0.014
1385526_at	similar to autophagy 5-like	LOC365601	656	1029	NM_001014250	20q13	1.57	0.020
1383617_at	similar to RIKEN cDNA 4921511I16	RGD1305302	662	1038	NM_001014000	16q11	1.57	0.025
1371952_at	RNA binding motif protein 18 (predicted)	Rbm18_predicted	2207	3463	NM_001107838 /// XM_001080367 /// XM_231176	3p11	1.57	0.002
1377808_at	Similar to IQ motif and WD repeats 1 (predicted)	RGD1561961_predicted	542	850	XM_001071288 /// XM_213926	13q23	1.57	0.022
1368272_at	glutamate oxaloacetate transaminase 1	Got1	2781	4359	NM_012571	1q54	1.57	0.000
1373908_at	---	---	4860	7617	AI407002	---	1.57	0.016
1377593_at	dolichol-phosphate (beta-D) mannosyltransferase 1 (predicted)	Dpm1_predicted	4184	6555	NM_001106544 /// XM_001072659 /// XM_215949	3q42	1.57	0.003
1369571_at	golgi phosphoprotein 3	Golph3	1414	2215	NM_023977	2q16	1.57	0.033
1381878_at	ubinuclein 1 (predicted)	Ubn1_predicted	857	1342	NM_001106977 /// XM_001079088 /// XM_220175	10q12	1.56	0.014
1375297_at	similar to RIKEN cDNA 0610008C08 (predicted)	RGD1565289_predicted	723	1132	XM_343793	Xq31	1.56	0.002
1379739_at	Transcribed locus	---	1509	2360	BF408431	---	1.56	0.003
1390027_at	ubiquitin specific protease 8 (predicted)	Usp8_predicted	1125	1760	NM_001106502 /// XM_001078286 /// XM_215821	3q36	1.56	0.000
1374692_at	Sorting nexin 14 (predicted)	Snx14_predicted	543	849	NM_001108174 /// XM_001065843 /// XM_236461	8q31	1.56	0.005

Table S4. Trophoblast differentiation-associated genes

Affymetrix ID	Gene Title	Gene Symbol	Stem Ave	Dif Ave	GenBank	Chromosomal Location	dif/ stem	t-test
1375687_at	RAB14, member RAS oncogene family	Rab14	8937	13964	BE097926	3	1.56	0.023
1395886_at	ARP3 actin-related protein 3 homolog (yeast)	Actr3	3318	5184	NM_031068	13q11	1.56	0.025
1368029_at	guanine nucleotide binding protein, alpha inhibiting 3 /// guanine nucleotide binding protein, alpha inhibiting 1	Gnai1 /// Gnai3	5317	8306	NM_013106 /// NM_013145	2q34 /// 4q11	1.56	0.000
1371707_at	transportin 2 (importin 3, karyopherin beta 2b) (predicted)	Tnpo2_predicted	2055	3210	NM_001107166 /// XM_001070750 /// XM_222478	19q11	1.56	0.044
1388307_at	serine incorporator 1	Serinc1	4998	7805	NM_182951	20q11	1.56	0.001
1396386_at	Beta-site APP-cleaving enzyme 2	Bace2	1537	2399	NM_001002802	11q12	1.56	0.007
1376885_at	chemokine-like factor super family 4 (predicted)	Cklfsf4_predicted	1025	1599	BE095660	19	1.56	0.007
1389317_at	similar to hypothetical protein FLJ20507 (predicted)	RGD1309744_predicted	1064	1659	NM_001100978 /// XM_001057254 /// XM_001081091 /// XM_230574	3q36	1.56	0.008
1377723_at	Transcribed locus	---	1200	1870	AI234810	---	1.56	0.018
1373087_at	Membrane-associated ring finger (C3HC4) 7	Mar7	4802	7484	NM_001012087	3q21	1.56	0.000
1375441_at	seryl-aminoacyl-tRNA synthetase 1	Sars1	4173	6504	NM_001007606	2q34	1.56	0.015
1389064_at	fem-1 homolog c (C.elegans) (predicted)	Fem1c_predicted	1055	1643	NM_001106932 /// XM_001053664 /// XM_228396	18q11	1.56	0.010
1372055_at	similar to P-Rex1 (predicted)	RGD1306534_predicted	835	1300	XM_230873 /// XR_008866	3q42	1.56	0.007
1370993_at	laminin, gamma 1	Lamc1	590	918	XM_001071300 /// XM_341133	13q21	1.56	0.011
1389742_at	Transcribed locus	---	957	1489	AW520758	---	1.56	0.002
1398330_at	syntaxin binding protein 1	Stxbp1	602	936	NM_013038	3p11	1.56	0.007
1372986_at	Jun dimerization protein 2	Jundp2	2616	4069	NM_053894	6q31	1.56	0.028
1399113_at	similar to chr2 synaptotagmin (predicted)	RGD1565705_predicted	1223	1900	AI111683	6	1.55	0.002
1383825_at	radixin	Rdx	993	1543	NM_001005889	8q24	1.55	0.025
1398814_at	RAB11a, member RAS oncogene family	Rab11a	3105	4822	NM_031152	8q24	1.55	0.000
1376665_at	Transcribed locus	---	1318	2047	BG670208	---	1.55	0.008
1373598_at	ubinuclein 1 (predicted)	Ubn1_predicted	730	1133	NM_001106977 /// XM_001079088 /// XM_220175	10q12	1.55	0.005
1372243_at	calcium binding protein 39 (predicted)	Cab39_predicted	1261	1958	NM_001106924 /// XM_001063411 /// XM_217464	9q35	1.55	0.002
1371610_at	tankyrase, TRF1-interacting ankyrin-related ADP-ribose polymerase (predicted)	Tnks_predicted	2371	3678	AI411017	---	1.55	0.003
1377147_at	TBC1 domain family, member 7 (predicted)	Tbc1d7_predicted	524	812	NM_001108411 /// XM_001057252 /// XM_001057308 /// XM_001057362 /// XM_341510	17p12	1.55	0.038
1392509_at	Casitas B-lineage lymphoma-like 1 (predicted)	Cbll1_predicted	778	1206	NM_001108018 /// XM_001073155 /// XM_234039	6q16	1.55	0.019
1371969_at	caldesmon 1	Cald1	4294	6656	BI291848	4	1.55	0.005

Table S4. Trophoblast differentiation-associated genes

Affymetrix ID	Gene Title	Gene Symbol	Stem Ave	Dif Ave	GenBank	Chromosomal Location	dif/ stem	t-test
1382154_at	Transcribed locus, moderately similar to NP_035333.2 protein tyrosine phosphatase, non-receptor type 12 [Mus musculus]	---	6473	10033	BI289625	---	1.55	0.002
1390026_at	Bcl2-associated athanogene 3	Bag3	765	1185	NM_001011936	1q36	1.55	0.005
1389556_at	kinesin-associated protein 3 (predicted)	Kifap3_predicted	761	1179	NM_001105964 /// XM_001070739 /// XM_213920	13q22	1.55	0.000
1376636_at	transforming growth factor, beta receptor 1	Tgfbr1	1419	2199	NM_012775	5q22	1.55	0.010
1387806_at	RAS related protein 1b	Rap1b	6218	9627	NM_134346	7q22	1.55	0.004
1392621_at	similar to FKSG26 protein (predicted)	RGD1309054_predicted	644	997	XM_001079503 /// XM_214707	19q12	1.55	0.008
1368067_at	zinc finger protein 148	Zfp148	1146	1774	NM_031615	11q22	1.55	0.013
1391431_at	transducer of ERBB2, 2	Tob2	568	878	AI712476	---	1.54	0.023
1373998_at	Transcribed locus	---	1107	1710	BM389853	---	1.54	0.004
1388800_at	RAB5A, member RAS oncogene family	Rab5a	2623	4051	NM_022692	9q11	1.54	0.001
1374285_at	Transcribed locus	---	810	1250	AW531275	---	1.54	0.035
1368187_at	glycoprotein (transmembrane) nmb	Gpnmb	3142	4848	NM_133298	4q24	1.54	0.009
1390488_a_at	serine/threonine kinase 38	Stk38	682	1051	NM_001015025	20p12	1.54	0.001
1383091_at	amyloid beta precursor protein (cytoplasmic tail) binding protein 2	Appbp2	1694	2609	XM_001081113 /// XM_220805	10q26	1.54	0.002
1373534_at	similar to SR rich protein	RGD1307395	1228	1890	XM_001055451 /// XM_001055505 /// XM_001055560 /// XM_001060221	5q21	1.54	0.047
1377595_at	sperm specific antigen 2 (predicted)	Ssfa2_predicted	4668	7180	NM_001107738 /// XM_001067501 /// XM_230036	3q24	1.54	0.012
1391262_at	similar to SUMO/sentrin specific protease 5 (predicted) /// similar to SUMO/sentrin specific protease 5	LOC686268 /// LOC690251 /// RGD1564247_predicted	1011	1555	XM_001058612 /// XM_001073192 /// XM_001073854 /// XM_574899	1q43 /// 2q24	1.54	0.006
1394388_at	hypothetical LOC100125371	LOC100125371	1927	2958	AA858844	---	1.54	0.026
1394741_at	heterogeneous nuclear ribonucleoprotein A3	Hnrpa3	552	848	AI409455	3	1.54	0.035
1374224_at	eukaryotic translation initiation factor 2 alpha kinase 4 (predicted)	Eif2ak4_predicted	1176	1805	AI169164	3	1.53	0.003
1373441_at	dynactin 5	Dctn5	2538	3895	NM_001037778	1q36	1.53	0.010
1374408_at	similar to CBF1 interacting corepressor	RGD1309199	716	1097	NM_001007799	3q22	1.53	0.040
1398445_at	autism susceptibility candidate 2 (predicted)	Auts2_predicted	3100	4749	BF420311	---	1.53	0.000
1384122_at	---	---	715	1095	AA996697	---	1.53	0.002
1386287_at	similar to RIKEN cDNA 5033405K12 (predicted)	RGD1311593_predicted	690	1056	NM_001107214 /// XM_001076917 /// XM_223362	14p11	1.53	0.010
1373946_at	similar to RIKEN cDNA 2810037C14	RGD1305915	565	866	NM_001106576 /// XM_001060587 /// XM_001060650 /// XM_231295	4q11	1.53	0.004
1372895_at	similar to RIKEN cDNA 5730469M10	RGD1309676	700	1072	NM_001014140	16p14	1.53	0.003

Table S4. Trophoblast differentiation-associated genes

Affymetrix ID	Gene Title	Gene Symbol	Stem Ave	Dif Ave	GenBank	Chromosomal Location	dif/ stem	t-test
1388677_at	ubiquitin-associated protein 1	Ubap1	820	1255	NM_001012190	5q22	1.53	0.013
1371722_at	similar to CGI-96 protein; gastric cancer antigen Zg14	RGD1311547	963	1473	XM_001077728 /// XM_343298	7q34	1.53	0.005
1379251_at	Transcribed locus	---	582	891	BF398485	---	1.53	0.011
1388801_at	Similar to RIKEN cDNA 9030221M09 gene (predicted)	RGD1305469_predicted	889	1359	XM_001066163 /// XM_223586	14q21	1.53	0.002
1373290_at	similar to Enhancer of zeste homolog 2 (ENX-1)	LOC312299	1051	1605	XM_001073768 /// XM_231705	4q24	1.53	0.021
1390684_at	Transcribed locus	---	1046	1598	AA892642	---	1.53	0.008
1369562_at	hippocalcin-like 1	Hpcal1	4157	6347	NM_017356	6q16	1.53	0.044
1368852_at	DnaJ (Hsp40) homolog, subfamily A, member 1	Dnaja1	2044	3119	NM_022934	5q22	1.53	0.000
1370348_at	ninjurin 1	Ninj1	2024	3086	NM_012867	17p14	1.52	0.005
1370199_at	nucleobindin 1	Nucb1	3689	5622	NM_053463	1q22	1.52	0.027
1388345_at	p21 (CDKN1A)-activated kinase 2	Pak2	3610	5500	AI599882	11	1.52	0.000
1368929_at	nuclear protein localization 4 homolog (S. cerevisiae)	Nploc4	655	998	NM_080577	10q32.3	1.52	0.008
1376129_at	similar to vacuolar protein sorting 13C protein (predicted)	RGD1560364_predicted	929	1414	BE108174	8	1.52	0.028
1374456_at	similar to Protein disulfide-isomerase TXNDC10 precursor (Thioredoxin domain-containing protein 10)	LOC682967	2652	4035	XM_001063895	---	1.52	0.002
1399159_a_at	vesicle-associated membrane protein 3	Vamp3	3519	5352	NM_057097	5q36	1.52	0.007
1373569_at	Transcribed locus	---	863	1312	AI013005	---	1.52	0.001
1371667_at	similar to RIKEN cDNA C530043G21 gene	RGD1311162_predicted	1306	1985	NM_001107187 /// XM_001068245 /// XM_222770	13q22	1.52	0.003
1367465_at	defender against cell death 1	Dad1	2452	3725	NM_138910	15p13	1.52	0.003
1389053_at	similar to hypothetical protein FLJ20627 (predicted)	RGD1309546_predicted	1242	1885	NM_001040128 /// XM_001054801 /// XM_001058471	1p11	1.52	0.004
1383070_at	UBX domain containing 8	Ubxd8	1276	1936	NM_001017445	17p14	1.52	0.009
1382076_at	solute carrier family 37 (glycerol-3-phosphate transporter), member 1	Slc37a1	919	1394	NM_001011944	20p12	1.52	0.002
1372136_at	similar to tetraspanin similar to TM4SF9 (predicted)	RGD1305714_predicted	6519	9883	AA848776	16	1.52	0.001
1369641_at	platelet-activating factor acetylhydrolase, isoform 1b, alpha2 subunit	Pafah1b2	1165	1767	NM_022387	8q22	1.52	0.006
1373150_at	catechol-O-methyltransferase domain containing 1 (predicted)	Comtd1_predicted	604	915	NM_001107249 /// XM_001065422 /// XM_223785	15p16	1.52	0.003
1372484_at	Transcribed locus, strongly similar to XP_234508.3 PREDICTED: similar to polyA polymerase [Rattus norvegicus]	---	2505	3792	AI176231	10	1.51	0.002
1374513_at	septin 7	Sept7	9641	14589	NM_022616	8q13	1.51	0.015
1390228_at	amine oxidase, flavin containing 1 (predicted)	Aof1_predicted	828	1253	NM_001107343 /// XM_001055844 /// XM_225213	17p13	1.51	0.002
1373391_at	transmembrane and coiled-coil domains 1	Tmco1	3354	5075	NM_001009631	13q23	1.51	0.000
1375853_at	similar to CG13957-PA (predicted)	RGD1309995_predicted	1646	2490	XM_001076152 /// XM_235003	7q13	1.51	0.001
1398995_at	similar to retinoid x receptor interacting protein	RGD1307009	926	1399	NM_001013884	17p14	1.51	0.027

Table S4. Trophoblast differentiation-associated genes

Affymetrix ID	Gene Title	Gene Symbol	Stem Ave	Dif Ave	GenBank	Chromosomal Location	dif/ stem	t-test
1387798_a_at	complement receptor related protein	Crry	2780	4200	NM_001005265 /// NM_001005330 /// NM_019301 /// XM_001070455	13q27	1.51	0.004
1379384_at	Sp1 transcription factor	Sp1	1025	1549	NM_012655	7q36	1.51	0.036
1374086_at	Rho GTPase activating protein 21 (predicted)	Arhgap21_predicted	3814	5760	XM_001074395 /// XM_225628	17q12.3	1.51	0.003
1376075_at	Presenilin 1	Psen1	1281	1935	NM_019163	6q31	1.51	0.003
1372966_at	hypothetical LOC298504 (predicted)	RGD1310174_predicted	1312	1980	NM_001106683 /// XM_001054640 /// XM_216525	5q36	1.51	0.001
1371880_at	Sp1 transcription factor	Sp1	2213	3340	NM_012655	7q36	1.51	0.003
1384938_at	Rho GTPase activating protein 1 (predicted)	Arhgap1_predicted	637	962	AI535143	3	1.51	0.005
1386846_at	similar to hypothetical protein MGC36325 (predicted)	RGD1307374_predicted	674	1017	XM_001056597 /// XM_214609	18p12	1.51	0.014
1390029_at	transmembrane protein 110	Tmem110	683	1029	NM_198774	16p16	1.51	0.014
1368046_at	solute carrier family 31 (copper transporters), member 1	Slc31a1	543	819	NM_133600	5q24	1.51	0.010
1388563_at	similar to RIKEN cDNA 1700034P14 (predicted)	RGD1305492_predicted	3780	5699	NM_001106410 /// XM_001066754 /// XM_215485	2q14	1.51	0.001
1383232_at	RAB33B, member of RAS oncogene family (predicted)	Rab33b_predicted	617	929	NM_001108944 /// XM_001054246 /// XM_001054312 /// XM_345208	2q26	1.51	0.000
1398893_at	Nedd4 family interacting protein 1	Ndfip1	4417	6651	NM_001013059	18p11	1.51	0.001
1388893_at	glycosyltransferase 8 domain containing 1	Glt8d1	550	827	NM_001007683	16p16	1.50	0.003
1371637_at	heterochromatin protein 1, binding protein 3	Hp1bp3	764	1149	NM_199108	5q36	1.50	0.007
1387440_at	iron responsive element binding protein 2	Ireb2	943	1417	NM_022863	8q24	1.50	0.007
1375916_at	protein-L-isoaspartate (D-aspartate) O-methyltransferase domain containing 2 (predicted)	Pcmtd2_predicted	583	876	NM_001107810 /// XM_001056609 /// XM_230971	3q43	1.50	0.007
1388903_at	T-complex associated-testis-expressed 1-like (Protein 91/23)	Tcte1l	612	919	NM_001013228	Xq13	1.50	0.011
1383099_at	similar to RIKEN cDNA 1700034P14 (predicted)	RGD1305492_predicted	1315	1975	NM_001106410 /// XM_001066754 /// XM_215485	2q14	1.50	0.001
1398925_at	similar to RIKEN cDNA 1300018I05	RGD1307801	830	1247	NM_001014031	20p12	1.50	0.007
1372901_at	similar to RIKEN cDNA 8030451K01 (predicted)	RGD1565414_predicted	623	934	XM_001054658 /// XM_227139	2q26	1.50	0.012
1373974_at	oxysterol binding protein (predicted)	Osbp_predicted	797	1195	NM_001108927 /// XM_001075837 /// XM_344995	1q43	1.50	0.009
