# Supplementary material for: Phosphatidylinositol 3 kinase modulation of trophoblast cell differentiation
Source: BMC Dev Biol. 2010 Sep 14;10:97. doi: 10.1186/1471-213X-10-97 (PMC2944162; doi:10.1186/1471-213X-10-97)
Supplement: Additional file 5 — Table S5: PI3K signaling: negatively regulated genes [file 1471-213X-10-97-S5.PDF]

Table S5. PI3K signaling: negatively regulated genes

Affymetrix ID	Gene Title	Gene Symbol	Stem Ave	Dif Ave	LY Ave	GenBank	Chromosomal Location	dif/ly	t-test
1394022_at	inhibitor of DNA binding 4	Id4	584	191	955	NM_175582	17p14	5.00	0.000
1376569_at	Kruppel-like factor 2 (lung) (predicted)	Klf2_predicted	163	447	2098	NM_001007684	16p14	4.70	0.000
1375532_at	Inhibitor of DNA binding 2, dominant negative helix-loop-helix protein	Id2	14665	1162	4365	AI008792	6	3.76	0.023
1390205_at	---	---	124	301	1011	BE108876	---	3.36	0.001
1377273_at	Transcribed locus	---	319	319	945	BF389637	---	2.97	0.000
1390151_at	hypothetical protein LOC683034	LOC683034	431	365	1055	XM_001061331 /// XM_001061393	---	2.89	0.000
1373680_at	Transcribed locus	---	216	335	928	AA800708	---	2.77	0.001
1388547_at	claudin 4	Cldn4	2905	591	1564	NM_001012022	12q12	2.65	0.002
1389616_at	Transcribed locus	---	710	970	2567	BF403163	---	2.65	0.004
1383480_at	similar to Gene model 784 (predicted)	RGD1560060_predicted /// RGD1561693_predicted	53	423	1061	XM_001054257 /// XM_001058468	Xq31	2.51	0.005
1389683_at	---	---	338	340	805	AI170768	---	2.37	0.011
1367802_at	serum/glucocorticoid regulated kinase	Sgk	445	484	1128	NM_019232	1p12	2.33	0.000
1368189_at	7-dehydrocholesterol reductase	Dhcr7	216	1037	2392	NM_022389	1q41	2.31	0.013
1374531_at	Transcribed locus	---	1419	474	1093	AA926305	---	2.30	0.001
1385350_at	Synaptogenesis-related mRNA sequence 6	---	365	597	1344	BF398122	---	2.25	0.001
1371694_at	dihydropyrimidinase-like 2	Dpysl2	443	773	1727	NM_001105717 /// XM_001067745 /// XM_573810	15p12	2.23	0.000
1373754_at	Transcribed locus	---	3011	524	1168	AI599232	---	2.23	0.000
1382873_at	CTTNBP2 N-terminal like (predicted)	Cttnbp2nl_predicted	421	759	1678	NM_001107712 /// XM_001066527 /// XM_227556	2q34	2.21	0.013
1372510_at	sulfiredoxin 1 homolog (S. cerevisiae)	Srxn1	1104	2274	5010	NM_001047858 /// XM_001058481 /// XM_215887	3q41	2.20	0.003
1373066_at	Transcribed locus	---	359	1185	2606	AI407797	---	2.20	0.001
1377975_at	Transcribed locus	---	128	408	893	AI100827	---	2.19	0.003
1370315_a_at	stathmin-like 4	Stmn4	183	441	957	XM_001067575 /// XM_001067632 /// XM_001074337 /// XM_341341	15p12	2.17	0.024
1389033_at	similar to RIKEN cDNA 2900010M23 (predicted)	RGD1306917_predicted	2774	536	1154	NM_001108528 /// XM_342099	20p12	2.15	0.002
1387017_at	squalene epoxidase	Sqle	197	626	1347	NM_017136	7q33	2.15	0.000
1368247_at	heat shock 70kD protein 1A /// heat shock 70kD protein 1B (mapped)	Hspa1a /// Hspa1b	1095	2044	4372	NM_031971 /// NM_212504	20p12	2.14	0.000
1370158_at	myosin, heavy polypeptide 10, non-muscle	Myh10	4095	916	1948	NM_031520	10q24	2.13	0.003
1384331_at	sulfiredoxin 1 homolog (S. cerevisiae)	Srxn1	615	1306	2775	NM_001047858 /// XM_001058481 /// XM_215887	3q41	2.12	0.002
1375906_at	hypothetical protein LOC683034	LOC683034	830	509	1080	XM_001061331 /// XM_001061393	20	2.12	0.000
1374864_at	sprouty homolog 2 (Drosophila)	Spry2	2766	2366	4974	NM_001012046	15q22	2.10	0.001
1395957_at	Transcribed locus	---	198	580	1218	AW532142	2	2.10	0.030

Table S5. PI3K signaling: negatively regulated genes

Affymetrix ID	Gene Title	Gene Symbol	Stem Ave	Dif Ave	LY Ave	GenBank	Chromosomal Location	dif/ly	t-test
1369830_at	protein kinase C, eta	Prkch	626	453	950	NM_031085	6q24	2.10	0.001
1372603_at	protein kinase C and casein kinase substrate in neurons 3	Pacsin3	313	396	830	NM_001009966	3q24	2.10	0.000
1383173_at	killer cell lectin-like receptor subfamily C, member 1	Klrc1	164	425	884	BI281630	---	2.08	0.022
1387772_at	calmodulin 1	Calm1	2012	2310	4774	NM_031969	6q31-q32	2.07	0.000
1388836_at	protein kinase C, eta	Prkch	1200	875	1789	NM_031085	6q24	2.05	0.000
1367932_at	3-hydroxy-3-methylglutaryl-Coenzyme A synthase 1	Hmgcs1	511	1966	3999	NM_017268	2q16	2.03	0.005
1395044_at	hypothetical protein LOC679298 /// hypothetical protein LOC688832	LOC679298 /// LOC688832	1649	988	2000	XM_001055692 /// XM_001068496	13q24	2.03	0.015
1378734_at	gastrulation brain homeobox 2	Gbx2	738	858	1718	XM_001066354 /// XM_346072	9q35	2.00	0.014
1394470_at	similar to hypothetical protein DKFZp566A1524 (predicted)	RGD1305961_predicted	214	458	907	NM_001106718 /// XM_001073211 /// XM_216666	6q14	1.98	0.000
1368910_at	protein phosphatase 2C, magnesium dependent, catalytic subunit	Ppm2c	475	645	1276	NM_019372	5q13	1.98	0.001
1370313_at	acyl-CoA thioesterase 7	Acot7	4483	2326	4501	NM_013214	5q36	1.94	0.041
1389367_at	schwannomin interacting protein 1	Schip1	1430	682	1315	XM_001061765 /// XM_001061820 /// XM_001069584 /// XM_215570	2q31	1.93	0.000
1377156_at	similar to transcription factor 7-like 2, T-cell specific, HMG-box /// similar to Transcription factor 7-like 2 (HMG box transcription factor 4) (T-cell-specific transcription factor 4) (TCF-4) (hTCF-4)	LOC679869 /// LOC683733	1768	1525	2928	XM_001054787 /// XM_001054844 /// XM_001072074 /// XM_001072106 /// XM_001072143 /// XM_001072322 /// XM_001072361 /// XM_001072389 /// XM_001072429 /// XM_001072472 /// XM_001072517 /// XM_001072554	1q55	1.92	0.005
1368321_at	early growth response 1	Egr1	170	1230	2355	NM_012551	18q	1.92	0.011
1393037_at	Transcribed locus	---	281	1071	2049	BG373089	8	1.91	0.002
1392264_s_at	serine (or cysteine) peptidase inhibitor, clade E, member 1	Serpine1	233	1226	2347	NM_012620	12q11-q12	1.91	0.003
1383979_at	similar to monoacylglycerol O-acyltransferase 2	LOC683355	1092	638	1218	XM_001065594	---	1.91	0.013
1372044_at	similar to Ser/Thr-rich protein T10 in DGCR region (predicted)	RGD1310348_predicted	209	447	850	NM_001108323 /// XM_001063969 /// XM_341010	11q23	1.90	0.002
1393558_at	integrin, alpha 6	Itga6	836	919	1747	XM_001059353 /// XM_001059408 /// XM_001059465 /// XM_001059528 /// XM_215984	3q22	1.90	0.001
1391167_at	Transcribed locus	---	1333	1409	2674	AW532567	---	1.90	0.005
1376419_at	Transcribed locus	---	775	684	1293	BE116226	---	1.89	0.016
1390441_at	Development and differentiation enhancing (predicted)	Ddef1_predicted	332	676	1278	NM_001044245 /// XM_001066206 /// XM_001066263 /// XM_235296	7q33	1.89	0.011

Table S5. PI3K signaling: negatively regulated genes

Affymetrix ID	Gene Title	Gene Symbol	Stem Ave	Dif Ave	LY Ave	GenBank	Chromosomal Location	dif/ly	t-test
1396803_at	THO complex 2 (predicted)	Thoc2_predicted	525	518	975	XM_001058768 /// XM_233081	Xq11	1.88	0.018
1393238_at	similar to DKFZP434B168 protein (predicted)	RGD1308014_predicted	1039	534	1003	XM_001067002 /// XM_001067053 /// XM_223075	13q27	1.88	0.000
1376606_a_at	similar to e(y)2 protein	LOC682575 /// LOC685258	3324	1370	2568	XM_001062411 /// XM_001062468 /// XM_001062808 /// XM_001062869	7q31	1.87	0.000
1390416_at	Solute carrier family 25, member 30	Slc25a30	1284	1351	2527	NM_001013187	15q11	1.87	0.000
1372554_at	similar to RW1 protein (predicted)	RGD1309266_predicted	1256	521	974	XM_001056771 /// XM_237056	9q21	1.87	0.001
1375358_at	Transcribed locus	---	3220	4009	7455	AA998150	---	1.86	0.000
1389172_at	enigma homolog	Enh	418	666	1239	AI179391	---	1.86	0.014
1390929_at	Transcribed locus	---	310	627	1161	BI275447	---	1.85	0.021
1395762_at	Transcribed locus	---	535	428	791	BG670091	---	1.85	0.005
1380148_at	Wolf-Hirschhorn syndrome candidate 1 (predicted)	Whsc1_predicted	1996	3207	5905	BE118688	14	1.84	0.001
1398447_at	Transcribed locus, strongly similar to XP_913560.1 PREDICTED: hypothetical protein LOC109169 [Mus musculus]	---	264	576	1058	AA801323	---	1.84	0.008
1388742_at	---	---	984	559	1025	AA945877	---	1.83	0.004
1390207_at	large tumor suppressor 2 (predicted)	Lats2_predicted	1414	1996	3653	NM_001107267 /// XM_001063422 /// XM_224169	15p12	1.83	0.000
1379450_at	similar to cDNA sequence BC003236 (predicted)	RGD1310822_predicted	909	1472	2690	BM383195	2	1.83	0.014
1386978_at	BCL2/adenovirus E1B 19 kDa-interacting protein 3-like	Bnip3l	1980	2369	4321	NM_080888	15p12	1.82	0.000
1388888_at	---	---	562	553	1007	BI274615	---	1.82	0.003
1386890_at	S100 calcium binding protein A10 (calpactin)	S100a10	5076	3249	5918	NM_031114	2	1.82	0.003
1371785_at	tumor necrosis factor receptor superfamily, member 12a	Tnfrsf12a	2101	1192	2167	NM_181086	10q12	1.82	0.007
1381428_a_at	Transcribed locus	---	344	3275	5923	AI177364	---	1.81	0.006
1383353_at	ephrin B2 (predicted)	Efnb2_predicted	1354	588	1061	NM_001107328 /// XM_001067856 /// XM_225050	16q12.5	1.81	0.001
1371186_at	integrin, alpha 6	Itga6	665	707	1276	XM_001059353 /// XM_001059408 /// XM_001059465 /// XM_001059528 /// XM_215984	3q22	1.80	0.000
1398333_at	Transcribed locus	---	209	861	1554	AI598434	6	1.80	0.008
1388882_at	FK506 binding protein 3 (predicted)	Fkbp3_predicted	4544	894	1610	NM_001106736 /// XM_001080093 /// XM_216717	6q24	1.80	0.003
1387279_at	F11 receptor	F11r	1232	963	1732	NM_053796	13q24	1.80	0.002
1378697_at	RNA binding motif, single stranded interacting protein 2	Rbms2	298	600	1079	NM_001025403	7q11	1.80	0.006
1379719_at	Transcribed locus	---	2366	2314	4157	AI408386	---	1.80	0.003
1382680_at	adipose differentiation-related protein	ADRP	258	625	1122	BG673602	5	1.80	0.000

Table S5. PI3K signaling: negatively regulated genes

Affymetrix ID	Gene Title	Gene Symbol	Stem Ave	Dif Ave	LY Ave	GenBank	Chromosomal Location	dif/ly	t-test
1389164_at	hect domain and RLD 3 (predicted)	Herc3_predicted	458	520	931	NM_001108631 /// XM_001060220 /// XM_001060283 /// XM_001060353 /// XM_001060411 /// XM_001060473 /// XM_342701	4q24	1.79	0.013
1380644_at	Transcribed locus	---	444	555	992	AI178542	---	1.79	0.002
1368115_at	claudin 3	Cldn3	2665	735	1311	NM_031700	12	1.78	0.043
1370808_at	cytochrome b5 reductase 3	Cyb5r3	2514	2170	3868	NM_138877	7q34	1.78	0.001
1398389_at	Transcribed locus	---	1229	1443	2570	AI236136	10	1.78	0.007
1373829_at	fibroblast growth factor receptor 2	Fgfr2	3813	877	1557	NM_001109892 /// NM_001109893 /// NM_001109894 /// NM_001109895 /// NM_001109896 /// NM_012712 /// XM_001077699 /// XM_001077810 /// XM_001079420 /// XM_001079450 /// XM_001079458 /// XM_001079467 /// XM_001079477 /// XM_001079488 /// XM_001079498 /// XM_001079510 /// XM_001079521 /// XM_001079530 /// XM_341940	1q37	1.78	0.003
1372507_at	T-cell leukemia translocation altered gene	Tcta	169	485	860	NM_001014005	16q12.5	1.77	0.000
1371424_at	similar to RIKEN cDNA 3110005O21	RGD1304823	882	580	1028	NM_001037196	1q36	1.77	0.012
1375523_at	similar to Myristoylated alanine-rich C-kinase substrate (MARCKS) (ACAMP-81) /// similar to Myristoylated alanine-rich C-kinase substrate (MARCKS) (Protein kinase C substrate 80 kDa protein)	LOC294446 /// LOC681252	2074	2782	4906	XM_001060954 /// XM_001061024 /// XM_001061084	20q12	1.76	0.002
1398910_at	STIP1 homology and U-Box containing protein 1	Stub1	2687	1279	2252	NM_001025625	10q12	1.76	0.001
1373807_at	Vascular endothelial growth factor A	Vegfa	391	490	862	NM_001110333 /// NM_001110334 /// NM_001110335 /// NM_001110336 /// NM_031836	9q12	1.76	0.006
1398427_at	Myocyte enhancer factor 2D	Mef2d	828	479	841	NM_030860 /// XM_001069089 /// XM_001069142	2q34	1.76	0.007
1379506_at	Transcribed locus	---	349	492	865	AA963909	---	1.76	0.046
1379424_at	similar to DKFZP434B168 protein (predicted)	RGD1308014_predicted	844	458	802	XM_001067002 /// XM_001067053 /// XM_223075	13q27	1.75	0.002

Table S5. PI3K signaling: negatively regulated genes

Affymetrix ID	Gene Title	Gene Symbol	Stem Ave	Dif Ave	LY Ave	GenBank	Chromosomal Location	dif/ly	t-test
1391935_at	similar to eukaryotic translation initiation factor 4E member 3	LOC297481	206	486	852	NM_001106612 /// XM_001077522 /// XM_216226	4q34	1.75	0.006
1388918_at	high density lipoprotein binding protein	Hdlbp	1637	1483	2595	BG378074	9	1.75	0.002
1388157_at	myristoylated alanine rich protein kinase C substrate	Marcks	836	1313	2296	BE111706	6	1.75	0.007
1371776_at	phosphatidylinositol 3-kinase, regulatory subunit, polypeptide 1	Pik3r1	1033	1168	2042	AA819268	2	1.75	0.010
1376768_at	Transcribed locus	---	250	570	996	BM386807	---	1.75	0.005
1372586_at	Transcribed locus	---	500	542	947	AI411995	---	1.75	0.006
1388837_at	Solute carrier family 44, member 2 (predicted)	Slc44a2_predicted	1030	581	1014	XM_001077428 /// XM_343355	8q13	1.75	0.013
1397164_at	Transcribed locus, weakly similar to XP_984565.1 PREDICTED: hypothetical protein LOC66961 [Mus musculus]	---	430	632	1101	AI175779	---	1.74	0.033
1369304_at	6-pyruvoyl-tetrahydropterin synthase	Pts	276	517	900	NM_017220	8q23	1.74	0.039
1388853_at	mitochondrial ribosomal protein L54 (predicted)	Mrpl54_predicted	2833	581	1010	NM_001106770 /// XM_001075791 /// XM_216854	7q11	1.74	0.018
1373181_at	similar to actin filament associated protein; actin filament-associated protein, 110 kDa (predicted)	RGD1311580_predicted	1667	1302	2260	NM_001106142 /// XM_001058822 /// XM_238021	18q12.1	1.74	0.012
1371358_at	glycoprotein, synaptic 2	Gpsn2	1676	1965	3408	NM_138549 /// XM_001072883	19q11	1.73	0.002
1393407_at	F-box and WD-40 domain protein 2 (predicted)	Fbxw2_predicted	743	579	1004	NM_001107835 /// XM_001080206 /// XM_231162	3p11	1.73	0.006
1372718_at	similar to anaphase promoting complex subunit 13	LOC685029	968	688	1192	XM_001060533 /// XM_001060592 /// XM_001068292 /// XM_001068345	8q32	1.73	0.000
1384059_at	PC4 and SFRS1 interacting protein 1	Psip1	286	546	945	BE115159	---	1.73	0.003
1375707_at	---	---	1389	9639	16684	AA817993	---	1.73	0.000
1371536_at	calcium regulated heat stable protein 1	Carhsp1	886	1184	2046	NM_152790	10q12	1.73	0.007
1373106_at	zinc finger protein 36, C3H type-like 2	Zfp36l2	1507	1036	1789	NM_001036626 /// XM_001060793	6q12	1.73	0.005
1383240_at	integrin, alpha 6	Itga6	2332	2567	4428	XM_001059353 /// XM_001059408 /// XM_001059465 /// XM_001059528 /// XM_215984	3q22	1.73	0.000
1368519_at	serine (or cysteine) peptidase inhibitor, clade E, member 1	Serpine1	349	2238	3858	NM_012620	12q11-q12	1.72	0.001
1367839_at	farnesyl diphosphate farnesyl transferase 1	Fdft1	402	1503	2587	NM_019238	15p12	1.72	0.003
1368277_at	protein phosphatase 3, catalytic subunit, alpha isoform	Ppp3ca	555	669	1150	NM_017041	2q43	1.72	0.002
1373676_at	Transcribed locus	---	319	769	1323	AI712626	---	1.72	0.001
1390902_at	Transcribed locus	---	1670	661	1137	BI275056	---	1.72	0.002

Table S5. PI3K signaling: negatively regulated genes

Affymetrix ID	Gene Title	Gene Symbol	Stem Ave	Dif Ave	LY Ave	GenBank	Chromosomal Location	dif/ly	t-test
1388943_at	chromatin accessibility complex 1 (predicted)	Chrac1_predicted	681	484	831	XM_001072895 /// XM_235400	7q34	1.72	0.015
1379471_at	similar to RIKEN cDNA 5730469D23 (predicted)	RGD1308324_predicted	2251	2111	3626	NM_001107213 /// XM_001074883 /// XM_223308	14p21	1.72	0.002
1372771_at	Transcribed locus	---	329	1010	1736	BI279659	---	1.72	0.040
1381969_at	similar to Recombining binding protein suppressor of hairless (J kappa-recombination signal binding protein) (RBP-J kappa)	LOC679028	3246	1609	2762	XM_001053717 /// XM_001053777 /// XM_001053835	---	1.72	0.007
1372096_at	oxidative-stress responsive 1 (predicted)	Oxsr1_predicted	874	965	1656	NM_001108194 /// XM_001078098 /// XM_236687	8q32	1.72	0.002
1372556_at	hypothetical protein LOC502374	LOC502374	2346	820	1405	XR_007265 /// XR_009623	1q41	1.71	0.000
1368438_at	phosphodiesterase 10A	Pde10a	144	530	908	NM_022236	1q11	1.71	0.001
1372106_at	EH-domain containing 4	Ehd4	428	1265	2163	NM_139324	3q35	1.71	0.006
1368275_at	sterol-C4-methyl oxidase-like	Sc4mol	1030	5389	9202	NM_080886	16p13	1.71	0.019
1373571_at	reticulon 3	Rtn3	581	658	1122	NM_001009953 /// NM_080909	1q43	1.70	0.021
1399150_at	similar to RIKEN cDNA A630054L15; hypothetical protein MGC38041 (predicted)	RGD1306064_predicted	414	624	1062	XM_001057380 /// XM_224581	16p16	1.70	0.026
1372228_at	acetylserotonin O-methyltransferase-like (predicted)	Asmtl_predicted	829	1002	1703	NM_001105915 /// XM_001075108 /// XM_213725	12q11	1.70	0.028
1383096_at	amyloid beta (A4) precursor-like protein 2	Aplp2	550	551	937	XM_001055798 /// XM_001055858 /// XM_001055911 /// XM_001055967 /// XM_001056031 /// XM_001056087 /// XM_001056156 /// XM_001056214 /// XM_001056279 /// XM_343513	8q13	1.70	0.002
1384392_at	cytochrome P450, family 26, subfamily b, polypeptide 1	Cyp26b1	719	912	1550	NM_181087	4q34	1.70	0.004
1370912_at	heat shock 70kD protein 1B (mapped)	Hspa1b	839	1562	2655	NM_212504	20p12	1.70	0.000
1382175_at	Similar to Wilms tumor 1-associating protein (WT1-associated protein) homolog) (predicted)	RGD1563824_predicted	1487	1779	3022	XM_001068104 /// XM_574313	1q11	1.70	0.005
1373858_at	karyopherin (importin) beta 1	Kpnb1	1345	1180	2002	BE109064	10	1.70	0.004
1387636_a_at	P11 protein	Cdtw1	921	785	1331	NM_134395	17p12	1.69	0.002
1372861_at	phosphatidylinositol-4-phosphate 5-kinase, type I, gamma	Pip5k1c	934	1115	1888	NM_001009967 /// NM_001033970	7q11	1.69	0.000
1398330_at	syntaxin binding protein 1	Stxbp1	602	936	1584	NM_013038	3p11	1.69	0.002
1369268_at	activating transcription factor 3	Atf3	545	517	873	NM_012912 /// XM_001068451	13q27	1.69	0.002
1395502_at	protein phosphatase 2, regulatory subunit B (B56), delta isoform	Ppp2r5d	727	821	1386	XM_001062510 /// XM_001065844	9q12	1.69	0.000

Table S5. PI3K signaling: negatively regulated genes

Affymetrix ID	Gene Title	Gene Symbol	Stem Ave	Dif Ave	LY Ave	GenBank	Chromosomal Location	dif/ly	t-test
1399166_a_at	similar to B-cell CLL/lymphoma 7B	LOC368001	346	560	944	NM_001109021 /// XM_001077499 /// XM_347165	12q12	1.69	0.000
1372419_at	vaccinia related kinase 3	Vrk3	302	531	895	NM_001005561	1q22	1.68	0.000
1367663_at	proteasome (prosome, macropain) 28 subunit, alpha	Psme1	1410	565	949	NM_017264	15p13	1.68	0.018
1376026_at	downstream neighbor of SON	Donson	2162	1990	3323	NM_001008287	11q11	1.67	0.002
1376655_at	Transcribed locus	---	896	1012	1690	AA850435	---	1.67	0.005
1388199_at	tumor-associated calcium signal transducer 1	Tacstd1	3655	2058	3435	NM_138541	6q12	1.67	0.017
1371615_at	diacylglycerol O-acyltransferase homolog 2 (mouse)	Dgat2	1647	571	953	NM_001012345	1q32	1.67	0.006
1398833_at	membrane-bound transcription factor peptidase, site 1	Mbtps1	1054	802	1338	NM_053569	19q12	1.67	0.004
1371352_at	high mobility group nucleosomal binding domain 2	Hmgn2	9747	5287	8794	NM_001025624	5q36	1.66	0.000
1376812_at	Transcribed locus	---	747	740	1228	BF419731	---	1.66	0.016
1390218_at	similar to hypothetical protein (predicted)	RGD1310440_predicted	1433	523	867	XM_001057055 /// XM_342912	5q36	1.66	0.002
1372931_at	PRA1 domain family 2 (predicted)	Praf2_predicted	801	511	844	NM_001109013 /// XM_001058767 /// XM_346274	Xq13	1.65	0.003
1376882_at	---	---	476	703	1162	BF398580	---	1.65	0.004
1383253_at	solute carrier family 30 (zinc transporter), member 1	Slc30a1	2375	2130	3519	AI179795	13	1.65	0.019
1384188_at	---	---	90	486	802	AW142766	---	1.65	0.025
1367661_at	S100 calcium binding protein A6 (calcyclin)	S100a6	6024	2709	4467	NM_053485	2q34	1.65	0.003
1377617_at	purine rich element binding protein A (predicted)	Pura_predicted	2274	2792	4598	XM_001063244 /// XM_226016	18p11	1.65	0.001
1375970_at	Transcribed locus	---	910	847	1391	AI010423	---	1.64	0.001
1390048_at	serine/arginine repetitive matrix 2 (predicted)	Srrm2_predicted	4978	2618	4299	XM_001056343 /// XM_220207	10q12	1.64	0.040
1388945_at	similar to 1300014I06Rik protein	RGD1311307	496	1691	2775	NM_001025719	17p12	1.64	0.000
1370287_a_at	tropomyosin 1, alpha	Tpm1	2221	1727	2832	NM_001034068 /// NM_001034069 /// NM_001034070 /// NM_001034071 /// NM_001034072 /// NM_001034073 /// NM_001034074 /// NM_001034075 /// NM_019131	8q24	1.64	0.004
1373479_at	Protein phosphatase 3, catalytic subunit, alpha isoform	Ppp3ca	997	1487	2438	NM_017041	2q43	1.64	0.004
1387130_at	solute carrier family 39 (iron-regulated transporter), member 1	Slc40a1	545	1669	2731	NM_133315	9q22	1.64	0.006
1373324_at	dual specificity phosphatase 14 (predicted) /// similar to Dual specificity protein phosphatase 14 (Mitogen-activated protein kinase phosphatase 6) (MAP kinase phosphatase 6) (MKP-6)	Dusp14_predicted /// LOC499736 /// LOC681749	234	550	898	NM_001079893 /// XM_001058250 /// XM_001081088 /// XM_340861 /// XM_575070	10q26	1.63	0.011
1370358_at	two pore channel 1	Tpcn1	1722	1189	1940	NM_139332	12q16	1.63	0.025
1386996_at	myosin light chain, regulatory B	Mrlcb	4629	1334	2173	NM_017343	9q38	1.63	0.007

Table S5. PI3K signaling: negatively regulated genes

Affymetrix ID	Gene Title	Gene Symbol	Stem Ave	Dif Ave	LY Ave	GenBank	Chromosomal Location	dif/ly	t-test
1388722_at	DnaJ (Hsp40) homolog, subfamily B, member 1 (predicted)	Dnajb1_predicted	1560	1621	2638	NM_001108441 /// XM_001069407 /// XM_341663	19q11	1.63	0.000
1376784_at	Formin binding protein 1	Fnbp1	279	541	878	NM_138914	3p12	1.62	0.041
1370156_at	prion protein	Prnp	1723	6476	10511	NM_012631	3q36	1.62	0.000
1375967_a_at	dual specificity phosphatase 22 (predicted)	Dusp22_predicted	1275	742	1204	NM_001108412 /// XM_001061713 /// XM_341523	17p12	1.62	0.022
1372462_at	acetyl-Coenzyme A acetyltransferase 2	Acat2	736	1876	3037	NM_001006995	1q11	1.62	0.000
1378668_at	Transcribed locus	---	485	526	849	BG672426	---	1.62	0.016
1387793_at	solute carrier family 9 (sodium/hydrogen exchanger), isoform 3 regulator 1	Slc9a3r1	2455	731	1180	NM_021594	10q32.2	1.61	0.001
1392469_at	F-box and WD-40 domain protein 2 (predicted)	Fbxw2_predicted	1077	925	1489	NM_001107835 /// XM_001080206 /// XM_231162	3p11	1.61	0.006
1372743_at	sorting nexin 5 (predicted)	Snx5_predicted	4394	4324	6964	NM_001106518 /// XM_001053951 /// XM_215872	3q41	1.61	0.001
1392668_at	recombining binding protein suppressor of hairless (Drosophila) (predicted) /// similar to Recombining binding protein suppressor of hairless (J kappa-recombination signal binding protein) (RBP-J kappa)	LOC679028 /// Rbpsuh_predicted	1303	566	912	NM_001106631 /// XM_001053717 /// XM_001053777 /// XM_001053835 /// XM_001064167 /// XM_232595	5q11	1.61	0.009
1382268_at	A kinase (PRKA) anchor protein 13	Akap13	492	572	918	NM_001106271 /// XM_001063231 /// XM_214969	1q31	1.60	0.009
1384442_at	claudin 6 (predicted)	Cldn6_predicted	1803	1152	1844	NM_001102364 /// XM_001055688 /// XM_220202	10q12	1.60	0.015
1387844_at	LIM and SH3 protein 1	Lasp1	1894	1447	2316	NM_032613	10q31	1.60	0.007
1375723_at	Transcribed locus	---	535	1485	2372	AI385171	---	1.60	0.001
1371966_at	protein-L-isoaspartate (D-aspartate) O-methyltransferase 1	Pcmt1	614	572	913	NM_013073	1p13	1.60	0.041
1387261_at	protein phosphatase 3, catalytic subunit, beta isoform	Ppp3cb	324	551	877	NM_017042	15p16	1.59	0.019
1381976_at	kinesin family member 21A (predicted)	Kif21a_predicted	490	674	1072	NM_001106790 /// XM_001056804 /// XM_217022	7q35	1.59	0.018
1388872_at	isopentenyl-diphosphate delta isomerase	Idi1	569	1575	2504	BI290053	17	1.59	0.003
1392818_at	growth arrest specific 5	Gas5	2842	760	1206	NR_002704	13q22	1.59	0.007
1376894_at	pallidin	Pldn	444	611	968	NM_001025714	3q35	1.59	0.000
1375396_at	pumilio 1 (Drosophila) (predicted)	Pum1_predicted	1870	1886	2990	NM_001108684 /// XM_001063603 /// XM_342928	5q36	1.58	0.013
1383635_at	DEAD (Asp-Glu-Ala-Asp) box polypeptide 59	Ddx59	743	595	941	NM_001005535	13q13	1.58	0.017
1395613_at	Transcribed locus	---	591	657	1039	BF408015	19	1.58	0.032
1398441_at	non-catalytic region of tyrosine kinase adaptor protein 2 (predicted)	Nck2_predicted	421	639	1011	NM_001108216 /// XM_001059817 /// XM_237115	9q22	1.58	0.006
1367538_at	---	---	246	821	1295	BF284303	---	1.58	0.033

Table S5. PI3K signaling: negatively regulated genes

Affymetrix ID	Gene Title	Gene Symbol	Stem Ave	Dif Ave	LY Ave	GenBank	Chromosomal Location	dif/ly	t-test
1389430_at	Transcribed locus	---	230	1241	1953	AI176172	---	1.57	0.001
1371918_at	CD99 antigen	Cd99	508	659	1035	XM_001061998	---	1.57	0.015
1395455_at	heterogeneous nuclear ribonucleoprotein H3 (2H9) (predicted)	Hnrph3_predicted	1531	947	1487	NM_001108532 /// XM_001074684 /// XM_342131	20p11	1.57	0.001
1398963_at	---	---	2465	1377	2162	BM392226	1	1.57	0.001
1395165_at	PC4 and SFRS1 interacting protein 1	Psip1	735	1479	2322	NM_175765	5q31	1.57	0.007
1374117_at	brain-specific angiogenesis inhibitor 1-associated protein 2	Baiap2	599	1215	1904	NM_057196	10q32.3	1.57	0.001
1388492_at	TNFAIP3 interacting protein 1 (predicted)	Tnip1_predicted	840	829	1299	NM_001108826 /// XM_001073849 /// XM_343894	10q22	1.57	0.000
1371975_at	Baz2a_predicted	Rbms2_predicted	563	1504	2355	BG377343	7	1.57	0.037
1367775_at	alpha-methylacyl-CoA racemase	Amacr	364	558	873	NM_012816	2q16	1.57	0.003
1372484_at	ddx5 gene	Ddx5	2505	3792	5920	AI176231	10	1.56	0.033
1390489_at	Synaptotagmin binding, cytoplasmic RNA interacting protein	Syncrip	1532	808	1262	NM_001047916 /// XM_001065902 /// XM_343446	8q31	1.56	0.026
1373466_at	calpastatin	Cast	390	570	888	NM_001033715 /// NM_001033716 /// NM_053295	2q11	1.56	0.004
1372609_at	protein phosphatase 2, regulatory subunit B (B56), delta isoform	Ppp2r5d	1451	1702	2652	XM_001062510 /// XM_001065844	9q12	1.56	0.004
1371304_a_at	myosin, light polypeptide 6, alkali, smooth muscle and non-muscle (predicted) /// similar to myosin, light polypeptide 6, alkali, smooth muscle and non-muscle /// similar to myosin light chain 1 slow a	LOC684520 /// LOC685867 /// LOC685883 /// Myl6_predicted	6120	4219	6565	NM_001100983 /// NM_001109484 /// NM_001109486 /// XM_001053789 /// XM_001065562 /// XM_001065619 /// XM_001070810 /// XM_343144	13p11 /// 7q11	1.56	0.001
1390776_at	Iroquois related homeobox 3 (Drosophila) (predicted)	Irx3_predicted	2143	1451	2256	NM_001107413 /// XM_001061997 /// XM_226322	19p11	1.55	0.005
1375879_at	G protein-coupled receptor 48	Gpr48	512	507	787	BE111762	3	1.55	0.005
1370057_at	cysteine and glycine-rich protein 1	Csrp1	2701	1064	1653	NM_017148	13q13	1.55	0.013
1393068_at	Transcribed locus	---	824	834	1294	AA956927	---	1.55	0.016
1393131_at	similar to Flt3 interacting zinc finger protein 1 (predicted)	RGD1306359_predicted	885	626	971	NM_001106223 /// XM_001059521 /// XM_218199	1q12	1.55	0.033
1375692_at	mitogen activated protein kinase 1	Mapk1	3071	2421	3754	AI229025	11	1.55	0.002
1384213_at	programmed cell death 6 interacting protein	Pdcd6ip	819	1131	1753	NM_001029910 /// XM_001076624	---	1.55	0.002
1395352_at	COP9 (constitutive photomorphogenic) homolog, subunit 3 (Arabidopsis thaliana)	Cops3	1436	1052	1628	NM_001004200	10q22	1.55	0.001
1372100_at	transmembrane protein 50A (predicted)	Tmem50a_predicted	1259	687	1063	XM_001067840 /// XM_216545	5q36	1.55	0.002
1376593_at	Transcribed locus	---	879	1653	2554	BF283990	---	1.54	0.001
1368122_at	ring finger protein 103	Rnf103	369	602	930	NM_053438 /// XM_001062640	4q33	1.54	0.002

Table S5. PI3K signaling: negatively regulated genes

Affymetrix ID	Gene Title	Gene Symbol	Stem Ave	Dif Ave	LY Ave	GenBank	Chromosomal Location	dif/ly	t-test
1369978_at	phosphoribosyl pyrophosphate synthetase-associated protein 2	Prpsap2	1468	804	1240	NM_057131	10q23	1.54	0.004
1374424_at	SKB1 homolog (S. pombe) (predicted)	Skb1_predicted	2089	668	1028	BE112720	15	1.54	0.014
1376868_at	Cobl-like 1 (predicted)	Cobll1_predicted	2972	957	1472	NM_001107733 /// XM_001056368 /// XM_229988	3q21	1.54	0.016
1398907_at	ORM1-like 2 (S. cerevisiae) (predicted)	Ormdl2_predicted	971	1113	1711	NM_001105940 /// XM_001071533 /// XM_213832	7q11	1.54	0.001
1382144_at	mitochondrial ribosomal protein L47	Mrpl47	2123	933	1434	NM_001037183	2q25	1.54	0.010
1389984_at	similar to jumonji protein	LOC681740	1800	1230	1890	XM_001056690 /// XM_001056762 /// XM_001056826 /// XM_001056897 /// XM_001056960	---	1.54	0.004
1389337_at	Transcribed locus	---	2159	846	1299	AA946032	---	1.54	0.001
1375739_at	EH-domain containing 4	Ehd4	1625	4072	6252	NM_139324	3q35	1.54	0.013
1373178_at	Transcribed locus	---	2062	5052	7755	AA945183	---	1.54	0.000
1369981_at	immunoglobulin (CD79A) binding protein 1	Igbp1	820	974	1492	NM_031624	Xq31	1.53	0.010
1379269_at	CD2-associated protein	Cd2ap	3030	1658	2536	NM_181475	9q12	1.53	0.000
1367898_at	BCL2/adenovirus E1B 19 kDa-interacting protein 3-like	Bnip3l	1915	2079	3174	NM_080888	15p12	1.53	0.003
1377818_at	Transcribed locus	---	185	548	836	AW532426	---	1.53	0.001
1383062_at	glutaredoxin 2 (thioltransferase)	Glrx2	849	1966	2998	BE105102	13	1.52	0.018
1381100_at	Rho guanine nucleotide exchange factor (GEF) 12	Arhgef12	589	1132	1725	BE108751	---	1.52	0.007
1371430_at	dystroglycan 1	Dag1	1484	959	1461	XM_001074892 /// XM_343483	8q32	1.52	0.005
1371632_at	similar to Coronin, actin binding protein 1C (predicted)	RGD1564490_predicted	7727	3176	4830	NM_001109327 /// XM_001080440 /// XM_577254	12q16	1.52	0.028
1387848_at	3-hydroxy-3-methylglutaryl-Coenzyme A reductase	Hmgcr	407	1203	1828	NM_013134	2q12	1.52	0.019
1374846_at	cardiac lineage protein 1	Clp1	4288	4790	7265	NM_001025136	10q32.1	1.52	0.001
1388677_at	ubiquitin-associated protein 1	Ubap1	820	1255	1903	NM_001012190	5q22	1.52	0.010
1388408_at	similar to RIKEN cDNA 1110020C13	RGD1307129	3435	5624	8529	NM_001008379	7q36	1.52	0.002
1372801_at	COMM domain containing 10	Commd10	1689	1463	2218	NM_001004276	18q11	1.52	0.031
1384413_at	similar to solute carrier family 35, member A5 (predicted)	RGD1564361_predicted	290	917	1390	XM_001066912 /// XM_573283	11q21	1.52	0.008
1372544_at	Transcribed locus	---	554	670	1014	AI410305	---	1.51	0.029
1367545_at	G protein-coupled receptor 21 (predicted)	Gpr21_predicted	596	1030	1559	NM_001107841 /// XM_001054010 /// XM_231251	3q11	1.51	0.002
1398933_at	HIG1 domain family, member 2A (predicted)	Higd2a_predicted	2565	1949	2947	NM_001106102 /// XM_001070385 /// XM_214433	17p14	1.51	0.002
1369027_at	alpha 1,4-galactosyltransferase	A4galt	958	2643	3995	NM_022240	7q34	1.51	0.001
1393915_at	membrane bound O-acyltransferase domain containing 5	Mboat5	1067	2927	4418	NM_001012189	4q42	1.51	0.004
1368254_a_at	sphingosine kinase 1	Sphk1	218	883	1333	NM_133386	10q32.3	1.51	0.013

Table S5. PI3K signaling: negatively regulated genes

Affymetrix ID	Gene Title	Gene Symbol	Stem Ave	Dif Ave	LY Ave	GenBank	Chromosomal Location	dif/ly	t-test
1390384_at	similar to Histone H2A.x (H2a/x) (predicted)	RGD1566119_predicted	2443	1062	1602	NM_001109291 /// XM_001066891 /// XM_576399	8q22	1.51	0.006
1379028_at	sperm associated antigen 7 (predicted)	Spag7_predicted	1209	737	1112	NM_001107016 /// XM_001079933 /// XM_220574	10q24	1.51	0.034
1372131_at	ubiquilin 2 (predicted)	Ubqln2_predicted	781	1120	1687	NM_001108251 /// XM_001061090 /// XM_228806	Xq14	1.51	0.034
1387020_at	cytochrome P450, subfamily 51	Cyp51	338	648	976	NM_012941	4	1.51	0.003
1370288_a_at	tropomyosin 1, alpha	Tpm1	1074	966	1453	NM_001034068 /// NM_001034069 /// NM_001034070 /// NM_001034071 /// NM_001034072 /// NM_001034073 /// NM_001034074 /// NM_001034075 /// NM_019131	8q24	1.50	0.002
1370282_at	cysteine and glycine-rich protein 2	Csrp2	737	568	854	NM_177425	7q13	1.50	0.002
1390526_at	kelch-like 9 (Drosophila) (predicted)	Klhl9_predicted	1168	1847	2775	NM_001107944 /// XM_001054585 /// XM_233157	5q32	1.50	0.023
1372423_at	PERP, TP53 apoptosis effector (predicted)	Perp_predicted	1991	1815	2724	NM_001106265 /// XM_001072627 /// XM_214953	1p12	1.50	0.005
1368100_at	phosphate cytidylyltransferase 2, ethanolamine	Pcyt2	695	938	1408	NM_053568	10q32.3	1.50	0.002
1393267_at	PC4 and SFRS1 interacting protein 1	Psip1	2077	3984	5977	NM_175765	5q31	1.50	0.049
1376004_at	Transcribed locus	---	1305	1345	2018	BF285297	---	1.50	0.003
1388575_at	similar to Opa-interacting protein 5 (predicted)	RGD1564263_predicted	1667	2001	3002	AA943740	---	1.50	0.012
1390032_at	RNA binding motif, single stranded interacting protein 2	Rbms2	2009	2100	3149	NM_001025403	7q11	1.50	0.013
