# Supplementary material for: Phosphatidylinositol 3 kinase modulation of trophoblast cell differentiation
Source: BMC Dev Biol. 2010 Sep 14;10:97. doi: 10.1186/1471-213X-10-97 (PMC2944162; doi:10.1186/1471-213X-10-97)
Supplement: Additional file 6 — Table S6: PI3K signaling: positively regulated genes [file 1471-213X-10-97-S6.PDF]

Table S6. PI3K signaling: positively regulated genes

Affymetrix ID	Gene Title	Gene Symbol	Stem Ave	Dif Ave	LY Ave	GenBank	Chromosomal Location	dif/ly	t-test
1387219_at	adrenomedullin	Adm	229	1327	92	NM_012715	1q33	14.40	0.009
1390511_at	---	---	10	1863	161	BI285801	1	11.59	0.009
1378534_at	similar to brain carcinoembryonic antigen	LOC308394	10	1746	153	NM_001025679	1q21	11.44	0.001
1370874_at	Prolactin family 3, subfamily b, member 1	Prl3b1	1556	13469	1742	NM_012535	17p11	7.73	0.000
1376934_x_at	---	---	53	2146	298	BI285801	1	7.19	0.000
1393449_at	junction adhesion molecule 2	Jam2	585	1167	180	NM_001034004	11q11	6.48	0.003
1369029_at	phospholipid scramblase 1	Plscr1	20	2050	328	NM_057194	8q31	6.25	0.049
1387123_at	cytochrome P450, family 17, subfamily a, polypeptide 1	Cyp17a1	413	4444	974	NM_012753	1q55	4.57	0.000
1383197_at	junction adhesion molecule 2	Jam2	812	1759	391	NM_001034004	11q11	4.49	0.005
1370852_at	spleen protein 1 precursor	LOC171573	692	20968	4736	NM_138537	8q21	4.43	0.008
1370471_at	prolactin family 6, subfamily a, member 1	Prl6a1	102	1481	357	NM_022176 /// XM_001069642 /// XM_341525	17p11	4.15	0.000
1367809_at	prolactin family 4, subfamily a, member 1	Prl4a1	683	22573	5771	NM_017036	17p11	3.91	0.011
1376845_at	putative ISG12(b) protein	isg12(b)	158	4110	1061	NM_206846 /// XM_001068101 /// XM_238467	6q32	3.87	0.040
1383765_at	similar to RIKEN cDNA 4921520P21	MGC114388	36	863	229	NM_001025288	---	3.76	0.000
1367571_a_at	insulin-like growth factor 2	Igf2	3273	14329	3836	NM_031511 /// XM_001064965	1q41	3.74	0.001
1369166_at	matrix metallopeptidase 9	Mmp9	245	1209	331	NM_031055	3q42	3.66	0.000
1374488_at	GRAM domain containing 1B (predicted)	Gramd1b_predicted	89	2514	697	XM_001059472 /// XM_217113	8q22	3.61	0.013
1367733_at	carbonic anhydrase 2	Ca2	340	1935	551	NM_019291	2q23	3.51	0.012
1382690_at	carcinoembryonic antigen gene family 4	Cgm4	81	2729	800	NM_012525	1q21	3.41	0.004
1373282_at	similar to mitochondrial carrier protein MGC4399	LOC691431	703	1234	363	XM_001078245	5q36	3.40	0.001
1370950_at	phosphatidic acid phosphatase type 2B	Ppap2b	164	922	272	NM_138905	5q34	3.39	0.008
1370281_at	fatty acid binding protein 5, epidermal	Fabp5	9936	6954	2153	NM_145878	---	3.23	0.001
1398275_at	matrix metallopeptidase 9	Mmp9	530	2120	683	NM_031055	3q42	3.11	0.000
1372101_at	phosphatidic acid phosphatase type 2B	Ppap2b	462	2245	733	NM_138905	5q34	3.06	0.001
1387587_at	Fas ligand (TNF superfamily, member 6)	Faslg	1816	6909	2259	NM_012908	13q22	3.06	0.000
1370869_at	branched chain aminotransferase 1, cytosolic	Bcat1	1480	1819	595	NM_017253	4q44	3.06	0.000
1390525_a_at	stimulated by retinoic acid gene 6 homolog (mouse)	Stra6	28	1037	342	NM_001029924	8q24	3.03	0.005
1369520_a_at	branched chain aminotransferase 1, cytosolic	Bcat1	645	891	294	NM_017253	4q44	3.03	0.002
1367651_at	cathepsin D	Ctsd	2606	10770	3585	NM_134334	1q41	3.00	0.000
1386922_at	carbonic anhydrase 2	Ca2	244	1363	479	NM_019291	2q23	2.85	0.010
1389856_at	carcinoembryonic antigen gene family 4	Cgm4	57	5073	1819	NM_012525	1q21	2.79	0.004
1369957_at	regulator of G-protein signaling 5	Rgs5	136	1287	462	NM_019341	13q24	2.78	0.005
1372665_at	phosphoserine aminotransferase 1	Psat1	10633	7071	2576	NM_198738	1q43	2.75	0.000

Table S6. PI3K signaling: positively regulated genes

Affymetrix ID	Gene Title	Gene Symbol	Stem Ave	Dif Ave	LY Ave	GenBank	Chromosomal Location	dif/ly	t-test
1392981_at	Iroquois related homeobox 4 (Drosophila) (predicted)	Irx4_predicted	3397	816	298	NM_001107330 /// XM_001057125 /// XM_001057181 /// XM_001057247 /// XM_225068	17p14	2.74	0.001
1372601_at	activating transcription factor 5	Atf5	521	1345	494	NM_172336	1q22	2.72	0.000
1379275_at	sorting nexin 10	Snx10	373	1259	481	NM_001013085	4q24	2.62	0.001
1377264_at	interleukin 17F	Il17f	179	4036	1546	NM_001015011	9q13	2.61	0.005
1391051_at	Transcribed locus	---	9250	1378	541	AA997873	5	2.55	0.049
1370695_s_at	tribbles homolog 3 (Drosophila)	Trib3	293	2284	899	NM_144755	3q41	2.54	0.000
1383987_at	Transcribed locus	---	1153	970	389	AI112807	---	2.49	0.036
1389210_at	lymphocyte cytosolic protein 1	Lcp1	1546	2976	1209	NM_001012044	15q11	2.46	0.011
1387747_at	gap junction membrane channel protein beta 3	Gjb3	5356	810	331	NM_019240	5q36	2.45	0.028
1373504_at	GLI pathogenesis-related 1 (glioma)	Glipr1	131	971	403	NM_001011987	7q21	2.41	0.006
1377880_at	Transcribed locus	---	292	1820	762	AI170633	---	2.39	0.000
1370817_at	Sec11-like 3 (S. cerevisiae)	Sec11l3	1299	1080	463	NM_153628	18q12.1	2.33	0.003
1394363_at	RIO kinase 3 (yeast) (predicted)	Riok3_predicted	825	1997	898	NM_001108423 /// XM_001072670 /// XM_341578	18p13	2.22	0.000
1376036_at	transporter	LOC314323	29	875	397	NM_199109	6q31	2.20	0.002
1388703_at	endothelial cell adhesion molecule	Esam	160	1748	793	NM_001004245	8q21	2.20	0.034
1369141_at	Prolactin family 3, subfamily d, member 1 /// Prolactin family 3, subfamily d, member 2	Prl3d1 /// Prl3d2	10452	22492	10213	NM_001083940 /// NM_017363 /// XM_001078794	17p11	2.20	0.000
1387156_at	hydroxysteroid (17-beta) dehydrogenase 2	Hsd17b2	507	5354	2444	NM_024391	19q12	2.19	0.010
1375071_at	nucleoporin 133 (predicted)	Nup133_predicted	1476	830	379	XM_001053507	19q12	2.19	0.003
1384769_a_at	similar to Zinc finger X-linked protein ZXDB (predicted) /// similar to Zinc finger X-linked protein ZXDB	LOC683508 /// LOC683516 /// RGD1563355_predicted	274	1434	657	XM_001066253 /// XM_001066304 /// XM_234140	Xq31	2.18	0.001
1384865_at	Transcribed locus, strongly similar to XP_346369.1 PREDICTED: similar to leucine zipper, down-regulated in cancer 1 [Rattus norvegicus]	---	1759	5568	2605	AW535518	X	2.14	0.016
1373418_at	glutamyl-prolyl-tRNA synthetase	Eprs	3871	6850	3216	NM_001024238	13q26	2.13	0.001
1386662_at	similar to Sestrin 2 (Hi95) (predicted)	RGD1566319_predicted	318	1090	515	NM_001109358 /// XM_001065176 /// XM_001065235 /// XM_001065290 /// XM_578496	5q36	2.12	0.001
1386321_s_at	tribbles homolog 3 (Drosophila)	Trib3	695	3032	1449	NM_144755	3q41	2.09	0.007
1393983_at	exportin, tRNA (nuclear export receptor for tRNAs) (predicted)	Xpot_predicted	2069	2580	1235	NM_001108102 /// XM_001079263 /// XM_235185	7q22	2.09	0.000
1370080_at	heme oxygenase (decycling) 1	Hmox1	3614	4953	2373	NM_012580	19p11	2.09	0.000

Table S6. PI3K signaling: positively regulated genes

Affymetrix ID	Gene Title	Gene Symbol	Stem Ave	Dif Ave	LY Ave	GenBank	Chromosomal Location	dif/ly	t-test
1371818_at	exportin, tRNA (nuclear export receptor for tRNAs) (predicted)	Xpot_predicted	2596	2913	1414	AW251204	7	2.06	0.001
1376620_at	Transcribed locus	---	669	1505	731	AI137912	---	2.06	0.004
1375962_at	Transcribed locus	---	775	3694	1824	BE102925	---	2.03	0.049
1370422_at	receptor-interacting serine-threonine kinase 3	Ripk3	2417	1710	847	NM_139342	15p13	2.02	0.000
1370694_at	tribbles homolog 3 (Drosophila)	Trib3	266	1176	584	NM_144755	3q41	2.01	0.001
1379396_at	engulfment and cell motility 1, ced-12 homolog (C. elegans) (predicted)	Elmo1_predicted	116	804	402	NM_001108415 /// XM_001059750 /// XM_341532	17q11	2.00	0.004
1398247_at	protease, serine, 15	Prss15	3109	4523	2279	NM_133404	9q11	1.98	0.001
1380743_at	similar to Cat eye syndrome critical region protein 2 (predicted)	RGD1564182_predicted	742	1170	591	BE116508	---	1.98	0.038
1395699_at	RIO kinase 3 (yeast) (predicted)	Riok3_predicted	1134	2186	1106	NM_001108423 /// XM_001072670 /// XM_341578	18p13	1.98	0.004
1389659_at	similar to ctla-2-beta protein (141 AA) (predicted)	RGD1565540_predicted	5855	3066	1552	NM_001109115 /// XM_001065725 /// XM_573975	17p14	1.98	0.002
1383585_s_at	sorting nexin 10	Snx10	658	1630	828	AI043753	4	1.97	0.000
1372808_at	similar to Bifunctional methylenetetrahydrofolate dehydrogenase/cyclohydrolase, mitochondrial precursor	LOC680308	1993	3873	1981	NM_001109398 /// XM_001056154 /// XM_001056213 /// XM_001067863 /// XM_001067912	4q34	1.96	0.001
1376660_at	Transcribed locus	---	526	900	461	BG672127	---	1.95	0.007
1384963_at	similar to Cat eye syndrome critical region protein 2 (predicted)	RGD1564182_predicted	1634	2383	1220	BI277632	---	1.95	0.017
1377923_at	START domain containing 8 (predicted)	Stard8_predicted	324	1165	604	NM_001107849 /// XM_001068833 /// XM_231459	Xq31	1.93	0.008
1391012_at	Transcribed locus	---	266	955	495	BF283210	---	1.93	0.001
1383455_at	glutamyl-prolyl-tRNA synthetase	Eprs	4207	6957	3618	NM_001024238	13q26	1.92	0.001
1370848_at	solute carrier family 2 (facilitated glucose transporter), member 1	Slc2a1	4906	5678	2957	NM_138827	5q36.1	1.92	0.000
1368025_at	DNA-damage-inducible transcript 4	Ddit4	814	2917	1520	NM_080906	20q11	1.92	0.003
1382500_at	similar to Sestrin 2 (Hi95) (predicted)	RGD1566319_predicted	772	2198	1150	NM_001109358 /// XM_001065176 /// XM_001065235 /// XM_001065290 /// XM_578496	5q36	1.91	0.001
1374113_at	bromodomain adjacent to zinc finger domain, 1A (predicted)	Baz1a_predicted	4784	2343	1228	XM_001079067 /// XM_234156	6q23	1.91	0.000
1372145_at	threonyl-tRNA synthetase	Tars	2951	3492	1832	NM_001006976	2q16	1.91	0.000
1370845_at	ectonucleoside triphosphate diphosphohydrolase 2	Entpd2	602	842	442	NM_172030	3p13	1.90	0.009
1372812_at	similar to mKIAA0256 protein (predicted)	RGD1559930_predicted	384	1008	534	XM_001077702 /// XM_215843	3q36	1.89	0.005

Table S6. PI3K signaling: positively regulated genes

Affymetrix ID	Gene Title	Gene Symbol	Stem Ave	Dif Ave	LY Ave	GenBank	Chromosomal Location	dif/ly	t-test
1391607_at	similar to Sestrin 2 (Hi95) (predicted)	RGD1566319_predicted	1017	2739	1453	NM_001109358 /// XM_001065176 /// XM_001065235 /// XM_001065290 /// XM_578496	5q36	1.89	0.000
1389450_at	similar to Williams-Beuren syndrome critical region protein 22 /// similar to Putative methyltransferase WBSCR22 (Williams-Beuren syndrome chromosome region 22 protein homolog)	LOC360830 /// LOC368084	4106	1834	973	XM_001071583 /// XM_341103 /// XM_347254	12q12	1.88	0.015
1388574_at	tryptophanyl-tRNA synthetase	Wars	1687	2051	1091	NM_001013170	6q32	1.88	0.001
1384449_at	Transcribed locus	---	942	868	464	AA964588	---	1.87	0.000
1370379_at	protease, serine, 8 (prostasin)	Prss8	264	1173	628	NM_138836	1q36	1.87	0.003
1393796_at	centrosomal protein 152 (predicted)	Cep152_predicted	676	928	497	XM_230555 /// XR_009444	3q36	1.87	0.001
1369590_a_at	DNA-damage inducible transcript 3	Ddit3	582	3287	1764	NM_001109986 /// NM_024134	7q22	1.86	0.046
1372642_at	Transcribed locus	---	3545	2111	1135	BE113397	---	1.86	0.002
1370371_a_at	CEA-related cell adhesion molecule 1 /// CEA-related cell adhesion molecule 10	Ceacam1 /// Ceacam10	514	4941	2662	NM_001033860 /// NM_001033861 /// NM_001033862 /// NM_031755 /// NM_173339	1q21 /// 1q21-q22	1.86	0.044
1371040_at	solute carrier family 1 (neutral amino acid transporter), member 5	Slc1a5	1286	1488	802	NM_175758	1q21	1.86	0.010
1386904_a_at	cytochrome b-5	Cyb5	3520	3893	2105	NM_022245	18q12.3	1.85	0.001
1368623_at	CEA-related cell adhesion molecule 9	Ceacam9	618	8892	4815	NM_053919	1q21	1.85	0.004
1387653_at	translin-associated factor X	Tsnax	606	1280	697	NM_022262	19	1.84	0.002
1395124_at	tryptophanyl-tRNA synthetase	Wars	789	1009	550	NM_001013170	6q32	1.84	0.000
1389554_at	nuclear receptor subfamily 2, group F, member 2	Nr2f2	532	2593	1413	AA956784	1	1.83	0.001
1367841_a_at	prolactin family 8, subfamily a, member 9	Prl8a9	402	4135	2260	NM_134385	17p11	1.83	0.010
1382040_at	glutamyl-prolyl-tRNA synthetase	Eprs	2218	4085	2252	NM_001024238	13q26	1.81	0.010
1372009_at	tyrosyl-tRNA synthetase	Yars	4596	5405	2981	NM_001025696	5q36	1.81	0.001
1388519_at	Sec61 beta subunit (predicted)	Sec61b_predicted	2830	3571	1973	NM_001106654 /// XM_001056613 /// XM_216400	5q22	1.81	0.003
1388695_at	serine hydroxymethyltransferase 2 (mitochondrial)	Shmt2	5493	3122	1724	NM_001008322	7q22	1.81	0.001
1368308_at	myelocytomatosis viral oncogene homolog (avian)	Myc	1806	4355	2410	NM_012603	7q33	1.81	0.016
1369525_at	GATA binding protein 3	Gata3	2657	2930	1623	NM_133293	17q12.3	1.81	0.005
1395730_at	glycyl-tRNA synthetase /// similar to Glycyl-tRNA synthetase (predicted)	Gars /// RGD1559871_predicted	1032	1325	736	XM_001061006 /// XM_216152 /// XM_575487	4q24	1.80	0.014
1398771_at	solute carrier family 3 (activators of dibasic and neutral amino acid transport), member 2	Slc3a2	3741	9339	5189	NM_019283	1q43	1.80	0.000
1392547_at	hypothetical LOC302884	MGC105649	352	2391	1334	NM_001008518	3q35	1.79	0.027

Table S6. PI3K signaling: positively regulated genes

Affymetrix ID	Gene Title	Gene Symbol	Stem Ave	Dif Ave	LY Ave	GenBank	Chromosomal Location	dif/ly	t-test
1375964_at	phosphoserine phosphatase	Psph	1055	1824	1022	NM_001009679	12q13	1.78	0.000
1371731_at	Similar to Coatomer gamma-2 subunit (Gamma-2 coat protein) (Gamma-2 COP) (predicted)	RGD1566215_predicted	4385	3128	1756	NM_001106929 /// XM_001065495 /// XM_217496	4q22	1.78	0.018
1377998_at	coproporphyrinogen oxidase	Cpox	1820	989	555	NM_001037095	11q12	1.78	0.006
1383974_at	E74-like factor 5 (predicted)	Elf5_predicted	8631	6083	3416	NM_001108956 /// XM_001079426 /// XM_345409	3q32	1.78	0.012
1382901_at	growth factor independent 1B (predicted)	Gfi1b_predicted	338	924	520	NM_001107823 /// XM_001079155 /// XM_231109	3p12	1.78	0.020
1368842_at	transcription factor 4	Tcf4	592	1329	748	NM_053369	18q12.1	1.78	0.040
1372661_at	transducin (beta)-like 3	Tbl3	1417	1134	642	NM_001008277	10q12	1.77	0.000
1386900_at	ribosome associated membrane protein 4	RAMP4	3143	4778	2704	NM_030835	2q31	1.77	0.012
1388715_at	glycyl-tRNA synthetase	Gars	4334	5182	2933	XM_001061006 /// XM_216152	4q24	1.77	0.007
1391279_at	Scinderin	Scin	1137	3965	2250	NM_198748	6q21	1.76	0.042
1370000_at	nucleobindin 2	Nucb2	675	1200	685	NM_021663	1q35	1.75	0.001
1368996_at	carcinoembryonic antigen-related cell adhesion molecule 3	Ceacam3	202	17879	10229	NM_012702	1q21	1.75	0.027
1390447_at	Transcribed locus	---	898	1114	640	BI295918	1	1.74	0.014
1371709_at	mitochondrial ribosomal protein L3 (predicted)	Mrpl3_predicted	3662	2838	1632	NM_001106852 /// XM_001072824 /// XM_217239	8q32	1.74	0.000
1389573_at	ChaC, cation transport regulator-like 1 (E. coli) (predicted)	Chac1_predicted	422	830	478	XM_001080834 /// XM_342497	3q35	1.74	0.016
1377016_at	cysteine-rich with EGF-like domains 2	Creld2	1618	1846	1063	NM_001037208	7q34	1.74	0.007
1377644_at	similar to RIKEN cDNA 4921524J17 (predicted)	RGD1308706_predicted	1886	5051	2910	XM_001068411 /// XM_214649	19q11	1.74	0.006
1374118_at	leucyl-tRNA synthetase	Lars	3031	4253	2452	NM_001009637	18p11	1.73	0.004
1374329_at	mcf.2 transforming sequence-like	Mcf2l	987	1064	615	BI286364	---	1.73	0.031
1388426_at	sterol regulatory element binding factor 1	Srebf1	870	2288	1322	XM_001075680 /// XM_213329	10q22	1.73	0.038
1387282_at	heat shock 22kDa protein 8	Hspb8	91	935	543	NM_053612	12q16	1.72	0.021
1389782_at	similar to RIKEN cDNA 2010107G23 (predicted) /// hypothetical protein LOC679430	LOC679430 /// RGD1305587_predicted	1761	1531	895	XM_001056304 /// XM_215413	20q11	1.71	0.047
1373448_at	acylphosphatase 1, erythrocyte (common) type (predicted)	Acyp1_predicted	1172	858	502	NM_001106746 /// XM_001060176 /// XM_216757	6q31	1.71	0.025
1368227_at	solute carrier family 28 (sodium-coupled nucleoside transporter), member 2	Slc28a2	240	5743	3359	NM_031664	3q35	1.71	0.001
1373035_at	---	---	695	860	504	AI031032	1	1.71	0.002

Table S6. PI3K signaling: positively regulated genes

Affymetrix ID	Gene Title	Gene Symbol	Stem Ave	Dif Ave	LY Ave	GenBank	Chromosomal Location	dif/ly	t-test
1379560_at	nuclear receptor binding SET domain protein 1 (predicted) /// similar to Histone-lysine N-methyltransferase, H3 lysine-36 and H4 lysine-20 specific (H3-K36-HMTase) (H4-K20-HMTase) (Nuclear receptor binding SET domain containing protein 1) (NR-binding SET domain containing protein)	LOC686060 /// Nsd1_predicted	594	1251	732	NM_001107337 /// XM_001066374 /// XM_001066409 /// XM_225168	17p14	1.71	0.014
1371883_at	monocyte to macrophage differentiation-associated	Mmd	847	3656	2142	NM_001007673 /// XM_001081244	10q26	1.71	0.004
1391572_at	cysteinyl-tRNA synthetase (predicted)	Cars_predicted	1091	2361	1383	NM_001106319 /// XM_001065753 /// XM_215134	1q41	1.71	0.000
1375441_at	seryl-aminoacyl-tRNA synthetase 1	Sars1	4173	6504	3829	NM_001007606	2q34	1.70	0.012
1390208_at	HIV-1 tat interactive protein 2, homolog (human) (predicted)	Htatip2_predicted	753	1416	840	NM_001106263 /// XM_001080298 /// XM_214927	1q22	1.69	0.007
1367815_at	solute carrier family 5 (sodium-dependent vitamin transporter), member 6	Slc5a6	1571	1738	1032	NM_130746	6q14	1.69	0.006
1389381_at	sequestosome 1	Sqstm1	7322	9069	5388	NM_175843 /// NM_181550	10q21	1.68	0.004
1394705_at	Transcribed locus	---	1590	1858	1104	BE117266	---	1.68	0.001
1381118_at	similar to RIKEN cDNA 2700007P21	RGD1311463	461	847	505	NM_001014045	3q33	1.68	0.000
1390406_at	Rho GTPase activating protein 18 (predicted)	Arhgap18_predicted	434	4093	2444	NM_001106354 /// XM_001062453 /// XM_215247	1p12	1.67	0.002
1380201_at	nucleolar protein 10	Nol10	1808	1647	986	NM_001014076	6q16	1.67	0.002
1371574_at	growth hormone inducible transmembrane protein	Ghitm	6548	9016	5401	NM_001005908	16p14	1.67	0.010
1372230_at	transmembrane protein 147	Tmem147	4009	3597	2164	NM_001038494	1q21	1.66	0.011
1389594_at	similar to Protein C20orf22 homolog	LOC499913	311	1557	941	NM_001024314	3q41	1.65	0.005
1370073_at	DnaJ (Hsp40) homolog, subfamily C, member 3	Dnajc3	740	1017	615	NM_022232	15q24	1.65	0.005
1397584_at	Transcribed locus	---	1413	988	599	BF289504	---	1.65	0.001
1374437_at	asparaginyl-tRNA synthetase	Nars	9640	13264	8080	NM_001025635	18q12.1	1.64	0.009
1389733_at	---	Mars_predicted	3160	3829	2335	BM384125	7	1.64	0.006
1385073_at	similar to SERTA domain containing 4 (predicted)	RGD1564561_predicted	5174	3195	1952	AI145639	6	1.64	0.008
1373142_at	Growth hormone inducible transmembrane protein	Ghitm	2283	3952	2416	NM_001005908	16p14	1.64	0.002
1373549_at	DEAD (Asp-Glu-Ala-Asp) box polypeptide 10 (predicted)	Ddx10_predicted	1567	1196	732	NM_001106820 /// XM_001071986 /// XM_236263	8q24	1.63	0.005
1379785_at	Transcribed locus	---	421	1052	644	BI275894	---	1.63	0.012
1384368_at	Transcribed locus	---	581	867	531	AA900084	---	1.63	0.001
1395363_at	methionine-tRNA synthetase (predicted)	Mars_predicted	1126	1601	982	AI102047	7	1.63	0.003
1367741_at	homocysteine-inducible, endoplasmic reticulum stress-inducible, ubiquitin-like domain member 1	Herpud1	642	2781	1710	NM_053523	19p12	1.63	0.019

Table S6. PI3K signaling: positively regulated genes

Affymetrix ID	Gene Title	Gene Symbol	Stem Ave	Dif Ave	LY Ave	GenBank	Chromosomal Location	dif/ly	t-test
1383831_at	glutaminyl-tRNA synthase (glutamine-hydrolyzing)-like 1	Qrsl1	1361	1421	876	NM_001014034 /// XM_001067712	20q13	1.62	0.001
1370252_at	arginine vasopressin-induced 1	Avpi1	2688	2451	1514	NM_134373	1q54	1.62	0.030
1369068_at	cullin 5	Cul5	722	882	546	NM_022683	8q24	1.62	0.001
1389760_at	polycomb group ring finger 6	Pcgf6	3357	2034	1259	NM_001013154	1q54	1.62	0.000
1367577_at	heat shock 27kDa protein 1	Hspb1	97	940	583	NM_031970	12q12	1.61	0.045
1372451_at	Trf (TATA binding protein-related factor)-proximal protein homolog (Drosophila) /// similar to ubiquitin specific protease 49 (predicted)	LOC501098 /// Trfp	1261	1776	1103	NM_001013178 /// XM_576513	9q12	1.61	0.012
1375343_at	Transcribed locus	---	1066	2755	1715	BE116572	---	1.61	0.038
1372876_at	selenophosphate synthetase 2	Sephs2	2072	1028	641	NM_001079889 /// XM_001080177 /// XM_219347	1q36	1.60	0.040
1367484_at	ubiquitin-conjugating enzyme E2E 2 (UBC4/5 homolog, yeast)	Ube2e2	3804	2522	1573	NM_001108371 /// XM_001067024 /// XM_341288	15p16	1.60	0.012
1391422_at	DnaJ (Hsp40) homolog, subfamily B, member 6	Dnajb6	833	931	581	NM_001013209	4q11	1.60	0.002
1392475_at	Transcribed locus	---	638	1396	872	BE104169	---	1.60	0.003
1371828_at	similar to RIKEN cDNA 1500011H22	RGD1310861	1265	1727	1078	NM_001008290	12q16	1.60	0.001
1383715_at	similar to hypothetical protein D4Ertd89e	RGD1305703	352	1931	1210	NM_001014191	5q24	1.60	0.028
1379337_at	similar to RIKEN cDNA 2810421I24	RGD1307883	2301	1622	1017	NM_001009673	9q32	1.59	0.022
1374034_at	cysteinyl-tRNA synthetase (predicted)	Cars_predicted	1339	2750	1725	NM_001106319 /// XM_001065753 /// XM_215134	1q41	1.59	0.000
1389151_at	Transcribed locus	---	2146	1540	969	AI103440	---	1.59	0.007
1370688_at	glutamate-cysteine ligase, catalytic subunit	Gclc	1317	6423	4040	NM_012815	8q31	1.59	0.000
1388105_at	D123 gene product	D123	3265	2617	1649	NM_053877	17q12.3	1.59	0.000
1382044_at	hypothetical protein LOC498796	LOC498796	397	1187	748	NM_001025034	17q12.3	1.59	0.026
1370134_at	solute carrier family 33 (acetyl-CoA transporter), member 1	Slc33a1	882	1294	816	NM_022252	2q31	1.59	0.006
1384115_at	Mitochondrial acyl-CoA thioesterase 1	Mte1	1406	1686	1065	NM_138907	6q31	1.58	0.000
1398312_s_at	solute carrier family 14 (urea transporter), member 2	Slc14a2	473	2020	1278	NM_001110270 /// NM_019347 /// NM_177962	18q12.3	1.58	0.024
1368037_at	carbonyl reductase 1	Cbr1	1858	1880	1191	NM_019170	11q11	1.58	0.001
1376039_at	aurora kinase A	Aurka	3166	2300	1462	NM_153296	3q42	1.57	0.029
1398384_at	exosome component 9	Exosc9	1838	1224	779	NM_001025406	2q25	1.57	0.007
1373556_at	similar to CG14903-PA (predicted)	RGD1561792_predicted	1923	1271	810	XM_001070434 /// XM_216648	6q14	1.57	0.016
1373053_at	---	---	6035	4042	2580	AI169620	---	1.57	0.006
1373841_at	rac/cdc42 guanine nucleotide exchange factor 6	arhgef6	129	2219	1418	AI177431	X	1.57	0.004
1376418_a_at	isoleucine-tRNA synthetase (predicted)	Iars_predicted	3654	3950	2526	XM_001055989 /// XM_225196	17p14	1.56	0.000
1390448_at	similar to 1110065L07Rik protein (predicted)	RGD1308317_predicted	592	955	612	NM_001107327 /// XM_001076411 /// XM_225044	16q12.5	1.56	0.002

Table S6. PI3K signaling: positively regulated genes

Affymetrix ID	Gene Title	Gene Symbol	Stem Ave	Dif Ave	LY Ave	GenBank	Chromosomal Location	dif/ly	t-test
1369962_at	5-aminoimidazole-4-carboxamide ribonucleotide formyltransferase/IMP cyclohydrolase	Atic	6392	2340	1502	NM_031014	9q33	1.56	0.006
1378134_at	ATPase, Class I, type 8B, member 1 (predicted)	Atp8b1_predicted	338	4260	2735	NM_001106140 /// XM_001064261 /// XM_214553	18q12.1	1.56	0.003
1371380_at	pyruvate dehydrogenase E1 alpha 1 /// pyruvate dehydrogenase E1 alpha 1 pseudogene	LOC685778 /// Pdha1	1065	1947	1250	NM_001004072 /// XM_001060860 /// XM_001060918 /// XM_001067950 /// XM_001067997	16q12.5 /// Xq21	1.56	0.030
1389353_at	sema domain, transmembrane domain (TM), and cytoplasmic domain, (semaphorin) 6D (predicted)	Sema6d_predicted	496	4154	2670	NM_001107768 /// XM_001077367 /// XM_230583	3q35	1.56	0.038
1393845_a_at	transmembrane channel-like gene family 4	Tmc4	278	982	632	NM_001034104	1q12	1.55	0.006
1383698_at	pyruvate dehydrogenase E1 alpha 1	Pdha1	518	1082	697	NM_001004072	Xq21	1.55	0.015
1370030_at	glutamate cysteine ligase, modifier subunit	Gclm	3435	1389	897	NM_017305	2q41	1.55	0.014
1389146_at	hypothetical protein LOC498796	LOC498796	2467	5559	3597	NM_001025034	17q12.3	1.55	0.006
1375230_at	Endogenous retrovirus mRNA, partial sequence	---	5919	2957	1913	AA800192	---	1.55	0.001
1372523_at	glutamate-cysteine ligase, catalytic subunit	Gclc	1617	7445	4819	NM_012815	8q31	1.54	0.000
1371955_at	mitochondrial ribosomal protein L35 (predicted)	Mrpl35_predicted	2042	1082	701	NM_001106596 /// XM_001063053 /// XM_216169	4q33	1.54	0.043
1382309_at	Transcribed locus, moderately similar to XP_238213.3 PREDICTED: similar to delangin [Rattus norvegicus]	---	897	1180	765	BI286390	---	1.54	0.003
1380521_at	hematopoietic cell specific Lyn substrate 1	Hcls1	581	813	527	NM_001011898	11q22	1.54	0.004
1368868_at	A kinase (PRKA) anchor protein (gravin) 12	Akap12	1013	809	525	NM_001033653 /// NM_057103	1p11	1.54	0.017
1369737_at	cAMP responsive element modulator	Crem	412	951	619	NM_001110860 /// NM_013086 /// NM_017334	17q12.1	1.54	0.024
1371051_at	glutamate receptor, ionotropic, N-methyl D-aspartate-like 1A	Grinl1a	2927	2065	1347	NM_183402	8q24	1.53	0.000
1388394_at	alanyl-tRNA synthetase	Aars	4267	5166	3377	XM_001077503 /// XM_214690	19q12	1.53	0.034
1392446_at	kelch-like 2, Mayven (Drosophila) (predicted)	Klhl2_predicted	2191	1034	676	XM_001073589 /// XM_214331	16p13	1.53	0.005
1388721_at	heat shock 22kDa protein 8	Hspb8	210	2061	1349	NM_053612	12q16	1.53	0.009
1387800_at	Fas death domain-associated protein	Daxx	1011	1688	1105	NM_080891	20p12	1.53	0.002
1367624_at	activating transcription factor 4	Atf4	9715	16615	10882	NM_024403	7q34	1.53	0.003
1390508_at	Transcribed locus	---	1323	1674	1098	BI284393	---	1.52	0.021
1388331_at	tumor rejection antigen gp96 (predicted)	Tra1_predicted	6438	5444	3575	NM_001012197	7q13	1.52	0.041

Table S6. PI3K signaling: positively regulated genes

Affymetrix ID	Gene Title	Gene Symbol	Stem Ave	Dif Ave	LY Ave	GenBank	Chromosomal Location	dif/ly	t-test
1376754_at	cysteinyl-tRNA synthetase (predicted)	Cars_predicted	920	1916	1259	NM_001106319 /// XM_001065753 /// XM_215134	1q41	1.52	0.000
1387065_at	phospholipase C, delta 4	Plcd4	906	2990	1969	NM_080688	9q33	1.52	0.015
1370690_at	heat shock 70kDa protein 9A (predicted)	Hspa9a_predicted	6543	6718	4426	NM_001100658 /// XM_001062585 /// XM_214583	18p12	1.52	0.011
1374503_at	pre B-cell leukemia transcription factor 3 (predicted)	Pbx3_predicted	1329	1378	909	NM_001107834 /// XM_001078717 /// XM_001078726 /// XM_001078743 /// XM_001078759 /// XM_231158	3p11	1.52	0.044
1368848_at	lectin, mannose-binding, 1	Lman1	1274	1449	958	NM_053886	18q12.1	1.51	0.009
1372502_at	neurofibromatosis 2	Nf2	1017	943	623	NM_013193 /// XM_001067432 /// XM_341248	14q21	1.51	0.015
1388366_at	mitochondrial ribosomal protein L4 (predicted)	Mrpl4_predicted	2008	1224	809	NM_001108754 /// XM_001071006 /// XM_343354	8q13	1.51	0.003
1388792_at	growth arrest and DNA-damage-inducible 45 gamma	Gadd45g	1426	1806	1194	NM_001077640 /// XM_001053888 /// XM_237999	17p14	1.51	0.009
1370812_at	Bcl2-like 1	Bcl2l1	339	892	590	NM_001033670 /// NM_001033671 /// NM_001033672 /// NM_031535	3q41.2	1.51	0.050
1373115_at	similar to 2310061I09Rik protein (predicted)	RGD1304653_predicted	652	1341	888	BI281909	9	1.51	0.001
1372170_at	aminoacylase 1	Acy1	1923	1322	878	NM_001005383	8q32	1.51	0.005
1393645_at	hypothetical protein LOC317274	LOC317274	549	864	574	NM_001025013	Xq22	1.51	0.023
1378400_at	similar to RIKEN cDNA 4632404H22 (predicted)	RGD1560129_predicted	292	3703	2460	XM_001054191 /// XM_577043	Xq36	1.51	0.004
1380257_at	SMC5 structural maintenance of chromosomes 5-like 1 (yeast) (predicted)	Smc5l1_predicted	1009	1199	798	NM_001106357 /// XM_001078644 /// XM_215254	1q51	1.50	0.001
1393214_at	similar to hypothetical protein A230042K10 (predicted)	RGD1566144_predicted	1259	1116	743	BG371744	---	1.50	0.000
1398961_at	similar to ring finger protein 13	LOC681578	1271	1608	1072	NM_001109444 /// XM_001057588 /// XM_001057631 /// XM_001057755 /// XM_001057819 /// XM_001057871	---	1.50	0.000
